# Phylogeny and a structural model of plant MHX transporters

**DOI:** 10.1186/1471-2229-13-75

**Published:** 2013-05-02

**Authors:** Rachel Gaash, Meirav Elazar, Keren Mizrahi, Meital Avramov-Mor, Irina Berezin, Orit Shaul

**Affiliations:** 1 The Mina and Everard Goodman Faculty of Life Sciences, Bar-Ilan University, Ramat-Gan 5290002, Israel

**Keywords:** CaCA superfamily, Magnesium proton exchanger, MHX, NCX, Sodium calcium exchanger, Transporter, Vacuole, Zinc

## Abstract

**Background:**

The *Arabidopsis thaliana MHX* gene (*AtMHX*) encodes a Mg^2+^/H^+^ exchanger. Among non-plant proteins, AtMHX showed the highest similarity to mammalian Na^+^/Ca^2+^ exchanger (NCX) transporters, which are part of the Ca^2+^/cation (CaCA) exchanger superfamily.

**Results:**

Sequences showing similarity to *AtMHX* were searched in the databases or sequenced from cDNA clones. Phylogenetic analysis showed that the MHX family is limited to plants, and constitutes a sixth family within the CaCA superfamily. Some plants include, besides a full *MHX* gene, partial *MHX*-related sequences. More than one full *MHX* gene was currently identified only in *Oryza sativa* and *Mimulus guttatus*, but an EST for more than one *MHX* was identified only in *M. guttatus*. *MHX* genes are not present in the currently available chlorophyte genomes. The prevalence of upstream ORFs in *MHX* genes is much higher than in most plant genes, and can limit their expression. A structural model of the MHXs, based on the resolved structure of NCX1, implies that the MHXs include nine transmembrane segments. The MHXs and NCXs share 32 conserved residues, including a GXG motif implicated in the formation of a tight-turn in a reentrant-loop. Three residues differ between all MHX and NCX proteins. Altered mobility under reducing and non-reducing conditions suggests the presence of an intramolecular disulfide-bond in AtMHX.

**Conclusions:**

The absence of *MHX* genes in non-plant genomes and in the currently available chlorophyte genomes, and the presence of an *NCX* in *Chlamydomonas*, are consistent with the suggestion that the *MHXs* evolved from the *NCXs* after the split of the chlorophyte and streptophyte lineages of the plant kingdom. The *MHXs* underwent functional diploidization in most plant species. *De novo* duplication of *MHX* occurred in *O. sativa* before the split between the Indica and Japonica subspecies, and was apparently followed by translocation of one *MHX* paralog from chromosome 2 to chromosome 11 in Japonica. The structural analysis presented and the identification of elements that differ between the MHXs and the NCXs, or between the MHXs of specific plant groups, can contribute to clarification of the structural basis of the function and ion selectivity of MHX transporters.

## Background

A number of Mg^2+^ transport proteins have been identified in prokaryotic and eukaryotic organisms (reviewed in [1-4]). The bacterial CorA Mg^2+^ channel includes two transmembrane segments (TMSs) [3]. Homologs of CorA, termed Alr proteins, were found in the plasma membrane of yeast [3]. Human MRS2, a mitochondrial Mg^2+^ channel, shares many of the properties of the bacterial CorA and yeast Alr1 proteins (reviewed in [4]). Another family of bacterial Mg^2+^ transporters comprises the MgtA and MgtB proteins that have ten TMSs and are members of the superfamily of P-type ATPases (reviewed in [4]). Mammalian Mg^2+^ transporters of the SLC41 family share similarity with some regions of the bacterial MgtE transporters, which possess five TMSs (reviewed in [4]). The mammalian ancient conserved domain protein (ACDP) Mg^2+^ transporters were also identified in prokaryotes (reviewed in [4]). However, other mammalian transporters, including the TRPM6/7, MagT, NIPA, MMgT, and HIP14 families, were not identified in prokaryotic genomes (reviewed in [4]).

Four groups of proteins were shown to carry Mg^2+^ ions in plants (reviewed in [1,2,4]). The first group comprises transporters that are part of the CorA superfamily [3,5,6]. In *Arabidopsis thaliana*, this gene family has ten members, and the family was named MRS2 [5], or alternatively MGT [6]. The slow-vacuolar (SV) channel is a non-selective cation channel that can also carry Mg^2+^ ions (reviewed in [7]). The cyclic nucleotide-gated channel AtCNGC10 carries Ca^2+^ and Mg^2+^ ions [8]. The forth group consists of magnesium proton exchangers (MHX) proteins. The activity of a protein that exchanges protons with Mg^2+^, Zn^2+^ and Cd^2+^ ions was first identified in vacuolar vesicles of rubber tree (*Hevea brasiliensis*) [9,10]. Electrophysiological analysis and overexpression studies indicated that the *A. thaliana MHX* gene (*AtMHX*) encodes a Mg^2+^/H^+^ exchanger, which exchanges protons with Mg^2+^, Zn^2+^, Cd^2+^, and possibly Fe^2+^ ions across the vacuolar membrane [11,12]. As the vacuole is acidic compared to the cytosol, AtMHX apparently sequesters the metal cations into the vacuolar lumen, at the expense of releasing vacuolar protons into the cytosol. It is currently unknown whether the main impact of AtMHX on plant physiology is related to metal or proton (pH) homeostasis [12,13]. AtMHX is highly expressed in the vascular region, particularly the phloem, of tissues with photosynthetic potential [14]. The 5′ untranslated region (5′ UTR) of *AtMHX* includes an AUG codon upstream to the initiation codon of the main open reading frame (ORF). The resulting upstream ORF (uORF) significantly inhibits AtMHX expression, by inhibiting its translation [15] and subjecting its transcript to degradation by the nonsense mediated mRNA decay (NMD) pathway [16].

AtMHX showed high similarity (32% identity) to mammalian sodium calcium exchanger (NCX) transporters [11]. NCX proteins are included in the Ca^2+^/cation (CaCA) exchanger superfamily. This superfamily was defined as a group of transporters that carry cytosolic Ca^2+^ ions across membranes against their electrochemical gradient, by utilizing the electrochemical gradients of other cations, such as H^+^, Na^+^, or K^+^[17]. The CaCA superfamily was classified into five major families, which were named, according to their first characterized member, YRBG, CAX, CCX, NCX, and NCKX [17,18]. YRBG transporters were mainly found in bacteria [18]. CAX (CAtion eXchangers) are cation/H^+^ exchangers found in plants, bacteria, fungi, and lower vertebrates, but not in higher animals (reviewed in [19]). All plant CAX genes tested thus far transported Ca^2+^, Mn^2+^, Cd^2+^, and Zn^2+^ to varying degrees [20]. CCX (Ca^2+^/cation exchangers) characterized thus far catalyse both Na^+^/Ca^2+^ and Li^+^/Ca^2+^ exchange (reviewed in [18]). NCX are Na^+^/Ca^2+^ exchangers, and NCKX are K^+^-dependent Na^+^/Ca^2+^ exchangers. NCX and NCKX proteins were identified in mammals, nematodes, insects, squid, and algae [17,21,22].Vertebrates NCX proteins were clasified into four groups (named NCX1-4) [18,21,23].

**Figure 1:**
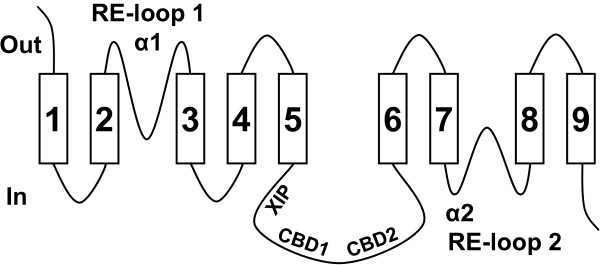
**A structural model of NCX1.** The schematic illustration is based on the experimental observations and resulting structural model of mammalian NCX1 transporters described in [33-35]. The rectangles and lines represent the TMSs and loops, respectively, of NCX1. The “in” and “out” positions correspond to the cytosol and extracellular space, respectively. See text for details about the RE-loop (reentrant loop), α1, α2, XIP, and CBD regions.

The cardiac sarcolemmal Na^+^/Ca^2+^ exchanger (NCX1) [24] is localized in the plasma membrane, and extrudes Ca^2+^ to the extracellular space by utilizing the gradient of Na^+^ ions. NCX1 is important for maintaining the balance of Ca^2+^ ions during cardiac excitation/contraction, and its structure and function were extensively studied (reviewed in [25-31]). The topology of NCX1 was investigated by mutating residues near the predicted TMSs to cysteines and then examining the effects of intracellular and extracellular sulfhydryl-modifying reagents. Based on this biochemical approach it was concluded that NCX1 includes a cleaved signal peptide, nine transmembrane segments (TMSs), and two reentrant loops [32-35] (Figure 1). It was suggested that the reentrant loops participate in the formation of the ion transport pathway of NCX1 [35]. The reentrant loops overlap two regions of internal similarity in NCX1, designated the α1 and α2 repeats, which apparently resulted from an ancient gene duplication event [36,37].

NCX1 includes a large intracellular loop between TMSs 5 and 6. This loop is not essential for Na^+^-Ca^2+^ exchange activity, but has a regulatory function [38]. NCX1 is activated by binding of intracellular Ca^2+^ ions to two high-affinity Ca^2+^-binding domains, called CBD1 and CBD2, which are located in this large loop [39] (Figure 1). NCX1 inactivation by intracellular Na^+^ ions is mediated by a basic 20-amino acid segment of this large loop, called the XIP (eXchanger Inhibitory Peptide) region [40,41] (Figure 1).

The crystal structure of a prokaryotic Na^+^/Ca^2+^ exchanger (NCX_Mj of the archaea *Methanococcus jannaschii*) was recently characterized [42]. While the biochemically-determined topological model of the mammalian NCX1 exchanger only possesses nine TMSs, the crystal structure of NCX_Mj contains ten TMSs.

With the recent increase in genomic information from various organisms, more knowledge is gained about the phylogenetics of plant metal transporters [22,43,44]. To increase our knowledge about the MHX group of Mg^2+^ transporters, we present here a phylogenetic and structural analysis of 31 MHX proteins originating from 26 plant species.

## Methods

### Sequencing the cDNA of the tomato, potato and wheat *MHXs*

We determined the cDNA sequence of the *MHX* genes of *Solanum lycopersicum* (tomato), *Solanum tuberosum* (potato), and *Triticum aestivum* (wheat). All sequences were determined on both strands. The specific clones used for sequencing were CTOA20E13, derived from *S. lycopersicum* cv. TA492, ST_BEa0006L05, derived from *S. tuberosum* cv. Bintje, and whoh15o16 (BJ273167), derived from *T. aestivum* cv. Chinese Spring. The CTOA20E13 clone of *S. lycopersicum* includes a foreign DNA insert of 112 bp, whose borders were determined by cloning the corresponding region of *S. lycopersicum* cv. VF-36.

### Database searching and editing

The RefSeq (NCBI) and Phytozome databases were searched using the BLASTP and TBLASTN commands. The JGI database was searched (using the TBLASTN command) for additional algal proteins (at http://genome.jgi.doe.gov/genome-projects/pages/projects.jsf?kingdom=Alga). Additional file 1 presents a table of all proteins analysed in this study, and Additional file 2 provides the sequences, accession numbers and explanations about manual modifications (see below). Proteins identified based on their similarity to AtMHX are detailed in section 1 of Additional file 1. Some sequences were partial, or apparently included extra N-terminal regions, probably due to misannotation of introns. These sequences were manually re-annotated based on similarity searches in the genomic sequences (Additional file 2). Most proteins were named here by their source organisms. The two MHX paralogs identified in each of the *Oryza sativa* (rice) subspecies, Japonica and Indica, were named O.sativa_J1 (J1 stands for Japonica 1), O.sativa_J2, O.sativa_I1 (I1 stands for Indica 1), and O.sativa_I2. Additional similarity searches were carried out using the proteins identified in *Chlamydomonas reinhardtii*, *Selaginella moellendorffii*, and *Physcomitrella patens* as queries. Only searches using the sequence from *C. reinhardtii* allowed us to identify new proteins, and this was followed by similarity searches using some of the new proteins as queries. The resulting 14 proteins are included in section 2 of Additional file 1. Nine proteins that were identified in lower organisms based on their similarity to *Homo sapiens* NCX1 (HsNCX1) are included in section 3 of Additional file 1. The proteins in sections 2 and 3 of Additional file 1 were named according to their source organisms, together with an arbitrary number when more than one protein was identified in the same organism (this number is, therefore, not related to the four groups of vertebrate NCXs).

The number of EST clones derived from each of the *M. guttatus* and *P. patens MHX* genes was determined using files including the EST data of these plants, which were sent to us ahead of publication by the Phytozome database.

### Phylogenetic analysis and drawing of the phylogenetic trees

The phylogenetic analyses included marker proteins for the CAX, CCX, NCKX and YRBG families of the CaCA superfamily (section 4 of Additional file 1). These markers were chosen from the proteins classified by Cai and Lytton [17], which performed the first phylogenetic analysis of the CaCA superfamily. The *A. thaliana* CAX1-5 proteins served as markers for the CAX family. The *A. thaliana* CAX7-9 and CAX11 proteins served as markers for the CCX family. Although the latter proteins were initially called CAX, they belong to the CCX family [17,19]. For clarity, the name of each marker protein included, besides the transporter name, the name of the family to which it belongs. For example, AtCAX7 was called AtCAX7_CCX. Markers for the NCX family (section 4.5 of Additional file 1) were chosen from the proteins classified by Marshall and co-workers [21]. The latter proteins were named by their source organisms, the name of their family (NCX), and a number that indicates to which of the four groups of vertebrate NCXs they belong, as indicated by [21]. Updated sequence information (as for April 2011) was retrieved from the databases for each of the marker proteins.

Phylogenetic analyses were conducted using *MEGA5*[45]. We utilized the Maximum Likelihood method based on the Jones-Taylor-Thornton (JTT) amino acid substitution model [46]. The bootstrap consensus trees inferred from 1000 replicates [47] were presented. Branches corresponding to partitions reproduced in less than 50% bootstrap replicates were collapsed. The phylogenetic trees were drawn to scale, with branch lengths measured in the number of substitutions per site. All positions containing gaps and missing data were eliminated.

### Topological analysis

Hydropathy analysis and prediction of the TMSs and their orientation were conducted using the TMpred algorithm [48]. The presence of a signal peptide was predicted by the SignalP 4.0 algorithm [49].

### Sequence alignments and determination of pairwise similarity scores

Protein sequences were aligned by ClustalW2 [50] or Multalin [51] as indicated in the text. The pairwise similarity scores presented in Additional file 3 were determined by ClustalW2.

### Western blot analysis of AtMHX under reducing and non-reducing conditions

Western blot analysis of AtMHX under reducing or non-reducing conditions (in the presence of β-mercaptoethanol or N-ethylmaleimide, respectively) was carried out according to [52] with some modifications. Tobacco (*Nicotiana tabaccum* cv. Samsun NN) plants overexpressing AtMHX [12] were grown in a climate-controlled greenhouse with a photoperiod of 16 h light and 8 h darkness. Leaves of five-week-old plants were harvested and crushed into a fine powder in liquid nitrogen. Frozen plant powder (25 mg) was added into pre-weighted tubes containing 150 μl of 125 mM Tris-HCL pH 6.8, 20% glycerol, 6% SDS, 0.006% bromophenol blue, protease inhibitors (1 mM PMSF, 0.5 μg/ml leupeptin, and 1 μg/ml aprotinin), and either 2.145 M β-mercaptoethanol or 25 mM N-ethylmaleimide (NEM). The samples were mixed well and incubated on ice for 2 h with frequent mixing. The samples were then centrifuged for 2 min at 4°C, 14,000 g, and aliquots that derived from 2.5 mg plant powder were fractionated by SDS-PAGE and blotted on a PVDF membrane (Bio-Rad). Western blot analysis was performed by the chemiluminescence method, using polyclonal antibodies against a peptide from AtMHX sequence (Cys-Glu-Glu-Ile-Asp-Thr-Ser-Lys-Asp-Asp-Asn-Asp-Asn-Asp-Val-His-Asp) that were affinity-purified prior to use against the same peptide using the SulfoLink Coupling Gel (Pierce).

## Results and discussion

### MHX proteins constitute a sixth family within the CaCA superfamily

Genes with similarity to *AtMHX* were searched in the databases or sequenced by us (Additional files 1 and 2) (see Methods). For the sake of clarity, most proteins were named here according to their source organisms. To determine the identity of the identified proteins, it was necessary to include in the phylogenetic analysis marker proteins for each of the five known families of the CaCA superfamily (section 4 of Additional file 1; see Methods). In particular, many marker proteins were used for the NCX family.

**Figure 2:**
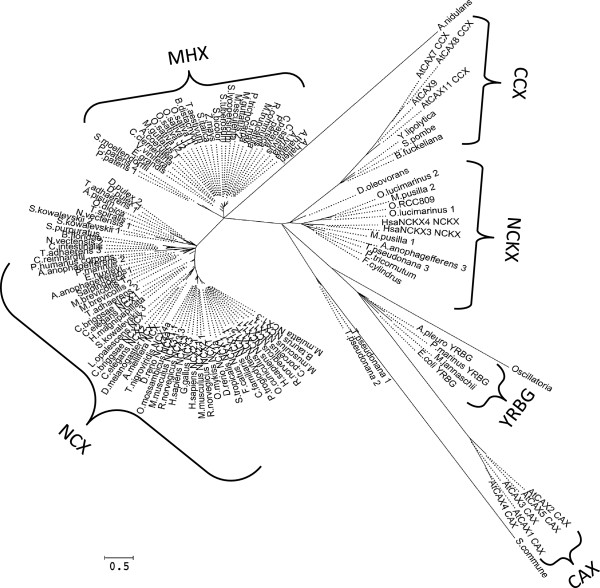
**An unrooted, maximum likelihood phylogenetic tree of all proteins.** The analysis included all the proteins detailed in Additional file 1. The tree was generated by *MEGA5*[45], after alignment of the sequences, as detailed in the Methods. The bootstrap consensus tree inferred from 1000 replicates is presented. Branch lengths are represented by the solid (but not the dashed) lines. The percentage of replicate trees in which the associated proteins cluster together in the bootstrap test are indicated in Additional file 4, which presents the results of the same phylogenetic analysis shown here in the form of a rooted tree.

Phylogenetic analysis of all the sequences showed that MHX and NCX proteins belong to two separate families of the CaCA superfamily (Figure 2 and Additional file 4). The appearance of the MHXs as a subgroup of the NCX family in a previous analysis [22] might result from the use of the hydrophobic regions only of the proteins analyzed, while the present analysis utilized the full protein sequences. The CaCA superfamily was originally indicated as containing five families [17]. Our results indicated that MHX proteins constitute a sixth family within this superfamily. The pairwise scores of each protein similarity to AtMHX, *Homo sapiens* NCX1 (HsNCX1), and the other proteins analysed are shown in Additional files 1 and 3. The phylogenetic identity of each protein, as determined based on its position in the phylogenetic tree and the scores of its similarity to the marker proteins, is presented in Additional file 1.

MHX proteins were found in three divisions of the plant kingdom. The 28 MHXs identified in the first division, Magnoliophyta (angiosperm) showed a similarly level of ~70% to each other (Additional files 1 and 3). In the second division, Lycopodiophyta, a protein showing 41% similarity to AtMHX was found in *S. moellendorffii*, a vascular non-seed plant. In the third division, Bryophyta, two proteins showing 39 and 40% similarity to AtMHX were found in the moss *P. patens*, a non-vascular, non-seed plant. Although the angiosperm and non-seed plant proteins appeared in two different clades (Figure 2), the non-seed plant proteins showed a higher similarity to angiosperm MHXs than to NCX proteins (scores of ~40 and ~30%, respectively; Additional file 3), and were, therefore, considered part of the MHX family. The existence of MHX family members in the moss *P. patens* indicates that although the vascular system is one of the major sites of AtMHX expression in *A. thaliana*[14], MHX proteins also exist in non-vascular plants.

### The MHXs apparently evolved from the NCXs after the split of the chlorophyte and streptophyte lineages

Database searches identified proteins with similarity to AtMHX in a fourth division of the plant kingdom, namely Chlorophyta (green algae). Within the Chlorophyta, six proteins showing similarity to AtMHX were identified in four single-celled algae - *Ostreococcus lucimarinus*, *Ostreococcus* sp. RCC809, *Micromonas pusilla*, and *C. reinhardtii* (section 1.1.4 of Additional file 1). The phylogenetic analysis showed that the protein identified in *C. reinhardtii* belongs to the NCX family, while the five proteins identified in the three other species belong to the NCKX family (Figure 2 and Additional file 1). NCX and NCKX proteins were previously identified in algae [22]. Among the chlorophytes whose genomic information is currently available, no proteins showing similarity to AtMHX were identified in *Volvox carteri* or *Chlorella* sp. NC64A. The currently available sequences of red algae do not include a protein with similarity to AtMHX. Proteins showing similarity to AtMHX were identified in some algae that do not belong to the Plantae but to the Chromalveolata kingdom (section 1.2 of Additional file 1). Among the nine proteins identified in Chromalveolata, three belong to the NCX family while four proteins belong to the NCKX family (Figure 2 and Additional file 1). Two additional proteins identified in *Thalassiosira pseudonana* apparently belong to a novel family of the CaCA superfamily (Figure 2).

Most of the proteins identified by their similarity to the *C. reinhardtii* NCX (CrNCX) (section 2 of Additional file 1), as well as the proteins identified in lower organisms by their similarity to HsNCX1 (section 3 of Additional file 1), were more related to NCX than to MHX proteins (Figure 2, and Additional files 1, 3, and 4). Searches in a large number of fungal species whose genome sequences were available revealed only five proteins showing limited similarity to AtMHX. The protein identified in *Schizosaccharomyces pombe* was classified to the CCX family [17]. The analysis showed that the proteins identified in *Botryotinia fuckeliana* and *Yarrowia lipolytica* were related to the CCX family as well (Figure 2). The proteins identified in the fungi *Aspergillus nidulans* and *Schizophyllum commune* could be the founder members of novel families of the CaCA superfamily (Figure 2). It, therefore, seems that there are no close homologs of MHX proteins in fungi. Yet, it is possible that Mg^2+^/H^+^ exchange is carried out in fungi by proteins with a different phylogenetic identity. It was reported that there is Mg^2+^/H^+^ exchange activity in *Saccharomyces cerevisiae*[53], but the involved protein has still not been identified.

Many prokaryotic proteins showed some degree of similarity to AtMHX or HsNCX1. However, preliminary phylogenetic analyses showed that even the prokaryotic proteins with the highest similarity to AtMHX or HsNCX1, which were identified in *Desulfococcus oleovorans* and the cyanobacterium *Oscillatoria*, respectively, did not belong to either the MHX or NCX families. We, therefore, did not include in the phylogenetic analysis other prokaryotic proteins except those of *D. oleovorans*, *Oscillatoria*, and *M. jannaschii*. The latter species was added because the crystal structure of its Na^+^/Ca^2+^ exchanger was recently characterized [42], and we wanted to evaluate the phylogenetic relatedness of this protein to MHX and NCX family proteins. As shown in Figure 2, the *M. jannaschii* Na^+^/Ca^2+^ exchanger belongs to the YRBG family, the *D. oleovorans* protein belongs to the NCKX family, and the *Oscillatoria* protein can belong to a novel family of the CaCA superfamily. It, therefore, seems that prokaryotes do not contain MHX-family proteins.

The analyses of all currently available sequences indicated that the MHX family is limited to plants and does not exist in other organisms. The plant kingdom includes two major phylogenetic groups that splitted ~1.2 billion years ago [54], namely the streptophytes (containing land plants and charophyte algae) and the chlorophytes (containing green algae, such as *C. reinhardtii* and *O. lucimarinus*). Genomic information is not yet available for charophyte algae. The currently available chlorophyte genomes do not include proteins with homology to the MHXs, but only to NCX or NCKX proteins. As indicated by their pairwise similarity scores (Additional files 1 and 3), the MHXs are more similar to the NCXs than to the NCKXs. The currently available data are consistent with the suggestion that the MHXs evolved from the NCXs after the split of the chlorophyte and streptophyte lineages of the plant kingdom.

### Evidence for functional diploidization of the *MHXs*

**Figure 3:**
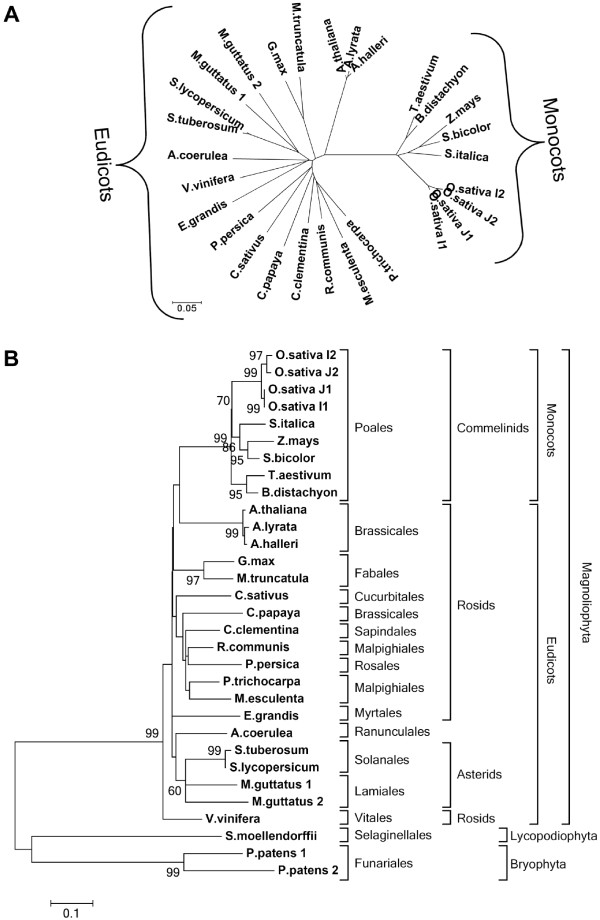
**Maximum likelihood phylogenetic trees of the MHXs.** The trees were generated by *MEGA5*[45], after alignment of the sequences, as detailed in the Methods. **A**. An unrooted bootstrap consensus tree of angiosperm MHXs inferred from 1000 replicates of the analysis. **B**. A rooted bootstrap consensus tree of plant MHXs. The percentage of replicate trees in which the associated proteins clustered together in the bootstrap test (1000 replicates) are shown next to the branches. Bootstrap values lower than 60% are not shown. The proteins were classified by the phylogenetic groups of their source organisms.

Angiosperm MHX proteins were analysed separately in order to view their phylogenetic relationships at a higher resolution (Figure 3). In general, the similarity between MHX orthologs of different plant species correlated with the phylogenetic relationships of the plants. For example, MHX proteins clearly diverged into monocot and eudicot clades (Figure 3A).

Most plants had only one MHX ortholog. Among the angiosperm whose genome sequences were available, two full *MHX* genes were identified only in *Mimulus guttatus* (an eudicot) and *O. sativa* (a monocot) (see below). Three of the plant species analysed here are polyploid: *T. aestivum* (a hexaploid), and *S. tuberosum* and *Glycine max* (which are both tetraploids). The *G. max* genome had been completely sequenced. Chromosome 2 of *G. max* includes a 1 kb fragment with high similarity to part of the full *G. max MHX* gene located on chromosome 10 (see Additional file 2). This 1 kb fragment cannot encode a full MHX protein. This indicates that the *MHX* loci were reduced to only two alleles subsequent to tetraploidization of the *G. max* genome. The secondary inactivation of some alleles subsequent to a previous polyploidization event, which results in the remaining of only two active alleles, was termed ‘functional diploidization’ (e.g., [55,56]). This process can occur, for example, by turning some of the genes into pseudogenes [57]. Thus, the *MHX* loci of *G. max* underwent functional diploidization. Only one cDNA clone encoding a full MHX protein could be identified in the currently available databases of *T. aestivum* and *S. tuberosum*, and all the ESTs found were related to the single cDNA identified in each of the two species. One possible explanation for the identification of only one *MHX* cDNA in each of these species is related to the fact that their genomes have not been completely sequenced. Yet, the absence of ESTs corresponding to another *MHX* gene in these polyploid species is in line with the possibility that their *MHX* genes underwent functional diploidization, which rendered extra gene copies non-functional.

*Zea mays* (maize) is a diploid but includes, in additional to a full *MHX* gene, two regions with similarity to parts of this gene (see Additional file 2). The existence of more than one copy of certain genes in diploid plants can be explained by the fact that there have been widespread genome duplication events throughout the history of flowering plants, including most species that are now considered to be diploid ([58] and references therein). A polyploidization event occurred about 70 million years ago (MYA) in the common ancestor of the major cereals [59]. Maize underwent another genome-wide duplication event approximately 11 MYA [60]. However, currently, only one functional *MHX* gene remains in the maize genome, indicating that the *MHXs* of this species underwent functional diploidization.

Although *O. sativa* is a diploid, it has two *MHX* genes. The most recent genome-wide duplication event in rice history predated rice divergence from sorghum [59]. This fact, together with the phylogenetic trees shown in Figure 3, indicate that the two *MHX* genes of rice originated from isolated gene duplication and not from a genome-wide duplication event. The two proteins identified in each of the *O. sativa* subspecies Japonica and Indica showed a high similarity (97%) to each other (Figure 3, and Additional file 3). These four proteins segregated into two mini-clades, each including one Japonica and one Indica ortholog (Figure 3B). This suggests that *MHX* gene duplication in *Oryza* occurred before the split between the *O. sativa* Indica and Japonica subspecies, which was estimated to have occurred 200,000–400,000 years ago [61,62]. It is interesting that the two Indica *MHX* paralogs are located relatively close to each other (about 57,000 bp apart) on chromosome 2, whereas in Japonica, one *MHX* paralog is located on chromosome 2 and the other on chromosome 11. This indicates that one *MHX* paralog had translocated in one of the two subspecies (most likely, following an initial duplication in chromosome 2, one Japonica *MHX* paralog translocated to chromosome 11). The two *O. Sativa* orthologous MHX proteins of the first mini-clade, O.sativa_J1 and O.sativa_I1 (of the Japonica and Indica subspecies, respectively), are highly similar to each other (Figure 3B). The two orthologs of the second mini-clade, O.sativa_J2 and O.sativa_I2, are somewhat less similar to each other (Figure 3B). Thus, the second mini-clade underwent a more rapid evolutionary divergence process compared to the first one. Moreover, for both Japonica and Indica, all the ESTs of *MHX* genes that were found in the RefSeq database (nine for Japonica and three for Indica) originated from the first, but not the second, mini-clade. This suggests that the second mini-clade is expressed at a much lower level than the first, or its expression is much more restricted at the spatial or temporal level. These data are also consistent with the possibility that the genes of the second mini-clade turned into pseudogenes and are, therefore, neither expressed nor subjected to natural selection that restricts their divergence process. In accord with this possibility, we were unable to identify in the genomic sequence of the Indica subspecies the coding region of the last ~20 amino acids of the O.sativa_I2 protein, despite the high similarity in this region between the MHXs and, in particular, the three other *O. sativa* MHX sequences (Additional files 2 and 5). O.sativa_I2 was, therefore, omitted from all sequence alignments.

The presence of two *MHX* genes in *M. guttatus*, which was sequenced from the diploid inbred line IM62, can be explained by the fact that members of the genus *Mimulus* underwent polyploidization events in their history [63]. As far as we are aware, no data were published about the exact time of the polyploidization event in *M. guttatus* history, but the observation of only 74% similarity between the two MHXs of *M. guttatus* (Figure 3 and Additional file 3) suggests that considerable time had passed since this event. One EST could currently be identified in the *M. guttatus* EST database for each of its two *MHX* genes. This makes *M. guttatus* the only plant species in which evidence for the expression of more than one *MHX*-paralogous gene was obtained thus far.

The moss *P. patens* includes two *MHX* paralogous genes, whose deduced protein sequences share 65% similarity with each other (Additional file 3). Similar to all mosses, the enduring *P. patens* plant represents the haploid gametophyte. However, there is evidence that similar to most seed plants, *P. patens* is a paleopolyploid, which underwent a genome duplication event between 30-60 MYA [64]. The phylogenetic analysis indicates that *MHX* gene duplication occurred in mosses (or some of them) after the divergence of vascular plants from the mosses, which occurred approximately 460 MYA ([64] and references therein). It is, therefore, likely that *MHX* gene duplication resulted from the indicated polyploidization event in *P. patens* history. One EST was found in the database for the gene encoding P.patens_1, but no EST was found for P.patens_2. In addition, we were unable to undoubtedly identify the sequence encoding the first ~16 amino acids of P.patens_2 in the *P. patens* genomic data, although this may result from the low similarity between the MHXs in this region. More data will be necessary to clearly determine if P.patens_2 is expressed, but based on the current data it is possible that *MHX* underwent functional diploidization in (the diploid generation of) *P. patens*.

To conclude, the current EST and genomic data suggest that the *MHXs* underwent functional diploidization in most plant species.

### Most plant *MHX* genes include uORFs

The 5′ UTR of *AtMHX* includes an uORF that substantially inhibits the expression of this gene by lowering its transcript content through NMD [16] and by inhibiting its translation [15]. It was interesting to learn whether the *MHX* genes of other plants include uORFs as well. Information about the 5′ UTR was available for only some of the *MHX* genes. It was found that whereas only about 20% of the genes in plants include an uORF [65], uORFs are present in a high percentage of the *MHX* genes (Additional file 6). Among the ten eudicot *MHX* genes whose 5′ UTR sequences were available, all genes (100%) included at least one uORF. Three (50%) of the six available 5′ UTR sequences of monocot *MHX* genes included an uORF. This suggests that the presence of an uORF in *MHX* genes has some evolutionary advantage, possibly for restricting the expression of these genes.

In eukaryotes, the likelihood that an upstream AUG (uAUG) codon will be recognized by the ribosome and, hence, can inhibit expression, depends on the strength of its sequence (Kozak) context [66,67]. The uAUGs of plant *MHX* genes have not only weak but also sub-optimal and strong contexts (Additional file 6A). Moreover, we showed that the uAUG codon of *AtMHX* is well recognized despite its weak context due to its stable secondary structure, resulting in strong inhibition of this gene expression [15,16,68]. Recognition of uAUG codons may lead to inhibition of translation and/or NMD [15,16]. The likelihood that either of the latter impacts will be realized is increased when the length of the uORF peptide is increased [69,70]. The potential uORF peptides of the currently identified *MHX* 5′ UTRs are presented in Additional file 6B. However, it is not possible to specify a definite cutoff size for peptide length that will inhibit translation [69], and even the short uORF of *AtMHX* inhibited translation and elicited NMD [15,16]. It will, therefore, be necessary to experimentally determine whether the expression of other plant *MHX* genes is inhibited by their uORFs. It will also be necessary to determine if uORFs, which are much more abundant in the *MHXs* than in most plant genes, play a role in functional diploidization of some *MHXs*. For example, there are two *MHXs* in *O. sativa*, but the 5′ UTR sequence is available for only O.sativa_J1. This 5′ UTR does not include any uAUG. Our data suggest that O.sativa_J1 is expressed either exclusively or to a much higher level than O.sativa_J2 (see above). In *M. guttatus*, the gene encoding the M.guttatus_1 protein has a particularly high number of uAUGs (11), including one with a strong Kozak context. The 5′ UTR of the gene encoding M.guttatus_2 is currently unknown.

The peptides encoded by the uORFs of plant *MHXs* are not evolutionary conserved (Additional file 6B). This suggests that the amino acid sequence of the uORF peptide does not have a functional importance not only for *AtMHX*[15,16] but also for the expression of other *MHX* genes. The similarity between the uORF peptides of the *MHXs* of *A. thaliana* and *A. halleri*, or *S. lycopersicum* and *S. tuberosum* (Additional file 6B) apparently resulted from the evolutionary relatedness of each pair of species.

### Conserved motifs in MHX and NCX transporters

We attempted to identify sequence elements that are conserved in the MHX and NCX families, as well as those that distinguish them from each other. We also attempted to identify sequence elements that characterize the MHX proteins of specific plant groups. This analysis can provide the basis for future experiments designed to determine the significance of the identified elements for the function and ion selectivity of these transporters. Alignment of all currently identified MHX proteins is shown in Additional file 5. Some of the proteins lack or apparently have few extra residues in the N-terminus. This is due to the low similarity between the MHXs in this region, which made it difficult to accurately predict the initiation point in the genomic sequence (see Additional file 2). The longest motif conserved in all MHX proteins, which includes six amino acids, is TADSAI at position 430 of AtMHX. There are also two conserved five-residue motifs – ASKIA and ELGGP – at positions 420 and 505 of AtMHX, respectively. As shown in Additional file 7, NCX proteins have, at the corresponding locations, similar motifs that are, however, not completely conserved in all NCXs. When only angiosperm MHX proteins were aligned, much longer conserved motifs could be identified (Additional file 8).

**Figure 4:**
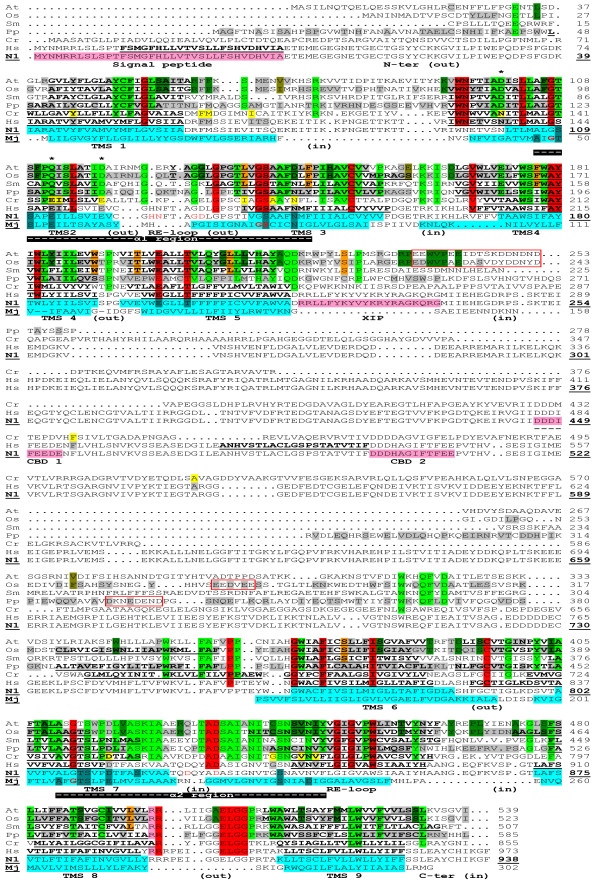
**Alignment of representative MHX proteins with the structural models of NCX1 and NCX**_**Mj.** The figure shows the alignment of the MHXs of *A. thaliana* (At), *O. sativa* (Os; O.sativa_J1 is presented), *P. patens* (Pp; P.patens_1 is presented), and *S. moellendorffii* (Sm), as well as CrNCX (Cr), HsNCX1 (the Hs and N1 sequences), and NCX_Mj (Mj). To create the alignment, all MHX and NCX proteins included in Additional file 1 were first aligned by ClustalW2. Then, all proteins except those presented in Figure 4 were omitted, without changing the relative positions of the remaining proteins. This was followed by slight manual modifications and removal of gaps that appeared in all the presented proteins. For the At, Os, Pp, and Hs sequences, only residues that were totally conserved in the whole group they represented were highlighted, either in gray or as follows: red - residues conserved in all the MHXs and NCXs, including CrNCX (the four residues that are also conserved in NCX_Mj are indicated by bold, red letters in the Mj sequence); pink – a residue that was conserved in all the MHXs and NCXs except CrNCX; light green - residues conserved in all MHXs; orange - residues conserved in all the MHXs of vascular plants that differ from the corresponding residues of both *P. patens* paralogs; dark green - residues conserved in all the MHXs of angiosperm that differ from the corresponding residues of non-seed plants; olive-green – residues that differ between the MHXs of all monocots and all eudicots. CrNCX residues that matched the conserved sequence of all MHX or NCX proteins were highlighted in light green or yellow, respectively. The asterisks indicate residues that differ between the consensuses of all MHXs and all NCXs analysed here. Bold, underlined letters indicate regions predicted to be TMSs by the TMpred algorithm. Below the first HsNCX1 sequence (Hs), there is a duplicate sequence of this protein (N1), which presents the current structural information about NCX1. The sequence of NCX_Mj (Mj) was used to present the current structural information about this protein, and was aligned with HsNCX1 as indicated in [83] with slight modifications. The TMSs of NCX_Mj (Mj) were highlighted in light blue and the residues forming the ion binding sites, as indicated in [83], were highlighted in cyan. All the other structural information shown in this figure resulted from the study of mammalian NCX1 proteins. The experimentally determined location of NCX1 TMSs [21,33-35] were highlighted in light blue on the N1 sequence. The position and orientation of NCX1 loops and reentrant (RE) loops were indicated below the sequences. The α1 and α2 repeat regions of NCX1, as defined in [36], were indicated by white dashes highlighted in black below the sequences. The signal peptide, XIP and CBD regions of NCX1 were indicated by pink highlighted letters in the N1 sequence. Residues highlighted in cyan or written in red letters in the N1 sequence indicate amino acids whose mutagenesis resulted in a complete loss of NCX1 activity [25,36] or significantly altered its Ca^2+^ affinity [35], respectively. The residue numbers of the N1 sequence (indicated by bold, underlined numbers) refer to the mature protein. Regions in the large central loops of the MHXs that have a relatively high density of negatively-charged residues were indicated by red boxes.

To facilitate the presentation of sequence elements that characterize each group of proteins, the sequences of several representative proteins were aligned (Figure 4). The MHX proteins shown were from *A. thaliana* (representing the MHXs of eudicots), *O. sativa* (for the monocots), *P. patens* (for the two sequences currently available from the Bryophyta), and *S. moellendorffii* (the only MHX protein currently available from the Lycopodiophyta). HsNCX1 represented the NCX group. CrNCX was shown separately and was not taken into account in determining the consensus of NCX proteins due to its plant origin and in order to facilitate the visualization of its similarity to MHX and NCX proteins. The sequences labeled N1 and Mj were used to present the current structural information about NCX1 and the *M. jannaschii* Na^+^/Ca^2+^ exchanger, respectively (see below; the latter protein is called below NCX_Mj, according to its name in [42]).

For each protein (except SmMHX and CrNCX), only residues that were totally conserved in the whole group it represented were highlighted, either in gray or as detailed below. Residues conserved among all MHX and NCX proteins investigated here were highlighted in red. The motif ELGG (at position 505 of AtMHX) is the longest common sequence element of the two protein families, and it will be necessary to determine its functional significance. Interestingly, ten of the 32 residues conserved between MHX and NCX proteins are glycines. It was suggested that glycines provide flexibility to active enzyme sites [71]. Many conserved glycines were also identified in CAX proteins [19]. NCX_Mj, which similar to NCX1 functions as a Na^+^/Ca^2+^ exchanger, shares only four of the 32 residues that are completely conserved in all MHX and NCX proteins (these four residues are indicated by bold, red letters in the Mj sequence in Figure 4). Thus, there might be some structural or functional properties that are shared by MHX transporters and Na^+^/Ca^2+^ exchangers of the NCX family, but not by other Na^+^/Ca^2+^ exchangers such as NCX_Mj.

Negatively charged residues are particularly important for the function of cation transporters. The negatively charged residues conserved between the MHXs and NCXs are Glu^200^-Glu^199^, Asp^432^-Asp^829^, and Glu^505^-Glu^901^ of AtMHX and HsNCX1, respectively (Figure 4) (note that all HsNCX1 residue numbers refer to those of the mature protein, which are indicated on the N1 sequences, for coherence with NCX1 literature). However, since MHX and NCX proteins carry different ions, these residues are unlikely to determine the ion specificity. The two-proline element at positions 361-362 and 758-759 of AtMHX and HsNCX1, respectively, might be structurally significant [all MHX and NCX proteins have two prolines at that position, except the *Ciona intestinalis* NCX that has one proline (Additional file 9)]. Serine, threonine and cysteine residues shared by the MHXs, NCXs and NCX_Mj will be discussed afterwards.

### Sequence elements that differ between MHX and NCX proteins

Sequence elements that distinguish MHX from NCX transporters can provide a clue to the basis of the difference in their biochemical activities and ion specificities. To illustrate these elements, residues (except those highlighted in red) that were totally conserved in all MHX proteins identified thus far were highlighted in light green (Figure 4). There are only three residues (asterisked in Figure 4) in which all MHX proteins differ from all NCX proteins analysed. These three residues, which are located inside or near the functionally important α1 region, include: (i) Asp at position 100 of AtMHX - a negatively charged residue present in all MHXs, whereas all NCXs include the non-charged amino acid asparagine at the corresponding position. This asparagine is essential for NCX1 function, since its mutation into cysteine rendered the exchanger insensitive to regulation by cytoplasmic Na^+^ or Ca^2+^ ions [72]. (ii) Gln at position 112 of AtMHX is a polar, non-charged amino acid found in all MHXs, while all NCXs have the negatively charged glutamate residue (Glu^113^ of NCX1) at the same position. The different properties of Gln^112^ of AtMHX and Glu^113^ of NCX1 have been previously noted [17]. It is also interesting to note that the mutation of Glu^113^ of NCX1 into Gln resulted in a complete loss of NCX1 activity [25]. This substitution is identical to the natural variation between NCX and MHX proteins at this position. It will be interesting to examine whether this mutation, despite abolishing Na^+^/Ca^2+^ exchange activity, allowed NCX1 to carry other ions. (iii) The third residue that differs between all MHX and NCX proteins is Asp at position 119 of AtMHX, which parallels a glutamate residue of the NCXs (Glu^120^ of HsNCX1). Although both aspartate and glutamate are negatively charged, this variation can be functionally significant. For example, two NCX1 variants that had a Glu-to-Asp mutation at either position 113 or 199, were completely inactive [25]. However, mutagenesis of Glu^120^ into glutamine resulted in only 36% reduction in NCX1 activity [25]. Among the three residues that differ between all MHXs and NCXs, NCX_Mj shares the same residue with the NCXs only in the position that corresponds to Gln^112^ of AtMHX. Residues in this position are, therefore, particularly interesting candidates for participation in the determination of the different ion selectivities of Mg^2+^/H^+^ and Na^+^/Ca^2+^ exchangers.

It is likely that in addition to the three residues that differ between all MHX and NCX proteins, other residues can contribute to the different ion specificities of the two types of transporters. Particularly interesting candidates are conserved MHX amino acids whose properties differ from those of the corresponding residues of most NCXs. Such residues include (as presented in Figure 4 and Additional file 7), Leu^104^ (the positions refer to AtMHX) – most NCXs have methionine at this position; Ser^114^ – most NCXs have leucine at this position; Glu^173^ and Leu^418^ – most NCXs have threonine at these positions; Gln^207^ – most NCXs have phenylalanine at this position; His^214^ – most NCXs do not have a positively charged residue at this position; Pro^228^ – most NCXs do not have proline, a structurally important amino acid, at the vicinity of this position; and Ala^513^ – most NCXs have a positively charged residue at this position.

According to the discussion about MHX evolution, CrNCX as well as the MHXs apparently evolved from an NCX protein that was present in the common ancestor of the streptophytes and chlorophytes. To visualize the similarity of CrNCX to the NCXs and the MHXs, residues of CrNCX that matched the conserved amino acids of either NCX or MHX proteins were highlighted in yellow or light green, respectively (Figure 4). CrNCX sequence is similar to that of the NCXs in almost all the conserved sites of the latter proteins, but the same doesn’t hold true for the MHXs. The few conserved NCX residues that do not match their corresponding CrNCX residues can provide the basis for a putative difference between the activities of CrNCX and NCX proteins (if any). The consensus sequence of the MHXs, NCXs and CrNCX (Additional file 9) apparently represents parts of the ancestral NCX protein that was present in the last common plant-animal ancestor, which existed ~1.6 billion years ago [73].

### Sequence elements that characterize the MHXs of different plant groups

We also attempted to identify sequence elements that characterize the MHXs of different plant groups – vascular versus non-vascular plants, angiosperm compared to non-seed plants, and monocots versus eudicots. The differences between the MHXs of vascular and non-vascular plants could be functionally significant, considering the fact that AtMHX is highly expressed in the vascular system. It is, therefore, possible that MHX proteins had to adapt for their role in this system during the evolution of vascular from non-vascular plants. Residues that are conserved in the currently identified MHXs of all vascular plants, but differ from the corresponding amino acids of both *P. patens* MHXs (alignment not shown), were highlighted in orange in Figure 4. Among these residues, there is a relatively large difference between the properties of corresponding amino acids for Trp^191^ (the positions refer to AtMHX) (Ser in *P. patens*), Thr^197^ (Glu in *P. patens*), and Ser^225^ (Cys in *P. patens*). However, the current analysis is based only on the two sequences currently available from non-vascular plants, and it will be necessary to expand it in the future.

Residues that are conserved in angiosperm MHXs but differ from the corresponding residues of all the currently identified MHXs of non-seed plants (the MHXs of *S. moellendorffii* and *P. patens*), are highlighted in dark green in Figure 4. Six of these residues are localized between amino acids 234 and 242 of AtMHX, and it will be necessary to investigate whether this region is related to some functional properties that distinguish the MHXs of angiosperm from those of non-seed plants. In addition, there is a relatively large difference between the properties of corresponding amino acids for Asp^144^ and Asp^391^ (negatively charged residues) of angiosperm MHXs versus the corresponding two asparagines (polar uncharged residues) of non-seed plants; His^149^ (a positively charged amino acid) of angiosperm versus leucine (a non-polar amino acid) of non-seed plants; and Gly^209^ of angiosperm versus proline of non-seed plants. Finally, residues that are conserved in either monocot or eudicot MHXs but differ between the two groups, are highlighted in olive-green in Figure 4. There are only three such residues and, among them, Glu^160^ of the eudicots (serine in monocots) should be particularly noted. It will be important to examine whether the variations described here between the MHXs of various plant groups are related to differences, if any, between the properties of these MHXs.

### A structural model of the MHXs based on the resolved structures of NCX1 and NCX_Mj

**Figure 5:**
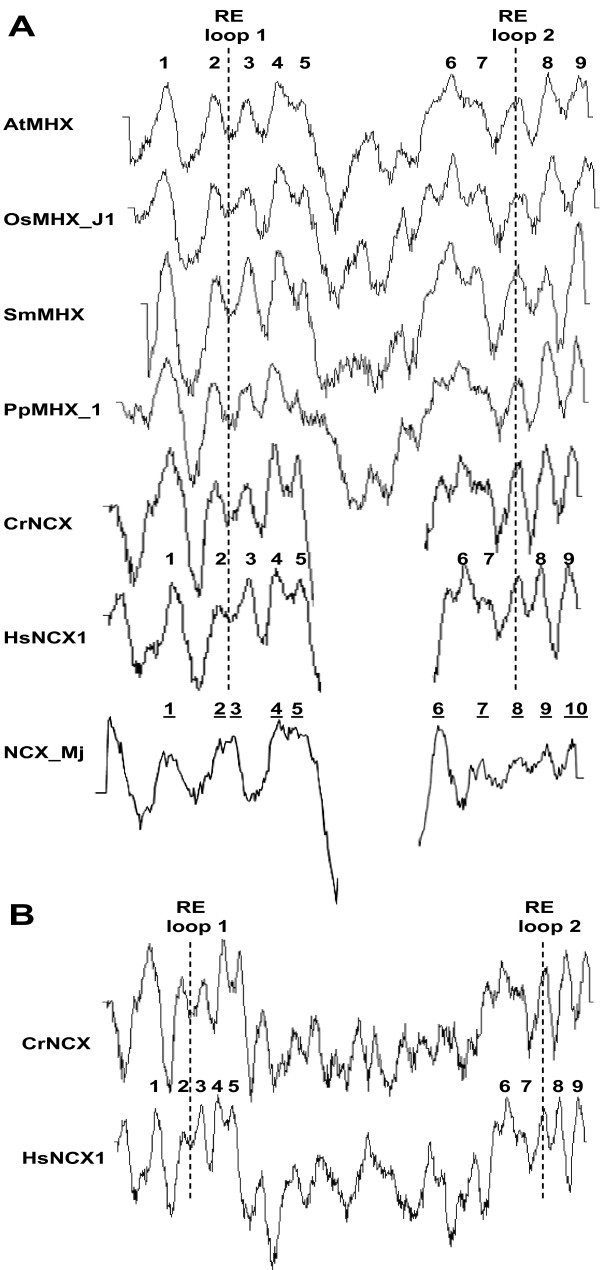
**Hydropathy plots of representative proteins. A**. The hydropathy plots of the proteins presented in Figure 4 were created by the TMpred algorithm [48] and aligned manually. The numbers indicate the positions of the experimentally-determined TMSs of NCX1, except the underlined numbers that refer to the TMSs of NCX_Mj. The dashed lines indicate the positions of the reentrant loops of NCX1. Most of the large central loop of HsNCX1 and CrNCX is not shown, while the full hydropathy plot of NCX_Mj is divided to two parts that are shown in different scales to achieve the best alignment with the other proteins. **B**. Hydropathy plots of the full HsNCX1 and CrNCX proteins.

The structure of NCX1 and NCX_Mj was extensively studied (see Background). We attempted to utilize this knowledge for gaining insight into the putative structure of MHX proteins. This could provide the basis for experimental investigation of MHX structure. Figure 5 shows the hydropathy plots of the representative proteins included in Figure 4. The hydropathy plots of MHX transporters from various phylogenetic groups resemble each other, and also show high similarity to the hydropathy plot of HsNCX1 and (perhaps to a somewhat lower extent) NCX_Mj (Figure 5A). This supports the idea that the structures of MHX transporters resemble those of the latter proteins. The duplicate sequence of HsNCX1 (labeled N1) and the sequence of NCX_Mj (labeled Mj) in Figure 4 present the current structural information gained from biochemical analyses of NCX1 and crystallography of NCX_Mj, respectively (see Background; note that the terms HsNCX1 and NCX1 refer to human NCX1 specifically and to NCX1 proteins in general, respectively). The experimentally identified TMSs of NCX1 and NCX_Mj are highlighted in light blue on the N1 and Mj sequences, respectively (Figure 4). Although NCX_Mj belongs to the YRBG family, it shares similarity with the NCXs, and was shown to function as a Na^+^/Ca^2+^ exchanger [42]. Yet, whereas NCX1 was shown to possesses nine TMSs and two reentrant loops [32-35], the crystal structure of NCX_Mj contains ten TMSs [42]. It was noted that there could be some deviation between the structures of NCX1 and NCX_Mj [74]. This possibility is sensible since despite its name (which is based on its function), NCX_Mj does not belong to the NCX but to the YRBG family. Biochemical methods correctly identified that YRBG proteins contain ten TMSs [75]. It is, therefore, reasonable that the biochemical analyses of NCX1 were able to correctly identify its nine TMSs and two reentrant loops [32-35]. Our suggested explanation for the basis of the structural difference between NCX1 and NCX_Mj is delineated below. In general, there was a good correlation between TMS position in NCX1 and NCX_Mj (Figure 4). However, the region that corresponds to the 8^th^ TMS of NCX_Mj was shown to be the second reentrant loop of NCX1 [33-35,74] (Figure 4). There is a conserved GXG motif at positions 449-451 of AtMHX and 846-848 of HsNCX1. It was noted [21,35] that this motif in NCX1 is similar to the GYG motif in the P-loop of K^+^ channels [76] and the GIG motif in the pore region of the sarcoplasmic Ca^2+^ release channels (RyR) [77,78]. It was suggested that this GXG motif creates a tight turn in the second reentrant loop of NCX1 [21,35]. Interestingly, this GXG motif is absent from NCX_Mj (Figure 4), possibly providing a structural basis for the existence in this location of a reentrant loop in the NCXs as compared to a TMS in NCX_Mj. Similar to the NCXs, the MHXs include this GXG motif. For this reason, in addition to the higher similarity of the MHXs to NCX1 than to NCX_Mj (pairwise similarity scores of ~30 and 15%, respectively; see Additional files 1 and 3) we assume that MHX structure is more similar to that of NCX1 than to the YRBG-family NCX_Mj protein. Therefore, the number and orientation of MHX TMSs and loops were indicated according to those of NCX1. However, except the difference detailed above, the crystal structure of NCX_Mj agrees with the structural analysis of NCX1, thereby strengthening the overall structural model presented in Figure 4.

In most regions, there was a good agreement between the experimental observations in NCX1 and the prediction of its TMSs by the TMpred algorithm (indicated by bold, underlined letters in the Hs sequence). The TMSs predicted by TMpred in the MHXs are indicated by bold, underlined letters in the MHXs sequences. It is reasonable that the positions of MHX TMSs correspond to those of NCX1, particularly where the TMpred predictions for the two groups of proteins agree with each other and with the experimentally-identified positions of NCX1 TMSs. As mentioned, NCX1 was shown to contain two reentrant loops [33-35]. The hydropathy plots of the MHXs resemble that of HsNCX1 also in the regions that correspond to the two reentrant loops of NCX1 (these regions are indicated by dashed lines in Figure 5). This analysis suggests that the MHXs have nine TMSs and two reentrant loops. However, it will be necessary to test this suggestion experimentally.

The TMSs predicted by TMpred were mainly located at equivalent positions of the four representative MHX proteins shown in Figure 4. However, there were certain regions of dissimilarity, for example, an extra TMS was predicted before TMS6 in the MHXs of *O. sativa* and *P. patens*, but not *A. thaliana* or *S. moellendorffii*. It was interesting to examine if there are subgroups of MHX proteins with distinct structures, for example, whether the MHXs of all monocots show structural similarity to O.sativa_J1. Additional file 10 shows the TMSs predicted by TMpred for all MHXs studied here. These TMSs were predominantly located at equivalent positions for most MHXs. This suggests that there is a relatively large similarity in the structures of various MHX proteins. For only few proteins, TMpred predicted a deviation from the common structure in the region of TMSs 5 and 6, which surround the large central loop. It was predicted that the *P. patens* MHXs lack TMS5, and that seven out of the 31 MHXs analysed have an extra TMS before TMS6. However, TMpred predictions for the large central loop region were not accurate also for NCX1, which was also predicted to have an extra TMS before TMS6 (Figure 4), while experimental evidence proved that this is not the case. The TMpred analysis was useful for identifying that there was no clear distinction between the MHXs of different phylogenetic groups, e.g., of monocots and eudicots (Additional file 10). Based on this fact, together with the high sequence similarity between different MHX proteins (Additional file 3), we assume it is not likely that some of them have a switched TMS topology, and they are all likely to share the common structure indicated in Figure 4. The *E. grandis* MHX was predicted to have an extra TMS before TMS1. The SignalP 4.0 algorithm suggests that the *E. grandis* MHX does not have a signal peptide, and it will be necessary to determine if the initiation point of this protein was correctly annotated.

While NCX1 has a cleaved signal peptide [32] (highlighted in pink in Figure 4), the corresponding region is missing in the MHXs. Except NCX1, none of the other proteins presented in Figure 4, including CrNCX, was predicted to have a signal peptide by the SignalP 4.0 algorithm [49]. The same algorithm correctly predicted the presence of a cleaved signal peptide in HsNCX1. The experimentally determined orientations of NCX1 loops, as well as N- and C-terminal regions [33-35], are indicated in Figure 4. For NCX1, which is located in the plasma membrane, the “in” or “out” oriented loops face the cytosol or the extracellular space, respectively. However, MHX proteins studied thus far are localized in the vacuolar membrane [11,79]. For tonoplast proteins, cytosolic loops are considered to be internal, whereas loops that face the vacuolar lumen are considered to be external. This categorization is based on the similar biophysical properties of the vacuolar lumen and the extracellular space in terms of their pH and electrochemical potential. The electrochemical potential plays an important role in determining the orientation of membrane-protein loops [80-82]. It will be necessary to determine if the orientation of MHX loops is similar to that of the corresponding NCX1 loops. The large central loop, which exists in both NCX and MHX proteins and faces the cytosol in NCX1, is much longer in NCX compared to MHX proteins (Figure 4). In accord with the segregation of the protein identified in *C. reinhardtii* with the NCX family, the length of its central loop is similar to that of HsNCX1 (Figures 4 and 5B).

The degree of sequence conservation was much higher in the predicted TMSs than in non-TMS regions of the various MHXs (34% and 16%, respectively) (Table 1). A similar phenomenon was observed in other membrane proteins. Exceptionally, loop f (the last loop before the C-terminus), which includes the conserved motif ELGGP, shows a high degree of conservation (50%) (Table 1). The large central loop (loop d) of the various MHXs showed a low degree of conservation. As mentioned above, the corresponding loop of NCX1 is not essential for its transport activity. Particularly notable is the high degree of conservation in TMSs 2, 3, 4, and 7, and in the second reentrant loop (Table 1). These areas (except TMS4) largely overlap the α-repeat regions, which have great importance for the transport activity of NCX1 and NCX_Mj [36,42]. The high degree of conservation of these regions in MHX proteins suggests that they play a critical role in the MHXs as well.

**Table 1:** The degree of sequence conservation in the predicted TMSs and loops of MHX proteins

**TMSs**			**Non**-**TMS regions**		
**TMS number**	**Identical/total aa**	**% Identity**^**a**^	**Region**	**Identical/total aa**	**% Identity**^**a**^
			N-ter^b^	1/37	3
1	5/20	25	Loop a	1/36	3
2	13/26	50	RE-loop 1	2/9	22
3	11/27	41	Loop b	3/12	25
4	11/25	44	Loop c	1/4	25
5	7/21	33	Loop d^b^	12/149	8
6	4/19	21	Loop e	0/5	0
7	7/20	35	RE-loop 2	27/67	40
8	4/19	21	Loop f	7/14	50
9	6/22	27	C-ter	0/6	0
**All TMSs**	**68**/**199**	**34**	**All non**-**TMS regions**	**54**/**339**	**16**

^a^The percentage of the completely conserved amino acids from the total number of amino acids in each TMS or loop of MHX proteins. The latter regions were defined according to the indications on the consensus sequence in Additional file 10.

^b^The total lengths assigned for the N-terminal and loop d regions were the averages of their lengths in the individual proteins (N-terminuses that lacked the initiation codon, as well as the N-terminus of E.grandis, were not taken into consideration in determining the average length).

**Figure 6:**
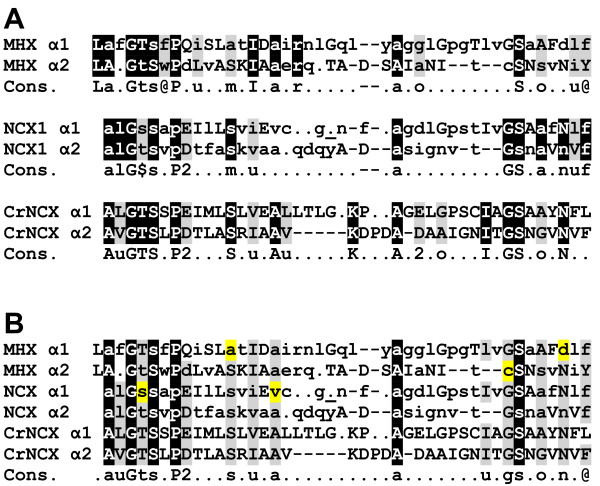
**Comparison of the α1 and α2 regions of the MHXs, NCXs and CrNCX. A**. The α1 and α2 regions of NCX proteins (as defined in [36]) were aligned. The comparable regions of MHX proteins and CrNCX were also aligned. All alignments were done manually. The sequences shown for the α1 and α2 regions of MHX and NCX are the consensus of each protein family in these regions. Uppercase letters indicate totally conserved residues, lowercase letters indicate the most common residue at each position, dots indicate residues for which there was no clear consensus, and dashes indicate gaps introduced to facilitate the alignments. The bold, underlined dot in the α1 of NCX1 indicates where three other dots were removed to simplify the alignment. White letters highlighted in black indicate residues conserved in the corresponding α1 and α2 positions of most or all proteins of each family. Letters highlighted in light gray represent residues with a similar nature in the corresponding α1 and α2 positions. The lower lines (Cons.) indicate the consensus sequence of the α1 and α2 regions of each protein family. Residue types were defined as: @ - aromatic (F, W, Y, or H); u - aliphatic (I, L, or V); 1 - basic (H, K, or R); 2 - acidic (D or E); p - charged (acidic or basic); $ - hydroxylic (S or T); m - methyl (A, S, or C,); o - small (G, A, S, or C). **B**. All the above indicated α1 and α2 regions were aligned together manually. White letters highlighted in black indicate residues that are conserved in the corresponding α1 and α2 positions of most or all MHX and NCX proteins. Letters highlighted in light gray represent positions in which there is a somewhat lower conservation. The lower line (Cons.) indicates the consensus sequence of the α1 and α2 regions of all the investigated MHXs and NCXs, as well as CrNCX. See text for explanation of the yellow highlighted letters.

The α1 and α2 repeat regions of NCX1, as defined in [36], are indicated in Figure 4. As mentioned, the α1 and α2 regions of NCX1 have internal similarity, and apparently originated from an ancient gene duplication event [36,37]. The α1 regions of the NCXs, MHXs and CrNCX were aligned to the α2 regions of the same proteins, either for each protein group separately (Figure 6A) or in combination (Figure 6B). The consensus sequence of the combined alignment (lower line in Figure 6B) apparently represents part of the amino acid sequence of the ancestral parental protein that existed before the duplication event. It is also possible to identify residues (highlighted in yellow in Figure 6B) that have apparently diverged from this presumed ancestral sequence in most MHX or NCX proteins.

Three of the ten glycines conserved in MHX and NCX proteins are located in the α1-reentrant loop 1 region and three are in the α2-reentrant loop 2 region (Figure 4). Of the three glycines conserved in the second reentrant loop, two create the GXG motif discussed above. The central amino acid of this motif is mainly Ile or Leu. The only three proteins (among the MHXs and NCXs analysed here) in which the central amino acid of this GXG motif is not Ile or Leu, are the MHXs of *S. moellendorffii* and *P. patens* (2^nd^ paralog), where the Xs are Fhe and Thr, respectively, and the NCX of *A. pisum*, where the X is Val. While the Ile, Leu, and Val residues have a similar nature, it will be interesting to learn if the benzyl or alcohol groups of Fhe or Thr, respectively, confer any special properties to the indicated MHXs of *S. moellendorffii* or *P. patens*, respectively.

### A search for MHX elements that correspond to functionally important NCX1 and NCX_Mj elements

The study of NCX1 resulted in identification of several sequence elements that play a critical role in the function of this transporter. Amino acids whose mutation resulted in a complete loss of NCX1 activity [25,36] are highlighted in cyan on the N1 sequence in Figure 4. These residues were mainly localized in the α repeat regions. Many of these residues corresponded to NCX_Mj amino acids that were shown to participate in ion binding (highlighted in cyan on the Mj sequence), which were all from the two α repeats [83]. The nine residues whose functional significance was indicated in both NCX1 and NCX_Mj (that are highlighted in cyan in both the N1 and Mj sequences) include two charged residues, six Ser/Thr residues, and Asn at position 143 (all numbers correspond to the N1 sequence). The charged residues are Glu^113^ (as discussed above, this residue is Glu in all NCXs and in NCX_Mj, but is Gln in all MHXs) and D^814^ (which is Asp in all MHXs and NCXs except SmMHX, and Glu in NCX_Mj). Interestingly, six of the positions whose functional significance was indicated in both NCX1 and NCX_Mj include serine or threonine in almost all MHX and NCX proteins and in NCX_Mj (Figure 4). Serine and threonine can be used as phosphorylation sites. NCX1 is controlled by phosphorylation (reviewed in [84]), and it will be interesting to determine whether the indicated Ser/Thr residues participate in regulating NCX (or MHX) proteins by phosphorylation. These Ser/Thr residues are located at positions 109 (all the MHXS, NCXs and NCX_Mj have there either Ser or Thr), 140 (a completely conserved Ser), 110, 811, and 838 (almost completely conserved serines), and 810 (an almost completely conserved threonine) (Figure 4 and Additional file 9). There are other sites in which serine or threonine residues (some of which shown to be important for NCX1 function) are almost completely conserved in the MHXs and NCXs, including positions 176, 201, 213, and 818 (Figure 4 and Additional file 9).

Six α-repeat residues whose mutagenesis altered the Ca^2+^ affinity of NCX1 [35] are indicated by red letters on the N1 sequence. A mutations in Thr^103^ altered NCX1 affinity for cytoplasmic Na^+^ ions [72]. It was suggested that this interface of a cytoplasmic loop and TMS2 is important for both Na^+^ transport and secondary regulation by Na^+^ ions (together with the large cytosolic loop). It will be interesting to examine the role of the corresponding residues of MHX proteins.

The large cytosolic loop of NCX1 regulates its activity [38]. Figure 4 shows the position in this loop of the regulatory Ca^2+^-binding domains CBD1 and CBD2, which are responsible for NCX1 activation by cytosolic Ca^2+^ ions [39]. Electrophysiological analysis showed that, similar to NCX1, AtMHX is activated when Ca^2+^ ions are applied to its cytosolic (but not luminal) side [11]. The CBD domains of NCX1 include a high density of negatively charged residues (Figure 4). Regions with relatively high densities of negatively charged residues are marked with red boxes on the central loops of the representative MHX proteins (Figure 4). The large cytosolic loop of NCX1 also includes the XIP peptide responsible for NCX1 inactivation [41] (Figure 4). Electrophysiological analysis showed that AtMHX undergoes an inactivation process with a time scale similar to that of NCX1 [11]. While the XIP peptide of HsNCX1 includes eight basic residues, the corresponding regions of plant MHXs, particularly of the angiosperm, include about four basic residues, most of which are evolutionarily conserved (Figure 4). The comparable region of CrNCX includes five basic residues. It will be necessary to determine if, according to the structural model presented in Figure 4, the large central loop of the MHXs faces the cytosol, if it is essential for MHX transport activity, and whether loop elements with similarity to the XIP or CBD regions of NCX1 play a role in MHX inactivation or regulation by Ca^2+^ ions, respectively.

### Altered mobility under reducing and non-reducing conditions suggests the presence of a disulfide bond in AtMHX

NCX1 includes a disulfide bond between Cys^792^ and a cysteine at position 14 or 20 [52]. Ddisulfide bonds are, however, not essential for NCX1 function [33]. Cys^792^ of HsNCX1 and the equivalent Cys^395^ of AtMHX are conserved in all NCX and MHX proteins (Figure 4). In parallel to the cysteines at position 14 and 20 of HsNCX1, all MHXs possess a cysteine in the N-terminal region that is equivalent to Cys^23^ of AtMHX in most proteins (Figure 4 and Additional file 5). Another cysteine residue - equivalent to Cys^152^ of AtMHX - is conserved in all MHX and NCX proteins. It is possible that some of these conserved cysteines participate in disulfide bond formation in MHX proteins.

The presence of disulfide bonds in NCX1 was demonstrated by comparing its mobility on SDS-PAGE under reducing and non-reducing conditions [52,85,86]. This approach is widely used to monitor the formation of disulfide bonds in proteins [83]. While under reducing conditions (in the presence of β-mercaptoethanol) NCX1 appeared as two bands in the gel [85,86], only a single band was observed under non-reducing conditions [in the presence of N-ethylmaleimide (NEM)]. NEM is a small compound that permanently blocks free cysteines, thereby trapping proteins at their original folding state [83,87]. The difference between the apparent molecular mass of NCX1 under reducing and non-reducing conditions indicated that it includes disulfide bonds [52,85,86].

**Figure 7:**
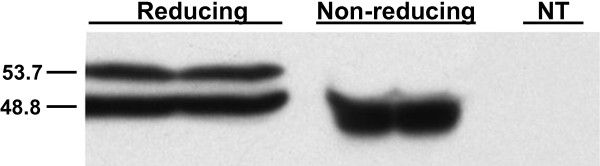
**Western blot analysis of AtMHX under reducing and non-reducing conditions.** Samples of tobacco plants overexpressing AtMHX were treated under reducing (β-mercaptoethanol) or non-reducing (NEM) conditions as detailed in the Methods. The size of the reduced and non-reduced forms (53.7 and 48.8 kDa, respectively) of AtMHX was determined based on size markers. NT – a sample of non-transformed tobacco plants treated under reducing conditions.

Similar to NCX1, we observed that in many cases AtMHX appears as two bands when analysed by SDS-PAGE under reducing conditions (in the presence of β-mercaptoethanol). To explore the presence of disulfide bonds in AtMHX, we treated leaves of tobacco plants overexpressing this protein with β-mercaptoethanol or NEM (see Methods). Following fractionation by SDS-PAGE, the samples were subjected to Western blot analysis using antibodies against a peptide from AtMHX sequence (these antibodies do not recognize the tobacco MHX) (Figure 7). Similar results were obtained in two independent tobacco plants overexpressing AtMHX, and when using antibodies against another AtMHX-derived peptide (data not shown). The two bands observed in the presence of β-mercaptoethanol had an apparent molecular mass of 53.7 and 48.8 kDa. The molecular mass of 53.7 kDa is in excellent agreement with the value predicted for a single AtMHX molecule based on the protein’s length (539 residues). Under non-reducing conditions (in the presence of NEM), we observed only the lower band with apparent molecular mass of 48.8 kDa, whose intensity was doubled. The lower apparent molecular mass of the non-reduced AtMHX protein is in agreement with the general observation that oxidized proteins migrate faster by SDS-PAGE compared with their reduced forms [83]. Similar to the observations in NCX1 [85,86], β-mercaptoethanol reduced only part of the oxidized AtMHX protein, resulting in the appearance of two bands in the gel. As mentioned above, NEM (which was directly applied to frozen plant samples) traps proteins at their original folding state [83,87]. These data suggest that AtMHX includes *in planta* at least one intramolecular disulfide bond.

## Conclusions

The MHX family is limited to plants, and constitutes a sixth family within the CaCA superfamily. Among the plants for which genomic information is currently available, more than one full *MHX* gene was identified only in *O. sativa* and *M. guttatus*. *MHX* gene duplication in *O. sativa* occurred *de novo* before the split between the Indica and Japonica subspecies, which happened 200,000–400,000 years ago. Most likely, following an initial duplication in chromosome 2, one *MHX* paralog translocated to chromosome 11 in Japonica. Some genomes include, in addition to a full *MHX* gene, loci with partial *MHX*-homologous sequences. Genomic and EST data suggest that *MHX* genes underwent functional diploidization in most plant species. Currently, *M. guttatus* is the only plant in which an EST was identified for more than one *MHX*-paralogous gene. The prevalence of uORFs in *MHX* genes is much higher than in most plant genes. These uORFs can limit expression and, potentially, contribute to functional diploidization of the *MHXs*. The currently available chlorophyte genomes do not include proteins with homology to the MHXs, but only to NCX or NCKX proteins. The MHXs are more similar to the NCXs than to the NCKXs. These data are consistent with the suggestion that the MHXs evolved from the NCXs after the split of the chlorophyte and streptophyte lineages of the plant kingdom, which occurred ~1.2 billion years ago.

A structural model of the MHXs, based on the resolved structure of NCX1, implies that the MHXs include nine TMSs. Altered mobility in reduced and non-reduced conditions suggests the presence of disulfide bonds in AtMHX. There are 32 residues that are completely conserved between all MHX and NCX proteins, among which ten are glycines. These conserved residues include an ELGG motif in the last loop and a GXG motif that was implicated in the formation of a tight-turn in a reentrant loop. There are only three residues in which all MHX proteins differ from all NCX proteins analysed. The identification of sequence elements that distinguish between the MHXs and NCXs, or between the MHXs of specific plant groups, can contribute to clarification of the structural basis of the function and ion selectivity of MHX transporters.

## Abbreviations

MYA: Million years ago; NEM: N-ethylmaleimide; NMD: Nonsense mediated mRNA decay; TMS: Transmembrane segment; uAUG: Upstream AUG; uORF: Upstream open reading frame; 5′ UTR: 5′ untranslated region.

## Competing interests

The authors declare that they have no competing interests.

## Authors’ contributions

RG, ME, and KM performed the database searching and editing, and sequenced the cDNA of the tomato, potato and wheat *MHXs*. RG and ME did the sequence alignments and topological analyses. MA and IB helped in data analysis. OS helped in data analysis, did the phylogenetic analyses and wrote the manuscript. All authors have read and approved the manuscript for publication.

## Supplementary Material

Additional file 1 **A table of all proteins analysed.** A table listing the proteins included in the phylogenetic analyses as well as their source organisms, phylogenetic identities, and scores of similarity to AtMHX, HsNCX1, and CrNCX. Click here for file

Additional file 2 **Gene identification.** Describes how each protein sequence was obtained, and provides the accession number of each protein, or of the sequences utilized to obtain it. Click here for file

Additional file 3 **Pairwise similarity scores.** A table listing the pairwise similarity scores of all proteins analysed. Click here for file

Additional file 4 A rooted maximum likelihood phylogenetic tree of all proteins. Click here for file

Additional file 5 Alignment of MHX proteins. Click here for file

Additional file 6 **The 5**′ **UTRs of plant MHXs.** Presents the available 5′ UTRs of plant *MHXs*, their upstream AUG codons, the strength of the Kozak context of these codons, and the uORF peptides. Click here for file

Additional file 7 Alignment of NCX proteins. Click here for file

Additional file 8 Alignment of angiosperm MHX proteins. Click here for file

Additional file 9 Alignment of MHX and NCX proteins. Click here for file

Additional file 10 **A TMpred**-**based prediction of MHX TMSs.** Click here for file

## Data deposition

The data sets supporting the results of this article are included within the article (and its additional files).
