# Supplementary material for: Phylogeny and a structural model of plant MHX transporters
Source: BMC Plant Biol. 2013 May 2;13:75. doi: 10.1186/1471-2229-13-75 (PMC3679957; doi:10.1186/1471-2229-13-75)
Supplement: Additional file 2 — Gene identification. Describes how each protein sequence was obtained, and provides the accession number of each protein, or of the sequences utilized to obtain it. [file 1471-2229-13-75-S2.doc]

**Additional file 2. Identification of the protein sequences**

A description of how each protein sequence was obtained, and the accession numbers of the sequences utilized. The chromosomal location of each *O. Sativa* *MHX* gene is highlighted in blue.

**1. New proteins identified by their similarity to the Arabidopsis thaliana MHX protein**

**1.1 Plantae**

**1.1.1 Division Magnoliophyta (Angiosperm)**

**1.1.1.1 Eudicots**

>gi|6492237|gb|AAF14229.1|AF109178_1 magnesium/proton exchanger AtMHX [Arabidopsis thaliana]

>A.thaliana MASILNQTQELQESSKVLGHLRCENFFLFPGENTLSDGLRGVLYFLGLAYCFIGLSAITARFFKSMENVVKHSRKVVTIDPITKAEVITYKKVWNFTIADISLLAFGTSFPQISLATIDAIRNMGERYAGGLGPGTLVGSAAFDLFPIHAVCVVVPKAGELKKISDLGVWLVELVWSFWAYIWLYIILEVWSPNVITLVEALLTVLQYGLLLVHAYAQDKRWPYLSLPMSRGDRPEEWVPEEIDTSKDDNDNDVHDVYSDAAQDAVESGSRNIVDIFSIHSANNDTGITYHTVADTPPDSATKKGKAKNSTVFDIWKHQFVDAITLETSESKKVDSIYLRIAKSFWHLLLAPWKLLFAFVPPCNIAHGWIAFICSLLFISGVAFVVTRFTDLISCVTGINPYVIAFTALASGTSWPDLVASKIAAERQLTADSAIANITCSNSVNIYVGIGVPWLINTVYNYFAYREPLYIENAKGLSFSLLIFFATSVGCIVVLVLRRLIIGAELGGPRLWAWLTSAYFMMLWVVFVVLSSLKVSGVI

>gi|297828483|ref|XP_002882124.1| hypothetical protein ARALYDRAFT_904236 [Arabidopsis lyrata subsp. lyrata]

>A.lyrata

MASILNQTQELQEASKVLGHVRCENFFIFPGENTLSDGLRGVLYFLGLAYCFIGLSAITARFFKSMENVVKHSRKVVAVDPITKAEVITYKKVWNFTIADISLLAFGTSFPQISLATIDAIRNIGERYAGGLGPGTLVGSAAFDLFPIHAVCVVVPKAGELKKISDLGVWLVELVWSFWAYIWLYIILEVWSPNVITLVEALLTVLQYGLLLVHAYAQDKRWPYLSLPMSRGDRPEEWVPEEIDTSKDENDNDVHDVYSDAAQEAVESGSRNIVDIFSIHSANNNTGITYHTVADTPPDSATKKGKAKNSTVFGIWKHQFVDAITLETSESKKVDSIYLRIAKSFWQLLLAPWKLLFAFVPPCNIAHGWIAFICSLLFISGVAFVVTRLTDLISCVTGINPYVIAFTALAGGTSWPDLVASKIAAERQLTADSAIANITCSNSVNIYVGIGVPWLINTVYNYFAYREPLYIENAKGLSFSLLIFFATSVGCIVVLVLRRLIIGAELGGPRLWAWLTSAYFMMLWVVFVVLSSLKVSGII

>gi|72388787|gb|AAZ68034.1| transporter MHX [Arabidopsis halleri subsp. halleri]

>A.halleri

MASILNQTQELQEASKVLGHVRCENFFIFPGENTLSDGLRGVLYFLGLAYCFIGLSAITARFFKSMENVVKHSRKVVAIDPITKAEIITYKKVWNFTIADISLLAFGTSFPQISLATIDAIRNIGERYAGGLGPGTLVGSAAFDLFPIHAVCVVVPKAGELKKISDLGVWLVELVWSFWAYIWLYIILEVWSPNVITLVEALLTVLQYGLLLVHAYAQDKRWPYLSLPMSRGDRPEEWVPEEIDTSKDDNDNDVHDVYSDAAQEAVESGSRNIVDIFSIHSANNDTGITYHTVADTPPDSATKKGKAKNSSVFDIWKHQFVDAITLETSESKKVDSIYLRIANSFWQLLLAPWKLLFAFVPPCNIAHGWIAFIFSLLFISGVAFVVTRFTDLISCVTGINPYVIAFTALASGTSWPDLVASKIAAERQLTADSAIANITCSNSVNIYVGIGVPWLINTVYNYFAYREPLYIENAKGLSFSLLIFFATSVGCIVVLVLRRLIIGAELGGPRLWAWLTSAYFMMLWVVFVVLSSLKVSGVI

Phytozome: Prunus persica gene ppa004071m.g

>P.persica

MPGNSSTMGQENCESYFIFQGETGLGVGFRTFLYFLGLAYCFVGLSAITARFFQSMESVVSHTRKVVDINPYTGAEIIRYEKVWNFTIADISLLAFGTSFPQISLATIDAIRNLGNLYAGGLGPGTLVGSAAFDLFPIHAVCVVVPRAGELKKIADVGVWLVELFWSFWAYIWLYIILEVWTPNVITIWEALLTVLQYGLLLMHAYAQDKRWPYFSLPLPRSERPEDWVPAEVVTCKSDSGPCNNYSEILEVGEDENRNIVDIFSFHSGSGLGPVYQNVPGTDETPEYSNKDSPEKMSLEDYHVFAIWGQQFVDAVKLKSTESRQLNNLYLRLARISWQLLLVPWRLLFAFVPPYHIAHGWIAFICSLVFISAIAYIVTQLTDLISCVTGINPYVIAFTALASGTSWPDLVASKIAAERQITADSAIANITCSNSVNIYVGIGVPWLIDTAYNFFVYKEPLRIENAAGLSFSLLVFFSTSVGCIAVLVIRRRTLGAELGGPRLWAWITFVFFMLLWLIFVVLSSLKVSGII

GenBank accession number: JF412737 (Solanum tuberosum)

>S.tuberosum

MTSLGNYTTDSTNGHSNILRHEKCDAYLLIHLETALGEGFRTFLYFLGLAYCFIGLSAITARFFRSMESVVKHSRTVETIDPLTNTKIVKNEKVWNYTIADITLLAFGTSFPQISLATIDAIRNIGKLYAGGLGPGTLVGSAAFDLFPIHAVCVVVPKAGELKKISDIGVWLVELFWSFWAYIWLYIILEVWTPNVVTLWESILTVLQFGLLLIHAYAQDKRWPYLSLPLERAERPEEWVPAEVVKHRPLDKVHEPHSEVSQVGEEENSGIVDIFSIHSGEGTGHFYRNLAGEDVTESSTPNNGNIIPEESDILSIWKHQFVDALMLESTESRKLNNIYLRVARIFWQLLLLPWKLLFAFVPPYQIAHGWIAFICSLIFISGIAYVVTKITDLISCVTGINPYVIAFTALASGTSWPDLVASKIAAERQLTADSAIANITCSNSVNIYIGIGVPWLINTLYNYIAYNEPLRIDNAEGLSFSLLVFFSTSVACIGVLVFRRLTIGAELGGPRVWAWVTCIFFMLLWLIFVVLSSLRVSGII

GenBank accession number: JF412736 (Solanum lycopersicum)

>S.lycopersicum

MTSLGNYTTHSTNGHSNILRHEKCDAYLLFHLETALGGGFRTFLYFLGLAYCFIGLSAITARFFRSMESVVKHSRTVETIDPLTNTKAVKNEKVWNYTIADITLLAFGTSFPQISLATIDAIRNIGKLYAGGLGPGTLVGSAAFDLFPIHAVCVVVPKAGELKKISDIGVWLVELFWSFWAYIWLYIILEVWTPNVVTLWESILTVLQFGLLLIHAYAQDKRWPYLSLPLERAERPEEWVPAEVVKYRPLDKVHEPHSEVSQVGEEENTGIVDIFSIHSGEGTGHFYQNLAGEDVTESSTPNNCYIIPEESDILSIWKHQFVDALMLESTESRKLNNTYLRVARIFWQLLLLPWKLLFAFVPPYQIAHGWIAFICSLIFISGIAYVVTKITDLISCVTGINPYVIAFTALASGTSWPDLVASKIAAERQLTADSAIANITCSNSVNIYIGIGVPWLIDTLYNYIAYNEPLRIENAEGLSFSLLVFFSTSVACIGVLVFRRLTIGAELGGPRVWAWVTCIFFMLLWLIFVVLSSLRVSGII

Phytozome: Vitis vinifera gene GSVIVG01028023001

>gi|297743899|emb|CBI36869.3| unnamed protein product [Vitis vinifera]

>V.vinifera

MAWVHEQLPGNILEAYNISVREKCESYLLFSGETSLGDGFRTFLYFLGLAYCFIGLSAITARFFQSMENVVKHKRKVVKIDPRSNTEIRHEKVWNYTIADITLLAFGTSFPQISLAIIDSIQNLGSRYAGGLGPGTLVGSAAFDLFPIHAVCVVVPKAGELKKISDVGVWLVELFWSFWAYVWLYIILEVWTPNVITIWEALLTVLQFGLLLIHAYAQDKQWPYLSLPLPRTERPEDWVPDEAASRKQDKIAGDECSELFQENGNIVDIFSIHSGDGSVYHRISGSEVAEPSNEHFQKNIILEDSHLLSLWKQQFVDAFTLESSESRKLDNIYLWVARIFWQLLLLPWRFLFAFVPPPHIAHGWFAFICSLIFISGIAYIVTKLTDLISCTSGINSYVIAFTALAAGTSWPDLVASKIAAERQTTADSAIANIICSNSVNIYMGIGIPWLINTTYNFIAYREPLGVQNAEGLSFSLLVFFCTSICCIGVLVLRRLTLGAELGGPKIWARLTCVYFMSLWIIFVVLSSLKIYGII

>gi|223537653|gb|EEF39276.1| magnesium/proton exchanger, putative [Ricinus communis]

The sequence highlighted in gray was deleted because it did not have similarity to MHX.

>R.communis

MEVAAHVFNIQLGTKSRQSAIMRASTKLISSAHPTLNFAHQTLESFTWNTILRFHVQASVRTHPPISVYLRMRYHGVKPDFHTFPFLLQSFHSQPHLVSGKQIHTQIHHFGLVHDSFVQTSLINMYSSCGNFSFARQIFDEIAQPDLPSWNSIINASVKVGLVDVARGLFDVMPERNVITWSCMINGFVKCGEYKEALALFREMQMLEVRDVKPNEFTMSSVLSACGRLGALEHGKWAHAYIEKCEMKIDIVLGTSLIDMYAKCGSIDRARLVFDNLGSNKDVMAWSAMISGLAMHGYGEEGLELFSKMVNQGLRPNNVTFLAVLYACVHGGLPLIVPDVELPRKSCITSCGIFRMSNNIGICCSNLKLPACYVMDYKLWVSTSYYYETVVEGAPWNIAFLTCRSHKSPENSENKKKQKRTQGSVGTPPIVGESPVNSLGLLIGTRLQICLMLFQSSLVTVDQTDNDPILPGKKPMALETNYQSGWSRFGLLNILASEKCESYLVFRGETGLSVGFRTFLYFLGLAYCFIGLSAITGRFFRSMENVVKHSRKVVEIDPYSNTEVIRYEKVWNYTIADISLLAFGTSFPQISLATIDAIRNIGNLYAGGLGPGTLVGSAAFDLFPIHAVCVVVPKAGELKKISDLGVWLVELFWSFWAYVWLYIILEVWTPNVITLWEALLTVLQYGLLLTHAYAQDKRWPYLSLPIERTERPEEWVPEEATSDKHQHNAYEEYSEIVQVSEEDSRNIVDIFSIHSPVGTDPVYQKVPETDEAAESSNNYSLSEKDLDVVALWKQQFVDAIMLERPESRKLINSHLRLARISWQLFLVPWRLLFAFVPPYHIAHGWIAFICSLLFISGIAYIVTQLTDLISCVTGINAYVIAFTALAAGTSWPDLVASKIAAERQITADSAIANITCSNSVNIYVGIGVPWLIDTAYNFFAYREPLRVQDAAGLSFSLLVFFSTSVGCIAVLVYRRLTLGAELGGPRIWAWVTSVYFMFLWLIFVVLSSLRVSGII

>gi|224131110|ref|XP_002321003.1| Mg2+ and Zn2+/H+ antiporter, atmhx1-like protein [Populus trichocarpa]

>P.trichocarpa

MASAYNQSDGGRFGDTNIVGHEKCESYFLFRGETTLGEGFRTFLYFLGLAYCFIGLSAITARFFRSMENVVKQSRKVVEIDPYTNAEVIRYEKVWNYAIADISLLAFGTSFPQISLATIDAIQNIGNLYAGGMGPGTLVGSAAFDLFPIHAVCVVVPKAGELKKIADIGVWLVELFWSFWAYIWLYIILEVWTPNVITLWEALLTVLQYGLLLMHAYAQDKRWPYLSLPIPRTERPEEWVPGEVPSPTHENNVYGEENRNVVDIFSIHSNNGTVYQKVPVTDDAAESSNKHFHQEKDLHVLSLWKEQFVDALTLESLETRKLNSIHLRVARISWQLILAPWRLLFAFVPPYHFAHGWIAFIFSLLFISGIAYIVTKLTDVISCVTGINAYVIAFTALASGTSWPDLVASKIAAERQTTADSAIANITCSNSVNIYVGIGVPWLIDTAYNFFMYNEPLRIQNAAGLSFSLLVFFCTSIGCIAVLVLRRLTLKAELGGPRIWAWVTFVYFMLLWIIFVVLSSLRVSGII

Phytozome: Glycine max gene Glyma10g02250

(Note: According to the Phytozome database, chromosome 2 of G. Max includes 1092 bp between positions 1531825 and 1532916 that show similarity to part of the Glyma10g02250 gene).

>G.max

MVPFSNILGHEKCESYLIFGGETTLGDNFRAFFYFLCLAYCFIGLSAITARFFQSMENVVKHTRKVVEVDPVTKTETIRHEKVWNYTIADISLLTFGTSFPQISLATIDAIQNIGKLYAGGLGPGTLVGSAAFDLFPIHAVCVVVPKAGELKKIADLGVWLVELFWSFWAYIWLYIILEVWTPNVVTLWEALLTVLQYGLLLTHAYAQDKRWPYISLPIARDERPEDWVPEETPYFQHEAHAEVDFSDIKHVNEENGDTVDIFSIHSENPTDPLYARVPSINDEAEILDKAKETTLVDTHLLTIWRQQFLDALRLLRPESKKIKNACLRLACIFWQLLILPWRFLFAFVPPCQIAHGWISFICSLLFISGIAYVVTKITDVISCVTGINAYVIAFTALASGTSWPDLVASKIAAERQKTADSAIANITCSNSVNIYVGIGVPWLIDTLYNFIAYREPLRIQNAGGLSFSLIVFFSTSVGCISVLVLRRIIFGAELGGPRLWAWITCAFFMLLWIIFVVLSSLKVSGFI

Phytozome: Manihot esculenta gene cassava4.1_005115m.g

>M.esculenta

MALETQNQSAGSKFGLLNILGRERCESYLLFHGETALDNGFRTFLYFLALAYCFFGLSAITARFFRSMENVVKHSRKIVEVDPDTNTEVIRYEKVWNYAIADISLLAFGTSFPQISLATIDAIRNIGNLYAGGLGPGTLVGSAAFDLFPIHAVCVVAPKAGELKKIADLGVWIVELFWSFWAYIWLYIILEVWTPNVITLWEALVTVLQYGLLLIHAYAQDKRWSYLSLPLGRTERPKEWVPEEITSSKHQPIDYEEYSEILQVDKDESRNVVDIFSIHSNVETDQVYHKVPETDDAVEFSDKNFHSEQDFNVAALWKRQFLDAITLESLESRNMFSIHIRLARTFWEILLAPWRLLFAFVPPYHIAHGWVAFICSLIFISGIAYIVTKITDLISCVTGINAYVIAFTALASGSSWPDLVASKIAAERQTTADSAIANITCSNSVNIYVGIGVPWLIDTTYNFFVYRQPLRIENAEGLSFSLLVFFATSVGCIGVLVYRRLTLGAELGGPRIWAWVTCVYFMLLWLIFVVLSSLRVSGFI

Phytozome: Medicago truncatula gene Medtr1g108330

>M.truncatula

MYKMIGYLSSFLAHDEKCESYLIFSGETNLGNSVRILLYFLGLAYCFIGLSAITSRFFQSMENVVKHSREVVVIDPVTKAETIRHEKVWNYTIADISLLAFGTSFPQISLATIDAIRNLGDLYAGGLGPGTLVGSAAFDLFPIHAVCVVIPKAGELKKIADLGVWIVELFWSFWAYIWLYIILEVWTPNVITLWEALLTVLQYGLLLIHAYAQDKRWPYISLPIARDERPEDWVPEETPKQKSHERVECSEINHFNEENGDTVDIFSIHSENPRDMSYVRVPQIDDAENSDKVIETRLEDTCLLTIWKQQFVDALTVESQESKKMNNIYIRTARIFWQLLLLPWRFMFAFVPPCHIAHGWISFICSLLFISGIAYIVTKITDLISCVTGINAYVIAFTALASGTSWPDLVASKIAAKRQKTADSAIANITCSNSVNIYVGIGVPWLIDTLYNFIAYRQPLRIQNAGGLSFSLIVFFATSVGCISVLVARRIVFGAELGGPRLWAWITCAFFMLLWIIFVVLSSLKVSGFI

Phytozome: Cucumis sativus gene Cucsa.097070

>C.sativus

MASFAIENVESGQAISSISGTGKCESYFIFSIETSLGDALRIFLYFMGLAYCFVGLSAITARFFRSMENVVKHSRKVVEIDPHTNTEIIRYEKVWNFTIADISLLAFGTSFPQISLATIDAIRNIGNLYAGGLGPGTLVGSAAFDLFPIHAVCVVVPKAGELKKISDIGVWLVELVWSFWAYIWLYIILEVWTPKVITLWEALLTVLQYGLLLTHAYAQDKRWPYLSLPLARTERPEEWVPPEIDICKQDNPCREEFQAHENEQRSIVDIFSIHDSDGKVYHEVPGHDIAESSNSNIPEEMDGKADHPHVLKIWKQQFVDALSLETSESKQRNNIYLRSARLCWQLIVAPWRLLFAFVPPYHIAHGWVAFICSLMFISGIAYVLTKFTDLISCVSGINPYVIAFTALASGTSWPDLVASKIAAERQTTADSAIANITCSNSVNIYVGIGVPWLISTTYNFIAYKEPLKIKDAGGLSFSLLVFFSTSVACIVVLVFRRVTLGAELGGPKVWAWITCIFFMVLWVIFVVLSSLKVSDII

Phytozome: Carica papaya gene evm.TU.supercontig_9.258

The sequence highlighted in gray was deleted because the initial alignments indicated that it did not have similarity to other MHX proteins.

>C.papaya

MTSPSHLSNNVQLIKMTWAHWRNAESIIQNFNVLEQEKCESYLIFRSETALSNGFRAFLYFLGIAYCFLGLSAITGRFFRSMEHVVKHTREVVEIDPITNQEITRHEKVWNYTIADISLLAFGTSFPQISLATIDAIRNLGNLNAGGLGPGTLVGSAAFDLFPIHAVCVVVPKGGEMKKIADIGVWLVELFWSFWAYIWLYIILEVWTPNVVTLWEALLTVLQYGLLLTHAYAQDKRWPYLSLPIGRTERPEDWVPEEVASVDHDNNDKFVVDIFSIHSAPSTEYQKVPADENIPEPSGKSSRKEILLEDPNVLTLWKQQFVDIFKVEKSDSRKLDNIYLQLARIVWQSLLAPWRFMFAFVPPYHISHGWIAFVCSLLFISGISYIVTKLTDLVSCVTGINPYVIAFTALACGTSWPDLVASKIAVERQITADSAIANITCSNSVNIYVGIGVPWLINTLYNFFAYREPLGIQNAEGLSFSLLVFFSTSVGCIAVLVFRRLTLGAELGGPRFWAWVTCVYFMLLWIIFVVLSSLRVSGII

Phytozome: Mimulus guttatus gene mgv1a004377m.g

>M.guttatus_1

MDPLNNSTAEIIDGGSNILGVEKCQLYLLFHDETVLSDGFRGFLYFMALAYCFIGLSAITSRFFQSMENVVKHTRTVEEIDPCTNTKTVRHEKVWNYTIADITLLAFGTSFPQISLATIDAIRNLGSLYAGGLGPGTLVGSAAFDLFPIHAVCVVVPKAGELKKISDVGVWLVELFWSFWAYIWLYIILEVWTPNIITLLEALLTVLQFGLLLIHAYAQDKRWPYLSLPIGRTERPEEWVPVETAPYKDRDKYSEIPEDDHESGRIVDIFSVHSDHVYQNLSGSDSGEPSGQSHEDIHVKDNLISIWKQQFVDAFVLEGQESRKLNNTYLRAAKVLWELLLAPWRILFAFVPPCQIAHGWISFISSLIFISGIAYVVTKLTDIISCVTGINAYVIAFTALAAGTSWPDLVASKIAAERQTTADSAIANITCSNSVNIYVGIGVPWLINTMYNYFAYNEPLRIENAGGLSFSLLVFFATSIGCIGGLVFRRLTLGAELGGPKIWAWITCAYFMFLWLIFVVLSSLRVSGFI

Phytozome: Mimulus guttatus gene mgv1a026063m.g

The phytozome database included another Mimulus guttatus MHX gene - mgv1a004654m.g. This gene was identical to mgv1a026063m.g except a 17 aa deletion in mgv1a004654m.g. This suggested that the two genes were alternative- or mis-splicing products. Only mgv1a026063m.g was used here for the alignments.

<M.guttatus_2

MASLNSSSTWDSNLGHERCLFYFIFHFETKLSHGLRGFLYFLALAYCFVGLSAITDRFFRSMENVVKHSRAVEEIDPLTNTKVIKYEKVWNYTIADITLLAFGTSFPQISLATIDAIRNIGSLTAGGLGPGTLVGSAAFDLFPIHAVCVVVPKAGELKRISDIGVWLVELFWSFWAYIWLYIILKIWTPNVITLWEALLTVAQFGLLLIHAYAQDKRWPYVSLPMTRSERPEDWVPAEKNAPYRDGNKTRDNYPEVDEDRNSIIVDIFSIHSANTGFVYENVADTDIHESSSEQCRVDTIPHKDDLLSVWKMQFVDALTLESPESKKLNSKSLRVAKALWKLVLAPWRLLFAFVPPYQIANGWIAFIFSLVFISGIAYIVTKITDLISCVTGINAYIIALTALAAGTSWPDLVASKIAAERQITADSAIANITCSNSVNIYIGIGVPWLIDTLYNYIAYKKPLRIENAAGLSFSLLIFFATSVGCIGVLVFRRLTLGAELGGPRHWAWLTCFYLMLLWLVFVVLSSLQVSNII

Phytozome: Citrus clementina gene clementine0.9_006752m.g

>C.clementina

MASIHTQAIESISRAFNILHYEKCESYLLFRGETSLGDGFRAFLYFLGLAYCFIGLSAITARFFRSMENVVKHSRKVVEIDPVTKAEVVRYEKVWNYAIADIALLAFGTSFPQISLATIDAIRSIGNLYAGGLGPGTLVGSAAFDLFPIHAVCVVVPKAGELKKISDIGVWLVELFWSFWAYVWLYIILEVWTPNVITFWEALLTVLQYGLLLIHAYAQDKRWPYLSLPIARTERPEDWVPQETTSCKDENCDYDECSEILQLGEDENRGVVDIFSIHSPNGAVTSPLYQKVPGSEDVAEISKESFREEINSELPHVHALWKQQFVDSIALESPESRKMNNIYLRLARIFWQSLLVPWRVLFAFVPPYHIAHGWIAFICSLIFISGIAYIVTKLTDIISCVTGINAYVIAFTALASGTSWPDLVASKIAAERQITADSAIANITCSNSVNIYVGIGIPWLIDTAYNFIVYKEPLRVQNAEGLSFSLLVFFCTSVGCIAVLVLRRLTLGAELGGPKLWAWVTSVYFMLLWIIFVVLSSLKVSGII

Phytozome: Eucalyptus grandis gene Egrandis_v1_0.008947m.g

The phytozome database included another Eucalyptus grandis MHX gene (Egrandis_v1_0.011671m.g). The latter sequence was partial and was therefore not used for the alignments. Since this sequence was not 100% identical to the Eucalyptus grandis gene called Egrandis_v1_0.008947m.g, it might be a different MHX gene].

>E.grandis

MQACFASVRLSLSSSLDSLPIFSVSFRRAGTPGDISRSHMMGHGVCESYLLFPGETALGDAFRTFLYFLGLAYCFIGLSAITARFFSSMENVVKQTREVVEIDPQTNSKVVRQEKVWNYTIADITLLAFGTSFPQISLATIDALRNLGELYAGGLGPGTLVGSAAFDLFPIHAVCVVVPKSGELKKISDLAVWAVELFWSFWAYIWLYIILKVWTPDIVTLWEALLTVLQFGLLLVHAYAQDKRWPYLSIPLARGERPEEWVPEEAASYKHVDNVDGPMYHNVPASDLAESSNELHVLALWKEQFVDALMLESAETKKLYSIYVRALRMLWQSLLAPWRLMFALVPPCQIAHGWFAFICSLTFISGIAYVVTKLTDLISCVTGINAYVIAFTALATGTSWPDLVASKIAAERQTTADSAIANITCSNSVNIYVGIGVPWLINTAYNFIMYREPLRIQNAAGLSFSLIVFFSTSVGCISVLVLRRLTLGAELGGPKPWAWLTSVYLMLLWVIFLVLSSLKVSGII

Phytozome: Aquilegia coerulea gene AcoGoldSmith_v1.024094m.g

>A.coerulea

KCESYFLFQGEMALGNGLRAFLYLLGLAYCFFGLSAITARFFRSMENVVKQTRKVVEIDHNTNTEVIRHEKVWNYAIADITLLAFGTSFPQISLATIDAIRNLGQIYAGGLGPGTLVGSAAFDLFPIHAVCVVVPKAGELKKISDIGVWLVELFWSFWAYIWLYIILEVWTPNVVTLWEALITVLQFGLLLIHTYAQDKRWPYLSLPLARTERPEDWVPVEASLDKLDNNDYSEILQLSEEDNIVDIFSIHSEKETGPLYQPVSNSDVAESSNMNFHNKSVLQDFNVFTVWKHQFMDAVMLENPESRKLDNTYLRLARGTWQVILAPWRVLFAFVPPCHIAHGWIAFICSLIFISGIAYIVTKLTDMISCVTGINGYVIAFTALASGTSWPDLVASKIAAERQTTADSAIANITCSNSVNIYIGIGIPWLIDTTYNFIAYREPLRIENADGLSFSLLIFFLTSIGCISVLVLRRLTIGAELGGPRIWAWITSVYFMSLWIVFVVLSSLKVSDII

**1.1.1.2 Monocots**

GenBank accession number: JF412735 (Triticum aestivum)

>T.aestivum

MGSTTRSCDAYLLFNGETLLPNGVRAFLCTVALAYCFIGLSAITARFFKSMESITNHSREVVTVDTETNTPIVKHEKVWNYTIADIALLAFGTSFPQISLATIDAIRNLGQLTAGGLGPGTLVGSAAFDLFPIHAVCVVMPRAGSMKKISDLGVWLVELFWSFWAYIWLYIILEVWTPNVITLWEALLTVLQYGLLLVHAYAQDKRWPYVSIPLVRGERPEDWVPAEDTSLHHDKNCDENSDILPSENDDVVDIFSIHSYSNAGYHHVPEKDIEESSKTTLVVKNTQEDIYWLSVWRQQFVDAVMLESPELKKMDPVCLRFIIICWNSIIAPWKLLFAFVPPYQIAHGWIAFIFSLIFISGIAYGVTNITDQISCVTGLNPYVIAFTALAAGTSWPDLVASKIAAERQVTADSAIANITCSNSVNIYVGIGVPWLINTVYNFFAYQEPLYIDNAAGLSFSLLVFFATSFGCITVLVLRRIVFGAELGGPRLWAWATSAYFMVLWVVFVLLSSLRVSGVI

Phytozome: Zea mays gene GRMZM2G126601

Note: the Phytozome database includes two other Zea mays genes, GRMZM2G404066 and GRMZM2G353990. We checked the sequences of these two genes in the maize genome site at <http://maizesequence.org/index.html>. It became clear that the two latter genes (which are located on chromosomes 6 and 8, respectively) include regions showing similarity to only part of the GRMZM2G126601 gene (which is located on chromosome 4). Therefore, the two latter genes were not included in the analyses.

>gi|226508714|ref|NP_001142176.1| hypothetical protein LOC100274343 [Zea mays]

>Z.mays

MAGAATPPPSSSSSASCGTDAAYLLFRGETLLPDGVHASLYAVALAYCFVGLSAVTARFFKSMEQIMRHSREVVVGVDPRTGAPVVRRDKVWNYTVADIALLAFGTSFPQISLATIDAIRNLGQLTAGGLGPGTLVGSAAFDLFPIHAVCVLMPRAGSKKKISDLGVWLVELFWSFWAYIWLYVILEVWTPRVITLWEASLTVLQYGLLLLHAYAQDKRCPFVSIPFARGERPEDWVPPEDVSAVYYAKCDGIDETLPMGADGNDGIVDIFSAHSYYDAAEYSQLAEEDMEGSSTMDQVVKNTQDNMSWLSIWWQQFVDASMLESPESMKMDSVCLRYTRIFWNLIIAPWKFLFAFVPPYHIAHGWIAFICSLIFISGIAYGVTKLTDQISCVTGVSPYVIAFTALAAGTSWPDLVASKIAAERQVTADSAIANITCSNSVNIYVGIGVPWLIDTVYNFFVYREPLYIDNAAGLSFSLLVFFATSFGCITVLVLRRIILGAELGGPRLWAWVTSVYFMILWVVFVVFSSLRVSGVI

Phytozome: Sorghum bicolor gene Sb05g026100

>gi|242071903|ref|XP_002451228.1| hypothetical protein SORBIDRAFT_05g026100 [Sorghum bicolor]

>S.bicolor

MSNIAMVAAPPSSSCDSDTYLLFHGETLLSTGARASLYTVALAYCFIGLSAITARFFKSMEQIMKHSREVVVIDPHTKEPVVKHEKVWNYTVADIALLAFGTSFPQISLAIIDAIRNLGQLTAGGLGPGTLVGSAAFDLFPIHAVCVVMPRAGSKKKISDLGVWLVELVWSFWAYIWLYVILEVWTPKVITLWEALLTVLQYGLLLLHAYAQDKRWPFVSIPFVRGERPEDWVPPEDVSVDYDNCDDTNETLPISANRNDGIVDVFSAHSYHIAEYSRVPEKDMEGSSTMDQVVKNTQEDISWLSIWWQQFVDASMLESPESRKMHSICLRFTTIFWNLIIAPWKFLFAFVPPYNIAHGWIAFICSLIFISGIAYGVTKLTDQISCVTGVSPYVIAFTALAAGTSWPDLVASKIAAERQVTADSAIANITCSNSVNIYVGIGVPWLIDTVYNFFVYQEPLYIDNAAGLSFSLLVFFATSFGCITVLVLRRIILGAELGGPRLWAWVTSVYFMILWVVFVVFSSLRVSGVI

Phytozome: Brachypodium distachyon gene Bradi4g11030

>B.distachyon

MANISMGSNAPSCNAYLLFYAEKMLPNGVRAFAYTVALGYCFIGLSAITARFFKSMESITNHSREVVTIDPHTNTPIVKHEKVWNYTIADIALLAFGTSFPQISLATIDAIRNLGQLTAGGLGPGTLVGSAAFDLFPIHAVCVIMPRAGSMKKISDLGVWLVELFWSFWAYIWLYIILEVWTPNVITLWEALLTVLQYGLLLVHAYAQDKRWPYVSIPLVRGERPEDWVPAEDTSVDHDKNCGESSEILPRENEDIVDIFSMHSYNNTGYHHVPEKDIEESSKTTLVVKNTQEDTCWLSIWRQQFVDALMLESPEQRKMDSICLRFIIIFWNSIIAPWKLLFAFVPPYQIAHGWIAFICSLIFISGIAYGVTKITDQISCVTGVNLYVIAFTALAAGTSWPDLVASKIAAERQVTADSAIANITCSNSVNIYVGIGVPWLIDTVYNYFVYQEPLYIDNAAGLSFSLLVFFATSFGCITVLVLRRIVLGAELGGPRLWAWVTSTYFMVLWVVFVVLSSLRVSGVI

Phytozome: Oryza sativa gene LOC_Os11g43860

>gi|115486517|ref|NP_001068402.1| Os11g0660000 [Oryza sativa Japonica Group]

>O.sativa_J1

MANINMADTVPSCDTYLLFNGETLLPIGVRAFIYTAVLAYCFIGLSAITGRFFKSMESIMRHSREVVTVDPHTNATIVKHEKVWNYTIADVALLAFGTSFPQISLATIDAIRNLGQLTAGGLGPGTLVGSAAFDLFPIHAVCVVMPRAGSKKKISDLGVWLVELFWSFWAYIWLYIILEVWTPRVITLWEALLTVLQYGLLLLHAYAQDKRWPYVSIPLARGERPEDWVPAEDASVDYDDNYDGIGDILPGQNEDIVDIFSAHSYSNEGYHHVSEEDVEESSTGLTLKNKWEDTHWFSIWWQQFVDAATLESSVSRKMDSTCLRVIGISWNLIIAPWKMLFAFVPPYEIAHGWIAFICSLIFISGIAYGVTKITDQISCVTGVSPYVIAFTALAAGTSWPDLVASKIAAERQITADSAITNITCSNSVNIYVGIGVPWLVDTMYNYFVYQKPLYIDNAAGLSFSLLVFFATSFGCITVLVLRRVILGAELGGPRMWAWATSVYFMILWVVFVVLSSLKISGVI

Explanation how the O.sativa_J2 sequence was obtained:

The sequence available in GenBank was:

>gi|49387612|dbj|BAD25808.1| putative magnesium/proton exchanger [Oryza sativa Japonica Group]

MADTAPSCDTYLLFNGETLLPNGVRAFIYTVVLAYCFIGLSAISGRFFKSMESIMRHSREVVTIDPHTNATIVKHEKVWNYTIADVALLAFGTSFPQISLATIDAIRNLGQLTAGGLGPGTLVGSAAFDLFPIHAVCVVMPRAGSKKKISDLGVWLVELFWSFWAYIWLYIILEVWTPRVITLWEALLTVLQYGLLLLHAYAQDKRWPYVSIPLARGDRPEDWVPTEDASVDYDDNYDGIGDILPGQNEDIVDIFSARSYSNEGYHHVSEKDVEESPTGLTLKNKWEDTHWFSIWWQQFVDAATLESSVSRKMDSTCLSVIGISWNLIIAPWKMLFAFIPPYEIAHGWIAFICSLIFISGIAYGVTKITDQISCVTGVSPYVIAFTALAAGTSWPDLVASKIAAERQITADSAIANITCRYISAHLLSN

Based on sequence similarity to Os11g43860 (the rice MHX gene called here O.sativa_J1), the sequence highlighted in gray was removed, and the sequence highlighted in yellow below was added. The yellow-highlighted sequence was obtained by translation of the genomic sequence provided in:

>gi|41016086|dbj|AP005306.2| Oryza sativa Japonica Group genomic DNA, chromosome 2, PAC clone:P0030D07

and search for sequence similarity to O.sativa_J1 sequence. The resulting sequence was:

>O.sativa_J2

MANINMADTAPSCDTYLLFNGETLLPNGVRAFIYTVVLAYCFIGLSAISGRFFKSMESIMRHSREVVTIDPHTNATIVKHEKVWNYTIADVALLAFGTSFPQISLATIDAIRNLGQLTAGGLGPGTLVGSAAFDLFPIHAVCVVMPRAGSKKKISDLGVWLVELFWSFWAYIWLYIILEVWTPRVITLWEALLTVLQYGLLLLHAYAQDKRWPYVSIPLARGDRPEDWVPTEDASVDYDDNYDGIGDILPGQNEDIVDIFSARSYSNEGYHHVSEKDVEESPTGLTLKNKWEDTHWFSIWWQQFVDAATLESSVSRKMDSTCLSVIGISWNLIIAPWKMLFAFIPPYEIAHGWIAFICSLIFISGIAYGVTKITDQISCVTGVSPYVIAFTALAAGTSWPDLVASKIAAERQITADSAIANITCSNSVNIYVGIGVPWLVDTMYNYFVYKKPLYIDNAAGLSFSLLVFFATSFGCITVLVLRRVILGAELGGPRMWAWATSVYFMILWVVFVVLSSLRISGVI

Explanation how the O.sativa_I1 sequence was obtained:

Most of the sequence was available in:

>gi|125540483|gb|EAY86878.1| hypothetical protein OsI_08262 [Oryza sativa Indica Group]

The sequence highlighted in yellow was obtained by translation of the genomic region upstream to the annotated coding sequence of this gene. The genomic sequence was provided in:

>gi|57015218|gb|CM000127.1| Oryza sativa (indica cultivar-group) chromosome 2, whole genome shotgun sequence

The resulting sequence was:

>O.sativa_I1

MANINMADTAPSCDTYLLFNGETLLPIGVRAFIYTAVLAYCFIGLSAITGRFFKSMESIMRHSREVVTVDPHTNATIVKHEKVWNYTIADVALLAFGTSFPQISLATIDAIRNLGQLTAGGLGPGTLVGSAAFDLFPIHAVCVVMPRAGSKKKISDLGVWLVELFWSFWAYIWLYIILEVWTPRVITLWEALLTVLQYGLLLLHAYAQDKRWPYVSIPLARGERPEDWVPAEDASVDYDDNYDGIGDILPGQNEDIVDIFSAHSYSNEGYHHVSEEDVEESSTGLTLKNKWEDTHWFSIWWQQFVDAATLESSVSRKMDSTCLRVIGISWNLIIAPWKMLFAFVPPYEIAHGWIAFICSLIFISGIAYGVTKITDQISCVTGVSPYVIAFTALAAGTSWPDLVASKIAAERQITADSAITNITCSNSVNIYVGIGVPWLVDTMYNYFVYQKPLYIDNAAGLSFSLLVFFATSFGCITVLVLRRVILGAELGGPRMWAWATSVYFMILWVVFVVLSSLKISGVI

Explanation how the O.sativa_I2 sequence was obtained:

Most of the sequence was available in:

>gi|125540490|gb|EAY86885.1| hypothetical protein OsI_08269 [Oryza sativa Indica Group]

The sequence highlighted in yellow was obtained by translation of the genomic region upstream to the annotated coding sequence. The genomic sequence was provided in:

>gi|57015218|gb|CM000127.1| Oryza sativa (indica cultivar-group) chromosome 2, whole genome shotgun sequence

We were unable to identify in this genomic sequence the coding region of the last ~20 amino acids of the O.sativa_I2 protein, which were missing according to the alignments. The resulting sequence was:

>O.sativa_I2

MANINMADTVPSCDTYLLFNGETLLPNGVRAFIYTVVLAYCFIGLCAITGRFFKSMESIMRHSREVVTVDPHTNATIVKHEKVWNYTIADVALLAFGTSFPQISLATIDTIRNLDQLTAGGLGPGILVGSAAFDLFPIHAVCVVMPRAGKKKISDLGVWLVELFWSFRAYIWLYIILEVWTPRVITLWEALLTVLQYGLLLLHAYAQDKRWPYVSIPLARGDRPEDWVPTEDASVDYDDNYDGIGDILPGQNEDIVDIFSAHSYSNEGYHHVSEKDVEESPTGLTLKNKWEDTHWFSIWWQQFVDAATLESSVSRKMDSTCLRVIGISWNLIIAPWKMLFAFIPPYEIAHGWIAFICSLIFISGIAYGVTKITDQISCVTGVSPYVIAFTALAAGTSWPDLVASKIAAERQITADSAIANITCSNSVNIYVGIGVPWLVDTMYNYFVYKKPLYIDNAAGLSFSLLVFFATSFGCITVLVLRRVILGAELGGPRMWAWATSVY

Phytozome: Setaria italica gene Si026220m.g

Based on similarity to all other plant MHX genes, the last 7 aa of the protein in the phytozome database (highlighted in gray) seemed to be mis-annotated - the correct aa were apparently in another reading frame. We added these aa (highlighted in yellow) at the end of the S.italica sequence used for analyses. However, this modification is not 100% certain.

MSGPSSACEGTYLLFHGETLLSGGVRASLYTVALAYCFIGLSAITARFFKSMEQIMKHSREVVSVDPHTNAPVVKQEKVWNYTIADIALLAFGTSFPQISLATIDAIRNLGQLTAGGLGPGTLVGSAAFDMFPIHAVCVIMPKAGSKKKITDLGVWLVELFWSFWAYIWLYVILEVWTPKVITVWEALLTVLQYGLLLLHAYAQDKRWPYVSIPLVRGDRPEDWVPEEGASVDYDNCNETSEILPGSSDKDIADIFSGHSYHNAEYHKVPENDMECSSTMNNFVKNTREDTSWRSLWRQQFVDAFMLESPESRKMASVCLRLIRIFWNLLIAPWKLFFAFVPPYHIAHGWVAFICSLIFISGIAYGVTKLTDQISCVTGVSPYVIAFTALAAGTSWPDLVASKIAAERQVTADSAIANITCSNSVNIYVGIGVPWLIDTVYNFFVYREPLYIDNAAGLSFSLLVFFATSFGCITVLVLRRIIIGAELGGPRLWAWITSVYFMILWIVFVVFSSLSLWNYKT

The resulting sequence was:

>S.italica

MSGPSSACEGTYLLFHGETLLSGGVRASLYTVALAYCFIGLSAITARFFKSMEQIMKHSREVVSVDPHTNAPVVKQEKVWNYTIADIALLAFGTSFPQISLATIDAIRNLGQLTAGGLGPGTLVGSAAFDMFPIHAVCVIMPKAGSKKKITDLGVWLVELFWSFWAYIWLYVILEVWTPKVITVWEALLTVLQYGLLLLHAYAQDKRWPYVSIPLVRGDRPEDWVPEEGASVDYDNCNETSEILPGSSDKDIADIFSGHSYHNAEYHKVPENDMECSSTMNNFVKNTREDTSWRSLWRQQFVDAFMLESPESRKMASVCLRLIRIFWNLLIAPWKLFFAFVPPYHIAHGWVAFICSLIFISGIAYGVTKLTDQISCVTGVSPYVIAFTALAAGTSWPDLVASKIAAERQVTADSAIANITCSNSVNIYVGIGVPWLIDTVYNFFVYREPLYIDNAAGLSFSLLVFFATSFGCITVLVLRRIIIGAELGGPRLWAWITSVYFMILWIVFVVFSSLKVYGII

**1.1.2 Division Lycopodiophyta**

Explanation how the Selaginella moellendorffii sequence was obtained:

Most of the sequence was available in:

>gi|300166342|gb|EFJ32948.1| hypothetical protein SELMODRAFT_84653 [Selaginella moellendorffii]

The sequence highlighted in yellow was obtained by translation of the genomic region downstream the coding sequence provided in gi|300166342. The final amino acid (R) of this sequence was removed as it was apparently (based on similarity to other plant MHXs) a mistake due to improper prediction of splice-site. The amino terminal region of the resulting sequence, which is missing, could not be certainly identified in the upstream genomic region due to lack of strong sequence similarity with other plant MHX proteins in this region.

The genomic sequence is available as part of the Selaginella moellendorffii v1.0 sequencing project, as part of scaffold 56, in site:

<http://genome.jgi-psf.org/cgi-bin/getScaffold?db=Selmo1&scaffold=scaffold_56&start=1&end=1299822>

The resulting sequence was:

>S.moellendorffii

CPSLLLTQEEQRWRFGTRAFAYCLGLAYCFLGLAVITRVYMRALDSIVRHSRKSVHRDPITGLRIFSERRIWNPTVADITLLALGTCAPQVSLAVIDAFQHIGQTSGKLGAGTLLGSTAFNLFLILAVCVVAPKRFQTKSIRNVGVYIIEVVWSFWAYVWLFLILEIWTPNEITLWEAVLTVAQFPLLVLHAYVQDRNWKYLSIPLRESDAIESSDMNNLHELANVSRSSKFAAMRELVATRPHNFRLFFFSSRAEDVDTSSRDNFAFLREGAETEHFSWKALGTAWKNQFYDAMTVSFFEGRGQRKRTPSTLQLLLHPIVSYWKVSFAFIPPVQLLHGWLAFLGSICFITWISYVVVALSNRINCVTGISSYVLALTVLAAGTSLPNLMASKIAAEEYDTADSAIANINASNCINVYVGFGVPWCVSALYSTGFHQNLVVPVEGLKFLLSVYFSTAITCFVALTARRRLLGGELGGPRKWAWASAIFFFLLWITFLTLACLAGRFT

**1.1.3 Division Bryophyta**

Phytozome: Physcomitrella patens gene Pp1s163_19V6

>P.patens_1

MAGFTNASISAHPSPGVWTNHFANAAVNATAELCSNHIIFKAEPSWWLSARAILYGLCLLYCFVGLATITNLFMQAGGSAMGTIANRTRKIVRHNDESGSEEVVHVRVWNPVIADITLLALGTSAPQISLSIIDAIQQIGQKTNAGLGPETIVGSAAFNLYPILAVCVLVPKAGSVKRIQNVGVWIVELSWSIWAYVWLAIILQVSSPNVVEPWEAMCTVLQFPILMTHAYIQDKGWGNFCRPLWPCMHVSWSPLKDFSLSVHNGTVHDQSTAYSSSPRVDLEQHRSEWELVDLQHQPKQEIRNRVTCDDHPIKPIEWQQVAVAVDKNEDENDPGSNQEFLKQRLAYDIYEQTSMWTYIYSTWKKQFLDVIVFQGQVDDSGKNLALTAVEFIGYLITLPWRFIFAFLPPPSLLHGWAAFLCALAHITVIACFLIKLTNLFGCVTGISKYTLALTVLAAGTSLPDLIASKIAAEIQPTADSAIANINASNCINVYVGIGIPWLMQSFYNWIHLKEEFRVPSAGLGFALVLFFVTFAICLVVIIARRFLFGGELGGPRKWAWVSSFCFLSLWLIFVIFSCLRNYHHL

Explanation how the P.patens_2 sequence was obtained:

The P.patens_2 sequence was identified based on similarity to the P.patens_1 protein. It was composed of the sequences highlighted in yellow below, which were available in two databases, and from translation of the genomic sequence provided for the Physcomitrella patens gene Pp1s7_388V6. The N terminal region could not be clearly identified.

Phytozome: Physcomitrella patens gene Pp1s7_388V6

MACCEADSGGGNRNELVVCISVTSMDASIFCYGRADMAALKPRSSLRFLHYLLLRRVGYDHQPLHGGKELYAMEKIVHQTRKVVRHNYEIGSDEIVHERIWNLVIADITLLALGTSAPQISLAIIDAFQQLGQKTEAGLGPGTIIGSAAFNLYIILAVCVLVPKAGSTKHIRSIGVWIVELTWSMWAYIWLAIILQVSSPDVVEPWEAVCTVLQFPILMMHAYVQDVGLETLCCPSMIVSWKPIRKYFSLAIYDGEIHDQSASFSNLEVFSEQSTSDLERVDLQYLPMKEIRNGVTFDDHRISPFERQQVIGANDISEQLTCGSKNSESNQELVKLRSTNEIDEHTSGRTNACSLWKMQFLDAIFIEKHVDESGKDHSPIAVDCVGHLIILPWRFLFAFLPPPMLLNGWPAFMCALAFITVISCFLIKLANSFGCVTGVSDYVLALTILAVGTSWPDLIASKIAAKHLPTADSAIANINASNCINVYVGTGIPWLLQSFYNKLQLDEEFRVPSVGIGFSLMLFLVTFVLCQIVVVGRRFIFEGELGGPRKWAWASSFYFIFLWLIFVIFSCLRNYNLL

>gi|168000677|ref|XP_001753042.1| predicted protein [Physcomitrella patens subsp. patens]

CPNHIMFEAEPTWLHSSRAVLYAFCIIYCFVGLATITNLYMEARSSMHLHSSISHTIQCSRSDEIVHERIWNLVIADITLLALGTSAPQISLAIIDAFQQLGQKTEAGLGPGTIIGSAAFNLYIILAVCVLVPKAGSTKHIRSIGVWIVELTWSMWAYIWLAIILQVSSPDVVEPWEAVCTVLQFPILMMHAYVQDVGLETLCCPSMIVSWKPIRKYFSLAIYDGEIHDQSASFSNLDKNSESNQELVKLRSTNEIDEHTSGRTNACSLWKMQFLDAIFKHVDESGKDHSPIAVDCVGHLIILPWRFLFAFLPPPMLLNGWPAFMCALAFITVISCFLIKLANSFGCVTGVSDYVLALTILAVGTSWPDLIASKIAAKHLPTADSAIANINASNCINVYVGTGIPWLLQSFYNKLQLDEEFRVPSVGIGFSLMLFLVTFVLCQIVVVGRRFIFEGELGGPRKWAWASSFYFIFLWLIFVIFSCLR

The resulting sequence was:

>P.patens_2

AGLSSALVSAFSSPEKNTIHVVHASATSTAELCPNHIMFEAEPTWLHSSRAVLYAFCIIYCFVGLATITNLYMEARSSAMEKIVHQTRKVVRHNYEIGSDEIVHERIWNLVIADITLLALGTSAPQISLAIIDAFQQLGQKTEAGLGPGTIIGSAAFNLYIILAVCVLVPKAGSTKHIRSIGVWIVELTWSMWAYIWLAIILQVSSPDVVEPWEAVCTVLQFPILMMHAYVQDVGLETLCCPSMIVSWKPIRKYFSLAIYDGEIHDQSASFSNLEVFSEQSTSDLERVDLQYLPMKEIRNGVTFDDHRISPFERQQVIGANDISEQLTCGSKNSESNQELVKLRSTNEIDEHTSGRTNACSLWKMQFLDAIFIEKHVDESGKDHSPIAVDCVGHLIILPWRFLFAFLPPPMLLNGWPAFMCALAFITVISCFLIKLANSFGCVTGVSDYVLALTILAVGTSWPDLIASKIAAKHLPTADSAIANINASNCINVYVGTGIPWLLQSFYNKLQLDEEFRVPSVGIGFSLMLFLVTFVLCQIVVVGRRFIFEGELGGPRKWAWASSFYFIFLWLIFVIFSCLRNYNLL

**1.1.4 Division** **Chlorophyta**

Explanation how the Chlamydomonas reinhardtii sequence was obtained:

Alignment with other NCXs showed that the C.reinhardtii sequence had one region annotated to be an intron that was apparently part of the coding sequence. To correct this, the M highlighted in gray in the first sequence below was deleted. The sequence highlighted in yellow in the subsequent sequence was obtained by translation of the misannotated ‘intron’ sequence. The genomic sequence was found in the C.reinhardtii chromosome_11 sequence available at the Phytozome database.

>gi|159478581|ref|XP_001697381.1| hypothetical protein CHLREDRAFT_192884 [Chlamydomonas reinhardtii]

MAASATGLLPIADVLQQIEALVQVLPLCTDTQEAPCREDPTSARGVAIYTQNSDVVCTSDILLPGFNMLPRWLLGAVYLLFLLYLFAGVAIASDMFMDGIMNICAITKIYKRKNEKGETIYVKEPVWNWVVANITLMALGTSSPEIMLSLVEALLTLGKPAGELGPSCIAGSAAYNFLMLRVYVVTAAWSIWAYIWMLIVYVYWTPNEVTLAEAFLTLGFFVLMVLTAWIVDKQPWKKNNKIISRSDPEAPAALPPPSVTAIVVSPAPEQAPGEAPVRTHAHYRHILAARQRHAAAAHRRLPGAHGEGGDTELQLGSGGHAYGDVVVPADPTKEQVMFRSRAYAFLESAGTARVAVTRVAPEGGSLDHPLRVHYRTEDGDAVAGLDYEAREGTLHFAPGEAYKYVEVRIIDDDMTEPDVHFSIVLTGADAPNGAGREVLVAQERVRVTIVDDDDAGVIGFELPDYEVAFNEKRTFAEVTLVRRRGADGRVTVDYETQDLSAVAGDDYVAAKGTVVFESGEKSARVRLQLLQSFVPEAHKALQLVLSNPEGGAELGKRSACKVTLVRRQFTLMPGAATAAGQKEGLELGNGSIKLVGGAEGGAGSDKGEGEGEEFNLWSAWREQIVSVFSPDEPDEGEVVSWAGLMLQYINITWKLVLFILVPPAEWKGGYPCFFAALGSIVGIVYLVNEAGSLFGCIIGLKEVMVGVSIVAVGTSLPDTLASRIAAVKDPDADAAIGNITGSNGVNVFLGLGLPWAVCSVYYHVRGEKYVTPGGDLEFAVMLYAILGGCGIFILAVARYFGGELGGTKLRQYSIAGLLTVLWLLYLILSGLRAYGNI

The resulting sequence was:

>C.reinhardtii

MAASATGLLPIADVLQQIEALVQVLPLCTDTQEAPCREDPTSARGVAIYTQNSDVVCTSDILLPGFNMLPRWLLGAVYLLFLLYLFAGVAIASDMFMDGIMNICAITKIYKRKNEKGETIYVKEPVWNWVVANITLMALGTSSPEIMLSLVEALLTLGKPAGELGPSCIAGSAAYNFLISAVCTTALPDGQFKKISQLRVYVVTAAWSIWAYIWMLIVYVYWTPNEVTLAEAFLTLGFFVLMVLTAWIVDKQPWKKNNKIISRSDPEAPAALPPPSVTAIVVSPAPEQAPGEAPVRTHAHYRHILAARQRHAAAAHRRLPGAHGEGGDTELQLGSGGHAYGDVVVPADPTKEQVMFRSRAYAFLESAGTARVAVTRVAPEGGSLDHPLRVHYRTEDGDAVAGLDYEAREGTLHFAPGEAYKYVEVRIIDDDMTEPDVHFSIVLTGADAPNGAGREVLVAQERVRVTIVDDDDAGVIGFELPDYEVAFNEKRTFAEVTLVRRRGADGRVTVDYETQDLSAVAGDDYVAAKGTVVFESGEKSARVRLQLLQSFVPEAHKALQLVLSNPEGGAELGKRSACKVTLVRRQFTLMPGAATAAGQKEGLELGNGSIKLVGGAEGGAGSDKGEGEGEEFNLWSAWREQIVSVFSPDEPDEGEVVSWAGLMLQYINITWKLVLFILVPPAEWKGGYPCFFAALGSIVGIVYLVNEAGSLFGCIIGLKEVMVGVSIVAVGTSLPDTLASRIAAVKDPDADAAIGNITGSNGVNVFLGLGLPWAVCSVYYHVRGEKYVTPGGDLEFAVMLYAILGGCGIFILAVARYFGGELGGTKLRQYSIAGLLTVLWLLYLILSGLRAYGNI

>gi|145352821|ref|XP_001420734.1| CaCA family transporter: sodium ion/potassium ion/calcium ion [Ostreococcus lucimarinus CCE9901]

>O.lucimarinus_1

QGGWTLYVIGILYMFLGIAIVCDDYFVASLEKICDKLNLSEDVAGATFMAAGSSAPELFSSGMSLISPDATNEIGISAIVGSAVFNILFIIGATVLCAGQTLELDWRPVTRDCSFYALAITTILLIFHDGEVYWYEGLISVILYFTYVCFMCYNEKIMRWMSGKGAKQPNPEPAPLAMPVLRRADTRRQSRRPTKEETHGVDEGFEWPDSMAAVPLHVLGLPWRYAFHYTIPDCSKRHLEKWFVVAFLASISWISVISYFMVSWAARMGCIIGIPEVVMGMLVVAAGTSIPDALGSIAVAKAGEGDMAVANAVGSNVFDIWLGLGLPWLIILPTKPGGRVLIETSQLMPSIGILFGVLAMYYFALFFNGWRLTRRAGGAFLVAYLLFAGYCIL

>gi|145352010|ref|XP_001420352.1| CaCA family transporter: sodium ion/potassium ion/calcium ion [Ostreococcus lucimarinus CCE9901]

>O.lucimarinus_2

GGIFAYLVGVIYLFIGIAIVCDDFFVASLEKICEALGLSDDVAGATFMAAGSSAPELASSAMSLINSGTDNALGVGTIVGSAVFNILVIIGTTVIFAGQTLKLDWKPLARDCSFYFAAIVGIVATFNGGRVNWWEGLIYVFLYCLYIAFMWKNVYFMKLLDDKFGDYLGRPDAAVEMEEAAGGGLGAQSASKATTSAMSKELSFTGSIYVGFAAQRFKAGLGNKQHRAKALSPGQLRAIRNATEKVTWSHHHVKHAPTEDGVASEDGDDETNPFVMPAEWKDRPIWALSLPWYALFTISIPPCHNPKWEKWYFGSFLASIGWIGIISHFMVEWCARIGCLLKIPAIVMGTTVLAAGTSIPDALSSISVAKDGFADMAVANAVGSNVFDIWLGLGLPWLLYLSWQKPSYITVSTTELLPSSLILLGVLILYV

JGI: Ostreococcus sp. RCC809

>O.RCC809

MALAATARRLARGSETASLGVGRTLLSSSTCKPTKDWELPGGIFLYFIGVFYLFIGIAIVCDDFFVGSLEKICEVLGLSDDVAGATFMAAGSSAPELASSAMSLINSGTDNAIGVGTIVGSAVFNILVIIGTTVIFAGQTLTLDWKPLARDCSFYCAAIIGIVATFNGGRIDWWEGLIYVFLYCVYIAFMWKNAFFMEWLDAKFGHRFGRPDPAVEMEEAVGGNELSSATRSKTKEEGDKLVRELSIAGSMLVGFAAQRFKAGIQGRPKLRPLSPGQLRAIRRDADRVTWNHHHHHRSKAQNATAIGEDTNANAAAGNDDDDDERSPWEMPAELKDRPIWVLSLPWYAMFTTTVPPCHKEKWEKWYLLSFGTSIAWIGVISHFMVEWCARIGCLLEIPPVVMGTTVLAAGTSIPDALSSISVAKDGYADMAVANAVGSNVFDIWLGLGLPWLCYLSWQKPTYILVNTDELLPSALILLGVLIFYIVSVSSNKFRLTKQMGYVYLVFYGVYALYNIVLVWVLDVYKLGE

JGI: Micromonas pusilla NOUM17 (RCC 299)

>M.pusilla_1

MMGVGARFKRSPGARRTHQVLRLGCFGAAAVVLAVFSLLSESDDGSHGDAFTGGGALSTMRRRLTEEGSLYPDDAFGKSGMKKGAIILHVIGVMYTFAGIAIVCDDFFVPALEVLVEKYKIEDDVAGATFMAAGGSAPELFTALIGVFIAKSNVGFGTIIGSAVFNVLFVIGACAFFSKEILVLTWWPLFRDCCWYTFDLLMLYVFFRDEHIDMWESGVLLLFYISYVAFMSQNTRVETFFKRSILRQRDFVATTRHDEERAEKKAQAIGSPATPSKLSQVSPGPGAVTESELARTLTPKGLERTSTPRALVRKESKRLIKSDSSKDPGPDDDDDDDGGIDLDWPEGLGNQVYYVLLCPIMFGLAYTIPNCKKKEHLWPVVFIESILYIGFFSYFMVWWATSIGAVVGISDEVMGYTFLAAGTSVPDLMSSVIVARQGFGDMAVSSSIGSNIFDITFGLPLPWFLWSIIEGGKGIDVISNSLNFSLLLLILMLVSVVVIIAACGWKMTSGLGFSMVVLYGLFLALAIMNSRGVIGGID

JGI: Micromonas pusilla CCMP1545

>M.pusilla_2

MLPSMIRRVRAARAKLSYKDKLRQRQGLMLLAAAVSCVSTYYYGGGSKSFFGRKDWTPSFDFASDGERASLSAITGLGSSSTTARRLLSSSTCSTEDWEDNGGVVLYFIGMFYMFLGIAIVCDDFFVPALEVICEVLDLSDDVAGATFMAAGSSAPELASSTMSLINPNAGSEIGVGTIVGSAIFNILIIIGATVMATGAALQLDWKPVTRDCFFYAAAIAGIVGTFAGGVVNWWEGGIYVCFYGTYIVTMKYNKHLMKWMDKVGGKWMQPEKEFKQISMKEKKAAALAEKAQSSKEPSSHGPGAVVESTDGAAPPKTSIADVVLDLIKQQRKENAEKWRKKIAIVKQDDGTDVTVTPYMSPGHIRAYRKEMKKVAAMLEAKKKKDVREALNLEEGKDKDDGDEEDDAPWWKVPEEKKDWPLWFLSFPWYACFAGTIPPCGEEKYRKYYIMSFFMSIGWIGFITHWMIEWCVVIGCVLNIPNVVMGTTVLAAGTSIPDALSSISVAKDGLADMAVANAVGSNVFDIWLGLGLPWLLYLSWQNKNYIEVNTNELVPSSLILAGVLVLYYSAIFFNKFKLEIWHGKVFFVIYGFYVAYSIILVWILDVYDLDD-NRENGSGTIGGGGGHCVYTRVRGWMD

**1.2 Chromalveolata**

JGI: Emiliania huxleyi

>E.huxleyi

MLNSTAVITNETAPLLNDTCEDGLLLAGPLRTHRNALIYMAALLWTFLGVAIAADAFMLGIETITSQEQAKTVVVDGRKRVYSLKIWNATVANLTLMALGSSAPEILLSLIEIVSSGFYSGELGPSTIVGSAAFNLFIIAAVCVVAVAAPKKVADTGVFSITAAASVFAYVWLAYILLVSSPNVIEVWEGAATLAFFPLLVWLSHRCGIRVPPPTLSAQLRTMLAYLADVGAFSCPDKARPATHVVVAFTRDGKPIRHRSFAREAATQWARGDLARELQRMDPDAADGTSDAQGLASYLASSLGTLRSRAAYRVATTRGATGVAAPQARAAAEQEVAPPPSAAVLSFAVSGAAFSEGAKEASLVVERSGEELARELDWEGSVEYESRDGTAHAGQDYTAVSGRLCFARGQRSAVLAVPLLDDEVAEEDETFFHRPSAGARLGRRSTMAVTIIDDDGPGEVTFAVAETRVRESQRVAVLEVERKRGVQGAVSVGWRTADGTALASRDYVPGRGRLTFAPGVTRAAIEVEIIDDGAYDLADESFAVVLDEPGGGASLGEIGRAVVVIESDDERRALVKQVASLMSLNVDAMQLAGASWREQLAEAVTFEPPRQATTARALDGPLLGGASPPAALSLSCPFAFAGFALALPWRLLFALTPPPRVAGGWACFFVALVGIGVITALVGDLAAHMGCCLGMKPAVTAITFVALGTSLPDTFASRTAAVKERERHADSSIGNITGSNSVNVFLGLGLPWLAAALFWASPRGAAQEASWRDRYRGEAWYSEAMPVAFVVASGDLGFSVAVFTVLALVCLAVLVARRATVGGELGGGAASKYSTATLFTALWLGYIWLSIAKTEGDPRLARLLQQLPPQARALLGA

JGI: Aureococcus anophagefferens clone 1984, and:

>gi|323450711|gb|EGB06591.1| hypothetical protein AURANDRAFT_28918 [Aureococcus anophagefferens]

>A.anophagefferens_1

RAVLYLLGLLWFFLGVSIIADVFMAAIETITSKEKKVPGTDVWNATVANLTLMALGSSAPEILLSVIEIL

TNSFFTGDLGPSTIVGSAAFNMFCIIAICMVSLPGTETRKIEDMSVYTVTAFFSVFAYVWLLFILMVTSP

DVVEIWEAIFTFLFFPILVALAYAADRGYFDTAKISPVSSAHVIQVGSTPFRPYEFSDLLKKLQDPTLEE

SERHDLVKKLAMGQKAKPSRAVLRMNAVRAATGQKVIKADGPDPKVLAKYLEGAAGPKHRPEDVVAFFSD

VNGKINTKYALLESDKAVTLHVMRSPAEGPMTIKWATRDGTAKGSEVGGTGDYETCSGELTFADKENFKT

IVIKVFDDNETEDDEVFYVDLLEVAHGGKAYAPGAFGDASTCEVTIIDDDEPGELSFEPPKEGAEGGPVL

TMAETCGKARVKVGRYNGSAGQVQCDYELVDGTACCGKDFGGTVGFEREGTFVFENTEVEKFIEIPVINT

NRYEGESEFTIVLKNFKCAVERAKFGKHTELKVVIVADTETKDMIDNVQKYLESNDASYNVGSTWGQQFS

DALKVGGGDADDDFKASAGDWVMHIITLPWKVAFAFVPPTSYGGGWVCFYVALTFIGIVTAFIGDLAALM

GCCLGLKDTVTAITFVALGTSLPDAFASKAATINDDSADAAVGNVTGSNSVNVFLGLGLPWSIAAIYWSG

GFAGGSAEKQWRIKYGGKDVAFIVPAGNLGLSVGVFVFCACTCLGVLAYRRKAYGAELGGDRTMANRHAT

LFVMLWFLYVAMSILASEGIV

JGI: Aureococcus anophagefferens clone 1984

>A.anophagefferens_2

RLAPGARLLLPRRRHRERRLHGRHRAHHVRREDRHARRYGQRADREADLPRARLERDHRQPHADGPRVLGAGDPAVHHRDRRQRLLLGRPGAVDDRRLRGVQLDDHPRGLRRGDPRWRVAAHQRLRGLPGHGRLLRLRVPLAFVHPGGEHAGLLRRLGGSSCFGRRPHPGLRRGRGLQVRDARRQRLDVEIVDDAEVEAHSETFFVDLTDATGGATFDATWDGGEDRAVATVVIIDDTDRRGLAGLLIPLINRDKLNLALNDYKSQFRDAVALEGDAPTPGDYAMHVLALPWKVCFACIPPPSYGGGWVCFFVALVYIGGLTLVIGEVATLLGSALGIADSVTAITFVALGTSLPDTFASKTAATADPYADAAVGNVTGSNSVNVFLGLGLPWARKDGAWDHVGFLVPAGSLGVSVTTFSACACATLVVLVLRRKLLGAELGGPRAAKHATAALFVCLWLVYIVVSTSKAYGWI

JGI: Aureococcus anophagefferens clone 1984

>A.anophagefferens_3

GIFAVSVVCVIYLFVGIAIVCDELFVPSLEQIAEDWGLSDDVSGATLMAAGGSAPELATSFIGTFQGSTVGLGTIVGSAVFNVLFVIGMCALATPKELGPLKLTWWPLARDCCYYILTLGTLASFFTTTSTGLITGAEAGIQFSLYFGYVFLMSQNAKLESWVKRKLAKEERYKVSPGDAAADPSDGEAKEEHTTFSAGFLGNLTHADLKMNAGIAVVARIKGDVRATFDSLDADRNGSLDAGEFRKILGLLGDATSYDETELEALRKELDIDHSNTIDFKEFTLWDLTTASPGDALATVLLLPLNGSLALTVPDCRVIGNEHYCYLSFVCSIAWIGAYSFGMVECIVSIGCFTGIPIFIMGLTFLAAGTSVPDLLSSVAVAKQGKGDMAVSSDVESPFCCPGSNIFDVAVGLPLPWLAFTLVHGCPVPVGGHANGNALSVAILLAMVAAVIISIAASGWKMSKGLGFTMFGLYFLFI

JGI: Phaeodactylum tricornutum

>P.tricornutum

MMLSSIRHQTSCTPRQEDDDAAITTSSGMYRRKYHVRAVCQSLAFLTLIGVFSVYNYTFDDSEGDYAVQGVVTPIDTRRVAELALMSPAQRRRAETLVSCDDIEKADPRWLTVFLCIGVLYMFLALAIVCDEFFVPALEEMSSKRRMNLSMDVAGATLMAAGGSAPELFTSLFGTFTESEIGFGTIVGSAVFNVLFVIAMCTIFSKEVLALTWWPLFRDSLFYAIGLVVLSIFVGVTSPEEIELWEAIVLFAMYFLYCVIMYFNADIYHYLTGKVLIYPEDSDDEESTASQEQRQEAAAATRDIERPSLEKEGSANSLASALHLVTLQNDLQLMGQHSFRWQGTFRAGILKLLRDPHTWVETGGVGIVAKIAGDADYIFRKIDANGDGHLDKEELKRLFEALDCHVSPEELTEVFDILDVNKDGVISEEEFNKWYTTSAELIRILSWTQYIIVFPLVFAMVFTIPDVRRPGWGRWCYLSFVLSIAWIGGFAYLMVTWAETIGNTVGIPSVIMGLTVLAAGTSVPDLLSSVIVARRGSGDMAVSSSIGSNIFDILVGLPVPWILYTSWPSKDSTVVIASGKIWISIFVLIGMLVFVIAAVHCQGWKLTKTLGAMMIVFYFAFLAQAILLELPFETCISSP

JGI: Fragilariopsis cylindrus CCMP 1102

>F.cylindrus

MTVIYIIGILYMFLALAIVCDEFFVPALEELSGPRRMNLSMDVAGATLMAAGGSAPELATALIGTFRQSEIGFGTIVGSAVFNILFVIGMCSILAKEVLTLTWWPLFRDTTYYTLGLVMLAVFTGYVTPNMISLWEACVLFAMYLGYILLMWQNKNLYKAITGKVLEYPDEDEENDTEHVIETAASHEHSVEHSANSNGNGNSNGNSKVGDNKEMPTPTEDTSESASASKDSGGGGGMRGALKKELSKSSIHSQVSQLNEITFEEFSEWYKSSMIYERQKQLIEDDIGGVFDNLCPPKGGGVRGWAWYIISLPLVFLMTFTIPDVSRPGMGKWCYVSFIMSIAWIGAFSYFMVDWAELVGNTIGIPSDIMGLTVLAAGTSVPDLLSSVIVARRGQGDMAVSSSVGSNIFDILVGLPVPWILYSAINNGDPVYIGSDGLLRSLIILIAMLVLVVGSIHCQGWKLTKILGGVMFL

JGI: Thalassiosira pseudonana CCMP 1335

>T.pseudonana_1

MAYNTDDDASNDGSDAAADNLWVYVALSFVACVSFWCQSVVTEESLGLGTIVGSEIFNLLIICAGSVYASKSHEHGKKYIVLDRVMVVREVGFYGLSIGLLYLALSERGSEADGEVEHIYISFWKACLLFGGYALYVVVCSNMELCVKCICFLRRRFGFGGKNEASFKENEEATYQHSEDEVEMSGSVRYRSFEDHELHQLPFIYNISTEPIENWDTAESSDDIYTCNSDMNKHNETENHDNTTGEKKRNILSNTIHKSLKLMKANHLLCSDEYPVEMNDVYELTNNEGFREISCFLWQRSIFYSKAYFGLHAFHLRWFSITPTRISSVPDRHDPKKHIIVYPLFDELHVDENRMIINIVNPVDGKRDFTLMAPSKSIFEAIVHAFKLYMEVNQPLRLQGMTELDDDGGDDAARAKTKQGKRDNDADPHVTLIEFPSNASPLEIALWISLYPLRLVMHHTLPDVRHLDRHGDPTSSIGYAYLSTATCLMWLIVGSYAMVTSLEKLAALLRISDSVMGVTVSAAGTSLPAYIASRMAAERGFGNQAVANVFGSNTFNITVGLGLPWVLYIAVTGESYHDLENEGIAESMLILIGTLLLFIVLMLSTKFVLLKWHADIFVVLYIIYVVFALAKG

JGI: Thalassiosira pseudonana CCMP 1335

>T.pseudonana_2

MSVLTIDNNSWGDDYDHDALLTRSEEVDVTASSSAPLWLWVVLSATACASFWCQAVVTEERLVPALNVIATHYNIPSDVAGATLMAAGASSPELFSSLVSLFITHSSLGLGTIVGSEIFNQLVICAGAVYASKTGTLVLDRAIVIREVGFYALGIGLLYFALKDSRVDSEDVEEGVEHIYVSFLDAALLVGGYLMYVGVCANMELIEGWFRRSSGELDGQDLTRLKELDVNGGGDEYENHGYGSITTMANGERSHKGVMRAMPFMHNITHEPVENWNAVAVNMAPSQSATQSRAAHEDPNRRSLLTESIRSSVRRMGDIFMKHSTRPTELQTMYDFQYNEYNQELHCYLWQQSVFYTMAYFGSNAWHLRWFTITPNRVSSIPDRRDAENGRLVYPKFREIFVDEKRLIINIPNPNEGKQDFMLMAPSKTIFDKALSVLGVYTSNMRIQQMENIVNTQVSEESIVSDPKFEDADVHEDLIDFPSEGKITQIIFWILLSPLRFLMHYTLPDVRQLDSRGELKLSDSKRVVHAFAATFMCLVWLVAGSYAMVASLEALAELLDIPDAVIGFTVSAAGTSLPNYVASKVAAENGFGNQAVSNAFGSNTFNIMIGLGLPWTVYIAATGFEPYHSLRNEGIVESIIILAGVLAVFVILMLQTNFVILKWHGILFLGLYVAYIAFAIGQVYRGNDPNN

JGI: Thalassiosira pseudonana CCMP 1335

>T.pseudonana_3

MATNTAEKSTTSRPSQRPFSAGGIHRRKHTHRAIFRSVSFLLLLGGYAVYNGLSGDDGGVGPKTTTAAAAHHRRLQFIDDDHFQSLLQTNQSPRTLQDAASTDNSSSPSCPALETTSPPKAIAYALGTLYMFLALAIVCDEFFVPALEEMSSAHHLNLSMDVAGATLMAAGGSAPELFTSFVGTFQESDIGIGTIVGSAVFNVLFVIGMCTLLSKEVLTLTWWPLFRDSSYYAMGLIVLSILVGVVSAQEVHWWEALLLLALYGGYVLLMAHNRKLYKRLTGKELVLPNEEEQVEGEENGGGHVATANAVENGEAGQINANDNGPTSPTSIAKTPSATRDFRWPGTFRAGVLKLLLHPESYIEKGGIGIVAKIQGDCCSSSILLSSPRTQHSTSSHSFLSLRSRTKNFPNGTSNRRNESSPKSDPSLTNSTQINQGVGAWFKYILVLPLVAALTFTIPDARRPGLGKWCYLSFVLSICWIGIFSYFMVDWAEILGNTIGIPSVVMGYTFLAAGTSVPDLLSSVIVARMGEGDMAVSSSIGSNIFDILVGLPLPWLIFTLWPSTPNVISAKGIWVSIFILIGMLVAIITIVHCQGWKMTRTLGAMMFILYFCFLAQAIIVEYYRDPCF

**1.3 Fungi**

>gi|19114244|ref|NP_593332.1| sodium/calcium exchanger (predicted) [Schizosaccharomyces pombe 972h-]

>S.pombe

MSKTSCFLKKYRLILLWCILGIAYILFWTHRISKAFASVSSTSDSTVHLFERGNTLNDSNDQILLTCNDIKNITPANQCRFAKAYCKGEASGFFDYVEFYFCTINSLRFPVLSIIVGWLIFLFITIGISASDFFSTNLVTISWLLQLPDSVVGVTFLALGNGSPDILSTFAAVRVNSGGMAIGELLGSAFFIVAIVAGSVCLIKPFKIPRRHFLRDVAFLTGTILLVIMFVLHDGSLSIWQSLVMILYYLLYVLFVFFSGSSGVSVVITDENYLPVSLPVYSPVLNSFDDSDSYSSTDSELSEEAFLLPAQASRKTQKIHYINDNDPSNSYSSYQHSHVHDFIHKNNTHSNRVLSQSSGPIVRPSLLAALDFRSSNEEQHPGLRSLDPLNIQDGDLTMHPMHIRHSQSDFYPSGINTPVSGINYPNLGFSANNSVQSLVSEIFRHPTHTDEDFPLPSPSLSSLLFPTLRNFAKKSWYEKLMDVLAVPSVLIFTLALPVYQCPRLAVDPIYHMDVSNCNPSKPTWSRKLRLLQCVFVPFAFVTFSITGGNRLYIYAASSVFSILCITALYYYTDEEKPPKFLPWVSFIGFVLGIIWISTIANEVVGILRALGVIFNLNESILGLTVFAAGNSLSDLIADIMIARSGFPEMAMGGVFGGPTLNILIGIGISSFYSSISNHGNDSVIEIPHSLSITAYFLLACLLLLLIYVPLNRFRVNRVLGLLLFILYIVGTSTNIVVELLKDK

>gi|154314255|ref|XP_001556452.1| hypothetical protein BC1G_05221 [Botryotinia fuckeliana B05.10]

>B.fuckeliana

MPRTSSTKVLSRPPKRSKYNFRAFYLTILIISSLAVISLVADQSAKYRYGSQYGVAQKRALDALDSRRLVKRDEECRLVHHAENKCQFILANCPDETAGLLNYLSLYYCKLPTAQPLAFAILILWLGLLFSTIGIAASDFFCINLSTIASILGMSESMAGVTFLAFGNGSPDVFSTFAAMSSHSGSLAVGELIGAAGFITAVVAGSMALVREFKVGKKTFVRDIGFFIVAASFSMVFLADGALHLWECFVMIGFYLFYVVIVVMWHWYLGGRRRRREREAAARGQYLAMTNEEIEVTEEDGDDEDAPAGERNVRGNEDFAALERGSSPHPRVPEDEDSDEDGDEGRHIAAEVASNMRVIRPSGSRRNTLTPIRPSLVGALEFRSVLSSLQKSRGSHGTPINLRRYSDDPSASFDQIRTSATTSAPASEYASSINPIEIGENDTHHPKLDTVRSRAVSMNAASALKPPDPAAFTVADVPDIGIVAATPTFPQLLRVPSPAPKSPTVSISPPPSFDGSQNDRLFTSSTSRWNDDLLAPPGNESPSVSPRHPRSPNLEPASSQASSTRSRTVQRPQLQIPSSSRDSSRNRTQSPILPFPIYTDSPLLLSANSSKAPSILLPDSEIAPESVNLEHELYCPERPIRWWPYRILPSPKIVLTALFPTLCTWKDKSQWDKFISVISAPSIFLLAITLPVVESESNDQEEINSIDQRRESGTSRRNTGPLLIPDSPSFISEAGSEWAEYQRSRRNTQTENCPRSPISDFNQHNTAAVAISAERHHSHSHQMPTPPTKNIGQSQEDAVHSGPGVENPRDWNRWLVVLQLFTAPLFVVFIFWANSDDGDSKSLIRLALFSILGSLVAFAILALTTTHSRPPKHRFVLCFVGFVVAIAWISTIANEVVGVLKAFGVILGISDAILGLTIFAVGNSLGDLVADITVARLGYPVMALSACFGGPMLNILIGIGVSGAYMTIKEANHKHHKHPNKDIKYKPYEIEVSGTLMVSAITLLITLVGLLIAVPMNKWVMSRKIGWGLIILWSISTMINLAVEVSGVWGDNLDLSTFFS

>gi|50556306|ref|XP_505561.1| YALI0F18040p [Yarrowia lipolytica]

>Y.lipolytica

MAQIRGKLRKALVSLAALSVFVLCVLAIPRSHSISGANRLLNKRDVLPEDECSNVRHIPRADQCEFVKLHCSEDENTFIDYMQVYYCSPYGQRPFLLIMLLLWLLTLFMTLGIAASDFLCPNLGTISSLLGMSESLAGVTFLAFGNGSPDLFSTYSSMKIGSGSLAIGELIGAASFISAVVVGAMALARPFKVARKSFVRDIGFFTAAVLCTMAFISDGKLRRSECILMLIIYMIYVATVVSWHYHYTKKRQAYLSEIRAREFFLDAGQEANVIEDEEVGESSNSVLQDGFDREGFLSLTNSDDASRTSRSPVKSPQTPTANNLSPHPNPHAPHGRSLSPHGRLPDRGRSPLAVERLRTEHAQSRSYSPLDNRSSPIITPVHSHPSSHSHRNVPRIVTHNLDLDEDEEEDRYTGLTSSMRLPTPLHTTHRVHDSAPIRPSLYGALDFRDRINNLNDGEDHHAHNHSDLIHSPLTRRLDTELSPVPSSIEPMWSNVKEVTESAWVVALFPTLTDFWSKSFMGMVSSVITALPVFLLSITIPVVESVVEEPQVPNASEPVSAYSDDASFVSNNTATTNPLLAAESENKVLYNMARRNKILVIVQAMLAPPVINILMLDEVPGVWGTIFCIICSMTLMWLAATNYDRMVQSGQYRFLAFFGFLTSIAWVSHIADVVVGVLKALGAILGISDPVLGLTVFAFGNSLGDLIANTTIAKMGFPMMALSACFGGPLLNVLVGVGVSGLIVTSSPGNAVKNGSYHIEISGTLIISAATLFLTLVITMVMVPLARWHMTKTIGVTIISIWTISTIVNVIFI

>gi|67541919|ref|XP_664727.1| hypothetical protein AN7123.2 [Aspergillus nidulans FGSC A4]

>A.nidulans

MSGSSSPDYKALFLKAEDERKQAEERQRQAEERQRQAEERQRQAEERERQERERNRPTTFEEFIRHCHNLLWRPLQAEAPSRSTTGKIPPPTGKYCPIRLLPWTDCEARQQEIYESVCRHLQSTEEDAKQLFTPLVALEDHGRRFARRPISSEQDLETYERLAVEDHVHDIIAELCKIPNAREEFQLGSGVWFDNHANALDEDVGIDASQTSTARPSRHDQFCIHRVDSNTSTLLTTVEYKPPHKLSVESLREGLRPMDFWQEVVEPDTIPTEEPKKSMYNVARLVGSAIVQEFHVMIQEGLEYSYLTNGLMDVQLWVPYDDPCTLYYDLGDPSMYGTMSVGRLGTPRTRIERTLCLCLMSFRSSCRNQAWRNDARGQLPTWHTSFDSERSQISAAGLPQYPSVEHTSSDHTSPEQTTSEYLPSSSPAGSPVTKGRQVTTQAASRCASSSDQHYLEDSSDSEVEPAASDGRKRTFSQVTSSSPTQQSRPRTDPQVNQSGQSRQHVAQYCTQKCLLGLLQGSTLDPDCPNMELHTLGRSDNRHLISAEDLVEKLKAQLDQDLDHNCTPIGPCGSYGAPFKITCATFGYTIVGKGTTSRLWKEVSSEVDVYRVLQPAQGSAVPVFLGAIDLAQIYFLHGAGEICHMLLMGWGGEGMGNIKLDKTIQRAISRSVKEIRSLGIFHQDLRSENILWNAELKRALIIDFHRCTLDPQLMHKRPGSLKRTRLGHEERESRRLRVVEPGPPLLGLPSVGAMFITTPHAWECFLACLFRLSAH

>gi|302692368|ref|XP_003035863.1| hypothetical protein SCHCODRAFT_232410 [Schizophyllum commune H4-8]

>S.commune

MVADSPATLQYQIDCLREALCQPETEDSWDKIQRAIESLGKICSENSEDYHQVVTSAVRSLSRPLTSAMISERTRLSGSAVDFVGAIATSLERNFETLIPIFIPGLLTLCSRTNKVFGTRARACISIIIEMTQLASLLPYFVQAGKDKSALLRHAAAEAGLACLNSCNPQDLEKESRIRDIETMVRTTAKDANADVRKVGKKIFQAYQALMPDRIAGFAAPLSPTLRKYLELPAGPQALPKKESRPNLRTLVKSKSHAHLSSSASSVRSTSPTLSSSVGVGPRVPERPMSSMSRAPPAPSHAYSKLRNEMGPPPLPHTRSQPAQPPQRPQSTLSRSTSAMERPRSMMLPSVAALRTVAPSSSSELGVAVPRARVTGAVRVRPESIHGMPSAGTTTTTTAGSTVMARRVPAREPSPTDKVKDEKKDKGGHLFIPKRPMSAVDPAMASTSSARSDNGHRIKAPPPPPVLGKGKEKETSKPLPKITAKLTTSARAVSVTAKDRVASQSKPSVSRQASATMQHGSRFAPSRSASGTHPKPSPADEEGAPAFPVTEPEAAQAAPTARPTHKVTASLTISKERHAPRTGGLSQPTQAQLARQRPPVERKVTAPAPSKPGWGRTGAAPPPRARTVSTTAISKAAADAAAKPGPRQRVVSMRPGAPALVKKSSVSSLRVDAAKVPLPPSPMPAEKKKRATPSPAPVEEEPVSVEEEQAPAEQQEQEHLSQVLEEEDVFEEKKVLEQAKPAPEPEPTPVAPEPTPVAAEAVPLPEEQDVEEAGSSAAQEAAENPFQDTTPRKSTPPAEPSNIAFHTTAATPPTNASPMVPTNAGPAITLDYDPNSQYGPNSHYNPTTPARLTSHIHPGDLDKTPISQLLMSIERGFHWTPEPPEPLTPAEAYRDMPVMSGALGQPIPIHLKKQPEGGYDA

**1.4 Eubacteria**

gi|158522375|ref|YP_001530245.1| CaCA family Na(+)/Ca(+) antiporter [Desulfococcus oleovorans Hxd3]

>D.oleovorans

MTAVFDLVVIGFAIYLLTLVANHFFMTSLDGIAARLKLSPSVAGATLMAAGSSAPELAIAMVALFTQGGTHSDIGIGTIVGSAVFNILVITGVCAVIRTAHITLKAIIRDTGFYLAGIGVMLYVFIDGEITPLEPLLLVGAYGIYLIAVFRFPGDSFEVYEHGPEETPLIETRWKTILLWRMVNRTVKQTVGLITGDPGQHYIRAFLASIAIIIVLSKLLVDHAVSLSQIMGLPPVVIGLTVLAAGTSAPDLIASMIAVRRGHGDMAVANAIGSNTFDILIGLGLPWLVALGFTGRSVIPVGTSNLLESVFILIGTVLVLFGFLAVNRQLGRKAGITLLMIYAGYVAWIVAGQ

**2. New proteins identified by their similarity to the Chlamydomonas reinhardtii protein**

>gi|260829777|ref|XP_002609838.1| hypothetical protein BRAFLDRAFT_78683 [Branchiostoma floridae]

>B.floridae

MDLIAWQTNYSAGYVVETLPNGTEPCESWLLLPAENLWPSALRGILYFLAMAYIFIGITIGSDVFMCSIEVITSKKRKVVRWDEERQETVEREVLLWNETIANLTLLALGSSAPEIMLNIIEAVKELGNPPEDGLGPFTIVGSAAFNLLVINAICVASVPSPEIKAVREFGVFVITSLWSMFAYVWMLVVVLWVTPGVIDVWEAWVTLGFTPLLVLTAYAQDSGWWCKRQHSRIHNSEPSIQEDMNVRVIGSPQGRASVLNTRAPTELLALEGRNRNLNDMDRPSQQGQQPTNALSRARFRHAVVRSMLYQKKAPKPKARFAEVVTKMVSIKALTSPAMRRQLAANDMSGKFTFGTHTYSVLESSGAIEVDVLFHRRRLSKLTLLNSMSKLNLVNKSNNGPNNGPVVNGTTTDSGQSSAPSKQDEAKEDVSVEFETRDGTGKTGKDYTHTQGRLVFKETEYRKKIKIPIINDQQYQSDKDFYVILKSPAPREDAALGDPSVARVTIIDDDKPGNFVFEQPIYYGDSRTFKVTATVLRQNGTDGNTSVEYTTMDGTAKGGPFSSPTLDYISNSGVLYFAHGETSKQISINLNKEKMGHRNFVILLRNPSLGSKIGEPGAAVGFLCKDELAERVANVVPMGDTEEEDTSWGGQFRSAMCLESEEDEDGKKIPPSVGQLIMHFVTFFWKVLFAFIPPRSMLGGWPAFVMSLGFITMLTAFIETLGHLLGCVFGVRTSVTGITIIALGTSVPDTFASRTVAIHDVHADAAIGNVTGSNSVNVFLGLGLPWVISTMYHLINGTQYRVLPGNLRFAVFTFLAVGSLCLLMLFVRRKCFGGELGGKKVPKWISALLMFSFWVVFIVLCSLKAYDLLPF

>gi|195999284|ref|XP_002109510.1| hypothetical protein TRIADDRAFT_53617 [Trichoplax adhaerens]

>T.adhaerens_1

MSYCNKTGLLLPILDESNWNQGFRGFLYLAGLLYSFLGVAIIADIFMCAIEVITSQTRRIKISDKDGNMKEVEVRIWNDTVANLTLMALGSSAPEILLSIIEIVGNNFKAGDLGPGTIVGSAAFNLLVITATCIVAIPNPEGRRIKGIKVFAVTATFSIFAYLWLYIVLVARTKNIVDLTEAIITLCFFFVLVLISYFVDRNFFCPRKARRISDTENGHLAFDDSTGRLVPDSFDKSSANSFLKAVKTRTDITAEEAAKLAAAKLQEQEKKSYIWYRVGAARLVSGSKRVTPLLDARLQKIFDDIEMKNKKFSSTADVRDDPETTTRTTIEFTTAACAVMENEKMVKVGISRTGKLNNTIKVRYETLPGTAEPEKDYIPQNDVLVFEPNEKIKYVTIPIVDDNEWEPDENFFIQIWLEGSYDDAIIGEKNSTEITIINDDEPGEIAFNKASYLFKESCGTAKVEIVRKNGSDGTISVNWKTIDGSAINGKDYRGGTGKIEFQHGEMSKLIEIPLIDDDIFENDETFHIELFDPNGGAKLGSRTRTALTIVNDDVYNSLLDKVVALTRINVHRLKLGSQSWGEQFTNAMTVNGGDVENATGMDYFLHFLTFGWKVIFAIIPPPSMLGGWLTFIASLGMIGFLTAIVGDLAGIFGCIVGLRDAVTAITFVALGTSLPDLFASKTAALNEKYADNSIGNVTGSNSVNVFLGLGLPWLIATIYWNSQNKEFAVPAGNLASSVILFTICAVICIALLMLRRIIPSFGGSELGGPTKSKYISGLLLVTLWIVYVVISSLQVYRIIPPIVKT

>gi|321459173|gb|EFX70229.1| hypothetical protein DAPPUDRAFT_328233 [Daphnia pulex]

>D.pulex_1

MTRNNGTLDDYTCSNALLLPLISEFTWSIEVRAFLYLFGLLYCFTGVAIIADIFMGAIEKITSTTRKVFINHRSQEPEVIEVRIWNDTVANLTLMALGSSAPEILLSIIEIVGNNFESGSLGPGTIVGSAAFNLMGISAVCVAAIPGVESRQIKGLKVFAITAVFSILAYLWLIFVLMGVSKNVVEVWEAVVTFLLFPILVYLAYLADKGFPWNKSRVSATTANGKQIELGSIQPGELEAMVDGLTLTKDGQVDPNELAKWVRQASRMGLTGEDAAKLAAYKLITSKEHSRAWYRIGATRIFSGSKKIQPQLSIKLREVYDAINEHPDAVNLSPLIETVPQNKAIIEFHAIACAVPESVGRFPITIVRSGKTDNSVSVRVETIDGSAVGGQDFVTFNQVIQFEAGELEKIVTMEIIDDNQYEPDEQFYIKLSLTGTKAIKQKDVMLGRISIMEVTVLNDDDPGTITFEERGILVKESVGVAQVPVLRKNGADGEVSVRWRTIDKNAVSGKAYIGGEGCLVFKHSEMKRVIEIPISNDMMPEKDECFEVELSDPTGGAVLGSITKLAVTITNDEEFNSVLNRLADMTTANVDSMEVHHSTWTSQIKDAMNVNGGDIENATTSDYVMHFLTFGWKLLFALVPPAGIWGGWLSFFVSLVVIGALTAIVGDLASIFGCLVGLDDAVTAITFVALGTSLPDLFASRAAAMNEKYADNAIGNVTGSNSVNVFLGLGLPWLIASIYHASNGNEFKVEAGSLGFSITVFTITAVLSLALLVTRRCSAACGRGELGGPLGTRYASAIFLLVLWMAYVLLSSLETYGHITGF

>gi|196014843|ref|XP_002117280.1| hypothetical protein TRIADDRAFT_32198 [Trichoplax adhaerens

>T.adhaerens_2

MQLFSALLWVIAVVVLNFKVDPTLGSSIISERDVSTINSTSTPTLSDAEKRLIACNTTTRCKNGLFLPHWPYPKDSKGNILPLSTGERVGRTIVYFFSLFFFFIGVSIISDRFMSAIEIITSKERFIKVKMSNGEYKRISVRVWNETVSNLTLMALGSSAPEILLSIIEICGNNFKPGDLGPSTIVGSAAFNLLIITALCVLAIPDDEIRRIKHLSVFFITASCSVFAYLWLLLILKGISRDVIDIWEAVVTLLFFPILVTIAWVADRQLLSFKKLSKKYRADGKNRAVIVEPDSIIMRSPKEERDSEGFNSVYKNNPMYDVDSIDDVDSEKNDEMRRKKAMQIVKEIKQNHPNATAKEIEELASYEALKYQPKSRAYYRIQATRKLTGSGNVIKQRKMSLPANPDGNVDPEDIEKCSLTQIYFSPEKYTVLENCGTVGVTVERTGNLNNVLTVDYKTQDGTANSGEDYVTTNGTLRFKAGESRKDIKITIIDDDIYEEDEYFYVLLSNAKFVSTTDNEQKCVIESPSTATVVILDDDHPGVFSFSESKYSVIETAGTLSLTVERKSGARGTIRVPYHTKEGTAKGGGDDYEDAVGELEFKNDETSKTIEVHIVDDEEYEKNEIFYVLLGEPYFKDKKRIEKERNHVAKTTGNQPIDVNPSLGPYKEAEVTIIESHEFKNTVDKLLKKANLAMAIGTSTWREQFRAALTVNGGDNDDDDDEETKPTCTDYIMHYLTIFWKLIFALVPPTEIWGGWACFCVSIIMIGILTTFIGDLASHFGCTVGLKDSVTAITFVALGTSLPDTFASKTAAVNDKYADSSIGNVTGSNSVNVFLGLGLAWCVAAVANAIKGEKFYVPSGSLAFSVTIFCAVAIVCIAVLMLRRTKSVGGELGGPRGPKYATAVFFIFLWLLYIILSSLESYCHIPGF

gi|196001263|ref|XP_002110499.1| hypothetical protein TRIADDRAFT_54574 [Trichoplax adhaerens]

>T.adhaerens_3

MAAIPNGSTFYNFSNGFIVEYDGSCSSWLLIIGENFWHPAVRCILYIAAMIYLFVGVAIMSDVFMGAIEVITSKKKTVITYDRETGEQATKEVLIWNETVANLSLMALGSSAPEILLSIVETVSEISDPNSVADGLGSFTIIGSASFNLLIITAVCIVSVPAPHYKRVKEFGVFIITAIWSVFAYVWMLIVTTVWTPNKIDIFEAWITLAFFPLLILTAWAQDNGWWWKRHSQILPNEDKSLTVRVVGGKSRHPSASIIRSTSRELSNVHRESVASAHNSTYDLESVTKLNPGENRSRANSARTSISRSSFVYEDHGKIQVDIKPSNSFARARFRHAAIRSLMGGKRHRRSKSVTDKMVELESGLFNSSEPKMAHFVNIITHRLNEQSNASSRNIAATFVFEIAAYSVREDSGSIDLIVLLNRHRALIKKSQENKNNKTVTFGLGKSTSETIIKSLSSSFKTDKSIKSCDPITEESIQDLESSRYSDEEGDDATQNNFSVDFETRDGGAKQGVNFKYSAGRLNFTKSEYQKTITVDILNDGQYHPNLSFYVILKNPSEYSKLADPSVALVTIIDTNVPGEFQFEKAKYHVDKDSNQISLKVLRKKGVDGTVHLEFATIDGTAHGGIIPEVEENHKDYESKTDMLIFANGETEKEIVINVASHVKLDKNFLVALRNASMGARIGEIGATIVSFDIERDALGDRVAEFAALNGVEGEDVSWAEQFTNALTVGSDIDEFGEESPPSNLDYILHFIAFLWKIIAATIPPRTVLGGWPAFVLSLVYIGFLTAIIEQFGNLLGCVILLKPSVTGITIVALGTSLPDTFASRNAAVHDHSADASIGNVTGSNSVNVFLGLGLPWVLVTTYRQIMFSNSDYAISPGDMQFSVILFTVVAGAGVAILLFRRFVLGGELGGDIKWQYITAATLVLLWAVYITLASLRAYDVIRFQ

>gi|321467196|gb|EFX78187.1| hypothetical protein DAPPUDRAFT_53556 [Daphnia pulex]

>D.pulex_2

MTKNNGTLEEYSCSSGLILPLINEFTWSIEMRAFLYFFGLLYCFMGVAIIAEIFMGAIEKITATTRKVYLSRGVDQEPTFIEVNIWNDTVANLTLMALGSSAPEILLSIIEIVGNNFESGALGPGTIVGSAAFNLMCISAVCIVAIPGVETRKIKGLRVFAITAVFSILAYLWLIFVLVGVSRNVVEVWEAVVTFLLFPLLVGLAYWADKGFPLCWKKPSTISAMMVDGPVLTKDGKMDPDELAKWVRQATHLGLTGEDAAKLAAYKLIMTKHHSHAWYRIGATRVFTGSKKVKPQLSIKLQEVNRNSDSRYYRNGVISIVLLNLPVSENVGKFPVRIIRHGETDTLVCVRVETVDGSAVSGEDFVPVDQVIEFQPKEVVKEIMIEIVDDDQYEPDEQFYLKLSLTHNLSYKHKEVALGRISIMEVTILNDDEPGTITFEERGILVKESVGVAQIPVLRKSGSDGEVSIRWRTIDKKALSGRDFEGGEGVLEFRNAEAKRFIEIPISNDMETQMKDECFEVELYDPSGGAVLGPITKLAVTITNDEDFNAVMDRLAEMTSANVDSMVVHHNDWTSQIKDAMNVNGGDIENATTSDYVMHFLTFGWKLLFALVPPAGIWGGWLSFFVSLGVIGLLTAIVGDLAGIFGCLIGLDDAVTAITFVALGTSLPDLFASRGAAMNEKYADNAIGNVTGSNSVNVFLGLGLPWLIASIYHTSMVCLPT

>gi|156368177|ref|XP_001627572.1| predicted protein [Nematostella vectensis]

>N.vectensis_1

MTCANCTGCVPLVYDKCSKQALGTLFLSFLDESSWNQGLRTFIYLIALLWCFMGVAIVADIFMCAIETITSKTKKVKIAKQNSSEIIEVEVRVWNETVANLTLMALGSSAPEILLSIIEITIMNGFEAGALGPSTIVGSAAFNLLCITGVCVMAVPKFETRRIKSMNVFAVTAITSLLAYIWLFIILSVNTKDVVELWEAILTFLFFPILVIIAYIMDKNYCCPGRVEPEGMLELGDNSHGELFVISKIFLMILNKIMKNEVSQIPDITEDDTAKLVAAHIKAKQPKNYGYYRVGAIRNIGGGHKLKPKMDPRLNMIYEELSEVGFSTSGASTILHGKKDAIKPAVIEFATTKCAVMENDKLVKIGVMRSGNLNVSASVNFETIDGTAESGSDYVAKKDVMVFKPGETLKHLDIEIIDDNEWEENEIFFVKLAPYDMKDAGTVVGKQSITEITIINDDEPGVLSLQKPSFVIKESIGKANFLVERTGGTDGTIKVTWKTTDQSAINGKDYHGGIGELVFEHGEQMKDITIEIMDDKAFEKDECFLLEIMDPTGGATLGKITRSVVTIVNDDEYKRLFDRVVALTHLNLDRLELGSSSWGQQFRDALSVNGGDIENATTMDYIMHFLTLAWKVIFAAVPPCTWAGGWVTFSVALCFIGILTAIVGDLATIFGCLVGLKNEVTAFTFVALGTSLPDLFASKAAAINEKYADACIGNVTGSNSVNVFLGLGTPWVIATIY

>gi|156397050|ref|XP_001637705.1| predicted protein [Nematostella vectensis]

>N.vectensis_2

MAYRTNFSRGYVVEYSPPCFESWLLLPAENLWNDGIRGFLYILGMLYLFLGIAIVADIFMSCIEVITSKKRKVTRYDHEKGESVEIEVFVWNETVANLTLMALGSSAPEILLAVVETGQELALGQTTATDGLGTFTIVGSASFNLLLITAVCVVSVPNGTVKRIREFGVFVVTAVWSMFAYVWMLFVIQWNSPEVIELWEAFMTLAFFPLLVLTAWCTDHGWWCRNRGKILSVDPPQVIVIIFTKGLVDRNSLALLRFVRFVKQARNCDVVSKTSRFSHLQVRVLGFTDAPSSHAMIHRSMELRALEQHRGSVRNSSFDRFRHAALRSVTRSKKLIKINEGEDVEDDNLNRFVFGTSSYSVLKSAKHIDLEIILLQNNNNNKNKEMIYAHNDSIADTISENSFEDGVTPSSPGSSQRFSVDYETHDGSAKNGKEYQTVKGTMAFRAGINKHILRIPILPCEDFSKNKDFYVLLKNPSPEGITDLGEPNLTRITIIDDDMPGEFSFEQASYHANFVTGEATCTVVRRKGCDGTVTLKYHTMNGTAKGGTKNELEAKTCDYEQVTDGLLTFHHGEMSKHITVKINTETQDTNFVVLLHDPSTGARIGNRTATVVHISSGIAMIIQGDEDKDGNELPLNSIDFIMHFLTFFWKVLFAFIPPRPLLGGWPAFVVSLLFIAALTAVIEQLGKLLGCVVDLRNSVTGITIIAIGTSLPDTMASRSAALQDTGADAAIGNITGYNSVNVFLGLGLPWVMSTCYHAARGTVYKVSSGNLAFSVLVFALCGGVCLFLLVLRRYLYKGELGGPTLSKWMTGLLLLFLWFIYVVLSSLMAYGHISGSL

>gi|242016779|ref|XP_002428906.1| predicted protein [Pediculus humanus corporis]

>P.humanus_corporis

MNVTGDLNELQNGEEDKCSESLFFPFLNDIHLNPIIRGILYFFALAYSFVGVSVVTDIFMGAVVTITSQTKKVYLGKFKKKPEESESALNVTATSTHQLDFIEVRVWNDTVANLTLMALGTSAPEILLACMEVIKRNFESGELGPGTIVGSAAFNLLLITAICIISVPNGESRRIRLFNVFIVCSIFSILAYVWLWIILSLFSPHVVELWEAIATFLMFPALIFFAFATDKKWLDIRLLMSKKKRLTRAHEIKMEESKKIFVERKFIKDGKIDKETLYSFFKELKKYPGISNEDAAILAASKIIDSEPQSAATYRMSAIRDLTGGRKLRPRLSLKLQEVYDTINQYPLGSALSLLPEISMKNAIVEFHSATCAVRENIGSFFITVCRHGNLEPSVKVRVESVDGSAKAGDDYVKVDEILVFEPEETEKLVLVKIVDDTQWEPNEEFFLKLSLTEHSGNANVQIGRISIMEITILDDDKPGVISFEKTGILVKESAECVVINVRRKYGADGEVSVKWRTNNDTAISGKDFLGGEGELIFKHREILQTIEIPLLNEIRPEKDSYFFVELFSPSNDASLGDIKVICVTITNDEDFANIIDKMLAMTNLKLKTLSIYRKTWASQIKDAMNVNGGDVKNATATDYVLHFISFNWKVLFSFIPPPSMFRGWLCFIVSLCAIGFVTAVIEDIASLFGCVVGLKDTITAITLVAMGTSLPDTFASRTAALKEQYADTSIGNITGSNSVNVFLGLGLPWLMATIYHTHNGTVFKVPAGTLGFSVLLFTISAVIAIALLIGRRNSSFCGKAELGGSVFFKLLSGGLLIILWVFYIVMSCLKEYRVIDVNF

>gi|313235239|emb|CBY10804.1| unnamed protein product [Oikopleura dioica]

>O.dioica

MDGNVSVVDPGAYPQCAMGMLLPLVDESEMSMSLRIIMYLVGLFWFFIGVAIVADIFMVSIEVITSRETHITILVDGQPEKQKVRFWNATVANLTLMAMGSSMPEIFLNIIEIVFNKFETGALGPGTIVGSAAFNLMVIIGVCTFGIPDGEKRRIDRFYVYGITAFFSIFAYVWLLIILQVTSPGVIDLWEALVTLLMFPLLVGVAYLADRNMLCGQTRTDNDDEGQKQIMEYNPDTPEGIELLGKQRPNSMALVDEFMKDFEGDPLTIDEQAAAIAVASKMQGSFCVVPSFHVTKNLQCPTQGRSRAWYRVQATRTLTAGARIEPNKALSRMARSFSNLPNKNNTSDYAMFNFACTSSSVMENAKQIELKVMRTGNIMMPATCSYETIDGTATKGEDYVEAKGVLEFREKETQKSITVEIIDDDQWEPDETFFVKLFLCVSRLVPNAADPRCKLGDRSINQVTIINDDDPGTFQFKEPAFMCKESQGILKLPVCRTNGADGQVSVEWRVTPITAQHGSDYKEDCGTLVFNHGETQNDITITIIQTPEKEPDETFKVSIVKVSTGAAMGHTRETVVTVIGDEEYKNLVKRVAAQTHVALSKMEIGGDSWYEQFVEAMNVNGGDIENATGFDYIMHLLTFGFKTIFAIIPPSNMGGGFPCFFGSLTMIGVMTVIISDLANIFGCLVGLKPAVNSITLVALGTSLPDLFASKIAATNEPNADDAVGNVTGSNSVNVFLGLGLPWSMAAIYHYCNGSTFNVPPGSLVFSVTIFSICAVCTIAFLFLRRTLSYFGNAELGGPQKQKWFSGIFLICLWLFYVLMSTLQAYEFIPGF

>gi|193657365|ref|XP_001948742.1| PREDICTED: similar to predicted protein [Acyrthosiphon pisum]

>A.pisum

MGHGNTSSYPWGPNDVCPGGLILPILDERLWPIKVRVAVYLFTLLYFLLGISIVADVFMTAIDKITSHTKKVYLAKELKPNGSDVSLRPDQPEVIEVRIWNPTIANLTLMALGTSSPEILLSIIETVGHHFEARKLGPNIIVGSAAFNLLIITSLCTLSLGLNEKRRIHDLKVFLVIGAFSFFAYIWLLLIIEVISPNVIELWEAAATFLLFPILMFFAYATDQNWCGKSTEHNTHQLDLDLGQNKENLIKFVKNVKKYSGITDADAAKLAATKLIDSTHRGAMWYRIGAVRRFTGGKQIEPVLEDHLRQPPTLSQNDETKYAIIEFHAPTIAVNENIGRFPVTIWRHGNLNTKATVRVDSINGTAKEGEDFVKVKEIVEFKENEEEKEIYIEILDDNKWEPDEEFFLRLSVVHHKDLSVKLGCISIMEVTIIDNDEPGIISFGKRGLLVKESTGFVEVPIVRTHGSDSEVSVKWKTIDESAISGRDYVGGEGEIIFKDKEVAKILRVEIINDMCPEKDECFEIKLFETTGGARIGNVNRIAITISSDDDFDSVVDRLMLLTNINIHALELHRQTWAEQIKTAMTVNGGDLTNATTLTYVLHFFSFFWKVVFAFIPPAAVFSGWLRFFTSLTLIGVMATIIGDLATIFGCLIGLDDAVTAITIVALGMSLPDILGACMVTRAETHADEAMIHIAGSIAVKVLMGVGLPWFISALYHYSHGNLFEIPSTRIGFNVLLYSVMAILAVTVLMVRRNVEFFGKAELGGPTKGRYITVAILISLWVIYLSLTCFQPF

>gi|316976164|gb|EFV59500.1| sodium/calcium exchanger 1 [Trichinella spiralis]

>T.spiralis

MLQYNNGTTALIFVENAYPCSDRGLLLPLIDESRWPTATRAFLYLIGLLYSFVGVAILADSFMCAVERITSTKRTLKLQTAIDEETGSVLEQYQEVLIWNPTVANLTLMALGSSAPEILLSIIEIIGNGFKAGDLGPGTIVGSAAFNLFIITAVCILSVGCNSKKRIEDFQVFCVTTGWSVFAYIWLIIVLVLVSPNRIEVWEGLLTLLFFGALLLHAYLVSKKLCSGGRGRGRPTTERDRTPKALRELNRRSWTGPALNGKEKLLDVVEQNRVDRLSRELRRKYPHLTDDSLAEVVAKQIEKEAPHDHLWYRIMAVRRLASSMRSRVRAKPVSEQVCQKLETLSRTGMLRRTQTETGVRSVTNVEFAANAYAVDPTGERRVRLKVVRRGSARKQLVFNYRTMSGSAVQDVHFLKKSETVVFAPQEREKTIRIEVCNPAQWRPGITFWVKLELIPGDKDDRITLGLASAAKIIYPKAAVGESVEFAISDLRVGENEGFARIPISRRASLSTDPRSVFVMTTMFSSASVEWKTENGTAKQGEDFVGEKGELILEPGVVEAYIDIPIVNDYEPEKDETFTVKLLPGKASLGACQSIVVTIVNDDNISRYYAKFEEKVRQSLLTCKLESSTWKEQFLKAVSVNGGETSDASMVDCFAHIIAFPWKVIAAFVPPTTIFGGWLAFIVALALIGLLTAIIGDLAAIFGCLVGLKDSVTAITFVALGTSLPDTFASRLAALQDKTADNAIGNINGSNSVNVFLGLGLPWFIASVYWAARGEAFVVHSGDVGFSVTIYTVLSLICIVMMMLRRFLPALGEAELGGPTIPKWICSVTLILFWFLYILLSALQAYGHISSF

>gi|291222885|ref|XP_002731448.1| PREDICTED: sodium-calcium exchanger-like [Saccoglossus kowalevskii]

>S.kowalevskii_3

MMKLLCSILSRTLSELTTVSRGVGASALLLVVAFLNFHPTLTQETFRNSTYPPVWDGGEEEEVCSRNATA

CKPGVLLPKWTPDPVNVGDVAARATVYFAGLAYMFLGVSIVADRFMAAIEVITSKEKEVTVKRPNGEKVV

VSVRIWNETVSNLTLMALGSSAPEILLSLIEIIGNGFHAGDLGPGTIVGSAAFNLFVIIAICVYAIPDGE

VRRLKHLRVFFVTATCSLFAYIWLYMILAVFSAGIIEVWEALLTFMFFPLLVVVAYLVDTRVFFMKFLRK

KYKAKTMPGESRGNHYPLSHEYDDVEYRGRFEDDELDFEDIKDLDESRQEAIRILRDLKKKHPEADHETL

EQMANYEALNRQTKSRAFYRIQATRKMVGAGNILKKNLDKKRTSIAEVKIEVPQDQNFTRVFFEPAEYTV

FENVGTFNATVVRRGGDMNSTIYVDYRSEDGTANAGSDFHYAEGTLVFKPGETQKNIQLTIIDDDIFEED

EHFYIRLSNVRVGTADGMFESNHAGPQARLVEPAFATVVILDDDHAGIFHFEEKLVKVAETAGELEIKVV

RSSGARGTVIVPYHSIEGTAKGHGDDFEDADGELEFHNDETSKFINVKIIDDEEYEKNENFYVELGEPRL

VKRGQGSDDEDSAYGDKENSGSNHMTEDEKRVAEMGKPRLGDLIKCEVKILESTEFKNTVDKLIKKANLS

LVVGTSSWREQFVEAVTVSSGDEDEDGEEKLPSCSDYVMHFLSIFWKILFAFVPPTDIWGGWACFTVSIL

MIGLLTAVIGDLASHFGCTVGLKDTVTAISFVALGTSVPDTFASKTAAVGDKYADASVGNVTGSNAVNVF

LGIGLAWSAAAIYWAAKGEVFEVKPGSLAFSVTIYSIFAVIAITVLMIRRKLPNTLGELGGPKTTKIITS

CFLVGLWLLYLLLCSFESYCYIEGF

>gi|326437229|gb|EGD82799.1| Na+/Ca2+ exchanger NCX3 isoform [Salpingoeca sp. ATCC 50818]

>Salpingoeca

MVRGLSLALVATTVIVSLSLVAGVHGEPCGDRIVNNTEKPICKGGSGTFLPAFNAYGEDNWSDGLKIFLY

LAGLLWTFSGIGIITDVFMEAIEVITSKEKEVKLKDGRVVHVQVWNPTIANLSLMALGSSAPEIILSVIE

VVAGNFFAGALGPSTIVGSAAFNLLVIFAVCSLAVPQGEVRRLEQYGVFIVTAIFSVFAYVWLIIILVVS

SPNVVEVWEGVLTLLFFPMLLGLSFMADRGYFDFGKRHPASVDNVLRIDNDVEHGHQFHDPYEADTIIKR

IARSNSMTMEDKVNLALAKKLKGERVSRAQFRIDATRKLVGSGGILPDYNKAKLQEALLRDDFFEAHKTK

LAKKNFVGLESLQYSIGEGDGHVNVKILRTGDLSLPLTIEYYTEGDTATMGVDFEETKGVAEFSAGEEEK

VIPIKIIDDDEEEDDEHFYFYIKNPSPEQYLIVPGRNRARITIIDDDHPGVVSLELSKFSCLENVGTIFL

SVLRQNGSKGELKVNYATQDGTAHAPHDYEETKGTLVFEDGETEKVIAINIVDDEEYELDEHFFLTLEES

EDVTFGEVTKCKITILNDDEVTTLADKVTALLNLNLDKFRIGGTDWKSQFVDALAWPEEGSGTFAIVMHL

INIPWKIIAACLPPTLFLNGWVTFGSALIMIGIVTATIGDLASLMGCAMGLEDAVTAITFVALGTSLPDT

FASMSATIASDTADAAITNVTGSNSVNVFLGLGLSWIIAAIYWPVVGKTDEWIATVPPCINAKYSNAVFW

VPSGDLAFSVIIFSLCCIICLGTLVVRRYTVGAELGGAYAKPTAALFLTLWLMYILLSSLSTYEHISL

**3. New proteins identified by their similarity to the Homo Sapiens NCX1 protein**

gi|167537060|ref|XP_001750200.1| hypothetical protein [Monosiga brevicollis MX1]

>M.brevicollis_1

MRPQTWPAWAVLAMLVAACVVPGLAQAPGPEPRPSDPPCSDTVLSQGTPICKGGSGTLLPAFNAYGEADWPEGLKIVLYFVGLLWTFAGVGIITDVFMEAIEVITSRERHVMVDGIETTVLIWNPTIANLSLMALGSSAPEIILSIIEIVAGEFFAGALGPSTIVGSAAFNLFVIIAVCLLALPAMEVRRIESMGVFLVTAIFSIFAYIWLLIILIASSPDVIEIWEGVVTLLFFPILLGLSLMADRGYFDFSRDQKPHPNEEELQVINIRTEDGRNIAFDPYDMAVVLKDVETEALTEDEIANIAAARKIDGTKVSRARFRIDANRRLRGAAPLLPDWQRALLDTQKSRAIKDSYYAHRATALRQEQTFGFMTIEYHVREDAGAVTLTIVRTGDLSQSASIGIETQEGTAKAGSDYVALDRRVEFAAGQAEARVSIEIVDDDEPEDDEFFLARLKQPQPDNYRLIPARELAKIIIVDDDKPGEIEFEQTRMEVEEDCGCCEVVVRRRNGTKGAVACRYFMRNGSAIDGHDYEGSSGELQFQDGQATSIIRIPIIKDNVCEPDEFFTVHLEEVSGSTLGKVTVCRVVILNDDKAQRLLEKVSRRMKINMDRFRVGSSDWKQQFRDAVAWPEREAGVLGIVMHLITLPWKVLAACIPPTVFVHGWVAFGVALCFIGLVTALIGDLASLFGCSLGLADTITAITFVALGTSLPDTFASRSATLASKTADAAVTNVTGSNSVNVFLGLGLSWFIAAVYWPAMGATDQWRARVPTCLQQKHDGAFFYVPAGDLAFSVVIFTCCCLVCLGSLLFRRYKFGAELGGPYSRQFAFFFIGLWIFYVLMSSLQVEGYIASI

gi|167534943|ref|XP_001749146.1| hypothetical protein [Monosiga brevicollis MX1]

>M.brevicollis_2

MKASSTALGLGVLALAFVGASAGSCDGTLLSYWGDEDDKDADFALFFLYLILMLYFFGGVAVTADKFMESIEYITSKTRTTIDSQGRTHTIRVWNETGPGTIVGSAAFNMLMIIAYCMTSEQPLRVEAITVLHFTAGASIFAYIWLYIIVSASSPGEIEIWEAALTTIFFPLFVLVAWLFDTGRISTGAPDWMLDVLPLQRQAKRESRRLGQHVHEHLTEADGIRLDRKAQLRLRRYLRKLRQINPNVNEDILLREAIDAMRGHIQQSDTLRPGVIDMPDKPDERSGRHLASVYAADGVSVHSQSHGPIRGQFSFETDEFYTTNRDATVRIKVRRATSEGEPQIEQPASMLVKIVPGTAKLNFHFAAASGSGNCFKVNFSDKGPEEHEVELRILSPPTYQGTVAFSLQLEAMDEKQALNYKHRICRVEISDKFQFTSVWARITTLAIFADEMVEHNWVEQIRAAIRGDDGDEDANKLVENHLNSNTGDRHLQQTRLELLSSEPESRELEIVPGHAPVAEHRAGGLAPNMVVTEEDGRQVNPPDISFHVPEKTKSRGFVSSLIYVLMLPWNVLFAILTPPGDYFGGWVSFVMSLVYIGFLSAVLGDLATGLGCAIGLKDTVTAITLVALGTSVPDTFASKYAAENDGADPAIGNVTGSNAVNVFLGVGLAWLCGSIYHASQGTTFVVAEGTIAFSVVLFSIFALVWYAVLMLRRGTAAVGGPQSLQGAELGGAQPYRNVTTGLFVGMWLLYIILSALVAYEIIPGF

gi|294953203|ref|XP_002787646.1| sodium/calcium exchanger, putative [Perkinsus marinus ATCC 50983]

>P.marinus

MIGLSLWGVDSPRSLATPVIEQPIPICEHGGSGLVLEDRLMGYVEGSHPQILPLFGDSEQDWPNGLRVVLYFLGLCWCFVGVAIISDFFMGAIEKITSKKKRARLTIQGETKLVTVRVWNDTVANLTLMALGSSAPEILLSIIELFSQDMYSGHLGPSTIVGSAAFNLLVISAVCIMAIPDGQVRAINDIGVFVVTAGCSIFAYIWLLLVLQVSSPDVVDIWEAVVTLVLFPALVVMAFAADKGMFSIKTNVASAHRDHIVGLEDLSPDELVDLMAEVTRRRRLQERCPSQIRQRHGSSLTDDAVMRIIQAERPPRTTRAQHRIQASRMLTRRSGASKRSSVWNSLPAHLVALKDTVEADEEAVRSESTIPVINFKCSRFAVMEGAGRIVLPLVTSRPLERDVVVRYETLQGTATAGEDYVPVIDGKITIRAGESSSDEVTIAIMDDDIVEEDENFWVRVTHVSEGAVIGKLSVAEVTIIDDDEIGELVLEKEELVVEGDVSPSPWPVVDPSEARLWQEAQVTVLRTKGCSGEVRVLYRTESGNAVAPNDYIHTEGELIMDHNQAAGVIKIPLRSTSTRTPHFRLILSKPSGGAKFCSKTDGGHDSLICTIWIKPKSGISQGRTTVTDLLNVHWGKMSLGATNWREQFNEALWVNGSREEQSEAGVGDWVMHVITFPWKIVFAFCPPLEYADGWLTFFISLSMIGGVTAIIADMASLLGCTMDIPDSVTAITLVALGTSLPDTFASRLAAMQDPYADASIGNVTGSNSVNVFLGLGLPWVIGSLYWSAKGATQEWIARVGLDIYTKYPDGGFVVEAGDLSFSVMIFTICAFLCLTVLVARRAKYGGELGGTKHGKRISAAILTYALEVDVTSNGVALNSNLHLNYRDASDVDRGEY

gi|198426482|ref|XP_002122973.1| PREDICTED: similar to solute carrier family 8 (sodium/calcium exchanger), member 4b [Ciona intestinalis]

>C.intestinalis

MATTDVISYYSNGFVVEHVPIDETGNFTDAYCQSWLLIPAENLWPAWFRGFIYIIAIAYLFIGVAIGSDVFMTSIEVITSKKRTIIIWDEELGENTKKEVLVWNETVANLTLLALGSSAPEILLATVETINEILSGNQAKGGGLGFYTIVGSAAFNLLVITGICIISVPSPSHKSIRELGVFILTSMWSLLAYVWLLCVMLWTSPNVVEPWEAWLTLIFFPLLVITAYAQDNGWWMYKLRGQNSVQDASKPRDEDPAMVNGLSSRRGSAVGVKSPEKSRKMNWTNEGVCSVSNGDICGGTPNMGSPALKRHIRRVSIAAPTNPAAIELASISKKDNGRKESERRQVEPEPAYRAFARARFRHAAIWSIGGRKRKPINLNLQPSPKLPVNDPKLLSVVSKIASIRKLYSNNNAIQDESSGNVAFASSAYSTLESAGELTIDVLLHRRKRPKGQTVIPTTNANGKFDTQIMPGVISVDYETREGTAKINKDFKYTSGTLIFPEDTYVQSISIPIINDDQYYPDTDFYVILKNPSGDATLGDPSVSRVTIIDDDKPGDFVLETSQLYADMEKGEVSATVLRQCGSDGVVFVHYATIDGTAHGGETMDLGVDYVHTSGKLEFKHQETSKVITIEINKDITETKNFVIILRNPSQGSHLGEHSAAVVNINLEACGINSMSRVLIDENDTTWVGQIRNAMIVGGEMDDYGNESPPSNTDFLMHAISMPWKIVFALAPTRHAYGGFPAFIISLLMIGCLTAIIEQLGHLLGCVATLKTQVTGITIIALGTSLPDTIASRTAALQDDYADASIGNITGSNSVNVFLGLGLPWVIRTMYFAVKGGQYIVDTDGLDFAVTLFDSFGAVCVVLLILRRYVLGGELGGSTRVKWASGIFLFTLWFIFIIVSSLKAYNYF

gi|291242662|ref|XP_002741228.1| PREDICTED: solute carrier family 8 (sodium/calcium exchanger), member 3-like [Saccoglossus kowalevskii]

>S.kowalevskii_1

MSNSTTDPGAYYCYDKGLILPIVNESTWSIGARTTIYLIGLLWSFMGVSIIADIFMCSIEVITSKTKTVNIANPKAPGGTEEIKVRVWNSTVANLTLMALGSSAPEILLSVIEVVGNGFQAGELGPSTIVGSAAFNLLVITAVCISCLPTGEVRYIKLVKVFALTSFFCIFAYIWLFLVLVVSSPDVVELWEAIFTFLCFPILVILAFVADKDYCGKKEVEDAELARLGIGHPGDRYLTAENVTKEGVADVMKEIGKHPDITPEQAATIAAIEAEKNSPHTKAWYRVNATRNMTGGQKLIPAVDPKLLSAYDQAKMGSAISVGSTLISPEIPIVEFAASSCAVYENEKRVRVYVNRRGNIQPQVIFKFETLDGTAEAGSDYIAQRDTMVFGSNETQKFIDIDIIDDNEWEPDEVFFVKLSVDSKSGAKLGKKNTMQITIINDDEPGTFEFTMPSYLVKESIGVLQVPVSRNNGTDGKVVLKWKTTDMTAISGKDYEGGEGELVFEHGEKQKSIDIPIVDDQEYEKDESFKLELLEVSDGAKLGKTRTSVITIVNDDEYNSLLDRVVSITNVNLDRLRIGNASWADQFKEAMNVNGGDIESATWVDYVMHFLTFGWKVIFALVPPPSFLNSGGWICFTVAISFIGLLTAIIGDLAAIFGCLIDLRESVVAITFVALGTSLPDLFASKTAAVNEKYADASIGNVTGSNSVNVFLGLGLSWLIASIYWTSKGLVFEMEAGALSFSVMIYTICAIMCLSLLVLRRFIPFFGKAELGGPTAGRYSSSIFLILLWIIYILLCSLQAYGYIHF

gi|291242660|ref|XP_002741227.1| PREDICTED: predicted protein-like [Saccoglossus kowalevskii]

>S.kowalevskii_2

MSNSTTDPDAYLCYDKGLILPIVNESTWSTGARITVYLIGLIYCLYGVSIITGIFMSSIEVITSKTKTVNIANPKAPGGTQKLKVRVWNSTVANLTLVTLGSRTTEILLCLIEIVGNGFHAGYIGPSTIIGSAAFNLLVITAVCIACPPIGEVRYIKQIKVSTLTAAFSIIAYIWLLLVLLVITPNIVELWEAICTFICFPILVVIAFMADKGYRGRNVAKDDDKQALLGIGNVTREGVADVMKEMGKHPDITQEQAATIAAIEAEKNSPHTRAWYRVSETRNMTGGQKLIPTVDPKLLLAYDQVKMGSSISIGSTLFPEIPIVEFAAASCAVYEHEKRVRVWINRKGNIHPQIVFKFETLDGTAEAGSDYIAQRDTMVFGSNETQKFIDIDIIDDNEWEPDEVFFVKLSVDSKSGAKLGKKHIMQITIINDEEPGTFEFTMPSYLVKESIGVLQVPVSRNNGTDGKVVLKWKTTDMTAISGKDYEGGKGELVFEHGEKQKSIDIPIVDDQEYEKDESFKLELLEVSDGAKLGKTRTSVITIVNDDELNNLLDRVVSITNVNLDRLRIGNASWADQFKEAMNVNGGDIESATWVDYVMHFLSFGWKVIFALVPPPSFLDSGGWISFCVALTFIGVLTTIIGSLGSIFGCLINMKDSVTGLTFVTLGTCIVDLCVSKKASVGEKYADCCMTGSYSVYVFFGLGLSWVTASIYWTSKGLVFVVDSSSFSFSVMMYIICAIMCLSLLVLRRFTPFFGKAELGGPTAGRYLSSIFLLLLWCLYILLCSFQSYDYIDF

gi|221121648|ref|XP_002154308.1| PREDICTED: similar to predicted protein [Hydra magnipapillata]

>H.magnipapillata

MQFVLTFVFIWFVNAVFANETIIFTNITVKNTTCRTLENLKCKPGVVLPVWKSADSESKAVIAGKAFVYLVAMFYFFLGISIISDIFMSSIEIITSKEKEVLVRDRVTGKKHSVTVKIWNETVANLTLMALGSSAPEILLSVIEVIGQGFKAGELGPSTIVGSAAFNLFMITAVCVVVLPPGEVRRIKHIKVFAFTASCSMFAYIWLYLILSVFSPGVIEIWEGIITFLCFPAMVIAAWAIDRKINFYHLLRKKIRKVKKRGQAFIQTGDGDVVAVSLKSTIKHDSSNDNVDEKDIELLPLEEDDDPLEIINDKKKKAMEAFHRARTKYPNADSITLKKLIEQENIKLQHKSRAFYRVQATHALTGQGNLLRLKDRDNSIELNNKMEEVSASQKDTVLENCVQFEPDQYTVVESCGQCFLTVKRFGNDLSDTLYVDYETSDGTANEGDDYLPVKGTLEFKPNETTKKISITIIDDDLFELDEHFYCKLTAIRYYHVADNTQNKSTKTTIGKANTATIIILDDDYPGIFTFEHSNFEVMESVGVLTLRVLRLIGARGKVSIPYQTVEGSALGGGADYEDSKGEIEFFDDETIKTVDIPIVNREEYEKTKTFTVILGEPKIVGKLNSTITNLSSIKDDDLRKVLEVGKPTLGDHKACEIKIVESKEFRKTIDQMLSAKNKSILGSSSWAQQFKDAFQVEYGGDEEEGDDVEPTYGDYVMHYLSFFWKVLFACVPPTDIWGGWACFTASITMIGLLTAITGDIASHFGCTVGLADSVVAISFVALGTSLPDTFASKVATINDEHADGSIGNVTGSNSVNVFLGIGLAWSIAAIYHASNGNKFEVDPGSLGFSVMLFCLEASVCIGILMYRRSSKNIRAELGGPSISRYITFTTFLLLWIIYLLVSGLESYCYFTLKF

gi|115709819|ref|XP_782358.2| PREDICTED: similar to sodium-calcium exchanger [Strongylocentrotus purpuratus]

>S.purpuratus

MSYRGVPYYDNYSLGYVVEFVKENQTRCSSWLLLPAENLWPAWLRCTLYLMAMFYLFIGIAIISDVFMCSIEMITSKQKTIIKYDAERKVHVKKEVLVWNETVANLTLMALGSSAPEILLNTVETLQNLTEPPNRKDSLGTFTIIGSAAFNLLIITAVCVVSVPSPEAKKIKEFGVFLRMPKTLDGGVREHHLLAMILIVEVIWVLRDLEAGTEDSRPLYLEEEKQQFARARFRHAVVRSMIGGKKHFMAQRIAAGGAAGNQPRLLALVDKVQSLQKASDGALSEDLKGKFTFASPNYTVMESCGKLEIDVLFHRSTPRRTRQLTQENGRAGHNHEIVDEISRSEAYDDCIKGTVYVTYETRDGTAKQGTEFTHAQGKLVFTETEWRKTITVPIINDTQYEANMDFYIILKCPEGGGALGDPSVTRVTIIDDDVPGEFTFDSPNYTADLEMGVVMTTVTRSKGFDGTVTLQYATMDGNAKGDVNLEGGADYRSMAGTLCFKHEEKSKTIEIPVNKESVGLKNFVIVLRNPSIGAKLGEHSAAVANLRPGHAMTLGGDIDDNGKEVPPSKMDCIMHFITFFWKVIFAMVPPKNYFGGWPAFLGSLLFIFALTAVVEQIATLLSCVALIEPSVAGITIIALGTSVPDTFASRTAAIQDQHADAAIGNITGSNSVNVFLGLGLPWIIKVMYLYVNSDQPYYVGTDNLDLAVILFTGVGTCCICIIIGRRYIVGGELGGKKLTKYLTGLFLCLLWVVYVVVASLRSYKII

>gi|300868090|ref|ZP_07112727.1| hypothetical protein OSCI_3540026 [Oscillatoria sp. PCC 6506]

>Oscillatoria

MLISTTPAFKAIAFIDAAIPDFQTLVNGVEIGTEVIVVDSSRGGVEQISDILASRKSIKSIHIVSHGCSG

SLQLGSIWLNLDNLEAYTQDLQQWSAALADRAEILLYGCEVAAGKKGKAFVQQVSQLVGVDVAASINLTG

NKALGGDWDLEFATGEIETPLVFSREVMQTYNYVLMPDIIINEFRRDGQFANTEYVELVLTKDLTSAEMN

SFFVGDSTATTDAKYSAYQFINMATIAPIFKAGTIVAIGGTTAIGTEDTSYNPVPGGTNADWNIQLQIGG

GFLNKVVPAGIAHGDFAATDVVWVDTSSTSTTSVDSITWDNTPGAFGSAAKVQITAPNNGANVEFTNSIT

AVNVTANYTINSSGSLGLPNGGINTVWIDNLRNPMAPLNFIGSETFASNTNYAGTTLGGLSGITYNSTNN

TYYSISDGRNIVGSPDDPRFYTLTIDVSSGALLPGGVAFTGVRTIGNPPPFGSDTSDTEGIALASNATDV

YISSEGIFSGANPTSQPFINTFNISTGQQNQALTIPSKFIASAGATGITSNRAFESLTITPDQQFLFTAT

EQALKQDGTASNSRILRYNLSTGTPDREFLYETDSGNGISELLALDNNTFLAIDRNVTTGVGKLYQLSFE

GATDISSGFDPLPGSGVTAVKKTLVLDLSSFPLGTNFEGMTFGPPQANGKRSLILVSDNNFGAGFGIPTA

FAAFTGNNAPVLDNSLSPTLTSINKNIAPASNLGTVVSSLIGGGAVTDPDTVDTQGMVVTSVDNSNGSWE

YSTDNGSNWTSLAPTTTASVLLAGNALIRFLPSLNYSGIANINYRAWDGTTGTNGGTVDISVASSVGGVF

AFSNAIETATITVNDPGIFSFSSPTYSVNENGATATITVERTGSGNVAVNVSYTTSDGSANAAIDYTAVN

GTLSFGSGEINKNFTIPITNDTLVEGDETVNLSLTSPTNGATLGTQNTATLTINDDDVADPGIFTFSNPT

YSVNENGLTATITVNRTGSANVAANVSYTTSDGSANAGSDYTTINGSLSFGIGEINKIFTIPITNDTLVE

GNETVNLSLNSPTNGATLGANSQAVLTIVDTTPTPTPVPTPIPTPSVTPTPTPIPSNTPIPTPIPSVTPA

PSVTQTPSVTPTPIPIPSLTPTPSATPTPSVTPIPSTTPTPSVTQTPSFTPIPTPILSVTPTPSVTPTPS

FTQTPSVTPTPTPIPSLTPTPSVTPTPSFTQTPSVTPTPTPIPSATPIPSNTPIPSATPTPSATPIPSVT

LTPTLTLIPSHFDETDCICKQISLPSLSSIPDLNSTDSTLNGTDNDDILIGSNANDAINGFGNNDILFGQ

LGNDNIYGGFASEFPIGSESDRDLLLGNEGNDYINGNAGNDTIFAGKDNDVAIAGKDDDLIWGDRGNDTI

IGDEGADSLFGGTFNPFDPDLNGRDLLFGGTGNDFLSGQEGEDTLLGGEDNDTIQGGKDDDVILGGDRDD

LLWGDRGDDTICGGDGNDTIFGDIGSAIALAANGEEDCICGGSGNDLIFGGEGDDRLDGDEGNDTLYGGK

DNDSLRGGSGEDWLCGDLGDNILVGGSGSDRFVLGLGGSDAIADFTVGEDFLILRGGLTFEQLSVSQDVG

GSLIAIATTGQVLATLSGVQANLITQDNFRLIG

**4. Proteins used as markers for distinct families of the CaCA superfamily**

**4.1 The CAX family**

>gi|15224460|ref|NP_181352.1| CAX1 (cation exchanger 1); calcium ion transmembrane transporter/ calcium:cation antiporter/ calcium:hydrogen antiporter [Arabidopsis thaliana[

>AtCAX1_CAX

MAGIVTEPWSVAENGNPSITAKGSSRELRLGRTAHNMSSSSLRKKSDLRVIQKVPYKGLKDFLSNLQEVILGTKLAILFPAIPAAIICTYCGVSQPWIFGLSLLGLTPLAERVSFLTEQLAFYTGPTLGGLLNATCGNATELIIAILALTNNKVAVVKYSLLGSILSNLLLVLGTSLFCGGIANIRREQRFDRKQADVNFFLLLLGFLCHLLPLLVGYLKNGEASAAVLSDMQLSISRGFSIVMLISYIAYLVFQLWTHRQLFDAQEQEDEYDDDVEQETAVISFWSGFAWLVGMTLVIALLSEYVVATIEEASDKWNLSVSFISIILLPIVGNAAEHAGAVIFAFKNKLDISLGVALGSATQIGLFVVPLTIIVAWILGINMDLNFGPLETGCLAVSIIITAFTLQDGSSHYMKGLVLLLCYFIIAICFFVDKLPQKQNAIHLGHQAMNNVVTATGGGVFSS

>gi|18399970|ref|NP_566452.1| CAX2 (CATION EXCHANGER 2); calcium:cation antiporter/ calcium:hydrogen antiporter [Arabidopsis thaliana]

>AtCAX2_CAX

MSCCKVPVLIEAQVEMVSANELENKSLFRQEEDATQTKEASLMEQGSLSTSFPQHTPKAPKNSVLNSIKI

VIFCNKLNLLLPFGPLAILVHYMIDSKGWVFLLTLVGITPLAERLGYATEQLACYTGPTVGGLLNATFGN

VTELIISIFALKNGMIRVVQLTLLGSILSNMLLVLGCAFFCGGLVFYQKDQVFDKGIATVNSGLLLMAVM

GILFPAVLHYTHSEVHAGSSELALSRFSSCIMLIAYAAYLFFQLKSQSNSYSPLDEESNQNEETSAEDED

PEISKWEAIIWLSILTAWVSLLSGYLVDAIEGASVSWNIPIAFISTILLPIVGNAAEHAGAIMFAMKDKL

DLSLGVAIGSSIQISMFAVPFCVVIGWMMGQQMDLNFQLFETAMLFITVIVVAFFLQEGSSNYFKGLMLI

LCYLIVAASFFVHEDPHQDGI

>gi|30693547|ref|NP_190754.2| CAX3 (CATION EXCHANGER 3); calcium:cation antiporter/ calcium:hydrogen antiporter/ cation:cation antiporter [Arabidopsis thaliana]

>AtCAX3_CAX

MGSIVEPWAAIAENGNANVTAKGSSRELRHGRTAHNMSSSSLRKKSDLRLVQKVPCKTLKNILSNLQEVI

LGTKLTLLFLAIPLAILANSYNYGRPLIFGLSLIGLTPLAERVSFLTEQLAFYTGPTVGGLLNATCGNAT

ELIIAILALANNKVAVVKYSLLGSILSNLLLVLGTSLFFGGIANIRREQRFDRKQADVNFFLLLMGLLCH

LLPLLLKYAATGEVSTSMINKMSLTLSRTSSIVMLIAYIAYLIFQLWTHRQLFEAQQDDDDAYDDEVSVE

ETPVIGFWSGFAWLVGMTIVIALLSEYVVDTIEDASDSWGLSVSFISIILLPIVGNAAEHAGAIIFAFKN

KLDISLGVALGSATQISLFVVPLSVIVAWILGIKMDLNFNILETSSLALAIIITAFTLQDGTSHYMKGLV

LLLCYVIIAACFFVDQIPQPNDLDVGLQPMNNLGEVFSA

>gi|30679280|ref|NP_568091.2| CAX4 (CATION EXCHANGER 4); calcium:cation antiporter/ calcium:hydrogen antiporter/ cation:cation antiporter [Arabidopsis thaliana]

>AtCAX4_CAX

MSSISTESSSNLSLLENGGGGSDKPTAETSRRVRRTVSASSLIRKRSDLKLISRVRWEFMRRILTNLQEV

LLGTKLFILFPAVPLAVVAHRYDCPRAWVFALSLLGLTPLAERISFLTEQIAFHTGPTVGGLMNATCGNA

TEMIIAILAVGQRKMRIVKLSLLGSILSNLLFVLGTSLFLGGISNLRKHQSFDPRQGDMNSMLLYLALLC

QTLPMIMRFTMEAEEYDGSDVVVLSRASSFVMLIAYLAFLIFHLFSSHLSPPPPPLPQREDVHDDDVSDK

EEEGAVIGMWSAIFWLIIMTLLVALLSDYLVSTIQDAADSWGLSVGFIGIILLPIVGNAAEHAGAVIFAF

RNKLDITLGIALGSATQIALFVVPVTVLVAWTMGIEMDLNFNLLETACFALSILVTSLVLQDGTSNYMKG

LVLLLCYVVIAACFFVSNSPSTETNTTNHTITKR

>gi|30696001|ref|NP_175969.2| ATCAX5; calcium:cation antiporter/ cation:cation antiporter [Arabidopsis thaliana]

>AtCAX5_CAX

MGCCKVPALIQAQVEMGLVNDVEHKSLFRRDTDSPERKAASLMEQGSLSASFRECSTKTPNNSVLQSFKI

VILSNKLNLLLPFGPLAILLHYLTDNKGWIFLLSLVGITPLAERLGYATEQLACYTGSTVGGLLNATFGN

VTELIISIFALKSGMIRVVQLTLLGSILSNMLLVLGCAFFCGGLVFSQKEQVFDKGNAVVNSGLLLMAVM

GLLFPAVLHYTHSEVHAGSSELALSRFSSCIMLVAYAAYLFFQLKSQPSSYTPLTEETNQNEETSDDDED

PEISKWEAIIWLSILTAWVSLLSGYLVDAIEGASVSWKIPISFISVILLPIVGNAAEHAGAIMFAMKDKL

DLSLGVAIGSSIQISMFAVPFCVVIGWMMGAQMDLNFQLFETATLFITVIVVAFFLQEGTSNYFKGLMLI

LCYLIVAASFFVHEDPHQDDI

**4.2 The CCX family**

>gi|15238665|ref|NP_197288.1| CAX7 (calcium exchanger 7); calcium:sodium antiporter/ cation:cation antiporter [Arabidopsis thaliana]

>AtCAX7_CCX

MASLFSSRLGSQSLSLLINIFFIFLIFLHFASQTPPPSGSIQTLNSFAGGDSDSCSGGLASLDDHRSKCS

YIRSQSKCGPQGYIDYLKIFFCIFGQSPVLGHLVLSAWLFVLFYLLGDTAASYFCPSLDSLSKVLKLSPT

MAGVTLLSLGNGAPDLFSSVVSFTRSNNGDFGLNSILGGAFFVSSFVVGTICVLIGSRDVAIDRNSFIRD

VVFLLVALCCLGLIIFIGKVTIWVALCYLSIYLLYVGFLSVSHFFDRKKRMSDQILRSREDLAEMGVSLL

GYIAEEKLALPEKTTQEFKIVFEDSPKRHRSCFSVLVSIIGLPLYLPRRLTIPVVCEEKWSKPCAVVSTA

IAPVLLTELYCSHYSGSQRNLILYIISGSIGLIVGILAYLTTEKSHPPKKFSLVWLLGGFTMSVTWTYMI

AQELVSLLISLGNIFGISPSVLGLTVLAWGNSLGDLIANVTVAFHGGNDGAQIALSGCYAGPLFNTVIGL

GVPLVISSLAEYPGVYIIPSDNSLLETLGFLMVGLLWALVIMPKKKMRLDKLVGGGLLAIYLCFLSLRLA

RVFGVLDTDR

>gi|15238663|ref|NP_197287.1| cation exchanger, putative (CAX8) [Arabidopsis thaliana]

>AtCAX8_CCX

MGFSFSSNRFGYLTVTFLLVISCLLLGFFTNPVDSSALRPKSEHDCSALKHFHDYKSKCAYLKSIDPCAS

QGFFDYLSFLYCNFEGFPILGQFLLFLWLLLLFYLLGHTASEYFCSSLESLSKLLNLSPTVAGVTLLSLG

NGAPDLFASLVSFMGESKGTYDVGLNTVVGGSGFVTCVVVGIISISLHKRRVRIERAAFIRDICFFCAAI

GSLALILVYGKINFWGALGFCSLYAVYVAFVVLSWRFGGDQGAESDLESIHKRGSLSEPILQRDGLEEIE

DGVVNGEHQIVDDDDDHQRYYYWKRLVIWAITLPLNLPRILTIPVVSEDKWSKPLAVASVTFAPVLLSFL

WNWKRKPTSFEAGVVYLIGCLIGIALGFIAGATTKKLTPPKKWLLPWLAGGFVMSMTWSYISAQELVALL

TSLGYIFGVSPSILGLTVLAWGNSIGDLITNLTMALHDGNEGAQVAVSGCYAGPIFNTLFALGISLVGCA

WEAYPLSIVIKTDPRLLESLGFLVAGLVWSFLVLFSNRMRLGGVMGIGLLVIYLASLSLRIMQTVGDAH

>gi|18400266|ref|NP_566474.1| CAX9 (CATION EXCHANGER 9); cation transmembrane transporter/ cation:cation antiporter/ manganese ion transmembrane transporter/ potassium ion transmembrane transporter/ sodium ion transmembrane transporter [Arabidopsis thaliana]

>AtCAX9_CCX

MSAVSFLYSSKTPKFRGVFNGICALVLFCFFFDRSELLRNPLLRNASFVNGGSGSTSGGITQFMVIRRNARQIETNGSGNNSSLSSSSTVLCSGLHKHMGYADQCEFLKANPICSPDGFFDYLSFFYCSCRDFSILGYMMLGVWLVALFYLLGNTAADYFCCSLEKLSKLLRLPPTVAGVTLLPLGNGAPDVFASIAAFVGTDKGEVGLNSVLGGAVFVTSVVVGIVSLCVADKEVKIDKNCFIRDLSFFLFSLVSLLVILMVGRVTVRIAIAFVSIYVVYAFLVAANVILRKHAKRFKLEALTPLLPMQGSVFSPSVGEDMPMNTPLIETETEDGPPRLQSLPQWMWASNVAIYSNHFAKVSVHDEDRPPWGWIDDTAEVESSSCTKFTSLLEIPLTIPRRLTIPSVEEDTWSKTYAVASVSLAPVLLASLWSSQDDVSLQACGVAYFFSVVIGSTLGFLAYKNTEPDHPPRRFLIPWVLGGFIMSIVWFYMIANELVALLVTFGEIYGINPSILALTVLAWGNSMGDLVSNIALTMNGGDGVQIALSGCYAGPMFNTLVGLGMSMLFGAWSKSPDTYMLPEDKSLFYTLGFLVLGLVWAMVILPRNDMQPSRTLGVGLIAIYLIFVTFRLSCAMGFIPWAA

>gi|79339424|ref|NP_172370.3| CAX11; calcium:sodium antiporter/ cation:cation antiporter [Arabidopsis thaliana]

>AtCAX11_CCX

MELISSSTIGNSALCLTLISILIFFFLTTTTIPTFPDHPLRSLLDDSQITTNSSSIVNPKSSCVSSRSHD

NGGVINYFSLHYCIFNENLFFSIPILSLLILLHFYILIKTAQTHFSTVTTKLADRLNLSPSMAAVTLLAL

GNGAPDVFASVAALRGGQYRTGFGAILSAGTFVSAFVVGFVAIYAAPFPVDAASFVRDVLFYLIAALFLF

YVYLSGEIFVWQAIGFVGFYIFFVGFVFWMDFGTNVEKGKSISEEEKDLLRLQDCEIAAGSLGSYKAEKE

HQFSGIFRLYGTISRMWETPVSVLLNLTIPKPSPSEWSRFYRSANIVFCPFALLYTCNSFVQLNHPISFL

FPNTHLPLWLVVLFMTSSLAFLHFTVEKQPPKTEQLPVIVVAFIMSVFWISTIAGELLNCLAALGTLLKL

PPALLGLTVLAWGNSVGDLVADVAVAKAGRPAMAMAGCFAGPMFNMLVGLGSALVMQTANVYPDAYKLGF

HVGIVIAFVFLLLSLMGSLLVITWSRFRVPRFWGICLVGLYVAFTFVSLIIASVST

**4.3 The NCKX family**

>gi|21702721|gb|AAM76070.1|AF520704_1 K-dependent Na/Ca exchanger NCKX4 [Homo sapiens]

>HsaNCKX4_NCKX

MLPQQVGFVCAVLALVCCASGLFGSLGHKTASASKRVLPDTWRNRKLMAPVNGTQTAKNCTDPAIHEFPT

DLFSNKERQHGAVLLHILGALYMFYALAIVCDDFFVPSLEKICERLHLSEDVAGATFMAAGSSTPELFAS

VIGVFITHGDVGVGTIVGSAVFNILCIIGVCGLFAGQVVRLTWWAVCRDSVYYTISVIVLIVFIYDEQIV

WWEGLVLIILYVFYILIMKYNVKMQAFFTVKQKSIANGNPVNSELEAGNDFYDGSYDDPSVPLLGQVKEK

PQYGKNPVVMVDEIMSSSPPKFTFPEAGLRIMITNKFGPRTRLRMASRIIINERQRLINSANGVSSKPLQ

NGRHENIENGNVPVENPEDPQQNQEQQPPPQPPPPEPEPVEADFLSPFSVPEARGDKVKWVFTWPLIFLL

CVTIPNCSKPRWEKFFMVTFITATLWIAVFSYIMVWLVTIIGYTLGIPDVIMGITFLAAGTSVPDCMASL

IVARQGLGDMAVSNTIGSNVFDILVGLGVPWGLQTMVVNYGSTVKINSRGLVYSVVLLLGSVALTVLGIH

LNKWRLDRKLGVYVLVLYAIFLCFSIMIEFNVFTFVNLPMCREDD

>gi|14717396|gb|AAG12988.2|AF169257_1 potassium-dependent Na/Ca exchanger NCKX3 [Homo sapiens]

>HsaNCKX3_NCKX

RDLLLSQLCFLASVALLLWSLSSLREQKELDLMDLVGEDRKWMMARKLMQVNDTLTSEDAGLRNSKNCTE

PALHEFPNDIFTNEDRRQGAVVLHVLCAIYMFYALAIVCDDFFVPSLEKICERLHLSEDVAGATFMAAGS

SAPELFTSVIGVFITKGDVGVGTIVGSAVFNILCIIGVCGLFAGQVVALSSWCLLRDSIYYTLSVIALIV

FIYDEKVSWWESLVLVLMYLIYIVIMKYNACIHQCFERRTKGAGNMVNGLANNAEIDDSSNCDATVVLLK

KANFHRKASVIMVDELLSAYPHQLSFSEAGLRIMITSHFPPKTRLSMASRMLINERQRLINSRAYTNGES

EVAIKIPIKHTVENGTGPSSAPDRGVNGTRRDDVVAEAGNETENENEDNENDEEEEEDEDDDEGPYTPFD

TPSGKLETVKWAFTWPLSFVLYFTVPNCNKPRWEKWFMVTFASSTLWIAAFSYMMVWMVTIIGYTLGIPD

VIMGITFLAAGTSVPDCMASLIVARQGMGDMAVSNSIGSNVFDILIGLGLPWALQTLAVDYGSYIRLNSR

GLIYSVGLLLASVFVTVFGVHLNKWQLDKKLGCGCLLLYGVFLCFSIMTEFNVFTFVNLPMCGDH

**4.4 The YRBG family**

>gi|26249782|ref|NP_755822.1| putative calcium/sodium:proton antiporter [Escherichia coli CFT073]

>E.coli_YRBG

MLLATALLIVGLLLVVYSADRLVFAASILCRTFGIPPLIIGMTVVSIGTSLPEIIVSLAASLHEQRDLAV

GTALGSNIINILLILGLAALVRPFTVHSDVLRRELPLMLLVSVVAGSVLYDGQLSRSDGIFLLFLAVLWL

LFIVKLARQAERQGTDSLTREQLAELPREGGLPVAFLWLGIALIIMPVATRMVVDNATVLANYFAISELT

MGLTAIAIGTSLPELATAIAGVRKGENDIAVGNIIGANIFNIVIVLGLPALITPGEIDPLAYSRDYSVML

LVSIIFALLCWRRSPQPGRGVGVLLTGGFIVWLAMLYWLSPILVE

>gi|32034202|ref|ZP_00134424.1| COG0530: Ca2+/Na+ antiporter [Actinobacillus pleuropneumoniae serovar 1 str. 4074]

>A.pleuro_YRBG

MMLYASLAIVIGLILLVWSADKFVEGAASSARHFGMSPLLIGIVIVGFGTSAPEMLVSASSALKGASGIA

LGNAYGSNITNIALILGVTALIKPIIVASEVIKKELPMLILITLVSAYMLFDAQVTQIEAVILLIIFAVY

MGWTIWVALRSKNDTLAQTESLNEERLPIGKALFWVVIGLLFLMASSQLLVWGAVEIAKYFGVSDLVIGL

TIVAVGTSLPELASSIIAARKGESDLALGNIVGSNLFNTLAVVGIAGAIEPMQVEAEVFSRDMLVMLAVT

VLLFFFAFSFRKRAGRINRAEGAILFICYVAYTLYLLKTAV

>gi|33862742|ref|NP_894302.1| CaCA family, sodium/calcium exchanger [Prochlorococcus marinus str. MIT 9313]

>P.marinus_YRBG

MPQFLEPALQVLAGIGLLFGGGELFVQGSVALALILGIPELVIGLTVVALGTCAPELFVSVSSVLKGSDA

LAVSNVVGSNIFNVMVVLGSSALVLPLRVESRLVRRDVPLLLAVSAAVWGMASAGRVTWQAGVALLIALV

INSVWEIRTAREEPDGMVEAEPEINSKTANGGWIKACIRLLLGILLLTVGSNQLVKGASDAASLLGVSET

VIGLTIVSAGTSMPELFTSLVAALRGRTDLAIGNVVGSSLLNQLLVLSSCALVSGGAGLQVDRLIIERDL

PVMVLTTLACMPIFWTRGRINRLEGGILVGLYVFYVIDQVLPRTLPTWQDGFRLTMLCIVLPIILVMIIL

QALLYWRQLRKERITQSVD

Note: The M. jannaschii Na/Ca exchanger was added to this section although it was not used as a marker protein. See text for more details.

>gi|15668262|ref|NP_247055.1| Na+/Ca+ exchanger protein [Methanocaldococcus jannaschii DSM 2661]

>M.jannaschii

MLILGVGYFLLGLILLYYGSDWFVLGSERIARHFNVSNFVIGATVMAIGTSLPEILTSAYASYMHAPGISIGNAIGSCICNIGLVLGLSAIISPIIVDKNLQKNILVYLLFVIFAAVIGIDGFSWIDGVVLLILFIIYLRWTVKNGSAEIEENNDKNNPSVVFSLVLLIIGLIGVLVGAELFVDGAKKIALALDISDKVIGFTLVAFGTSLPELMVSLAAAKRNLGGMVLGNVIGSNIADIGGALAVGSLFMHLPAENVQMAVLVIMSLLLYLFAKYSKIGRWQGILFLALYIIAIASLRMG

**4.5 The NCX family**

>gi|10863913|ref|NP_066920.1| sodium/calcium exchanger 1 isoform A [Homo sapiens]

>H.sapiens_NCX1.1

MYNMRRLSLSPTFSMGFHLLVTVSLLFSHVDHVIAETEMEGEGNETGECTGSYYCKKGVILPIWEPQDPSFGDKIARATVYFVAMVYMFLGVSIIADRFMSSIEVITSQEKEITIKKPNGETTKTTVRIWNETVSNLTLMALGSSAPEILLSVIEVCGHNFTAGDLGPSTIVGSAAFNMFIIIALCVYVVPDGETRKIKHLRVFFVTAAWSIFAYTWLYIILSVISPGVVEVWEGLLTFFFFPICVVFAWVADRRLLFYKYVYKRYRAGKQRGMIIEHEGDRPSSKTEIEMDGKVVNSHVENFLDGALVLEVDERDQDDEEARREMARILKELKQKHPDKEIEQLIELANYQVLSQQQKSRAFYRIQATRLMTGAGNILKRHAADQARKAVSMHEVNTEVTENDPVSKIFFEQGTYQCLENCGTVALTIIRRGGDLTNTVFVDFRTEDGTANAGSDYEFTEGTVVFKPGDTQKEIRVGIIDDDIFEEDENFLVHLSNVKVSSEASEDGILEANHVSTLACLGSPSTATVTIFDDDHAGIFTFEEPVTHVSESIGIMEVKVLRTSGARGNVIVPYKTIEGTARGGGEDFEDTCGELEFQNDEIVKTISVKVIDDEEYEKNKTFFLEIGEPRLVEMSEKKALLLNELGGFTITGKYLFGQPVFRKVHAREHPILSTVITIADEYDDKQPLTSKEEEERRIAEMGRPILGEHTKLEVIIEESYEFKSTVDKLIKKTNLALVVGTNSWREQFIEAITVSAGEDDDDDECGEEKLPSCFDYVMHFLTVFWKVLFAFVPPTEYWNGWACFIVSILMIGLLTAFIGDLASHFGCTIGLKDSVTAVVFVALGTSVPDTFASKVAATQDQYADASIGNVTGSNAVNVFLGIGVAWSIAAIYHAANGEQFKVSPGTLAFSVTLFTIFAFINVGVLLYRRRPEIGGELGGPRTAKLLTSCLFVLLWLLYIFFSSLEAYCHIKGF

>gi|114577107|ref|XP_001137264.1| PREDICTED: solute carrier family 8 (sodium/calcium exchanger), member 1 isoform 11 [Pan troglodytes]

>P.troglodytes_NCX1.1

MYNMRRLSLSPTFSMGFHLLVIVTLLFSHVDHVIAETEMEGEGNETGECTGSYYCKKGVILPIWEPQDPS

FGDKIARATVYFVAMVYMFLGVSIIADRFMSSIEVITSQEKEITIKKPNGETTKTTVRIWNETVSNLTLM

ALGSSAPEILLSVIEVCGHNFTAGDLGPSTIVGSAAFNMFIIIALCVYVVPDGETRKIKHLRVFFVTAAW

SIFAYTWLYIILSVISPGVVEVWEGLLTFFFFPICVVFAWVADRRLLFYKYVYKRYRAGKQRGMIIEHEG

DRPSSKTEIEMDGKVVNSHVENFLDGALVLEVDERDQDDEEARREMARILKELKQKHPDKEIEQLIELAN

YQVLSQQQKSRAFYRIQATRLMTGAGNILKRHAADQARKAVSMHEVNTEVTENDPVSKIFFEQGTYQCLE

NCGTVALTIIRRGGDLTNTVFVDFRTEDGTANAGSDYEFTEGTVVFKPGETQKEIRVGIIDDDIFEEDEN

FLVHLSNVKVSSEASEDGILEANHVSTLACLGSPSTATVTIFDDDHAGIFTFEEPVTHVSESIGIMEVKV

LRTSGARGNVIVPYKTIEGTARGGGEDFEDTCGELEFQNDEIVKTISVKVIDDEEYEKNKTFFLEIGEPR

LVEMSEKKALLLNELGGFTITGKYLFGQPVFRKVHAREHPILSTVITIADEYDDKQPLTSKEEEERRIAE

MGRPILGEHTKLEVIIEESYEFKSTVDKLIKKTNLALVVGTNSWREQFIEAITVSAGEDDDDDECGEEKL

PSCFDYVMHFLTVFWKVLFAFVPPTEYWNGWACFIVSILMIGLLTAFIGDLASHFGCTIGLKDSVTAVVF

VALGTSVPDTFASKVAATQDQYADASIGNVTGSNAVNVFLGIGVAWSIAAIYHAANGEQFKVSPGTLAFS

VTLFTIFAFINVGVLLYRRRPEIGGELGGPRTAKLLTSCLFVLLWLLYIFFSSLEAYCHIKGF

>gi|74136283|ref|NP_001028033.1| sodium/calcium exchanger 1 [Macaca mulatta]

>M.mulatta_NCX1.3

MRRLSLSPTFSMGFHLLVIVALLFSHVDYVIAETEMEGEGNETGECTGSYYCKKGVILPIWEPQDPSFGD

KIARATVYFVAMVYMFLGVSIIADRFMSSIEVITSQEKEITIKKPNGETTKTTVRIWNETVSNLTLMALG

SSAPEILLSVIEVCGHNFTAGDLGPSTIVGSAAFNMFIIIALCVYVVPDGETRKIKHLRVFFVTAAWSIF

AYTWLYIILSVISPGVVEVWEGLLTFFFFPICVVFAWVADRRLLFYKYVYKRYRAGKQRGMIIEHEGDRP

SSKTEIEMDGKVVNSHVENFLDGALVLEVDERDQDDEEARREMARILKELKQKHPEKEIEQLIELANYQV

LSQQQKSRAFYRIQATRLMTGAGNILKRHAADQARKAVSMHEVNTEVTENDPVSKIFFEQGTYQCLENCG

TVALTIIRRGGDLTNTVFVDFRTEDGTANAGSDYEFTEGTVVFKPGETQKEIRVGIIDDDIFEEDENFLV

HLSNVKVSSEASEDGILEANHVSTLACLGSPSTATVTIFDDDHAGIFTFEEPVTHVSESIGIMEVKVLRT

SGARGNVIVPYKTIEGTARGGGEDFEDTCGELEFQNDEIVKIITIRIFDREEYEKECSFSLVLEEPKWIR

RGMKGGFTITDEYDDKQPLTSKEEEERRIAEMGRPILGEHTKLEVIIEESYEFKSTVDKLIKKTNLALVV

GTNSWREQFIEAITVSAGEDDDDDECGEEKLPSCFDYVMHFLTVFWKVLFAFVPPTEYWNGWACFIVSIL

MIGLLTAFIGDLASHFGCTIGLKDSVTAVVFVALGTSVPDTFASKVAATQDQYADASIGNVTGSNAVNVF

LGIGVAWSIAAIYHAANGEQFKVSPGTLAFSVTLFTIFAFINVGVLLYRRRPEIGGELGGPRTAKLLTSC

LFVLLWLLYIFFSSLEAYCHIKGF

>gi|127793|sp|P23685.1|NAC1_CANFA RecName: Full=Sodium/calcium exchanger 1; AltName: Full=Na(+)/Ca(2+)-exchange protein 1; Flags: Precursor

>C.familiaris_NCX1.1

MLQLRLLPTFSMGCHLLAVVALLFSHVDLISAETEMEGEGNETGECTGSYYCKKGVILPIWEPQDPSFGD

KIARATVYFVAMVYMFLGVSIIADRFMSSIEVITSQEKEITIKKPNGETTKTTVRIWNETVSNLTLMALG

SSAPEILLSVIEVCGHNFTAGDLGPSTIVGSAAFNMFIIIALCVYVVPDGETRKIKHLRVFFVTAAWSIF

AYTWLYIILSVISPGVVEVWEGLLTFFFFPICVVFAWVADRRLLFYKYVYKRYRAGKQRGMIIEHEGDRP

SSKTEIEMDGKVVNSHVDNFLDGALVLEVDERDQDDEEARREMARILKELKQKHPEKEIEQLIELANYQV

LSQQQKSRAFYRIQATRLMTGAGNILKRHAADQARKAVSMHEVNTEVAENDPVSKIFFEQGTYQCLENCG

TVALTIIRRGGDLTNTVFVDFRTEDGTANAGSDYEFTEGTVVFKPGETQKEIRVGIIDDDIFEEDENFLV

HLSNVKVSSEASEDGILEANHVSALACLGSPSTATVTIFDDDHAGIFTFEEPVTHVSESIGIMEVKVLRT

SGARGNVIVPYKTIEGTARGGGEDFEDTCGELEFQNDEIVKTISVKVIDDEEYEKNKTFFLEIGEPRLVE

MSEKKALLLNELGGFTITGKYLYGQPVFRKVHAREHPIPSTVITIAEEYDDKQPLTSKEEEERRIAEMGR

PILGEHTKLEVIIEESYEFKSTVDKLIKKTNLALVVGTNSWREQFIEAITVSAGEDDDDDECGEEKLPSC

FDYVMHFLTVFWKVLFAFVPPTEYWNGWACFIVSILMIGILTAFIGDLASHFGCTIGLKDSVTAVVFVAL

GTSVPDTFASKVAATQDQYADASIGNVTGSNAVNVFLGIGVAWSIAAIYHAANGEQFKVSPGTLAFSVTL

FTIFAFINVGVLLYRRRPEIGGELGGPRTAKLLTSCLFVLLWLLYIFFSSLEAYCHIKGF

>gi|57619018|ref|NP_001009848.1| sodium/calcium exchanger 1 precursor [Felis catus]

>F.catus_NCX1.1

MLRLRLSPTFSVGFHLLAFVPLLFSHVDLISADTEMEGEGNETGECTGSYYCKKGVILPIWEPQDPSFGD

KIARATVYFVAMVYMFLGVSIIADRFMSSIEVITSQEKEITIKKPNGETTKTTVRIWNETVSNLTLMALG

SSAPEILLSVIEVCGHNFTAGDLGPSTIVGSAAFNMFIIIALCVYVVPDGETRKIKHLRVFFVTAAWSIF

AYTWLYIILSVISPGVVEVWEGLLTFFFFPICVVFAWVADRRLLFYKYVYKRYRAGKQRGMIIEHEGDRP

SSKTEIEMDGKVVNSHVDNFLDGALVLEVDERDQDDEEARREMARILKELKQKHPEKEIEQLIELANYQV

LSQQQKSRAFYRIQATRLMTGAGNILKRHAADQARKAVSMHEVNTEVAENDPVSKIFFEQGTYQCLENCG

TVALTILRRGGDLTNTVFVDFRTEDGTANAGSDYEFTEGTVVFKPGETQKEIRVGIIDDDIFEEDENFLV

HLSNVKVSSEASEDGILEANHVSTLACLGSPSTATVTIFDDDHAGIFTFEEPVTHVSESIGIMEVKVLRT

SGARGNVIVPYKTIEGTARGGGEDFEDTCGELEFQNDEIVKTISVKVIDDEEYEKNKTFFLEIGEPRLVE

MSEKKALLLNELGGFTITGKYLYGQPVFRKVHAREHPIPSTVITIAEECDAKQPLTSKEEEERRIAEMGR

PILGEHTKLEVIIEESYEFKSTVDKLIKKTNLALVVGTNSWREQFIEAITVSAGEDDDDDECGEEKLPSC

FDYVMHFLTVFWKVLFAFVPPTEYWNGWACFIVSILMIGILTAFIGDLASHFGCTIGLKDSVTAVVFVAL

GTSVPDTFASKVAATQDQYADASIGNVTGSNAVNVFLGIGVAWSIAAIYHAANGEQFKVSPGTLAFSVTL

FTIFAFINVGVLLYRRRPEIGGELGGPRTAKLLTSCLFVLLWLLYIFFSSLEAYCHIKGF

>gi|28603740|ref|NP_788805.1| sodium/calcium exchanger 1 precursor [Bos taurus]

>B.taurus_NCX1.1

MLQFSLSPTLSMGFHVIAMVALLFSHVDHISAETEMEGEGNETGECTGSYYCKKGVILPIWEPQDPSFGD

KIARATVYFVAMVYMFLGVSIIADRFMSSIEVITSQEKEITIKKPNGETTKTTVRIWNETVSNLTLMALG

SSAPEILLSVIEVCGHNFTAGDLGPSTIVGSAAFNMFIIIALCVYVVPDGETRKIKHLRVFFVTAAWSIF

AYTWLYIILSVSSPGVVEVWEGLLTFFFFPICVVFAWVADRRLLFYKYVYKRYRAGKQRGMIIEHEGDRP

SSKTEIEMDGKVVNSHVDSFLDGALVLEVDERDQDDEEARREMARILKELKQKHPEKEIEQLIELANYQV

LSQQQKSRAFYRIQATRLMTGAGNILKRHAADQARKAVSMHEVNTEVAENDPVSKIFFEQGTYQCLENCG

TVALTIIRRGGDLTNTVFVDFRTEDGTANAGSDYEFTEGTVVFKPGETQKEIRVGIIDDDIFEEDENFLV

HLSNVKVSLEASEDGILEASHVSTLACLGSPSTATVTIFDDDHAGIFTFEEPVTHVSESIGIMEVKVLRT

SGARGNVIVPYKTIEGTARGGGEDFEDTCGELEFQNDEIVKTISVKVIDDEEYEKNKTFFLEIGEPRLVE

MSEKKALLLNELGGFTITGKYLYGQPVFRKVHAREHPLPSTIITIADEYDDKQPLTSKEEEERRIAEMGR

PILGEHTRLEVIIEESYEFKSTVDKLIKKTNLALVVGTNSWREQFIEAITVSAGEDDDDDECGEEKLPSC

FDYVMHFLTVFWKVLFAFVPPTEYWNGWACFIVSILMIGLLTAFIGDLASHFACTIALKDSVTAVVFVAL

GTSVPDTFASKVAATQDQYADASIGNVTGSNAVNVFLGIGVAWSIAAIYHAANGEQFKVSPGTLAFSVTL

FTIFAFINVGVLLYRRRPEIGGELGGPRTAKLLTSCLFVLLWLLYIFFSSLEAYCHIKGF

>gi|283436126|ref|NP_001164429.1| sodium/calcium exchanger 1 [Oryctolagus cuniculus]

>O.cuniculus_NCX1.2

MPRFSLSPPFSMGFHLLAIVALFFFRVDHVSAETEMEGEGNETGECTGSYYCKKGVILPIWEPQDPSFGD

KIARATVYFVAMVYMFLGVSIIADRFMSSIEVITSQEKEITIKKPNGETTKTTVRIWNETVSNLTLMALG

SSAPEILLSVIEVCGHNFTAGDLGPSTIVGSAAFNMFIIIALCVYVVPDGETRKIKHLRVFFVTAAWSIF

AYTWLYIILSVISPGIVEVWEGLLTFFFFPICVVFAWVADRRLLFYKYVYKRYRAGKQRGMIIEHEGDRP

SSKTEIEMDGKVVNSHVDNFLDGALVLDVDERDQDDEEARREMARILKELKQKHPEKEIEQLIELANYQV

LSQQQKSRAFYRIQATRLMTGAGNILKRHAADQARKAVSMHEVNTEMAENDPVSKIFFEQGTYQCLENCG

TVALTIIRRGGDLTNTVFVDFRTEDGTANAGSDYEFTEGTVVFKPGETQKEIRVGIIDDDIFEEDENFLV

HLSNVKVSSETSEDGILEANHISTLACLGSPCTATVTIFDDDHAGIFTFEESVTHVSESIGIMEVKVLRT

SGARGNVIVPYKTIEGTARGGGEDFEDTCGELEFQNDEIVKIITIRIFDREEYEKECSLSLVLEEPKWIR

RGMKALLLNELGGFTITEEYDDKQPLTSKEEEERRIAEMGRPILGEHTKLEVIIEESYEFKSTVDKLIKK

TNLALVVGTNSWREQFIEAITVSAGEDDDDDECGEEKLPSCFDYVMHFLTVFWKVLFAFVPPTEYWNGWA

CFIVSILMIGLLTAFIGDLASHFGCTIGLKDSVTAVVFVALGTSVPDTFASKVAATQDQYADASIGNVTG

SNAVNVFLGIGVAWSIAAIYHAANGEHFKVSPGTLAFSVTLFTIFAFINVGVLLYRRRPEIGGELGGPRT

AKLLTSCLFVLLWLLYIFFSSLEAYCHIKGF

>gi|290491228|ref|NP_001166490.1| sodium/calcium exchanger 1 [Cavia porcellus]

>C.porcellus_NCX1.1

MLRLSLSPTYSLGFHLLAMMTLLISHVDHITAETEMVEEGNETGECTGSYYCKKGVILPIWEPQDPSFGD

KIARATVYFVAMVYMFLGVSIIADRFMSSIEVITSQEKEITIKKPNGETTKTTVRIWNETVSNLTLMALG

SSAPEILLSVIEVCGHNFTAGDLGPSTIVGSAAFNMFIIIALCVYVVPDGETRKIKHLRVFFVTAAWSIF

AYTWLYIILSVISPGVVEVWEGLLTFFFFPICVVFAWVADRRLLFYKYVYKRYRAGKQRGMIIEHEGDRP

SSKTEIEMDGKVVNSHVENFLDGALVLEVDERDQDDEEARREMARILKELKQKHPEKEIEQLIELANYQV

LSQQQKSRAFYRIQATRLMTGAGNILKRHAADQARKAVSMHEVNTEVAENDPVSKIFFEQGTYQCLENCG

TVALTIIRRGGDLTNTVFVDFRTEDGTANAGSDYEFTEGTVVFKPGETQKEIRVGIIDDDIFEEDENFLV

HLSNVKVSSEASEDGILEANHISTLACLGSPSTATVTIFDDDHAGIFTFEEPVTHVSESIGIMEVKVLRT

SGARGNVIVPYKTIEGTARGGGEDFEDTCGELEFQNDEIVKTISVKVIDDEEYEKNKTFFLEIGEPRLVE

MSEKKALLLNELGGFTITGKHLYGQPVLRKVHARDHPIPSTVITIADEYDDKQPLTSKEEEERRIAELGR

PILGEHTKLEVIIEESYEFKSTVDKLIKKTNLALVVGTNSWREQFIEAITVSAGEDDDDDECGEEKLPSC

FDYVMHFLTVFWKVLFAFVPPTEYWNGWACFIVSILMIGLLTAFIGDLASHFGCTIGLKDSVTAVVFVAL

GTSVPDTFASKVAATQDQYADASIGNVTGSNAVNVFLGIGVAWSIAAIYHAANGEQFKVSPGTLAFSVTL

FTIFAFINVGVLLYRRRPEIGGELGGPRTAKLLTSCLFVLLWLLYIFFSSLEAYCHIKGF

>gi|78214331|ref|NP_062141.2| sodium/calcium exchanger 1 precursor [Rattus norvegicus]

>R.norvegicus_NCX1.1

MLRLSLPPNVSMGFRLVTLVALLFTHVDHITADTEAETGGNETTECTGSYYCKKGVILPIWEPQDPSFGD

KIARATVYFVAMVYMFLGVSIIADRFMSSIEVITSQEKEITIKKPNGETTKTTVRIWNETVSNLTLMALG

SSAPEILLSVIEVCGHNFTAGDLGPSTIVGSAAFNMFIIIALCVYVVPDGETRKIKHLRVFFVTAAWSIF

AYTWLYIILSVSSPGVVEVWEGLLTFFFFPICVVFAWVADRRLLFYKYVYKRYRAGKQRGMIIEHEGDRP

ASKTEIEMDGKVVNSHVDNFLDGALVLEVDERDQDDEEARREMARILKELKQKHPDKEIEQLIELANYQV

LSQQQKSRAFYRIQATRLMTGAGNILKRHAADQARKAVSMHEVNMDVVENDPVSKVFFEQGTYQCLENCG

TVALTIIRRGGDLTNTVFVDFRTEDGTANAGSDYEFTEGTVIFKPGETQKEIRVGIIDDDIFEEDENFLV

HLSNVRVSSEVSEDGILDSNHVSAIACLGSPNTATITIFDDDHAGIFTFEEPVTHVSESIGIMEVKVLRT

SGARGNVIIPYKTIEGTARGGGEDFEDTCGELEFQNDEIVKTISVKVIDDEEYEKNKTFFIEIGEPRLVE

MSEKKALLLNELGGFTLTEGKKMYGQPVFRKVHARDHPIPSTVISISEEYDDKQPLTSKEEEERRIAEMG

RPILGEHTKLEVIIEESYEFKSTVDKLIKKTNLALVVGTNSWREQFIEAITVSAGEDDDDDECGEEKLPS

CFDYVMHFLTVFWKVLFAFVPPTEYWNGWACFIVSILMIGLLTAFIGDLASHFGCTIGLKDSVTAVVFVA

LGTSVPDTFASKVAATQDQYADASIGNVTGSNAVNVFLGIGVAWSIAAIYHAANGEQFKVSPGTLAFSVT

LFTIFAFINVGVLLYRRRPEIGGELGGPRTAKLLTSSLFVLLWLLYIFFSSLEAYCHIKGF

>gi|2829482|sp|P70414.1|NAC1_MOUSE RecName: Full=Sodium/calcium exchanger 1; AltName: Full=Na(+)/Ca(2+)-exchange protein 1; Flags: Precursor

>M.musculus_NCX1.1

MLRLSLPPNVSMGFRLVALVALLFSHVDHITADTEAETGGNETTECTGSYYCKKGVILPIWEPQDPSFGD

KIARATVYFVAMVYMFLGVSIIADRFMSSIEVITSQEKEITIKKPNGETTKTTVRIWNETVSNLTLMALG

SSAPEILLSVIEVCGHNFTAGDLGPSTIVGSAAFNMFIIIALCVYVVPDGETRKIKHLRVFFVTAAWSIF

AYTWLYIILSVSSPGVVEVWEGLLTFFFFPICVVFAWVADRRLLFYKYVYKRYRAGKQRGMIIEHEGDRP

ASKTEIEMDGKVVNSHVDNFLDGALVLEVDERDQDDEEARREMARILKELKQKHPEKEIEQLIELANYQV

LSQQQKSRAFYRIQATRLMTGAGNILKRHAADQARKAVSMHEVNMEMAENDPVSKIFFEQGTYQCLENCG

TVALTIMRRGGDLSTTVFVDFRTEDGTANAASDYEFTEGTVIFKPGETQKEIRVGIIDDDIFEEDENFLV

HLSNVRVSSDVSEDGILESNHASSIACLGSPSTATITIFDDDHAGIFTFEEPVTHVSESIGIMEVKVLRT

SGARGNVIIPYKTIEGTARGGGEDFEDTCGEPEFQNDEIVKTISVKVIDDEEYEKNKTFFIEIGEPRLVE

MSEKKALLLNELGGFTLTGKEMYGQPIFRKVHARDHPIPSTVITISEEYDDKQPLTSKEEEERRIAEMGR

PILGEHTKLEVIIQESYEFKSTVDKLIKKTNLALVVGTNSWREQFIEAITVSAGEDDDDDECGEEKLPSC

FDYVMHFLTVFWKVLFAFVPPTEYWNGWACFIVSILMIGLLTAFIGDLASHFGCTIGLKDSVTAVVFVAL

GTSVPDTFASKVAATQDQYADASIGNVTGSNAVNVFLGIGVAWSIAAIYHAANGEQFKVSPGTLAFSVTL

FTIFAFINVGVLLYRRRPEIGGELGGPRTAKLLTSSLFVLLWLLYIFFSSLEAYCHIKGF

>gi|301608730|ref|XP_002933933.1| PREDICTED: sodium/calcium exchanger 1-like [Xenopus (Silurana) tropicalis]

>S.tropicalis_NCX1

MMKLGTSLAFFARFHFLVLLLLLCNVGSIRSETTTVADFENHTDSCTGSYYCKEGVILPIWEPQNPSFGD

KIARATVYFVAMVYMFLGVSIIADRFMSSIEVITSQEKEITIKKPNGETTKTTVRIWNETVSNLTLMALG

SSAPEILLSVIEVCGHNFQAGDLGPSTIVGSAAFNMFIIIALCVYVVPDGEIRKIKHLRVFFVTAAWSIF

AYTWLYMILSVFSPGVVEVWEGLLTFFFFPICVVFAWVADRRLLFYKYVYKRYRAGKQRGMIIETEGDRP

SSKADIEMDGKVLNSHTETFLDGSLVLEVDEKDQDEEEARRDMAKILKELKQKHPEKEIEQLIELANYQV

LSQQQKSRAFYRIQATRLMTGAGNILKRHAADQARKAVSMHEVNTDVVENDPVSKIYFEQATYQCLENCG

TVALTIVRRGGDLTNTVYVDFRTEDGSANAGSDYEFTEGTIIFKPGETQKELRVGIIDDDIFEEDENFLV

HLSNVRVNAENTEANLESNHVTPLACLGATCTATVTIFDDDHAGIFTFEETVTHVSESVGIMEVKVLRTS

GARGTVIVPYKTVEGTAKGGGEDFEDTCGQLEFQNDEIVKIISVKIIDDEEYEKNKTFFLEVGEPRLVEM

SEKKALLLNELGDFTITGKILYGKPVLRKVQVRDHPIPSTVIILTEENEEKQPLTSKEEEERRIAEMGRP

VLGEHTRLEIIIEESYEFKSTVDKLIKKTNLALVVGTNSWREQFIEAITVSAGEDDDDDECGEEKLPSCF

DYVMHFLTVFWKVLFAFVPPTEYWNGWACFIVSISMIGLLTAFIGDLASHFGCTIGLKDSVTAVVFVALG

TSVPDTFASKVAATQDQYADASIGNVTGSNAVNVFLGIGVAWSIAAIYHAANGDVFRVQPGTLAFSVTLF

TIFAFINVGVLLYRRRPEIGGELGGPRTAKLLTTALFTLLWLLYIFFSSLEAYCHIKGF

>gi|62637560|gb|AAP37041.2| sodium calcium exchanger [Oreochromis mossambicus]

>O.mossambicus_NCX1.1

MSPVRTVPMFFTYKLIFFATVISTEFPYSAAAGSTLTLTSNQTATNHSKCGGSTDCIEGVILPLWKPENP

AFTDRLARATIYFVGLVYMFLGVSIIADRFMASIEVITSQERRITIKKPNGEKITTTVRIWNETVSNLTL

MALGSSAPEILLSVVEVCGHNFNAGELGPNTIVGSAAFNMFVIIGLCVSVIPEGETRKVKHLRVFFITAA

WSVFAYTWLYLILAVFSPGVVEIWEGLLTLFFFPLCVGLAYIADRRLLFYKYMYKRYRAGKRKGVIIETE

GEPELPSKVDIEMDGKMLNSHGEEFTEELEGKELDEEEARREVARILKELKQKHPEKEMEQLMELANYQV

LTQQQKSRAFYRCQATRIMTGAGNVLKKHACDQAKRATHDIFSEVSVNDFSSKVFFDPGTYQCLENCGSV

ALNVVRRGGDLTSTVSVDYRTEDGTANASSDYEFTEGTVVFKPGETEKEIRINIIDDDIFEEDEHFLVHL

SNVRVRAEGSNSHEANHVDTLAGLGLPCTATVTIFDDDHAGIFTFEEPVMTVSESVGVMEVKVIRTSGAR

GVVVVPYKTIQGTAKGGGEDFEDTHGVLEFENDEISKTIRINIIDDEEYEKNKNFFLEIGEPRLLEMSER

KAVLLQEVGGFVKTGRDIYRKVQGREHPVPSNIISIAEDGAEEPLTKKEEEERRIAEMGRPMLGEHVKLE

VIIEESYEFKSTVDKLIKKTNLALLIGTNSWREQFVEAITVSSGDDDECREEKLPSCFDYVMHFLTIFWK

LLFAFVPPTDYWNGWACFVVSITVIGMLTAVIGDLASHFGCTVGLKDSVTAVVFVALGTSVPDTFASKVA

AIQDQYADASIGNVTGSNAVNVFLGIGVAWSIAAVYHYTQGQEFKVNPGTLAFSVTLFTIFAFICIAVLI

YRRRPEIGGELGGPRIPKILTTCLFFSLWLMYIVFSSLEAYCHVKGF

>gi|185132198|ref|NP_001118070.1| cardiac sodium-calcium exchanger [Oncorhynchus mykiss]

>O.mykiss_NCX1

MRRTGTSSFLFCALQLTVLLAVFSSEIKFVTAGNSNPSLGTNSSIGNQTNKKCDSVDTECKVGVILPIWL

PENTSFGDKLARATVYFVALFYMFLGVSIIADRFMASIEVITSQEREITIKKPNGEKVTTTVRIWNETVS

NLTLMALGSSAPEILLSVVEVCGHNFDAGDLGPNTIVGSAAFNMFVIIGFCVSVIPDGEHRKVKHLRVFF

VTATWSIFAYTWLYLILAVISPGIVQVWEGLVTLFFFPLCVGMAYVADRRLLVYKYMYKRYRAGKRRGVI

IETEGEAQIPSKMDIEMDGKMLNSESFMDGAMGFDEKDLDEEEARREMVRILKELKQKHPEKETEQLIEL

ANYQVLTQQQKSRAFYRCQATRIMTGAGNVLKKHAADQARKAVGAYEIRSEVSENDFSSKVFFDPGTYQC

LENCGTVALNVVRLGGDLTNTVSVEYRTEDGTANAGSDYQFTEGVVVFNPGETEKEIRIDIIDDDIFEED

EHFLVHLSNVKVISEGTGYVQPRANHLDTLAGLGLPCSATVTIFDDDHAGIFTFEEPVMTISESIGMMEV

KVLRTSGARGLVVVPYKTMEGTAKGGGEDFEDTHGALEFQNDEIFKSIQINIIDDEEYEKNKNFFLEMGE

PQLLEMSERKAVLLQEIGGFVKTGRDVYRKVQGRDNPVPATIISLAEEGDEEALSKKEEEERRIAEMGRP

TLGEHVKLEVVIEESYEFKNTVDKLIKKTNLALLIGTNSWRQQFMEAITVSSGDDDEDECGEEKLPSCFD

YVMHFLTVFWKLLFAFVPPTDYWNGWACFVVSISMIGLLTAFIGDLASHFGCTVGLKDSVTAVVFVALGT

SVPDTFASKVAAIQDQYADAFIGNVTGSNAVNVFLGIGVAWSIAAIYHNSKGNDFRVDPGTLAFSVTLFT

IFAFVAVAVLMYRRRPEIGGELGGPRGPKIATTCLFFSLWLMYIVFSSLEAYCHVKGF

>gi|57163987|ref|NP_055878.1| sodium/calcium exchanger 2 precursor [Homo sapiens]

>H.sapiens_NCX2.1

MAPLALVGVTLLLAAPPCSGAATPTPSLPPPPANDSDTSTGGCQGSYRCQPGVLLPVWEPDDPSLGDKAA

RAVVYFVAMVYMFLGVSIIADRFMAAIEVITSKEKEITITKANGETSVGTVRIWNETVSNLTLMALGSSA

PEILLSVIEVCGHNFQAGELGPGTIVGSAAFNMFVVIAVCIYVIPAGESRKIKHLRVFFVTASWSIFAYV

WLYLILAVFSPGVVQVWEALLTLVFFPVCVVFAWMADKRLLFYKYVYKRYRTDPRSGIIIGAEGDPPKSI

ELDGTFVGAEAPGELGGLGPGPAEARELDASRREVIQILKDLKQKHPDKDLEQLVGIANYYALLHQQKSR

AFYRIQATRLMTGAGNVLRRHAADASRRAAPAEGAGEDEDDGASRIFFEPSLYHCLENCGSVLLSVTCQG

GEGNSTFYVDYRTEDGSAKAGSDYEYSEGTLVFKPGETQKELRIGIIDDDIFEEDEHFFVRLLNLRVGDA

QGMFEPDGGGRPKGRLVAPLLATVTILDDDHAGIFSFQDRLLHVSECMGTVDVRVVRSSGARGTVRLPYR

TVDGTARGGGVHYEDACGELEFGDDETMKTLQVKIVDDEEYEKKDNFFIELGQPQWLKRGISALLLNQGD

GDRKLTAEEEEARRIAEMGKPVLGENCRLEVIIEESYDFKNTVDKLIKKTNLALVIGTHSWREQFLEAIT

VSAGDEEEEEDGSREERLPSCFDYVMHFLTVFWKVLFACVPPTEYCHGWACFGVSILVIGLLTALIGDLA

SHFGCTVGLKDSVNAVVFVALGTSIPDTFASKVAALQDQCADASIGNVTGSNAVNVFLGLGVAWSVAAVY

WAVQGRPFEVRTGTLAFSVTLFTVFAFVGIAVLLYRRRPHIGGELGGPRGPKLATTALFLGLWLLYILFA

SLEAYCHIRGF

>gi|17530967|ref|NP_511174.1| sodium/calcium exchanger 2 precursor [Rattus norvegicus]

>R.norvegicus_NCX2.1

MAPLALVGVALLLGAPHCLGEATPTPSLPPPPANDSDASPGGCQGSYRCQPGVLLPVWEPDDPSLGDKAA

RAVVYFVAMVYMFLGLSIIADRFMASIEVITSKEKEITITKANGETSVGTVRIWNETVSNLTLMALGSSA

PEILLSVIEVCGHNFQAGELGPGTIVGSAAFNMFVVIAVCVYVIPAGESRKIKHLRVFFVTASWSIFAYV

WLYLILAVFSPGVVQVWEALLTLVFFPVCVVFAWMADKRLLFYKYVYKRYRTDPRSGIIIGAEGDPPKSI

ELDGTFVGTEVPGELGALGTGPAEARELDASRREVIQILKDLKQKHPDKDLEQLVGIAKYYALLHQQKSR

AFYRIQATRLMTGAGNVLRRHAADAARRPGANDGAPDDEDDGASRIFFEPSLYHCLENCGSVLLSVACQG

GEGNSTFYVDYRTEDGSAKAGSDYEYSEGTLVFKPGETQKELRIGIIDDDIFEEDEHFFVRLLNLRVGDA

QGMFEPDGGGRPKGRLVAPLLATVTILDDDHAGIFSFQDRLLHVSECMGTVDVRVVRSSGARGTVRLPYR

TVDGTARGGGVHYEDACGELEFGDDETMKTLQVKIVDDEEYEKKDNFFIELGQPQWLKRGISALLLNQGD

GDRKLTAEEEEAQRIAEMGKPVLGENCRLEVIIEESYDFKNTVDKLIKKTNLALVIGTHSWREQFLEAVT

VSAGDEEEDEDGSREERLPSCFDYVMHFLTVFWKVLFACLPPTEYCHGWACFGVCILVIGLLTALIGDLA

SHFGCTVGLKDSVNAVVFVALGTSIPDTFASKVAALQDQCADASIGNVTGSNAVNVFLGLGVAWSVAAVY

WAVQGRPFEVRTGTLAFSVTLFTVFAFVGIAVLLYRRRPHIGGELGGPRGPKLATTALFLGLWFLYILFA

SLEAYCHIRGF

>gi|148710162|gb|EDL42108.1| solute carrier family 8 (sodium/calcium exchanger), member 2, isoform CRA_a [Mus musculus]

>M.musculus_NCX2.1

MAPLALMGVVLLLGVPHCLGEATPTPSLPPPTANDSDASPEGCQGSYRCQPGVLLPVWEPEDPSLGDKVA

RAVVYFVAMVYMFLGVSIIADRFMASIEVITSKEKEITITKANGETSVGTVRIWNETVSNLTLMALGSSA

PEILLTVIEVCGHNFQAGELGPGTIVGSAAFNMFVVIAVCVYVIPAGESRKIKHLRVFFVTASWSIFAYV

WLYLILAVFSPGVVQVWEALLTLIFFPVCVVFAWMADKRLLFYKYVYKRYRTDPRSGIIIGAEGDPPKSI

ELDGTFVGTEVPGELGALGTGPAEARELDASRREVIQILKDLKQKHPDKDLEQLMGIAKYYALLHQQKSR

AFYRIQATRLMTGAGNVLRRHAADAARRPGATDGAPDDEDDGASRIFFEPSLYHCLENCGSVLLSVACQG

GEGNSTFYVDYRTEDGSAKAGSDYEYSEGTLVFKPGETQKDLRIGIIDDDIFEEDEHFFVRLLNLRVGDA

QGMFEPDGGGRPKGRLVAPLLATVTILDDDHAGIFSFQDRLLHVSECMGTVDVRVVRSSGARGTVRLPYR

TVDGTARGGGVHYEDACGELEFGDDETMKTLQVKIVDDEEYEKKDNFFIELGQPQWLKRGISALLLNQGN

GDKKITAEQEEAQRIAEMGKPVLGENNRLEVIIEESYDFKNTVDKLIKKTNLALVIGTHSWREQFIEAVT

VSAGDEEEDEDGPREERLPSCFDYVMHFLTVFWKVLFACVPPTEYCNGWACFGVCILVIGVLTALIGDLA

SHFGCTVGLKDSVNAVVFVALGTSIPDTFASKVAALQDQCADASIGNVTGSNAVNVFLGLGVAWSVAAVY

WAVQGRPFEVRAGTLAFSVTLFTVFAFVCIAVLLYRRRPQIGGELGGPRGPKLATTALFLGLWFLYILFS

SLEAYCHIRGF

>gi|80751141|ref|NP_001032179.1| solute carrier family 8 (sodium/calcium exchanger), member 1a [Danio rerio]

>D.rerio_NCX1a

MGQSGTSSYFSLALNLSIFLLVFSYELTPVIAGSSKSSLDVDTSNANSSQETCGGSYECKEGVILPIWTP

VNPSFGDKLARATVYFVGLFYMFLGVSIIADRFMASIEVITSQEKEITIKKPNGETTTTTVRIWNETVSN

LTLMALGSSAPEILLSVVEVCGHNFDAGELGPNTIVGSAAFNMFVIIGLCVSVIPEGEHRKVKHLRVFFV

TATWSIFAYTWLYLILAVISPGIVEVWEGLLTLFFFPICVLFAWVADRRLLFYKYVYKRYRVGKKRGIII

ETEGEPELQSKADIEMDGGMLNSHAEEFLDGAVDNEDKDADEDEARREMAKILKELKQKHPEKEMEQLVE

LANYHVLSQQQKSRAFYRCQATRLMTGAGNILKKHAADQARKALGIHELRSEVSDNDISSKIFFDPGTYQ

CLENCGTVALNVVRRGGDLTSTVSVEYRTEDGTANAGSDYQFTEGVIIFKPGETEKEIRVDIIDDDIFEE

DEHFLVHLSNVKVISEGANNGNPGTNHVDALAGLGLPSTATVTIFDDDHAGIFLFEEPIVHISESIGMME

VKVVRTSGARGVVVIPYKTIEGTAKGGGEDFEDTHGVLEFQNDEISKTIQINIIDDEEYEKNKNFFLEIG

EPQLVEMSERKAMLLHECGGFVKTDKQLYGRDVYRKVQGRDKPIPSTIISISEDGEEETLTKKEKDERRI

AEMGRPTLGEHVKLEVIIEESYEFKSTVDKLIKKTNLALLVGTNSWRDQFVEAITVNSGDDDDEECGQEK

MPSCFDYVMHFLTVFWKVLFAFVPPTDYWNGWACFIVSIMMIGVLTAFIGDIASHFGCTIGLKDSVTAVV

FVALGTSVPDTFASKVAAIQDQYADASIGNVTGSNAVNVFLGIGVAWSIAAIFHQSQGRQFRVDPGTLAF

SVTLFTIFAFVCIAVLMYRRRPEIGGELGGPRHPKIITTTLFFSLWLMYIILSSMEAYCIIKGF

>gi|33946311|ref|NP_892114.1| sodium/calcium exchanger 3 isoform C precursor [Homo sapiens]

>H.sapiens_NCX3.3

MAWLRLQPLTSAFLHFGLVTFVLFLNGLRAEAGGSGDVPSTGQNNESCSGSSDCKEGVILPIWYPENPSL

GDKIARVIVYFVALIYMFLGVSIIADRFMASIEVITSQEREVTIKKPNGETSTTTIRVWNETVSNLTLMA

LGSSAPEILLSLIEVCGHGFIAGDLGPSTIVGSAAFNMFIIIGICVYVIPDGETRKIKHLRVFFITAAWS

IFAYIWLYMILAVFSPGVVQVWEGLLTLFFFPVCVLLAWVADKRLLFYKYMHKKYRTDKHRGIIIETEGD

HPKGIEMDGKMMNSHFLDGNLVPLEGKEVDESRREMIRILKDLKQKHPEKDLDQLVEMANYYALSHQQKS

RAFYRIQATRMMTGAGNILKKHAAEQAKKASSMSEVHTDEPEDFISKVFFDPCSYQCLENCGAVLLTVVR

KGGDMSKTMYVDYKTEDGSANAGADYEFTEGTVVLKPGETQKEFSVGIIDDDIFEEDEHFFVRLSNVRIE

EEQPEEGMPPAIFNSLPLPRAVLASPCVATVTILDDDHAGIFTFECDTIHVSESIGVMEVKVLRTSGARG

TVIVPFRTVEGTAKGGGEDFEDTYGELEFKNDETVKTIRVKIVDEEEYERQENFFIALGEPKWMERGISA

LLLSPDVTDRKLTMEEEEAKRIAEMGKPVLGEHPKLEVIIEESYEFKTTVDKLIKKTNLALVVGTHSWRD

QFMEAITVSAAGDEDEDESGEERLPSCFDYVMHFLTVFWKVLFACVPPTEYCHGWACFAVSILIIGMLTA

IIGDLASHFGCTIGLKDSVTAVVFVAFGTSVPDTFASKAAALQDVYADASIGNVTGSNAVNVFLGIGLAW

SVAAIYWALQGQEFHVSAGTLAFSVTLFTIFAFVCISVLLYRRRPHLGGELGGPRGCKLATTWLFVSLWL

LYILFATLEAYCYIKGF

>gi|17530969|ref|NP_511175.1| sodium/calcium exchanger 3 precursor [Rattus norvegicus]

>R.norvegicus_NCX3.3

MAWLRLQPLTSAFLHFGLVTFVLFLNGLRAEAGDLRDVPSAGQNNESCSGSSDCKEGVILPIWYPENPSL

GDKIARVIVYFVALIYMFLGVSIIADRFMASIEVITSQEREVTIKKPNGETSTTTIRVWNETVSNLTLMA

LGSSAPEILLSLIEVCGHGFIAGDLGPSTIVGSAAFNMFIIIGICVYVIPDGETRKIKHLRVFFVTAAWS

VFAYIWLYMILAVFSPGVVQVWEGLLTLFFFPVCVLLAWVADKRLLFYKYMHKRYRTDKHRGIIIETEGE

HPKGIEMDGKMMNSHFLDGNLIPLEGKEVDESRREMIRILKDLKQKHPEKDLDQLVEMANYYALSHQQKS

RAFYRIQATRMMTGAGNILKKHAAEQAKKTASMSEVHTDEPEDFASKVFFDPCSYQCLENCGAVLLTVVR

KGGDISKTMYVDYKTEDGSANAGADYEFTEGTVVLKPGETQKEFSVGIIDDDIFEEDEHFFVRLSNVRVE

EEQLEEGMTPAILNSLPLPRAVLASPCVATVTILDDDHAGIFTFECDTIHVSESIGVMEVKVLRTSGARG

TVIVPFRTVEGTAKGGGEDFEDTYGELEFKNDETVKTIRVKIVDEEEYERQENFFIALGEPKWMERGISA

LLLSPEVTDRKLTMEEEEAKRIAEMGKPVLGEHPKLEVIIEESYEFKSTVDKLIKKTNLALVVGTHSWRD

QFMEAITVSAAGDEEEDESGEERLPSCFDYVMHFLTVFWKVLFACVPPTEYCHGWACFVVSILIIGMLTA

IIGDLASHFGCTIGLKDSVTAVVFVAFGTSVPDTFASKAAALQDVYADASIGNVTGSNAVNVFLGIGLAW

SVAAIYWAMQGQEFHVSAGTLAFSVTLFTIFAFVCLSVLLYRRRPHLGGELGGPRGCKLATTWLFVSLWL

LYVLFATLEAYCYIKGF

>gi|17432811|gb|AAL39160.1|AF453257_1 sodium/calcium exchanger [Mus musculus]

>M.musculus_NCX3.1

MAWLRLQPLTSAFLHFGLVTFVLFLNCLRAEAGDSGDVPSAGQNNESCSGSSDCKEGVILPIWYPENPSL

GDKIARVIVYFVALIYMFLGVSIIADRFMASIEVITSQEREVTIKKPNGETSTTTIRVWNETVSNLTLMA

LGSSAPEILLSLIEVCGHGFIAGDLGPSTIVGSAAFNMFIIIGICVYVIPDGETRKIKHLRVFFVTAAWS

IFAYIWLYMILAVFSPGVVQVWEGLLTLFFFPVCVLLAWVADKRLLFYKYMHKKYRTDKHRGIIIETEGD

HPKGIEMDGKMMNSHFLDGNFTPLEGKEVDESRREMIRILKDLKQKHPEKDLDQLVEMANYYALSHQQKS

RAFYRIQATRMMTGAGNILKKHAAEQAKKTSSMSEVHTDEPEDFASKVFFDPCSYQCLENCGAVLLTVVR

KGGDISKTMYVDYKTEDGSANAGADYEFTEGTVVLKPGETQKEFSVGIIDDDIFEEDEHFFVRLSNVRVE

EEQLAEGMLPAILNSLPLPRAVLASPCVATVTILDDDHAGIFTFECDTIHVSESIGVMEVKVLRTSGARG

TVIVPFRTVEGTAKGGGEDFEDAYGELEFKNDETVKTIHIKVIDDKAYEKNKNYVIEMMGPRMVDMSVQK

ALLLSPEVTDRKLTVEEEEAKRIAEMGKPVLGEHPKLEVIIEESYEFKSTVDKLIKKTNLALVVGTHSWR

DQFMEAITVSAGGDEDEDESGEERLPSCFDYVMHFLTVFWKVLFACVPPTEYCPGWACFVVSILIIGMLT

AIIGDLASHFGCTIGLKDSVTAVVFVAFGTSVPDTFASKAAALQDVYADASIGNVTGSNAVNVFLGIGLA

WSVAAIYWAMQGQEFHVSAGTLAFSVTLFTIFAFVCLSVLLYRRRPHLGGELGGPRGCKLATTWLFVSLW

LLYILFATLEAYCYIKGF

>gi|118091715|ref|XP_421178.2| PREDICTED: similar to Na+/Ca2+ exchanger isoform 3 splice isoform 4 [Gallus gallus]

>G.gallus_NCX3

MAWLSLQPLTSAFLHFGLVTFVLFLHGLQVDAGLTGDSTSSVQNSSCSGSFDCKEGVILPIWYPENPSLG

DKIARVIVYFVALIYMFLGVSIIADRFMASIEVITSQEKEITIKKPNGETTTTTIRIWNETVSNLTLMAL

GSSAPEILLSLIEVCGHGFVAGDLGPSTIVGSAAFNMFIIIAICVYVIPDGETRKIKHLRVFFVTAAWSI

FAYIWLYMILAVFSPGVVQVWEGLLTLFFFPLCVLLAWIADRRLLFYKYMHKKYRTDKHRGIIIESEGDH

PKGIEMDGKMMNSHFLDGNLVTVEGKEVDESRKEMIRILKDLKQKHPEKDLDQLVEMANYYALSHQQKSR

AFYRIQATRMMTGAGNILKKHAAEQAKKSTSLHEVRPDEPEEFISKIYFDPCSYQCLENCGAVLLTVVRK

GGDVSKTIYVDYKTEDGSANAGADYEFTEGTVVLKSGETQKDFAVGIIDDDIFEEDEHFFVRLSNLRVVD

NEEPPELGNSPYPKAILASPCVATVTILDDDHAGIFTFECDVIHVSESIGVMEVKVLRTSGARGTVIVPF

RTVEGTAKGGGEDFEDAYGELEFKNDETVKTIHIKVIDDEEYEKNKSFFIELMSPRMVDMSLQKALILLA

ERKLTVEEEEAKRIAEMGKPILGEHPKLEVIIEESYEFKSTVDKLIKKTNLALVVGTHSWRDQFLEAITV

SAAGDEDEDESGEERLPSCFDYVMHFLTVFWKVLFACVPPTEYCNGWACFVVSILIIGMLTAVIGDLASH

FGCTIGLKDSVTAVVFVAFGTSVPDTFASKAAAIQDVYADASITNVTGSNAVNVFLGIGLAWSVAAIYWA

SQGQEFQVSAGTLAFSVTLFTIFAFICISVLLYRRRPHLGGELGGPRGCKLATTLLFVSLWLLYILFATL

EAYCYIKGF

>gi|190338724|gb|AAI63335.1| Solute carrier family 8 (sodium/calcium exchanger), member 4a [Danio rerio]

>D.rerio_NCX4a

MFHLRLSRFSFSSITPCLSSVLLLIFLSGLAHLSQASGDASHSGPGNCSGEDSCSEGVVLPIWNPQNPSV

GDKVARAIVYFVALIYMFLGMSIIADRFMSSIEVITSQEKEITIKKPNGETTTATVRIWNETVSNLTLMA

LGSSAPEILLSVIEVCGHKFEAGHLGPSTIVGSAAFNMFIIIALCVYVVPDGEVRKIKHLRVFFVTAAWS

IFAYIWLYLILSVFSPGVVEVWEAVLTFLFFPLCVVQAWIADRRLLFYKYVHKRYRTDKNRGIIIETEGD

GMFTKMDMEMDGQGANSHHKEALDGMLAGVEEGGGGGEEEEARREMARTLKELKQRHPEKDMEQLIEMAN

YQVLVQQQKSRAFYRIQATRMMIGAGNILKKHAADQARKVVSCHEANPQEEDPHTIYLEFEPSHYQCFEN

CGSLKLSVTRHGGDSGCTVKVDYRTEDGTANAGSDYEFAEGTLVFKPGETVKELTVGVIDDDIFEEDEHF

YVHLSNPRVVHRAEVSILDPNAVSPGNSIIGSSHVPPKAALGNAHSATVTIYDDDHAGIFTFESNSTRVS

ESVGIMQVKVHRTSGARGKVAVPYHTTEGTAKAGEDYDEVAGKLEFLNDETMKILEVKIIDDEEYEKNKT

FTIHLGEPVLLEIGQKHGDSNDNKPAVGAEEEEVAKMGCPSLGEHTKLEVVIEESYEFKNTVDKLIKKTN

LALVVGSSSWREQFVSAVTVSAGDDDEEESGEERLPSCFDYIMHFLTVFWKVLFAFVPPTEYWNGWACFI

VSITLIGVLTAVTGDLASHFGCTVGLKDTVTAVVFVALGTSVPDTFASKVAAIQDQYADASIGNVTGSNA

VNVFLGIGVAWTIAAVYWHSQGKKFQVPPGSLAFSVTLFTILALVCVVTLLYRRRPSVSGGELGGPRTPK

LLTAFLFIVLWLIYIMLASLEAYCHVPGF

>gi|47219419|emb|CAG01582.1| unnamed protein product [Tetraodon nigroviridis]

>T.nigroviridis_NCX4a

MPHTLISIVILLFPEVTRFSHGSVSHEDAGRTTGNCSSEDNCPEGVVLPLWNPQNPAVGDKVARAIVYFA

ALIYMFLGMSIIADRFMSSIEVITSQEKEITIKKPNGETTTTTVRIWNETVSNLTLMALGSSAPEILLSV

IEVIGHNFDAGALGPSTIVGSAAFNMFIIIAICVYVVPENETRKIKHLRVFFVTAAWSIFAYIWLYLILS

VFSPGEVQVWEAVLTFCFFPLCVLQAWIADRRLLFYKYVHKRYRADKTRGIIIESEGDAMFTKMDMEMDG

QGVNSHTHPKEALDGMLEGMEEGGGMISEQDQEEEARREMARTLKELKQRHPEKDIEQLIEMANYQVLIQ

QQKSRAFYRIQATRMMIGAGNILKKHAADQARKVVSCHEASGQEEDPHTIYLQFEPSHYQCFENCGSLKL

SVSRYGGESGCTVKVDYRTEDATASAGSDYEFAEGTLVFKPGETTKEFTVGIIDDDIFEEDEHFYQQHGG

SFLSRPPEAALGKAHTATVTIYDDDHAGIFTFESDSTKVSESIGNMQVKVHRTSGARGKVAIPYHTVEAT

AKAGEDYEEVSGKLEFQNDETMKLLNVKIIDDEEYEKNKTFTIVLEEPILLEVGQKHGDTNDNKTSVGPE

DVSKMGCPCLGEHTKLEVVIEESYEFKSTVDKLIKKTNLALVVGSSSWREQFVSAVTVSAGDDDEEESGE

ERLPSCFDYIMHFLTVFWKVLFAFVPPTEYWNGWACFFVSISLIGVLTAVTGDLASHFGCTIGLKDSVTA

VVFVALGTSVPDTFASKVAAIQDQYADASIGNVTGSNAVNVFLGIGVAWTIAAVYWHSKGKPFKVNPGSL

AFSVTLFTIMALVCVLVLLYRRRPSVSGGELGGPRTAKLVTFFLFISLWFVYILLASLEAYCHVHSF

>gi|1947092|gb|AAB52920.1| Na/Ca exchanger [Loligo opalescens]

>L.opalescens_NCX

MNPFKISFSWGVPLFLLGLFFDFAHASEDSNDTCTTEAETCRNGLIVPRWNPVGNLSVGDKLARATVYFV

LMFYLFLGVSIIADRFMAAIEVITSKEKDVVVKKPDGTTTVVNVRIWNETVSNLTLMALGSSAPEILLSV

IEVVGQKFEAGQLGPSTIVGSAAFNLFIITAICVTVIPNGEVRTIKHLGVFFITATWSVFAYLWLYFILA

VSSYGVVDVWEGLLTFMFFPATVVTAYIADRRLLNKYLSKKYRASKQKGVIVQCEGQDAEAGEGKSEDGA

LKEGGDDVEVREFEQHRKEYIEILREMRKKNPTLDMKTLEDMAESEAVNRGPKSRAFYRIQATRKLTGSG

NIIKKAKAQAGVAQPIVIDQKPEDEITRVSFDPGHYTVMENVGTFYGTVTREGGDLTKTLYVDYKTEDGT

ANAGSDYVYAEGTLVFYPMETHKQFPISIIDDDIFEEDEHFYIRLSNLRVGDSNGLFESGQAEAKAQLAN

PFLATVMILDDDHPGIFQIDEKEMSVTESSGEVEVRIIRTSGARGCVKVPFHSVDGTATYGKDYELVDKD

VIFDNDETEKFLRVRVVDDEEYEKNETFFIWLDEPYLVKKPTGSSSGSVVEDDDPVLAELGKPRRGENIK

ITVHIIESTEFKSVVDKLLKKANLSLVVGTSSWREQFIEAITVNAEGDDDDEEGEEEKLPSCMDYIMHFV

CLFWKVLFAFVPPTDYWGGWACFTVSIILIGVLTAFIGDLATYFGCTIGLKDAVTAVSFVALGTSVPDTF

ASKVAAINDKYADSSIGNVTGSNAVNVFLGIGIAWSIAAIYHAANGTVFRVDPGTLAFSVTIFCVFAVCT

IVLLVCRRHHLVGGELGGPPRCKYITSGILGSFWVSYLLLTGLMSYCHIPGF

>gi|110756703|ref|XP_393309.3| PREDICTED: similar to Na/Ca-exchange protein CG5685-PA, isoform A [Apis mellifera]

>A.mellifera_NCX

MPGNYSTRCEPGLVVPVWRPQENLNLSDIIFRGFVYFSLLLYLFIGVSIISDRFMAAIEVITSKEKELVV

RRQGKEPQIVVVRVWNETVANLTLMALGSSAPEILLSIIEIWAKNFEAGELGPGTIVGSAAYNLFVIISL

CVFVIPNGETRKIKHLRVFFVTATWSIFAYVWLYLILAVSSPGVLEVWEGILTFSFFPATVLTAYVADRR

LLIYKYLHKGYRMNKRGVIVQAEAGDSDGGVELEIKPQQDSFHNMMDADTPEAKEFEQTRRDYINTLREL

RKKYPALPLEQLEVMAHEEVLGKGPKSRAFYRVQATRKMVGAGNLSKKISERAQSDLSEVKAELQKQEAE

SIEIENVNAMRIFFEPGHYTVMENVGSFEVGVTRAGGDLSKPCTVDYCTEDGSAEAGSDYISAKGTLTFD

SGETKKTIKLSVIDDELFEEDEHFYVRLSNASQPAMLVSPSLATVMILDDDHSGVFGFPERDMELVESVG

QYPLRVMRYSGARGRVVVPYRTIEGTAKPGKQYMHTESSLTFEDNQTEKEITLEIIEEDSYEKDALFYVE

LGEPQLQGGDGLAAEMKQPEERTAEEKMALLGKPKLGEVFRAQIRIKESKEFKNTVDKLVQRANASILLG

TSSWKEQFTEALTVSGGDEDDEGGGEPAAPSTLDYLMHGVTILWKVLFAFVPPTDIAGGYLCFVVSIFGI

GVVTAVIGDVASYFGCTLGIKDSVTAVAFVALGTSIPDTFASKVAACQDKYADASVGNVTGSNAVNVFLG

IGVAWSIAAIYHALHGDKFRVKPGNLAFCVTLFCTEACLVILVLLLRRTKSIGGELGGPFVPKVITSLIL

FFLWVFYLIMSILEAYGYIEGF

>gi|2266953|gb|AAB63464.1| CALX [Drosophila melanogaster]

>D.melanogaster_NCX

MQLLLKSIFTCALFVIFVYATAQSLLKVQETEARQAYLNVTSSSSSNLSQDDGHFLSRRLRQVSHGEEGD

EGAPSQMDDELEQMTKVHGEAPDAEDVRECSEGLVLPLWMPQRNISVGDRLVRGFVYFVLLIYLFVGVSI

IADRFMAAIEAITSIERAVVVKGPNNTKQVMHVRIWNETVANLTLMALGSSAPEILLSVIEIYAKDFESG

DLGPGTIVGSAAYNLFMIIAVCMIWIPAGEVRRIRHLRVFFVTALFSVFAYVWLWLILSVFTPGVILVWE

AIVTLLFFPLTVLWAYIAERRLLVYKYMDKNYRVNKRGTVVAGEHDQVEMDAEKGPKQPMVTSARGNDAE

AFDEARREYITLLTELRQKYPDADLEQLEMMAQEQVLARSGRSRAFYRIQATRKMVGSGNLMRKIQERAH

SDLTGVKAQLHAGDDEEADDPIRMYFEPGHYTVMENCGEFEVRVVRRGDISTYASVEYETQDGTASAGTD

FVGRKGLLSFPPGVDEQRFRIEVIDDDVFEEDECFYIRLFNPSEGVKLAVPMIATVMILDDDHAGIFAFT

DSVFEITESVGRFELKVMRYSGARGTVIVPYWTENDTATESKDYEGARGELVFENNESEKFIDLFILEES

SYEKDVSFKVHIGEPRLAPDDELAAKIKEVEKKPVQDLTELDRILLLSKPRNGELTTAYVRIRESQEFKA

TVDKLVAKANVSAVLGTSSWKEQFKDALTVIPADESEFDNDDEEEEVPSCFSYVSHFVCLFWKVLFAFVP

PTDICGGYVTFVVSIFVIGVITAIIGDAASYFGCALNIKDSVTAILFVALGTSIPDTFASMIAAKHDEGA

DNCIGNVTGSNAVNVFLGIGLAWTIAAVYHSSHGMTFNVEPGTIGFAVALFCGEALIAIMLIMFRRWHKG

IGAELGGPKVSKYISAAILVFLWVFYVVICILEAYDVIRV

>gi|115533320|ref|NP_001041182.1| Na/Ca eXchangers family member (ncx-1) [Caenorhabditis elegans]

>C.elegans_NCX1

MTKLKIYLFLVVSLTTLGQYAAEPQNGEIIHVSSQRIPGPEPACAPAKPCSPGVIVPVWQPSENLSECKI

WFRAIVYLIALAYLFFGVSIVADRFMASIEVITSQQKSVKMKKITGEHFTIMVRVWNETVSNLTLMALGS

SAPEILLSVIEICGNNFEAGELGPSTIVGSAAFNLFIIIAVCIMAIPNGETRRVQHNGVFWVTVVWSTFA

YVWLYLILSVFSPGEVEVWEGVLTFVFFPLTVGSAYFADAHAGQFGQRLISGPLSSFVRRSPRRSPSKKT

RENVENGAGLPGDATQNLIGGDADALAFEIHRRHYLDIFKQLRSEHPDAPVVELEKHAMEKVVGEQKKSR

AFYRIQTTRKMIGSGDIQKKLKKSNKLEPMVVQKTMATVEFDPPHYTCLENVGDVYLTVKCDRGSVPEDT

TVTVHYRTIADTAQAESDFVHTEGTITFEPGQTEQKIKVGIVDNDIYEDDEQFMVRLSQVRAFRSEHFSS

VPARLGLAATATVIIVDDDHAGSFGFLSEKFKCTESCGSFVAEVIRSRGARGKVSIPYKTVDGAAKSPQD

YEHQEGVLKFADEQSKAEIYIPIVNDDEYEKHEDFYIELGEPIWHRELADDEEGIEGKPILGFSRCKVVI

TEDREFKNFMDRALVTANTSIMVGTSSWKQQFTEAWTLEPEEEDGEVTTMEKVMHYIALPWKLLFALIPP

TDYFNGWLCFVVAIAMIGLLTAFIGDIAAAFGCTVGLKDSVTALTLVAMGTSLPDTFASRTAAVGDQWAD

GSIGNVTGSNAVNVFLGIGIAWMIAACVHAYRGTKFLVATGSLAFSVTMFLIGSVVCVALLQYRRFNRKV

NGELGGPMGWRIISAGIFVSVWLLYILLSTLEAYCIIKGF

>gi|71983528|ref|NP_504414.4| Na/Ca eXchangers family member (ncx-2) [Caenorhabditis elegans]

>C.elegans_NCX2

MTRLGCWLAVAFLVALAGLADAGSNCSAADATRNCIDGLVIPIWRPFLDLTTGDRVLRGVLYFFVIAYMF

LGISIVADRFMSSIEVITSMERTIVVKRPGLDPMAVQVRIWNDTVSNLTLMALGSSAPEILLSIIEVIAR

GFEAGDLGPNTIVGSAAFNLFMIIAICVVVIPKGEIRRQKHLDVFCVTATWSVFAYVWLYLILAFFSPGE

IEIWEGALTFIFFPLTVFTAYMADIKLIQNKFLPHRYRRGSHGQMIATEAEEMKMLENGTQGDPALKAFE

EHRQEFIELMREIRKQNPHITPTELQKQAEYEMISRGPKSRAFYRVQATRRLIGGGDIVKKRIDKEHNKA

LDAAQEKQSRDNTCKIFLDPAHYTVLESVGSFDVVVGRDGGPDGLTVMVDYFTEDGSANAGSDYIPVKGT

LTFYPEDKHQKVTIEVVDDDVFEEDEHFYLRLCNLRVRTKDGIIIDPTRIGGLPVAQLEMPNTATIMILD

DDHAGVFGFEHDHFQVVENCGHLSLQMKRHSGARGKVIIPFRTVEGTASADKHFEMKEGEIVFEDNQTEA

LVEIGIVDTEQYERSDYFYIELSPPIWAKKMNDLSRIQERFQRRMERKRGSSVASESKDSNTENALAPAE

KSTRAASVDLLQPSSDPRRSSQTNSPHLTSRFRNRLGSWIAGMKGGNGDDEVTTSLTPSQLEIAEMGKPR

LGEFTKCQITIRESKEFQGIVDRMIKNANTRIMLGTHSWREQFMEALVVSAGDDDDDEGEDGEDGEEKEP

EEPGCMDYVMHVLTVPWKLTFATIPPTDYFGGWATFVVAIFMIGVLTAVVGDLASQFGCWVGLKDAVTAI

SFVALGTSVPDTFASKVSAVQDKYADNAVGNVTGSNAVNVFLGIGIAWSMAAIYHWNQGTKFLVDPGNLG

FSVLIFCTEAVLCIIVLVLRRNKKVGGELGGPIALRWMTAMFFASLWFMYLLLSALEAYCIIPGF

>gi|268562054|ref|XP_002638487.1| Hypothetical protein CBG12917 [Caenorhabditis briggsae]

>C.briggsae_NCX1

MRVSLKTITNAMTKLKIILFLVVSFGQFQAQQQGNTEVVRVFSQRLPSPEDTACPPAKPCAPGVIVPVWQ

PSENLSECKIWFRAIVYLLVLAYLFFGVSIVADRFMASIEVITSQTKSVKMKKITGEPFTVLVRVWNETV

SNLTLMALGSSAPEILLSVIEICGNNFEAGELGPSTIVGSAAFNLFIIIAVCIMAIPNGEIRRVQHNGVF

WVTVVWSTFAYVWLYLILSVFSPGEVEVWEGVLTFVFFPLTVASAYFADAHAGQFGQRLISGPLSSFVRR

SPRRSPSKKTHENALESGNGGIPGDQNQSLIGGDADALAFEIHRRHYLDIFKQLRSEHPDAPVDELEKHA

MEKVVGEQKKSRAFYRIQTTRKMIGSGDIQKKMKNKKGLLEPLVVKQTMATVEFDPPHYTCLENVGDVYL

TVKCDRGNVPEDTTVTVHYRTIADTAQENSDFMPAEGTITFEPGQTEQKIKVGIVDNDIYEDDEQFMVRL

SQVRAFRSEHFSAVPCRLGPAATATVIIVDDDHAGCFGFASEKFKCVESCGSFVAEVIRSRGARGEVSIP

YKTIDGAAKSPQDYVHQEGVLKFDDEQSKAEIYIPITNDDEYEKHEDFFIELGDPIWHKEIKDDEEGAEG

KPILGFARCKVVITEDRDFKNFVDKVLVTANTSIMVGTSSWKQQFTEALTLEPEEEGGDVSLQEKIMHYV

ALPWKLLFALIPPTDYFNGWCCFVVAIIMIGVLTAFIGDIAAAFGCTVGLKDSVTALTLVAMGTSLPDTF

ASRTAAVGDQWADGSIGNVTGSNAVNVFLGIGIAWMIAACVHAYRGTKFLVSTGSLAFSVTMFLIGSVIC

VALLQYRRFNRKINGELGGPTSWRVISAGIFVSVWLLYILLSTLEAYCVIKGF

>gi|309363385|emb|CAP27025.2| CBR-NCX-2 protein [Caenorhabditis briggsae AF16]

>C.briggsae_NCX2

MTRLGCWLAVAALVALAGLADAGSNCSAADATRNCIDGLVVPIWRPFLDLTTGDRVLRGVLYFFVIAYMF

LGISIVADRFMSSIEVITSMERTIVVKRPGLDPMEVQVRIWNDTVSNLTLMALGSSAPEILLSIIEVIAR

GFEAGDLGPNTIVGSAAFNLFMIIAICVVVIPKGEIRRQKHLDVFCVTATWSIFAYVWLYLILAFFSPGE

IEIWEGLLTFVFFPLTVGTAYMADIKIIQNRFLPHRYRRGSHGQMIATEAEEMKMLENGSPQGADPALKA

FEEHRQEFIELMREIRKQNPHITPTELQKQAEYEMISRGPKSRAFYRVQATRRLIGGGDIVKKRIDKEHN

KALDALVQAQEKQLRDNTCKIFLDPAHYTVLESVGSFDVVVGRDGGPDGLTVMVDYFTEDGTANAGSDYI

PVKGTLTFYPEDKHQKVTIEVVDDDVFEEDEHFYLRLTNLRVRTKDGIIIDPTRIGGLPVAQLEMPNTAT

IMILDDDHAGVFGFDHDHFQVVENCGHLSLQMKRHSGARGKVVIPFRTIEGTAQADKHFEMKEGEIVFED

NQTEATIDIGIIDTEQYERSDYFYIELSPPIWAKKMNDLSRIQERFQRRMERKRGSSVASESKDSNTETA

LAPTDKSTRAASVDLLQPCSDPRRSSQSNSPHLTSRFRNRLGSWIAGMKGGNGDDEVTTALTPSQLEIAE

MGKPRLGEFTKCQITIRESKEFQGIVDRMIKNANTRIMLGTHSWREQFMEALVVSAGDDDDDDEEGDDGE

EKEPEEPGCMDYFMHVLTVPWKLTFATIPPTDYIGGWATFVVAIFMIGVLTAVVGDLASQFGCWVGLKDA

VTAISFVALGTSVPDTFASKVSAVQDKYADNAVGNVTGSNAVNVFLGIGIAWSMAAIYHWNKGTKFLVDP

GNLGFSVLIFCVEALLCIAVLVLRRSKKVGGELGGPTALRWMTSMFFCSLWLMYLLLSALEAYCIIPGF
