# Supplementary material for: Phylogeny and a structural model of plant MHX transporters
Source: BMC Plant Biol. 2013 May 2;13:75. doi: 10.1186/1471-2229-13-75 (PMC3679957; doi:10.1186/1471-2229-13-75)
Supplement: Additional file 4 — A rooted maximum likelihood phylogenetic tree of all proteins. [file 1471-2229-13-75-S4.doc]

**Additional file 4. A rooted maximum likelihood phylogenetic tree of all proteins**

The same maximum likelihood phylogenetic analysis presented in Figure 2 as an unrooted tree is presented here as a rooted tree. The analysis included all the proteins presented in Additional file 1, and was generated by *MEGA5* after alignment of the sequences. The bootstrap consensus tree inferred from 1000 replicates is presented. The percentage of replicate trees in which the associated proteins clustered together in the bootstrap test are shown next to the branches. Bootstrap values lower than 60% are not shown. See Methods for more details.

**
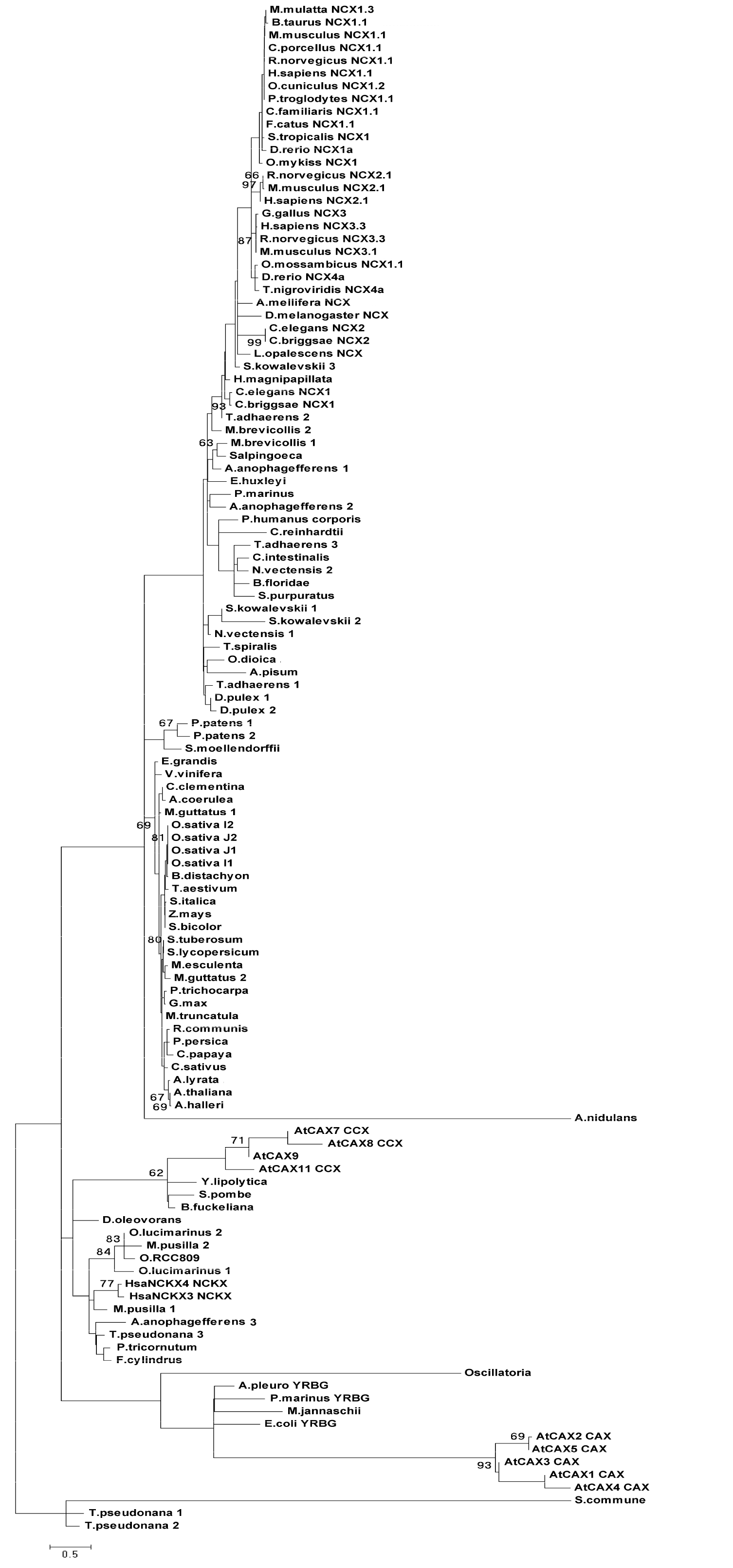
**
