# Supplementary material for: Phylogeny and a structural model of plant MHX transporters
Source: BMC Plant Biol. 2013 May 2;13:75. doi: 10.1186/1471-2229-13-75 (PMC3679957; doi:10.1186/1471-2229-13-75)
Supplement: Additional file 5 — Alignment of MHX proteins. [file 1471-2229-13-75-S5.pdf]

Additional file 5. Alignment of MHX proteins

The file presents the alignment of all currently identified MHX proteins. The sequences were aligned by Multalin using the identity-1-0 method. Identical residues were indicated by white letters on a red background and by uppercase letters in the consensus sequence. Similar residues, or the most conserved residues in each position, were indicated by red letters in the protein sequences and a lowercase letter in the consensus sequence. The similarity codes are ! - I or V, $ - L or M, % - F or Y, and # - D, E, N, or Q. Dots indicate gaps.

```
                                            1        10        20        30        40
A.thaliana           .......................MASILNQTQELQESSKVLGHLRCENFFLFPGENTLSDGLRGVLYFLG
A.halleri            .......................MASILNQTQELQEASKVLGHVRCENFFIFPGENTLSDGLRGVLYFLG
A.lyrata             .......................MASILNQTQELQEASKVLGHVRCENFFIFPGENTLSDGLRGVLYFLG
E.grandis            MQACFASVRLSLSSSLDSLPIFSVSFRRAGTPGDISRSHMMGHGVCESYLLFPGETALGDAFRTFLYFLG
C.clementina         ......................MASIHTQAIESISRAFNILHYEKCESYLLFRGETSLGDGFRAFLYFLG
R.communis           .....................MALETNYQSGWSRFGLLNILASEKCESYLVFRGETGLSVGFRTFLYFLG
M.esculenta          .....................MALETQNQSAGSKFGLLNILGRERCESYLLFHGETALDNGFRTFLYFLA
P.trichocarpa        ......................MASAYNQSDGGRFGDTNIVGHEKCESYFLFRGETTLGEGFRTFLYFLG
P.persica            .................................MPGNSSTMGQENCESYFIFQGETGLGVGFRTFLYFLG
C.papaya             ......................MTWAHWRNAESIIQNFNVLEQEKCESYLIFRSETALSNGFRAFLYFLG
A.coerulea           ............................................KCESYFLFQGEMALGNGLRAFLYLLG
V.vinifera           ......................MAWVHEQLPGNILEAYNISVREKCESYLLFSGETSLGDGFRTFLYFLG
G.max                .................................MVPFSNILGHEKCESYLIFGGETTLGDNFRAFFYFLC
M.truncatula         ............................MYKMIGYLSSFLAHDEKCESYLIFSGETNLGNSVRILLYFLG
S.tuberosum          ......................MTSLGNYTTDSTNGHSNILRHEKCDAYLLIHLETALGEGFRTFLYFLG
S.lycopersicum       ......................MTSLGNYTTHSTNGHSNILRHEKCDAYLLFHLETALGGGFRTFLYFLG
M.guttatus_1         ......................MDPLNNSTAEIIDGGSNILGVEKCQLYLLFHDETVLSDGFRGFLYFMA
M.guttatus_2         ...........................MASLNSSSTWDSNLGHERCLFYFIFHFETKLSHGLRGFLYFLA
C.sativus            ......................MASFAIENVESGQAISSISGTGKCESYFIFSIETSLGDALRIFLYFMG
O.sativa_J1          .................................MANINMADTVPSCDTYLLFNGETLLPIGVRAFIYTAV
O.sativa_I1          .................................MANINMADTAPSCDTYLLFNGETLLPIGVRAFIYTAV
O.sativa_J2          .................................MANINMADTAPSCDTYLLFNGETLLPNGVRAFIYTVV
T.aestivum           ......................................MGSTTRSCDAYLLFNGETLLPNGVRAFLCTVA
B.distachyon         .................................MANISMGSNAPSCNAYLLFYAEKMLPNGVRAFAYTVA
Z.mays               ..........................MAGAATPPPSSSSSASCGTDAAYLLFRGETLLPDGVHASLYAVA
S.bicolor            .......................MSNIAMVAAPPSSSCDSDT......YLLFHGETLLSTGARASLYTVA
S.italica            .....................................MSGPSSACEGTYLLFHGETLLSGGVRASLYTVA
S.moellendorffii     .............................................CPSLLLTQEEQRWRFGTRAFAYCLG
P.patens_1           ............MAGFTNASISAHPSPGVWTNHFANAAVNATAELCSNHIIFKAEPSWWLSARAILYGLC
P.patens_2           .............AGLSSALVSAFSSPEKNTIHVVHASATSTAELCPNHIMFEAEPTWLHSSRAVLYAFC
consensus>50         .............................................e.ce.yllf.gEt.l.dg.raflyflg

                      50        60           70         80        90       100       110
A.thaliana           LAYCFIGLSAITARFFK...SMENVVKHSRKVVT.IDPITKAEVITYKKVWNFTIADISLLAFGTSFPQI
A.halleri            LAYCFIGLSAITARFFK...SMENVVKHSRKVVA.IDPITKAEIITYKKVWNFTIADISLLAFGTSFPQI
A.lyrata             LAYCFIGLSAITARFFK...SMENVVKHSRKVVA.VDPITKAEVITYKKVWNFTIADISLLAFGTSFPQI
E.grandis            LAYCFIGLSAITARFFS...SMENVVKQTREVVE.IDPQTNSKVVRQEKVWNYTIADITLLAFGTSFPQI
C.clementina         LAYCFIGLSAITARFFR...SMENVVKHSRKVVE.IDPVTKAEVVRYEKVWNYAIADIALLAFGTSFPQI
R.communis           LAYCFIGLSAITGRFFR...SMENVVKHSRKVVE.IDPYSNTEVIRYEKVWNYTIADISLLAFGTSFPQI
M.esculenta          LAYCFFGLSAITARFFR...SMENVVKHSRKIVE.VDPDTNTEVIRYEKVWNYAIADISLLAFGTSFPQI
P.trichocarpa        LAYCFIGLSAITARFFR...SMENVVKQSRKVVE.IDPYTNAEVIRYEKVWNYAIADISLLAFGTSFPQI
P.persica            LAYCFVGLSAITARFFQ...SMESVVSHTRKVVD.INPYTGAEIIRYEKVWNFTIADISLLAFGTSFPQI
C.papaya             IAYCFLGLSAITGRFFR...SMEHVVKHTREVVE.IDPITNQEITRHEKVWNYTIADISLLAFGTSFPQI
A.coerulea           LAYCFFGLSAITARFFR...SMENVVKQTRKVVE.IDHNTNTEVIRHEKVWNYAIADITLLAFGTSFPQI
V.vinifera           LAYCFIGLSAITARFFQ...SMENVVKHKRKVVK.IDPRSNTE.IRHEKVWNYTIADITLLAFGTSFPQI
G.max                LAYCFIGLSAITARFFQ...SMENVVKHTRKVVE.VDPVTKTETIRHEKVWNYTIADISLLTFGTSFPQI
M.truncatula         LAYCFIGLSAITSRFFQ...SMENVVKHSREVVV.IDPVTKAETIRHEKVWNYTIADISLLAFGTSFPQI
S.tuberosum          LAYCFIGLSAITARFFR...SMESVVKHSRTVET.IDPLTNTKIVKNEKVWNYTIADITLLAFGTSFPQI
S.lycopersicum       LAYCFIGLSAITARFFR...SMESVVKHSRTVET.IDPLTNTKAVKNEKVWNYTIADITLLAFGTSFPQI
M.guttatus_1         LAYCFIGLSAITSRFFQ...SMENVVKHTRTVEE.IDPCTNTKTVRHEKVWNYTIADITLLAFGTSFPQI
M.guttatus_2         LAYCFVGLSAITDRFFR...SMENVVKHSRAVEE.IDPLTNTKVIKYEKVWNYTIADITLLAFGTSFPQI
C.sativus            LAYCFVGLSAITARFFR...SMENVVKHSRKVVE.IDPHTNTEIIRYEKVWNFTIADISLLAFGTSFPQI
O.sativa_J1          LAYCFIGLSAITGRFFK...SMESIMRHSREVVT.VDPHTNATIVKHEKVWNYTIADVALLAFGTSFPQI
O.sativa_I1          LAYCFIGLSAITGRFFK...SMESIMRHSREVVT.VDPHTNATIVKHEKVWNYTIADVALLAFGTSFPQI
O.sativa_J2          LAYCFIGLSAISGRFFK...SMESIMRHSREVVT.IDPHTNATIVKHEKVWNYTIADVALLAFGTSFPQI
T.aestivum           LAYCFIGLSAITARFFK...SMESITNHSREVVT.VDTETNTPIVKHEKVWNYTIADIALLAFGTSFPQI
B.distachyon         LGYCFIGLSAITARFFK...SMESITNHSREVVT.IDPHTNTPIVKHEKVWNYTIADIALLAFGTSFPQI
Z.mays               LAYCFVGLSAVTARFFK...SMEQIMRHSREVVVGVDPRTGAPVVRRDKVWNYTVADIALLAFGTSFPQI
S.bicolor            LAYCFIGLSAITARFFK...SMEQIMKHSREVVVI.DPHTKEPVVKHEKVWNYTVADIALLAFGTSFPQI
S.italica            LAYCFIGLSAITARFFK...SMEQIMKHSREVVS.VDPHTNAPVVKQEKVWNYTIADIALLAFGTSFPQI
S.moellendorffii     LAYCFLGLAVITRVYMRALDS....IVRHSRKSVHRDPITGLRIFSERRIWNPTVADITLLALGTCAPQV
P.patens_1           LLYCFVGLATITNLFMQAGGSAMGTIANRTRKIVRHNDESGSEEVVHVRVWNPVIADITLLALGTSAPQI
P.patens_2           IIYCFVGLATITNLYMEARSSAMEKIVHQTRKVVRHNYEIGSDEIVHERIWNLVIADITLLALGTSAPQI
consensus>50         laYCFiGLsa!tar%f....Smenvvkhsr.vv..i#p.tn.evi..ek!WNyt!AD!.LLafGTsfPQ!

                        120       130       140       150       160       170       180
A.thaliana           SLATIDAIRNMGERYAGGLGPGTLVGSAAFDLFPIHAVCVVVPKAGELKKISDLGVWLVELVWSFWAYIW
A.halleri            SLATIDAIRNIGERYAGGLGPGTLVGSAAFDLFPIHAVCVVVPKAGELKKISDLGVWLVELVWSFWAYIW
A.lyrata             SLATIDAIRNIGERYAGGLGPGTLVGSAAFDLFPIHAVCVVVPKAGELKKISDLGVWLVELVWSFWAYIW
E.grandis            SLATIDALRNLGELYAGGLGPGTLVGSAAFDLFPIHAVCVVVPKSGELKKISDLAVWAVELFWSFWAYIW
C.clementina         SLATIDAIRSIGNLYAGGLGPGTLVGSAAFDLFPIHAVCVVVPKAGELKKISDIGVWLVELFWSFWAYVW
R.communis           SLATIDAIRNIGNLYAGGLGPGTLVGSAAFDLFPIHAVCVVVPKAGELKKISDLGVWLVELFWSFWAYVW
M.esculenta          SLATIDAIRNIGNLYAGGLGPGTLVGSAAFDLFPIHAVCVVAPKAGELKKIADLGVWIVELFWSFWAYIW
P.trichocarpa        SLATIDAIQNIGNLYAGGMGPGTLVGSAAFDLFPIHAVCVVVPKAGELKKIADIGVWLVELFWSFWAYIW
P.persica            SLATIDAIRNLGNLYAGGLGPGTLVGSAAFDLFPIHAVCVVVPRAGELKKIADVGVWLVELFWSFWAYIW
C.papaya             SLATIDAIRNLGNLNAGGLGPGTLVGSAAFDLFPIHAVCVVVPKGGEMKKIADIGVWLVELFWSFWAYIW
A.coerulea           SLATIDAIRNLGQIYAGGLGPGTLVGSAAFDLFPIHAVCVVVPKAGELKKISDIGVWLVELFWSFWAYIW
V.vinifera           SLAIIDSIQNLGSRYAGGLGPGTLVGSAAFDLFPIHAVCVVVPKAGELKKISDVGVWLVELFWSFWAYVW
G.max                SLATIDAIQNIGKLYAGGLGPGTLVGSAAFDLFPIHAVCVVVPKAGELKKIADLGVWLVELFWSFWAYIW
M.truncatula         SLATIDAIRNLGDLYAGGLGPGTLVGSAAFDLFPIHAVCVVIPKAGELKKIADLGVWIVELFWSFWAYIW
S.tuberosum          SLATIDAIRNIGKLYAGGLGPGTLVGSAAFDLFPIHAVCVVVPKAGELKKISDIGVWLVELFWSFWAYIW
S.lycopersicum       SLATIDAIRNIGKLYAGGLGPGTLVGSAAFDLFPIHAVCVVVPKAGELKKISDIGVWLVELFWSFWAYIW
M.guttatus_1         SLATIDAIRNLGSLYAGGLGPGTLVGSAAFDLFPIHAVCVVVPKAGELKKISDVGVWLVELFWSFWAYIW
M.guttatus_2         SLATIDAIRNIGSLTAGGLGPGTLVGSAAFDLFPIHAVCVVVPKAGELKRISDIGVWLVELFWSFWAYIW
C.sativus            SLATIDAIRNIGNLYAGGLGPGTLVGSAAFDLFPIHAVCVVVPKAGELKKISDIGVWLVELVWSFWAYIW
O.sativa_J1          SLATIDAIRNLGQLTAGGLGPGTLVGSAAFDLFPIHAVCVVMPRAGSKKKISDLGVWLVELFWSFWAYIW
O.sativa_I1          SLATIDAIRNLGQLTAGGLGPGTLVGSAAFDLFPIHAVCVVMPRAGSKKKISDLGVWLVELFWSFWAYIW
O.sativa_J2          SLATIDAIRNLGQLTAGGLGPGTLVGSAAFDLFPIHAVCVVMPRAGSKKKISDLGVWLVELFWSFWAYIW
T.aestivum           SLATIDAIRNLGQLTAGGLGPGTLVGSAAFDLFPIHAVCVVMPRAGSMKKISDLGVWLVELFWSFWAYIW
B.distachyon         SLATIDAIRNLGQLTAGGLGPGTLVGSAAFDLFPIHAVCVIMPRAGSMKKISDLGVWLVELFWSFWAYIW
Z.mays               SLATIDAIRNLGQLTAGGLGPGTLVGSAAFDLFPIHAVCVLMPRAGSKKKISDLGVWLVELFWSFWAYIW
S.bicolor            SLAIIDAIRNLGQLTAGGLGPGTLVGSAAFDLFPIHAVCVVMPRAGSKKKISDLGVWLVELVWSFWAYIW
S.italica            SLATIDAIRNLGQLTAGGLGPGTLVGSAAFDMFPIHAVCVIMPKAGSKKKITDLGVWLVELFWSFWAYIW
S.moellendorffii     SLAVIDAFQHIGQ.TSGKLGAGTLLGSTAFNLFLILAVCVVAPKRFQTKSIRNVGVYIIEVVWSFWAYVW
P.patens_1           SLSIIDAIQQIGQKTNAGLGPETIVGSAAFNLYPILAVCVLVPKAGSVKRIQNVGVWIVELSWSIWAYVW
P.patens_2           SLAIIDAFQQLGQKTEAGLGPGTIIGSAAFNLYIILAVCVLVPKAGSTKHIRSIGVWIVELTWSMWAYIW
consensus>50         SLatIDairnlGqlyagg$GpgTlvGSaAF#$%pIhAVCVvvPkagelKkIsdlgVwl!ElfWSfWAY!W
```

```
                     190       200       210       220       230       240       250
A.thaliana        LYIILEVWSPNVITLVEALLTVLQYGLLLVHAYAQDKRWPYLSLPMSRGDRPEEWVPEEIDTSKDDNDND
A.halleri         LYIILEVWSPNVITLVEALLTVLQYGLLLVHAYAQDKRWPYLSLPMSRGDRPEEWVPEEIDTSKDDNDND
A.lyrata          LYIILEVWSPNVITLVEALLTVLQYGLLLVHAYAQDKRWPYLSLPMSRGDRPEEWVPEEIDTSKDENDND
E.grandis         LYIILKVWTPDIVTLWEALLTVLQYGLLLVHAYAQDKRWPYLSIPLARGERPEEWVPEEAASYKHVDNVD
C.clementina      LYIILEVWTPNVITFWEALLTVLQYGLLLIHAYAQDKRWPYLSLPIARTERPEDWVPQETTSCKDENCDY
R.communis        LYIILEVWTPNVITLWEALLTVLQYGLLLTHAYAQDKRWPYLSLPIERTERPEEWVPEEATSDKHQHNAY
M.esculenta       LYIILEVWTPNVITLWEALVTVLQYGLLLIHAYAQDKRWSYLSLPLGRTERPKEWVPEEITSSKHQPIDY
P.trichocarpa     LYIILEVWTPNVITLWEALLTVLQYGLLMHAYAQDKRWPYLSLPIPRTERPEEWVPGEVPSPTHENNVY
P.persica         LYIILEVWTPNVITIWEALLTVLQYGLLLMHAYAQDKRWPYFSLPLPRSERPEDWVPAEVVTCKSDSGPC
C.papaya          LYIILEVWTPNVVTLWEALLTVLQFGLLLTHAYAQDKRWPYLSLPLARTERPEDWVPEEVASVDHDNNDK
A.coerulea        LYIILEVWTPNVVTLWEALITVLQFGLLLIHTYAQDKRWPYLSLPLARTERPEDWVPVEASLDKLDNNDY
V.vinifera        LYIILEVWTPNVITIWEALLTVLQFGLLLIHAYAQDKQWPYLSLPLPRTERPEDWVPDEAASRKQDKIAG
G.max             LYIILEVWTPNVVTLWEALLTVLQYGLLLTHAYAQDKRWPYISLPIARDERPEDWVPEETPYFQHEAHAE
M.truncatula      LYIILEVWTPNVITLWEALLTVLQYGLLLIHAYAQDKRWPYISLPIARDERPEDWVPEETPKQKS..HER
S.tuberosum       LYIILEVWTPNVVTLWESILTVLQFGLLIHAYAQDKRWPYLSLPLERAERPEEWVPAEVVKHRPLDKVH
S.lycopersicum    LYIILEVWTPNVVTLWESILTVLQFGLLLIHAYAQDKRWPYLSLPLERAERPEEWVPAEVVKYRPLDKVH
M.guttatus_1      LYIILEVWTPNVITLLEALLTVLQFGLLLIHAYAQDKRWPYLSLPIGRTERPEEWVPVETAPYKDRDKYS
M.guttatus_2      LYIILKIWTPNVITLWEALLTVAQFGLLLIHAYAQDKRWPYVSLPMTRSERPEDWVPAE..KNAPYRDGN
C.sativus         LYIILEVWTPKVITLWEALLTVLQYGLLLTHAYAQDKRWPYLSLPLARTERPEEWVPPEI....DICKQD
O.sativa_J1       LYIILEVWTPRVITLWEALLTVLQYGLLLLHAYAQDKRWPYVSIPLARGERPEDWVPAEDASVDYDDNYD
O.sativa_I1       LYIILEVWTPRVITLWEALLTVLQYGLLLLHAYAQDKRWPYVSIPLARGERPEDWVPAEDASVDYDDNYD
O.sativa_J2       LYIILEVWTPRVITLWEALLTVLQYGLLLLHAYAQDKRWPYVSIPLARGDRPEDWVPTEDASVDYDDNYD
T.aestivum        LYIILEVWTPNVITLWEALLTVLQYGLLLVHAYAQDKRWPYVSIPLVRGERPEDWVPAEDTSLHHDKNCD
B.distachyon      LYIILEVWTPNVITLWEALLTVLQYGLLLVHAYAQDKRWPYVSIPLVRGERPEDWVPAEDTSVDHDKNCG
Z.mays            LYVILEVWTPRVITLWEASLTVLQYGLLLLHAYAQDKRCPFVSIPFARGERPEDWVPPEDVSAVYYAKCD
S.bicolor         LYVILEVWTPKVITLWEALLTVLQYGLLLLHAYAQDKRWPFVSIPFVRGERPEDWVPPEDVS.VDYDNCD
S.italica         LYVILEVWTPKVITVWEALLTVLQYGLLLLHAYAQDKRWPYVSIPLVRGDRPEDWVPEEGAS.VDYDNCN
S.moellendorffii  LFLILEIWTPNEITLWEAVLTVAQFPLLVLHAYVQDRNWKYLSIPLRESDAIESSDMNNLHELAN.....
P.patens_1        LAIILQVSSPNVVEPWEAMCTVLQFPILMTHAYIQDKGWGNFCRPLWPCMHVSWSPLKDFSLSVHNGTVH
P.patens_2        LAIILQVSSPDVVEPWEAVCTVLQFPILMMHAYVQDVGLETLCCPS..MIVSWKPIRKYFSLAIYDGEI
consensus>50      LyiILe!wtPnv!tlwEallTVlQ%glLliHaYaQDkrwpylslPl.r.erpedwvp.e..s...ddn..
```

```
                     260       270       280       290       300
A.thaliana        VHDVYSDAAQDAVESGSRNIVDIFSIHSANNDTGITYHTVADTPPDSATKKGKAKN..............
A.halleri         VHDVYSDAAQEAVESGSRNIVDIFSIHSANNDTGITYHTVADTPPDSATKKGKAKN..............
A.lyrata          VHDVYSDAAQEAVESGSRNIVDIFSIHSANNNTGITYHTVADTPPDSATKKGKAKN..............
E.grandis         GPMYHNVPASDLAESSNELHVLAL..............................................
C.clementina      DEC...SEILQLGEDENRGVVDIFSIHSPNGAVTSPLYQKVPGSEDVAEISKESFRE.............
R.communis        EEY...SEIVQVSEEDSRNIVDIFSIHSPVG..TDPVYQKVPETDEAAESSNNYSLS.............
M.esculenta       EEY...SEILQVDKDESRNVVDIFSIHSNVE..TDQVYHKVPETDDAVEFSDKNFHS.............
P.trichocarpa     GE............ENRNVVDIFSIHSNNG..T..VYQKVPVTDDAAESSNKHFHQ.............
P.persica         NNY...SEILEVGEDENRNIVDIFSFHSGSGLG..PVYQNVPGTDETPEYSNKDSPE.............
C.papaya          F...............VVDIFSIHSAP....STEYQKVPADENIPEPSGKSSRK.............
A.coerulea        SEI...LQLSE....ED.NIVDIFSIHSEKETGPLYQPVSNS...DVAESSNMNFHN.............
V.vinifera        DEC...SELFQ....ENGNIVDIFSIHSGD..GSVYHRISGS...EVAEPSNEHFQK.............
G.max             VDF...SDIKHVNE.ENGDTVDIFSIHSENPTDPLYARVPSIND....EAEILDKAK.............
M.truncatula      VEC...SEINHFNE.ENGDTVDIFSIHSENPRDMSYVRVPQIDD.....AENSDKVI.............
S.tuberosum       EPH...SEVSQVGEEENSGIVDIFSIH.SGEGTGHFYRN...LAGEDVTESSTPNNG.............
S.lycopersicum    EPH...SEVSQVGEEENTGIVDIFSIH.SGEGTGHFYQN...LAGEDVTESSTPNNC.............
M.guttatus_1      EIP...EDDHESGR.....IVDIFSVH.SD....HVYQN...LSGSDSGEPSGQSHE.............
M.guttatus_2      KTR...DNYPEVDEDRNSIIVDIFSIH.SANTGFVYENV...ADTDIHESSSEQCRV.............
C.sativus         NPC...REEFQAHENEQRSIVDIFSIHDSDGKVYHEVPG...HDIAESSNSNIPEEM.............
O.sativa_J1       GI....GDILPGQ...NEDIVDIFSAHSYSNEG.YHHV....SEEDVEESS.TGLTL.............
O.sativa_I1       GI....GDILPGQ...NEDIVDIFSAHSYSNEG.YHHV....SEEDVEESS.TGLTL.............
O.sativa_J2       GI....GDILPGQ...NEDIVDIFSARSYSNEG.YHHV....SEKDVEESP.TGLTL.............
T.aestivum        EN....SDILPSE...NDDVVDIFSIHSYSNAG.YHHV....PEKDIEESSKTTLVV.............
B.distachyon      ES....SEILPRE...NEDIVDIFSMHSYNNTG.YHHV....PEKDIEESSKTTLVV.............
Z.mays            GI....DETLPMGADGNDGIVDIFSAHSYYDAAEYSQL....AEEDMEGSSTMDQVV.............
S.bicolor         DT....NETLPISANRNDGIVDVFSAHSYHIA.EYSRV....PEKDMEGSSTMDQVV.............
S.italica         ET....SEILPGSSDKD..IADIFSGHSYHNA.EYHKV....PENDMECSSTMNNFV.............
S.moellendorffii  ............................................VSRSSKFAAMRELVATRPHNFRLFFFSSRAEDV
P.patens_1        DQSTAYSSSPRVDLEQHRSEWELVDLQHQPKQEIRNRVTCDDHPIKPIEWQQVAVAVDKNEDENDPG...
P.patens_2        HDQSASFSNLEVFSEQSTSDLERVDLQYLPMKEIRNGVTFDDHRISPFERQQVIGANDISEQLTCGSKNS
consensus>50      ......e..e.....n.nivdifsihs..n.............d..e.s.................
```

```
                                                   310       320       330       340       350
A.thaliana        ....................STVFDIWKHQFVDAITLETSESKKVDSIYLRIAKSFWHLLLAPWKLLF
A.halleri         ....................SSVFDIWKHQFVDAITLETSESKKVDSIYLRIANSFWQLLLAPWKLLF
A.lyrata          ....................STVFGIWKHQFVDAITLETSESKKVDSIYLRIAKSFWQLLLAPWKLLF
E.grandis         ..........................WKEQFVDALMLESAETKKLYSIYVRALRMLWQSLLAPWRLMF
C.clementina      ................EINSELPHVHALWKQQFVDSIALESPESRKMNNIYLRLARIFWQSLLVPWRVLF
R.communis        ................EKDL...DVVALWKQQFVDAIMLERPESRKLINSHLRLARISWQLFLVPWRLLF
M.esculenta       ................EQDF...NVAALWKRQFLDAITLESLESRNMFSIHIRLARTFWEILLAPWRLLF
P.trichocarpa     ................EKDL...HVLSLWKEQFVDALTLESLETRKLNSIHLRVARISWQLILAPWRLLF
P.persica         ................KMSLEDYHVFAIWGQQFVDAVKLKSTESRQLNNLYLRLARISWQLLLVPWRLLF
C.papaya          ................EILLEDPNVLTLWKQQFVDIFKVEKSDSRKLDNIYLQLARIVWQSLLAPWRFMF
A.coerulea        ................KSVLQDFNVFTVWKHQFMDAVMLENPESRKLDNTYLRLARGTWQVILAPWRVLF
V.vinifera        ................NIILEDSHLLSLWKQQFVDAFTLESSESRKLDNIYLWVARIFWQLLLPWRFLF
G.max             ................ETTLVDTHLLTIWRQQFLDALRLLRPESKKIKNACLRLACIFWQLLILPWRFLF
M.truncatula      ................ETRLEDTCLLTIWKQQFVDALTVESQESKKMNNIYIRTARIFWQLLLPWRFMF
S.tuberosum       ................NIIPEESDILSIWKHQFVDALMLESTESRKLNNIYLRVARIFWQLLLPWKLLF
S.lycopersicum    ................YIIPEESDILSIWKHQFVDALMLESTESRKLNNTYLRVARIFWQLLLPWKLLF
M.guttatus_1      ................DIHVKDNLI.SIWKQQFVDAFVLEGQESRKLNNTYLRAAKVLWELLLAPWRILF
M.guttatus_2      ................DTIPHKDDLLSVWKMQFVDALTLESPESKKRNNSKSLRVAKALWKLVLAPWRLLF
C.sativus         ................DGKADHPHVLKIWKQQFVDALSLETSESKQRNNIYLRSARLCWQLIVAPWRLLF
O.sativa_J1       ................KNKWEDTHWFSIWWQQFVDAATLESSVSRKMDSTCLRVIGISWNLIIAPWKMLF
O.sativa_I1       ................KNKWEDTHWFSIWWQQFVDAATLESSVSRKMDSTCLRVIGISWNLIIAPWKMLF
O.sativa_J2       ................KNKWEDTHWFSIWWQQFVDAATLESSVSRKMDSTCLSVIGISWNLIIAPWKMLF
T.aestivum        ................KNTQEDIYWLSVWRQQFVDAVMLESPELKKMDPVCLRFIIICWNSIIAPWKLLF
B.distachyon      ................KNTQEDTCWLSIWRQQFVDALMLESPEQRKMDSICLRFIIIFWNSIIAPWKLLF
Z.mays            ................KNTQDNMSWLSIWWQQFVDASMLESPESMKMDSVCLRYTRIFWNLIIAPWKFLF
S.bicolor         ................KNTQEDISWLSIWWQQFVDASMLESPESRKMHSICLRFTTIFWNLIIAPWKFLF
S.italica         ................KNTREDTSWRSLWRQQFVDAFMLESPESRKMASVCLRLIRIFWNLIAPWKLFF
S.moellendorffii  DTSSRDNFAFLREGAETEHFSWKALGTAWKNQFYDAMTVSFFEGRGQRKRTPSTLQLLLHPIVSYWKVSF
P.patens_1        .SNQEFLKQRLAYDIYEQTSMWTYIYSTWKKQFLDVIVFQGQVDDSGKNLALTAVEFIGYLITLPWRFIF
P.patens_2        ESNQELVKLRSTNEIDEHTSGRTNACSLWKMQFLDAIFIEKHVDESGKDHSPIAVDCVGHLIILPWRFLF
consensus>50      ...................ed..v.siWkqQFvDa..les.esrkld.i.lr.ari.wqlllapWrllF
```

```
                  360       370       380       390       400       410       420
A.thaliana        AFVPPCNIAHGWIAFICSLLFISGVAFVVTRFTDLISCVTGINPYVIAFTALASGTSWPDLVASKIAAER
A.halleri         AFVPPCNIAHGWIAFIFSLLFISGVAFVVTRFTDLISCVTGINPYVIAFTALASGTSWPDLVASKIAAER
A.lyrata          AFVPPCNIAHGWIAFICSLLFISGVAFVVTRLTDLISCVTGINPYVIAFTALAGGTSWPDLVASKIAAER
E.grandis         ALVPPCQIAHGWFAFICSLTFISGIAYVVTKLTDLISCVTGINAYVIAFTALATGTSWPDLVASKIAAER
C.clementina      AFVPPYHIAHGWIAFICSLLFISGIAYIVTKLTDIISCVTGINAYVIAFTALASGTSWPDLVASKIAAER
R.communis        AFVPPYHIAHGWIAFICSLLFISGIAYIVTQLTDLISCVTGINAYVIAFTALAAGTSWPDLVASKIAAER
M.esculenta       AFVPPYHIAHGWVAFICSLIFISGIAYIVTKITDLISCVTGINAYVIAFTALASGSSWPDLVASKIAAER
P.trichocarpa     AFVPPYHFAHGWIAFIFSLLFISGIAYIVTKLTDVISCVTGINAYVIAFTALASGTSWPDLVASKIAAER
P.persica         AFVPPYHIAHGWIAFICSLVFISAIAYIVTQLTDLISCVTGINPYVIAFTALASGTSWPDLVASKIAAER
C.papaya          AFVPPYHISHGWIAFVCSLLFISGISYIVTKLTDLVSCVTGINPYVIAFTALACGTSWPDLVASKIAVER
A.coerulea        AFVPPCHIAHGWIAFICSLIFISGIAYIVTKLTDMISCVTGINGYVIAFTALASGTSWPDLVASKIAAER
V.vinifera        AFVPPPHIAHGWFAFICSLIFISGIAYIVTKLTDLISCTSGINSYVIAFTALAAGTSWPDLVASKIAAER
G.max             AFVPPCQIAHGWISFICSLLFISGIAYVVTKITDVISCVTGINAYVIAFTALASGTSWPDLVASKIAAER
M.truncatula      AFVPPCHIAHGWISFICSLLFISGIAYIVTKLTDLISCVTGINAYVIAFTALASGTSWPDLVASKIAAKR
S.tuberosum       AFVPPYQIAHGWIAFICSLIFISGIAYVVTKITDLISCVTGINPYVIAFTALASGTSWPDLVASKIAAER
S.lycopersicum    AFVPPYQIAHGWIAFICSLIFISGIAYVVTKITDLISCVTGINPYVIAFTALASGTSWPDLVASKIAAER
M.guttatus_1      AFVPPCQIAHGWISFISSLIFISGIAYVVTKLTDIISCVTGINAYVIAFTALAAGTSWPDLVASKIAAER
M.guttatus_2      AFVPPYQIANGWIAFIFSLVFISGIAYIVTKITDLISCVTGINAYIIALTALAAGTSWPDLVASKIAAER
C.sativus         AFVPPYHIAHGWVAFICSLMFISGIAYVLTKFTDLISCVSGINPYVIAFTALASGTSWPDLVASKIAAER
O.sativa_J1       AFVPPYEIAHGWIAFICSLIFISGIAYGVTKITDQISCVTGVSPYVIAFTALAAGTSWPDLVASKIAAER
O.sativa_I1       AFVPPYEIAHGWIAFICSLIFISGIAYGVTKITDQISCVTGVSPYVIAFTALAAGTSWPDLVASKIAAER
O.sativa_J2       AFIPPYEIAHGWIAFICSLIFISGIAYGVTKITDQISCVTGVSPYVIAFTALAAGTSWPDLVASKIAAER
T.aestivum        AFVPPYQIAHGWIAFIFSLIFISGIAYGVTNITDQISCVTGLNPYVIAFTALAAGTSWPDLVASKIAAER
B.distachyon      AFVPPYQIAHGWIAFICSLIFISGIAYGVTKITDQISCVTGVNLYVIAFTALAAGTSWPDLVASKIAAER
Z.mays            AFVPPYHIAHGWIAFICSLIFISGIAYGVTKLTDQISCVTGVSPYVIAFTALAAGTSWPDLVASKIAAER
S.bicolor         AFVPPYNIAHGWIAFICSLIFISGIAYGVTKLTDQISCVTGVSPYVIAFTALAAGTSWPDLVASKIAAER
S.italica         AFVPPYHIAHGWVAFICSLIFISGIAYGVTKLTDQISCVTGVSPYVIAFTALAAGTSWPDLVASKIAAER
S.moellendorffii  AFIPPVQLLHGWLAFLGSICFITWISYVVALSNRINCVTGISSYVLALTVLAAGTSLPNLMASKIAAEE
P.patens_1        AFLPPPSLLHGWAAFLCALAHITVIACFLIKLTNLFGCVTGISKYTLALTVLAAGTSLPDLIASKIAAEI
P.patens_2        AFLPPPMLLNGWPAFMCALAFITVISCFLIKLANSFGCVTGVSDYVLALTILAVGTSWPDLIASKIAAKH
consensus>50      AfvPPyqiahGWiaFicslifIsg!ayvvtklt#lisCvtGinpYviAfTaLA.GtSwP#LvASKIAaer
```

```
                  430       440       450       460       470       480       490
A.thaliana        QLTADSAIANITCSNSVNIYVGIGVPWLINTVYNYFAYREPLYIENAKGLSFSLLIFFATSVGCIVVLVL
A.halleri         QLTADSAIANITCSNSVNIYVGIGVPWLINTVYNYFAYREPLYIENAKGLSFSLLIFFATSVGCIVVLVL
A.lyrata          QLTADSAIANITCSNSVNIYVGIGVPWLINTVYNYFAYREPLYIENAKGLSFSLLIFFATSVGCIVVLVL
E.grandis         QTTADSAIANITCSNSVNIYVGIGVPWLINTAYNFIMYREPLRIQNAAGLSFSLIVFFSTSVGCISVLVL
C.clementina      QITADSAIANITCSNSVNIYVGIGIPWLIDTAYNFIVYKEPLRVQNAEGLSFSLLVFFCTSVGCIAVLVL
R.communis        QITADSAIANITCSNSVNIYVGIGVPWLIDTAYNFFAYREPLRVQDAAGLSFSLLVFFSTSVGCIAVLVY
M.esculenta       QTTADSAIANITCSNSVNIYVGIGVPWLIDTTYNFFVYRQPLRIENAEGLSFSLLVFFATSVGCIGVLVY
P.trichocarpa     QTTADSAIANITCSNSVNIYVGIGVPWLIDTAYNFFMYNEPLRIQNAAGLSFSLLVFFCTSIGCIAVLVL
P.persica         QITADSAIANITCSNSVNIYVGIGVPWLIDTAYNFFVYKEPLRIENAAGLSFSLLVFFSTSVGCIAVLVI
C.papaya          QITADSAIANITCSNSVNIYVGIGVPWLIDTLYNFFAYREPLGIQNAEGLSFSLLVFFSTSVGCIAVLVF
A.coerulea        QTTADSAIANITCSNSVNIYIGIGIPWLIDTTYNFIAYREPLRIENADGLSFSLLIFFLTSIGCISVLVL
V.vinifera        QTTADSAIANIICSNSVNIYMGIGIPWLINTTYNFIAYREPLGVQNAEGLSFSLLVFFCTSICCIGVLVL
G.max             QKTADSAIANITCSNSVNIYVGIGVPWLIDTLYNFIAYREPLRIQNAEGLSFSLIVFFSTSVGCISVLVL
M.truncatula      QKTADSAIANITCSNSVNIYVGIGVPWLIDTLYNFIAYRQPLRIQNAGGLSFSLIVFFATSVGCISVLVA
S.tuberosum       QLTADSAIANITCSNSVNIYIGIGVPWLINTLYNYIAYNEPLRIDNAEGLSFSLLVFFSTSVACIGVLVF
S.lycopersicum    QLTADSAIANITCSNSVNIYIGIGVPWLIDTLYNYIAYNEPLRIENAEGLSFSLLVFFSTSVACIGVLVF
M.guttatus_1      QTTADSAIANITCSNSVNIYVGIGVPWLINTMYNYFAYNEPLRIENAGGLSFSLLVFFATSIGCIGGLVF
M.guttatus_2      QITADSAIANITCSNSVNIYIGIGVPWLIDTLYNYIAYKKPLRIENAAGLSFSLLIFFATSVGCIGVLVF
C.sativus         QTTADSAIANITCSNSVNIYVGIGVPWLISTTYNFIAYKEPLKIKDAGGLSFSLLVFFSTSVACIVVLVF
O.sativa_J1       QITADSAITNITCSNSVNIYVGIGVPWLVDTMYNYFVYQKPLYIDNAAGLSFSLLVFFATSFGCITVLVL
O.sativa_I1       QITADSAITNITCSNSVNIYVGIGVPWLVDTMYNYFVYQKPLYIDNAAGLSFSLLVFFATSFGCITVLVL
O.sativa_J2       QITADSAIANITCSNSVNIYVGIGVPWLVDTMYNYFVYKKPLYIDNAAGLSFSLLVFFATSFGCITVLVL
T.aestivum        QVTADSAIANITCSNSVNIYVGIGVPWLINTVYNFFAYQEPLYIDNAAGLSFSLLVFFATSFGCITVLVL
B.distachyon      QVTADSAIANITCSNSVNIYVGIGVPWLIDTVYNYFVYQEPLYIDNAAGLSFSLLVFFATSFGCITVLVL
Z.mays            QVTADSAIANITCSNSVNIYVGIGVPWLIDTVYNFFVYREPLYIDNAAGLSFSLLVFFATSFGCITVLVL
S.bicolor         QVTADSAIANITCSNSVNIYVGIGVPWLIDTVYNFFVYQEPLYIDNAAGLSFSLLVFFATSFGCITVLVL
S.italica         QVTADSAIANITCSNSVNIYVGIGVPWLIDTVYNFFVYREPLYIDNAAGLSFSLLVFFATSFGCITVLVL
S.moellendorffii  YDTADSAIANINASNCINVYVGFGVPWCVSALYSTGFHQNLV.VPVE.GLKFLLSVYFSTAITCFVALTA
P.patens_1        QPTADSAIANINASNCINVYVGIGIPWLMQSFYNWIHLKEEFRVPSA.GLGFALVLFFVTFAICLVVIIA
P.patens_2        LPTADSAIANINASNCINVYVGTGIPWLLQSFYNKLQLDEEFRVPSV.GIGFSLMLFLVTFVLCQIVVVG
consensus>50      q.TADSAIaNItcSNs!N!YvGiG!PWlidt.Ynffay.eplr!ena.GlsFsLlv%faTsvgCi.vlvl
```

```
                  500       510       520       530
A.thaliana        RRLIIGAELGGPRLWAWLTSAYFMMLWVVFVVLSSLKVSGVI
A.halleri         RRLIIGAELGGPRLWAWLTSAYFMMLWVVFVVLSSLKVSGVI
A.lyrata          RRLIIGAELGGPRLWAWLTSAYFMMLWVVFVVLSSLKVSGII
E.grandis         RRLTLGAELGGPKPWAWLTSVYLMLLWVIFLVLSSLKVSGII
C.clementina      RRLTLGAELGGPKLWAWVTSVYFMLLWIIFVVLSSLKVSGII
R.communis        RRLTLGAELGGPRIWAWVTSVYFMFLWLIFVVLSSLRVSGII
M.esculenta       RRLTLGAELGGPRIWAWVTCVYFMLLWLIFVVLSSLRVSGFI
P.trichocarpa     RRLTLKAELGGPRIWAWVTFVYFMLLWIIFVVLSSLRVSGII
P.persica         RRRTLGAELGGPRLWAWITFVFFMLLWLIFVVLSSLKVSGII
C.papaya          RRLTLGAELGGPRFWAWVTCVYFMLLWIIFVVLSSLRVSGII
A.coerulea        RRLTIGAELGGPRIWAWITSVYFMSLWIVFVVLSSLKVSDII
V.vinifera        RRLTLGAELGGPKIWARLTCVYFMSLWIIFVVLSSLKIYGII
G.max             RRIIFGAELGGPRLWAWITCAFFMLLWIIFVVLSSLKVSGFI
M.truncatula      RRIVFGAELGGPRLWAWITCAFFMLLWIIFVVLSSLKVSGFI
S.tuberosum       RRLTIGAELGGPRVWAWVTCIFFMLLWLIFVVLSSLRVSGII
S.lycopersicum    RRLTIGAELGGPRVWAWVTCIFFMLLWLIFVVLSSLRVSGII
M.guttatus_1      RRLTLGAELGGPKIWAWITCAYFMFLWLIFVVLSSLRVSGFI
M.guttatus_2      RRLTLGAELGGPRHWAWLTCFYLMLLWLVFVVLSSLQVSNII
C.sativus         RRVTLGAELGGPKVWAWITCIFFMVLWVIFVVLSSLKVSDII
O.sativa_J1       RRVILGAELGGPRMWAWATSVYFMILWVVFVVLSSLKISGVI
O.sativa_I1       RRVILGAELGGPRMWAWATSVYFMILWVVFVVLSSLKISGVI
O.sativa_J2       RRVILGAELGGPRMWAWATSVYFMILWVVFVVLSSLRISGVI
T.aestivum        RRIVFGAELGGPRLWAWATSAYFMVLWVVFVLLSSLRVSGVI
B.distachyon      RRIVLGAELGGPRLWAWVTSTYFMVLWVVFVVLSSLRVSGVI
Z.mays            RRIILGAELGGPRLWAWVTSVYFMILWVVFVVFSSLRVSGVI
S.bicolor         RRIILGAELGGPRLWAWVTSVYFMILWVVFVVFSSLRVSGVI
S.italica         RRIIIGAELGGPRLWAWITSVYFMILWIVFVVFSSLKVYGII
S.moellendorffii  RRRLLGGELGGPRKWAWASAIFFFLLWITFLTLACLAGRFT.
P.patens_1        RRFLFGGELGGPRKWAWVSSFCFLSLWLIFVIFSCLRNYHHL
P.patens_2        RRFIFEGELGGPRKWAWASSFYFIFLWLIFVIFSCLRNYNLL
consensus>50      RRl.lgaELGGPrlWAwvtsvyfmlLWviFvvlssL.vsgii
```
