# Supplementary material for: Phylogeny and a structural model of plant MHX transporters
Source: BMC Plant Biol. 2013 May 2;13:75. doi: 10.1186/1471-2229-13-75 (PMC3679957; doi:10.1186/1471-2229-13-75)
Supplement: Additional file 6 — The 5′ UTRs of plant MHXs. Presents the available 5′ UTRs of plant MHXs, their upstream AUG codons, the strength of the Kozak context of these codons, and the uORF peptides. [file 1471-2229-13-75-S6.doc]

**Additional file 6. The 5’ UTRs of plant *MHXs***

A. The 5’ UTR sequences that were available in the databases, or sequenced by us from the cDNA clones of the *S. lycopersicum*, *S. tuberosum* and *T. aestivum MHXs*, are presented. The name of each 5’ UTR is similar to that utilized for the corresponding protein. The uAUGs are underlined. It was shown that purines (A or G) at positions -3 and G at position +4 (where the A of the AUG is +1) are the most important nucleotides for AUG recognition [66,67]. The strength of the Kozak context of each uAUG codon is indicated on its -3 and +4 nucleotides, as follows: white letters highlighted in black – a strong context, black letters highlighted in gray – a sub-optimal context, and white letters highlighted in gray – a weak context. B. The peptides encoded by the uORFs of plant *MHX* genes (if their uAUGs are recognized). All possible peptide of each 5’ UTR are presented, and are indicated by sequential numbers. Peptides of different species that showed sequence similarity were aligned, and identical amino acids were highlighted in gray.

**Eudicots**

A.thaliana

ACGCTTGACCGATTCCAATCAGCTCCTCTCGATTTCCGTTTGTCGGAAAATCTCTCCCGTGATCGGCGTATTGTGA**ATG**CCGCTCACCGAGATATTCTCCGATTCTTTTCCCCAGTGAGGACAAGTGTTCAGTTGACTTATTAGGAGGTGGGGTTTGAATAAGTTACC

A.halleri

ACGCTTGCCCGATTCCAATCAGCTCCTCTCGATTTCCGTTTATCGGAAAGTCTCTCCCGTGATCGGCGGATTGTGA**ATG**CCGTTCACCGAGATATTCTCCGATTCTTTTCCCCAGTGAGGACAAGTGTTCAGTTGACTTAGCAGGAGGTGGGGTTTAAATAAGCTACA

S.tuberosum

GCACGAGGTACAAGCAATAATCCGGTGGTGCTCACTCC**ATG**CACGTCTCCTTCTTGTATATTCCCACCACATTTCTGTAAACTGCTATTCACAAATTAGTTCAAGACCCTATTTTTATTCCTTTTTTCCAATCACAAAGCGGTCCAAT**ATG**GAGCAAA**ATG**GATATCGATAGTCGTAT**ATG**CCTATTTGGTCCGCATCCGTCCGAAAGAATAATCTTTCTGGGGCTTGAACTGTGAAGGGAAAAATATTTATCAAG

S.lycopersicum

GCACGAGGTGTTTTTGGAAATAGCAAGTTGAAGTTCCTATTCGATCTCAATTACAAGCAATAGTCCGGTGGTGCTCACTCC**ATG**CACGTCTCCTTCTTGTATATTCTCACCACATTTCTGTAAACTACTATCTCACAAATCAGTTCAAGACCCCATTTTTATCCCTTTTTTTCAATCAATAAACTGCACAAT**ATG**GAGCAAA**ATG**GATATTGATAGTTGTAT**ATG**CCTATATTGTCCGCATCCGTCCGAAAGAATAATCTTTCTGGGGCTTGAACTGTGAAGAGAAAAATATTTATC

V.vinifera

TATATACCACATTTGCCGATTCTAGATTGTTGGAGACACTTAATCCGGTTCCCGAGGCCGTCGCATTCCGGGCCCGGCGAGTCTCCGCTGACGTTGGGATCGCTTTCTTACCTCTGGGACTGCACTGGGGAGGCGGGA**ATG**TAAATATTTTGCAAGAAGGTCTGTTGTTTCATTCTTGAACTGTGATTTGGGAACACTTAAG

P.trichocarpa

AAAGCAGCACCTGTCGCTATATTTTTCTCTAAAAACATTATTAACAATTTGAACCCGCACCCTCCTCATCTGTTGCCGTTCGGTCGTCAGCGATTGTCCGGTGAACCTGTTCTTGGTTTTGTTAGTGTTCTGCAATAATTGCTG**ATG**CTGCAGCTCATTTAAGCTGCAGCTTGAGGTTACTACG

M.esculenta

ACATTTCAGTCCAACGCATACAAATCCGATCTCCTCGTATTTCCCCGAGCTCAGAGAAAAGTGAAACACAC**ATG**CGGTCCTGAATTTT**ATG**AATTGTGCTGCGATTGTATTACACAGATAGATAGCTTCGCCTTAAATTTCGAATTGGATTGAGATATTGTTACTGTTCTGTAAGAACTACTACTGATTCGTCGGGCGCGAATTGCGACTTCAAGGCTATTCAG

C.sativus

TCTACCTTCCCCTCTCCCCCCCACCGCTGCCGTACCTCACTCATACAACCTTTCAACTGCATTCATTTTCCATCCATACCCATACTGGACGGCCGTACTCCGCTGCCGGATAGATCACCCGGACGCTG**ATG**TTACGGCCGGTTCTGAAGCTGTTACATTAGTTTACATACTCTTCCGGAGTTGATTAAA

M.guttatus_1

GTTCTACTTGTTGAGTGCATCTC**ATGATG**GAAAAATCTATTATCTATACTTAGCTTTCC**ATG**TCGAGCAGTAAATCCAAGT**ATG**CATATTCGTGTTGA**ATG**GAACA**ATG**T**ATG**AACTTGCTTACAT**ATG**CATTGCACTTTTCAGCTTCACGATTCTGCAA**ATG**ACGGT**ATG**CTGCTATTTGTTCGGGGAGATTGTTTCC**ATG**TCACGAGTGAGCTGTGAAATATAAACTACTCAGATTGAC

**Monocots**

T.aestivum

GCACACACGTCACCGCCTCGGGGAGCCCACATCGCCGCTGCTTCTCCAGCTACATCTTCTTCCTCAACCCCGATCCTGGTGGAGCGGCTAGGCTGCAGCAATCCGTGGGCAAAC**ATG**AGTGGGTTGAAAAAGAAGTTATTTTCTCTTTAGCAAGAACCAGAACTAAATCATATAGTTGTCTT**ATG**GCACGCTTCTG**ATG**TCCAACACTACA

Z.mays

CTAGCTTCCGTCACCGCTCCCCCAGTAGGCCAGTACCACCAAATCTCTAGCGAGGCCGCCCGCAACCGTGTCCCGGGGTTGAGGATCTGCGCGGGGCCGTCCTCGCGCGCGCCGCCGCATCCCCGCCGTCCCACTCCGGCGAGGCTGGCTGCGTTCCGTCGTCGTCCCCCGCACCGCCGCTGACTCCTGGAACAGGAACCAGCTTCTTCGCCCCCACCATCTCACGTCCAGCGTCGCGAACTCGCG

S.bicolor

ATTTCAGCTCACAA**ATG**CAGTAGCTTCCGTCACCGCTCCCCACTATCAAATCTGTCGCAAGTTCACGAAGCACAGTACCGGCAGCACGGAATCTGCGGGACCGTCCTCGCCTGCGCCACCGCGGGGGTCCCGCGCCGCCCGGCTCCGGCGAGGCTGCGACCCGCCGCTGACTGCTGGTACTGTCTCAGAGCGGCGCATTGGACTGGACAGTGGAGCAGAGCAACGGGGCCTCGCCGTCGGCGGAGAAGAAGGAGGCTGCAGCTGCTGTGCTTCGTGGTGATCCGATTCCTGCCACGGATTGGACCACTGGAACCAGCTAGAATTCTTTTCCCAGCTTCTCGTCTCATCTG**ATG**T

B.distachyon

ACGTGTGACAAGTAGGTACTCTGCCATTGCTCTCTCTGAAGAAAGAAACAGAACCAAATCAAGGTCCAGGGACGTGGATCTGCCACTGCCATTGCTCTTGGGGAGCCCCCAGATTTTGATTGGAGTCTAGTCTACCCATTAATCTCGTACATTTCCCATCGCTAAATTTTCATCAAAAAGGCTTTAGCTTATTTGTGGGAAGCGAGACTCCAAAATCCAAGCAGAAGAGGAAGAATCCAAGCAAGGGTGTCACGCGCACACGTCACCGCGTAGCGGGGCCCCACATCGCCGCCGCCGCCGCTACTGCCTCTTCTTCCTCCACCCAGGAACCAGAAATAAATCATATAGTTTTCATCTC**ATG**GTACTCTTGTG

O.sativa_J1

GCAGTAGCAAAGGTGGGCTTCTGCTTCCTTCATCCACACCAAAAGCAAGAACCCCAAGAACCGCAGATAAAAAGGGGAGCAAGAAGAACTTTGCTCCGATCCGCCGCCGCCATCTGCTCCCTCCACCCCGGAACTGGGAATACTGCAGTTCTCGTCTTATACTAGGCTACTAGCACTCCTCTGATCTG

S.italica

GATTCGGTCACGGCCTCGATCCGCAGTCTTGGGCGAGCTCCTCCTCAATTCCTTCCCCCACCCCTTTGGGTTTTCCTCCCCAATCCGCAGCAGAGTCTGTGCGGCGGGGCGGAGAGGAGAGGAGAGCAGAGCAGCGGGGGCGGCGGCGCCGCCGCGCGGAGGAAACGAAAGGCAACCACGCACGCCGGCGGCCTGGTTGCGTGCGCCGCCGTGGTTCGTGGGGGATCCGGTCGTCGATTCAACCACTGCAGGGAGTAGCAGCTAGCTAGGAGAATATTTTCTCAAGCTCCGGTCTTGGGGCTGCATCGGTCTTCCTG

**B. The peptides encoded by the uORFs of plant *MHX* genes**
