# Supplementary material for: Phylogeny and a structural model of plant MHX transporters
Source: BMC Plant Biol. 2013 May 2;13:75. doi: 10.1186/1471-2229-13-75 (PMC3679957; doi:10.1186/1471-2229-13-75)
Supplement: Additional file 7 — Alignment of NCX proteins. [file 1471-2229-13-75-S7.pdf]

Additional file 7. Alignment of NCX proteins

The file presents the alignment of all NCX proteins that were utilized as markers or identified in this work (excluding CrNCX). See the legends of Additional file 5 for details of sequence alignment and color annotation.

```
                                                                         1       10
H.sapiens_NCX1.1      ...................................................MYNMRRLSLSPTFSMGFHL
P.troglodytes_NCX1.1  ...................................................MYNMRRLSLSPTFSMGFHL
C.familiaris_NCX1.1   ......................................................MLQLRLLPTFSMGCHL
F.catus_NCX1.1        ......................................................MLRLRLSPTFSVGFHL
C.porcellus_NCX1.1    ......................................................MLRLSLSPTYSLGFHL
B.taurus_NCX1.1       ......................................................MLQFSLSPTLSMGFHV
R.norvegicus_NCX1.1   ......................................................MLRLSLPPNVSMGFRL
M.musculus_NCX1.1     ......................................................MLRLSLPPNVSMGFRL
M.mulatta_NCX1.3      ......................................................MRRLSLSPTFSMGFHL
O.cuniculus_NCX1.2    ......................................................MPRFSLSPPFSMGFHL
S.tropicalis_NCX1     ......................................................MMKLGTSLAFFARFHF
O.mossambicus_NCX1.1  ...............................................MSPVRTVPMFFTYKLIFFATVIS
O.mykiss_NCX1         ..............................................MRRTGTSSFLFCALQLTVLLAVFS
D.rerio_NCX1a         ..............................................MGQSGTSSYFSLALNLSIFLLVFS
H.sapiens_NCX3.3      ....................................................MAWLRLQPLTSAFLHFGL
R.norvegicus_NCX3.3   ....................................................MAWLRLQPLTSAFLHFGL
M.musculus_NCX3.1     ....................................................MAWLRLQPLTSAFLHFGL
G.gallus_NCX3         ....................................................MAWLSLQPLTSAFLHFGL
D.rerio_NCX4a         ....................................................MFHLRLSRFSFSSITPCL
T.nigroviridis_NCX4a  ................................................................MPHTLI
H.sapiens_NCX2.1      .........................................................MAPLALVGVTLLL
R.norvegicus_NCX2.1   .........................................................MAPLALVGVALLL
M.musculus_NCX2.1     .........................................................MAPLALMGVVLLL
S.kowalevskii_3       ...................................MMKLLCSILSRTLSELTTVSRGVGASALLLVVAFL
T.adhaerens_2         .....................................MQLFSALLWVIAVVVLNFKVDPTLGSSIISERD
L.opalescens_NCX      MNPFKISFSWGVPLFLLGLFFDFAHASEDSNDTCTTEAET..............................
A.mellifera_NCX       ......................................................................
D.melanogaster_NCX    ......MQLLLKSIFTCALFVIFVYATAQSLLKVQETEARQAYLNVTSSSSSNLSQDDGHFLSRRLRQVS
H.magnipapillata      ................................................................MQFVLT
C.elegans_NCX1        .......................................................MTKLKIYLFLVVSLT
C.briggsae_NCX1       ............................................MRVSLKTITNAMTKLKIILFLVVSFG
C.elegans_NCX2        ......................................................................
C.briggsae_NCX2       ......................................................................
T.spiralis            ......................................................................
M.brevicollis_1       ......................................................MRPQTWPAWAVLAMLV
Salpingoeca           ...............................................................MVRGLSL
S.kowalevskii_1       ......................................................................
S.kowalevskii_2       ......................................................................
T.adhaerens_1         ......................................................................
D.pulex_1             ......................................................................
P.humanus_corporis    ......................................................................
O.dioica              ......................................................................
A.pisum               ......................................................................
P.marinus             ..............................................................MIGLSLWG
A.anophagefferens_1   ......................................................................
E.huxleyi             ......................................................................
C.intestinalis        ......................................................................
B.floridae            ......................................................................
T.adhaerens_3         ......................................................................
N.vectensis_2         ......................................................................
consensus>50          ......................................................................
```

```
                        20        30        40        50        60        70        80
                        .         .         .         .         .         .         .
H.sapiens_NCX1.1        LVTVSLLFSHVDHVIAETEMEGEGNETGECTGSYYCKKGVILPIWEPQ.DPSFGDKIARATVYFVAMVYM
P.troglodytes_NCX1.1    LVIVTLLFSHVDHVIAETEMEGEGNETGECTGSYYCKKGVILPIWEPQ.DPSFGDKIARATVYFVAMVYM
C.familiaris_NCX1.1     LAVVALLFSHVDLISAETEMEGEGNETGECTGSYYCKKGVILPIWEPQ.DPSFGDKIARATVYFVAMVYM
F.catus_NCX1.1          LAFVPLLFSHVDLISADTEMEGEGNETGECTGSYYCKKGVILPIWEPQ.DPSFGDKIARATVYFVAMVYM
C.porcellus_NCX1.1      LAMMTLLISHVDHITAETEMVEEGNETGECTGSYYCKKGVILPIWEPQ.DPSFGDKIARATVYFVAMVYM
B.taurus_NCX1.1         IAMVALLFSHVDHISAETEMEGEGNETGECTGSYYCKKGVILPIWEPQ.DPSFGDKIARATVYFVAMVYM
R.norvegicus_NCX1.1     VTLVALLFTHVDHITADTEAETGGNETTECTGSYYCKKGVILPIWEPQ.DPSFGDKIARATVYFVAMVYM
M.musculus_NCX1.1       VALVALLFSHVDHITADTEAETGGNETTECTGSYYCKKGVILPIWEPQ.DPSFGDKIARATVYFVAMVYM
M.mulatta_NCX1.3        LVIVALLFSHVDYVIAETEMEGEGNETGECTGSYYCKKGVILPIWEPQ.DPSFGDKIARATVYFVAMVYM
O.cuniculus_NCX1.2      LAIVALFFFRVDHVSAETEMEGEGNETGECTGSYYCKKGVILPIWEPQ.DPSFGDKIARATVYFVAMVYM
S.tropicalis_NCX1       LVLLLLLCNVGSIRSETTTVADFENHTDSCTGSYYCKEGVILPIWEPQ.NPSFGDKIARATVYFVAMVYM
O.mossambicus_NCX1.1    TEFPYSAAAGSTLTL...TSNQTATNHSKCGGSTDCIEGVILPLWKPE.NPAFTDRLARATIYFVGLVYM
O.mykiss_NCX1           SEIKFVTAGNSNPSLGTNSSIGNQTNKKCDSVDTECKVGVILPIWLPE.NTSFGDKLARATVYFVALFYM
D.rerio_NCX1a           YELTPVIAGSSKSSLDVDTSNANSSQETCGGSY.ECKEGVILPIWTPV.NPSFGDKLARATVYFVGLFYM
H.sapiens_NCX3.3        VTFVLFLNGLRAEAGGSGDVPSTGQNNESCSGSSDCKEGVILPIWYPE.NPSLGDKIARVIVYFVALIYM
R.norvegicus_NCX3.3     VTFVLFLNGLRAEAGDLRDVPSAGQNNESCSGSSDCKEGVILPIWYPE.NPSLGDKIARVIVYFVALIYM
M.musculus_NCX3.1       VTFVLFLNCLRAEAGDSGDVPSAGQNNESCSGSSDCKEGVILPIWYPE.NPSLGDKIARVIVYFVALIYM
G.gallus_NCX3           VTFVLFLHGLQVDAGLTGDSTSSVQNS.SCSGSFDCKEGVILPIWYPE.NPSLGDKIARVIVYFVALIYM
D.rerio_NCX4a           SSVLLLIFLSGLAHLSQASGDASHSGPGNCSGEDSCSEGVVLPIWNPQ.NPSVGDKVARAIVYFVALIYM
T.nigroviridis_NCX4a    SIVILLFPEVTRFSHGSVSHEDAGRTTGNCSSEDNCPEGVVLPLWNPQ.NPAVGDKVARAIVYFAALIYM
H.sapiens_NCX2.1        AAPPCSGAATPTPSLPPPPANDSDTSTGGCQGSYRCQPGVLLPVWEPD.DPSLGDKAARAVVYFVAMVYM
R.norvegicus_NCX2.1     GAPHCLGEATPTPSLPPPPANDSDASPGGCQGSYRCQPGVLLPVWEPD.DPSLGDKAARAVVYFVAMVYM
M.musculus_NCX2.1       GVPHCLGEATPTPSLPPPTANDSDASPEGCQGSYRCQPGVLLPVWEPE.DPSLGDKVARAVVYFVAMVYM
S.kowalevskii_3         NFHPTLTQETFRNSTYPPVWDGGEEEEVCSRNATACKPGVLLPKWTPD.PVNVGDVAARATVYFAGLAYM
T.adhaerens_2           VSTINSTSTPTLSDAEKRLIACNTTTRCKNGLFLPHWPYPKDSKGNIL.PLSTGERVGRTIVYFFSLFFF
L.opalescens_NCX        ...................................CRNGLIVPRWNPVGNLSVGDKLARATVYFVLMFYL
A.mellifera_NCX         ...........................MPGNYSTRCEPGLVVPVWRPQENLNLSDIIFRGFVYFSLLLYL
D.melanogaster_NCX      HGEEGDEGAPSQMDDELEQMTKVHGEAPDAEDVRECSEGLVLPLWMPQRNISVGDRLVRGFVYFVLLIYL
H.magnipapillata        FVFIWFVNAVFANETIIFTNITVKNTTCRTLENLKCKPGVVLPVWKSADSESKAVIAGKAFVYLVAMFYF
C.elegans_NCX1          TLGQYAAEPQNGEIIHVSSQRIPGPEP.ACAPAKPCSPGVIVPVWQPSENLSECKIWFRAIVYLIALAYL
C.briggsae_NCX1         QFQ..AQQQGNTEVVRVFSQRLPSPEDTACPPAKPCAPGVIVPVWQPSENLSECKIWFRAIVYLLVLAYL
C.elegans_NCX2          .MTRLGCWLAVAFLVALAGLADAGSNCSAADATRNCIDGLVIPIWRPFLDLTTGDRVLRGVLYFFVIAYM
C.briggsae_NCX2         .MTRLGCWLAVAALVALAGLADAGSNCSAADATRNCIDGLVVPIWRPFLDLTTGDRVLRGVLYFFVIAYM
T.spiralis              ...................MLQYNNGTTALIFVENAYPCSDRGLLLPLIDESRWPTATRAFLYLIGLLYS
M.brevicollis_1         AACVVPGLAQAPGPEPRPSDPPCSDTVLSQGTPICKGGSGTLLPAFNAYGEADWPEGLKIVLYFVGLLWT
Salpingoeca             ALVATTVIVSLSLVAGVHGEPCGDRIVNNTEKPICKGGSGTFLPAFNAYGEDNWSDGLKIFLYLAGLLWT
S.kowalevskii_1         ..........................MSNSTTDPGAYYCYDKGLILPIVNESTWSIGARTTIYLIGLLWS
S.kowalevskii_2         ..........................MSNSTTDPDAYLCYDKGLILPIVNESTWSTGARITVYLIGLIYC
T.adhaerens_1           ...................................MSYCNKTGLLLPILDESNWNQGFRGFLYLAGLLYS
D.pulex_1               ...........................MTRNNGTLDDYTCSNALLLPLISEFTWSIEVRAFLYLFGLLYC
P.humanus_corporis      ......................MNVTGDLNELQNGEEDKCSESLFFPFLNDIHLNPIIRGILYFFALAYS
O.dioica                ........................MDGNVSVVDPGAYPQCAMGMLLPLVDESEMSMSLRIIMYLVGLFWF
A.pisum                 .......................MGHGNTSSYPWGPNDVCPGGLILPILDERLWPIKVRAVVYLFTLLYF
P.marinus               VDSPRSLATPVIEQPIPICEHGGSGLVLEDRLMGYVEGSHPQILPLFGDSEQDWPNGLRVVLYFLGLCWC
A.anophagefferens_1     ..........................................................RAVLYLLGLLWF
E.huxleyi               ..MLNSTAVITNETAPLLNDTCEDGLLLAGPLRTHRN......................ALIYMAALLWT
C.intestinalis          ..........MATTDVISYYSNGFVVEHVPIDETGNFTDAYCQSWLLIPAENLWPAWFRGFIYIIAIAYL
B.floridae              ..........MDLIAWQTNYSAGYVVETLPNGTEP......CESWLLLPAENLWPSALRGILYFLAMAYI
T.adhaerens_3           .......MAAIPNGSTFYNFSNGFIVEY..........DGSCSSWLLIIGENFWHPAVRCILYIAAMIYL
N.vectensis_2           ............MAYRTNFSRGYVVEYSPPCFE..........SWLLLPAENLWNDGIRGFLYILGMLYL
consensus>50            ...................................c..gvilpiw.pq.d.s.gd..ara.vYfval.ym
```

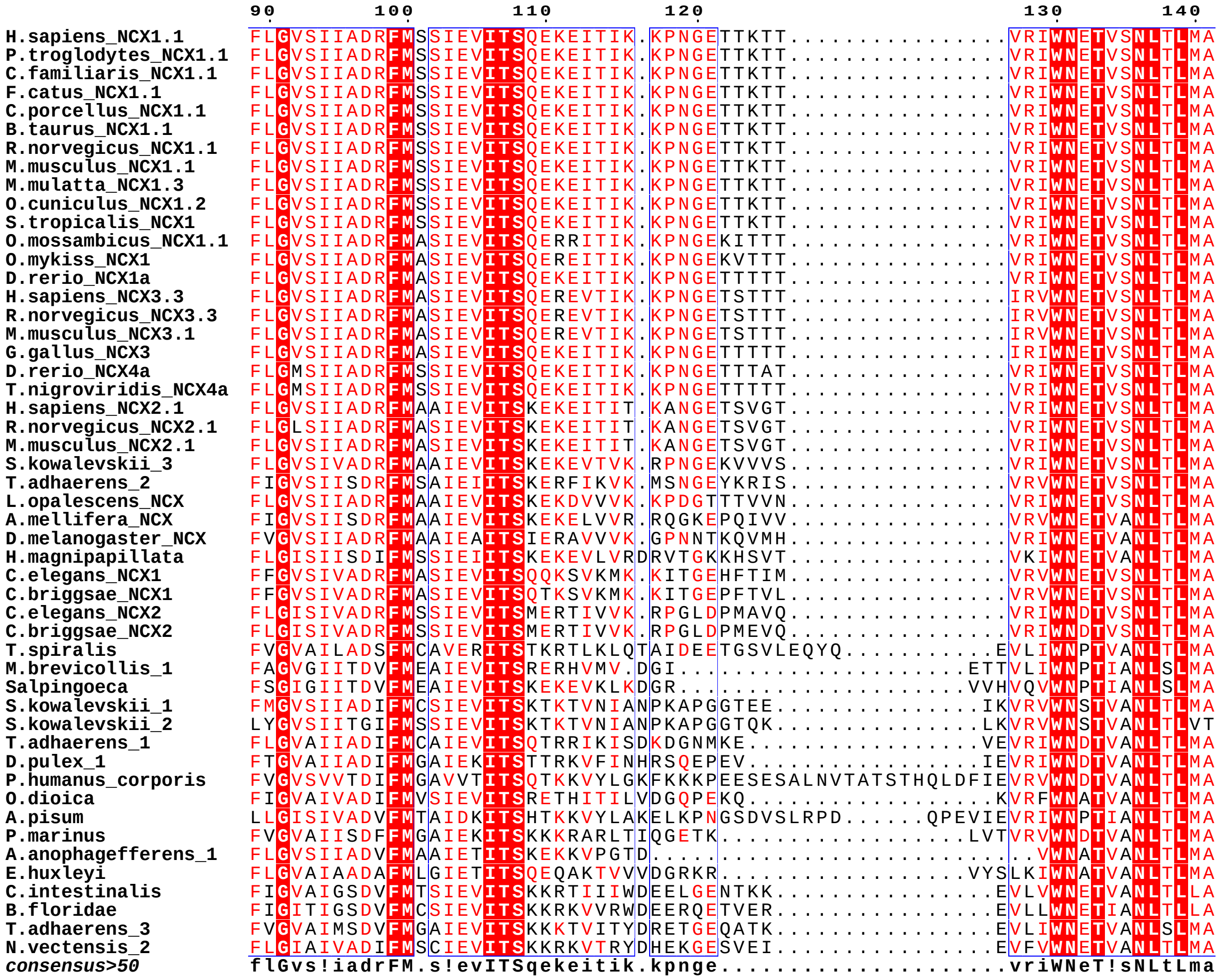
90 100 110 120 130 140
H.sapiens_NCX1.1 FLGVSIIADRFMSSIEVITSQEKEITIK.KPNGETTKTT................VRIWNETVSNLTLMA
P.troglodytes_NCX1.1 FLGVSIIADRFMSSIEVITSQEKEITIK.KPNGETTKTT................VRIWNETVSNLTLMA
C.familiaris_NCX1.1 FLGVSIIADRFMSSIEVITSQEKEITIK.KPNGETTKTT................VRIWNETVSNLTLMA
F.catus_NCX1.1 FLGVSIIADRFMSSIEVITSQEKEITIK.KPNGETTKTT................VRIWNETVSNLTLMA
C.porcellus_NCX1.1 FLGVSIIADRFMSSIEVITSQEKEITIK.KPNGETTKTT................VRIWNETVSNLTLMA
B.taurus_NCX1.1 FLGVSIIADRFMSSIEVITSQEKEITIK.KPNGETTKTT................VRIWNETVSNLTLMA
R.norvegicus_NCX1.1 FLGVSIIADRFMSSIEVITSQEKEITIK.KPNGETTKTT................VRIWNETVSNLTLMA
M.musculus_NCX1.1 FLGVSIIADRFMSSIEVITSQEKEITIK.KPNGETTKTT................VRIWNETVSNLTLMA
M.mulatta_NCX1.3 FLGVSIIADRFMSSIEVITSQEKEITIK.KPNGETTKTT................VRIWNETVSNLTLMA
O.cuniculus_NCX1.2 FLGVSIIADRFMSSIEVITSQEKEITIK.KPNGETTKTT................VRIWNETVSNLTLMA
S.tropicalis_NCX1 FLGVSIIADRFMSSIEVITSQEKEITIK.KPNGETTKTT................VRIWNETVSNLTLMA
O.mossambicus_NCX1.1 FLGVSIIADRFMASIEVITSQERRITIK.KPNGEKITTT................VRIWNETVSNLTLMA
O.mykiss_NCX1 FLGVSIIADRFMASIEVITSQERRITIK.KPNGEKVTTT................VRIWNETVSNLTLMA
D.rerio_NCX1a FLGVSIIADRFMASIEVITSQEKEITIK.KPNGETTTTT................VRIWNETVSNLTLMA
H.sapiens_NCX3.3 FLGVSIIADRFMASIEVITSQEREVTIK.KPNGETSTTT................IRVWNETVSNLTLMA
R.norvegicus_NCX3.3 FLGVSIIADRFMASIEVITSQEREVTIK.KPNGETSTTT................IRVWNETVSNLTLMA
M.musculus_NCX3.1 FLGVSIIADRFMASIEVITSQEREVTIK.KPNGETSTTT................IRVWNETVSNLTLMA
G.gallus_NCX3 FLGVSIIADRFMASIEVITSQEKEITIK.KPNGETTTTT................IRIWNETVSNLTLMA
D.rerio_NCX4a FLGMSIIADRFMSSIEVITSQEKEITIK.KPNGETTTAT................VRIWNETVSNLTLMA
T.nigroviridis_NCX4a FLGMSIIADRFMSSIEVITSQEKEITIK.KPNGETTTTT................VRIWNETVSNLTLMA
H.sapiens_NCX2.1 FLGVSIIADRFMAAIEVITSKEKEITIT.KANGETSVGT................VRIWNETVSNLTLMA
R.norvegicus_NCX2.1 FLGLSIIADRFMASIEVITSKEKEITIT.KANGETSVGT................VRIWNETVSNLTLMA
M.musculus_NCX2.1 FLGVSIIADRFMASIEVITSKEKEITIT.KANGETSVGT................VRIWNETVSNLTLMA
S.kowalevskii_3 FLGVSIVADRFMAAIEVITSKEKEVTVK.RPNGEKVVVS................VRIWNETVSNLTLMA
T.adhaerens_2 FIGVSIISDRFMSAIEIITSKERFIKVK.MSNGEYKRIS................VRVWNETVSNLTLMA
L.opalescens_NCX FLGVSIIADRFMAAIEVITSKEKDVVVK.KPDGTTTVVN................VRIWNETVSNLTLMA
A.mellifera_NCX FIGVSIISDRFMAAIEVITSKEKELVVR.RQGKEPQIVV................VRVWNETVANLTLMA
D.melanogaster_NCX FVGVSIIADRFMAAIEAITSIERAVVVK.GPNNTKQVMH................VRIWNETVANLTLMA
H.magnipapillata FLGISIISDIFMSSIEIITSKEKEVLVRDRVTGKKHSVT................VKIWNETVANLTLMA
C.elegans_NCX1 FFGVSIVADRFMASIEVITSQQKSVKMK.KITGEHFTIM................VRVWNETVSNLTLMA
C.briggsae_NCX1 FFGVSIVADRFMASIEVITSQTKSVKMK.KITGEPFTVL................VRVWNETVSNLTLMA
C.elegans_NCX2 FLGISIVADRFMSSIEVITSMERTIVVK.RPGLDPMAVQ................VRIWNDTVSNLTLMA
C.briggsae_NCX2 FLGISIVADRFMSSIEVITSMERTIVVK.RPGLDPMEVQ................VRIWNDTVSNLTLMA
T.spiralis FVGVAILADSFMCAVERITSTKRTLKLQTAIDEETGSVLEQYQ...........EVLIWNPTVANLTLMA
M.brevicollis_1 FAGVGIITDVFMEAIEVITSRERHVMV.DGI......................ETTVLIWNPTIANLSLMA
Salpingoeca FSGIGIITDVFMEAIEVITSKEKEVKLKDGR......................VVHVQVWNPTIANLSLMA
S.kowalevskii_1 FMGVSIIADIFMCSIEVITSKTKTVNIANPKAPGGTEE...............IKVRVWNSTVANLTLMA
S.kowalevskii_2 LYGVSIITGIFMSSIEVITSKTKTVNIANPKAPGGTQK...............LKVRVWNSTVANLTLVT
T.adhaerens_1 FLGVAIIADIFMCAIEVITSQTRRIKISDKDGNMKE.................VEVRIWNDTVANLTLMA
D.pulex_1 FTGVAIIADIFMGAIEKITSTTRKVFINHRSQEPEV.................IEVRIWNDTVANLTLMA
P.humanus_corporis FVGVSVVTDIFMGAVVTITSQTKKVYLGKFKKKPEESESALNVTATSTHQLDFIEVRVWNDTVANLTLMA
O.dioica FIGVAIVADIFMVSIEVITSRETHITILVDGQPEKQ..................KVRFWNATVANLTLMA
A.pisum LLGISIVADVFMTAIDKITSHTKKVYLAKELKPNGSDVSLRPD......QPEVIEVRIWNPTIANLTLMA
P.marinus FVGVAIISDFFMGAIEKITSKKKRARLTIQGETK..................LVTVRVWNDTVANLTLMA
A.anophagefferens_1 FLGVSIIADVFMAAIETITSKEKKVPGTD.........................VWNATVANLTLMA
E.huxleyi FLGVAIAADAFMLGIETITSQEQAKTVVVDGRKR..................VYSLKIWNATVANLTLMA
C.intestinalis FIGVAIGSDVFMTSIEVITSKKRTIIIWDEELGENTKK.................EVLVWNETVANLTLLA
B.floridae FIGITIGSDVFMCSIEVITSKKRKVVRWDEERQETVER.................EVLLWNETIANLTLLA
T.adhaerens_3 FVGVAIMSDVFMGAIEVITSKKKTVITYDRETGEQATK.................EVLIWNETVANLSLMA
N.vectensis_2 FLGIAIVADIFMSCIEVITSKKRKVTRYDHEKGESVEI.................EVFVWNETVANLTLMA
consensus>50 flGvs!iadrFM.s!evITSqekeitik.kpnge....................vriWNeT!sNLtLma

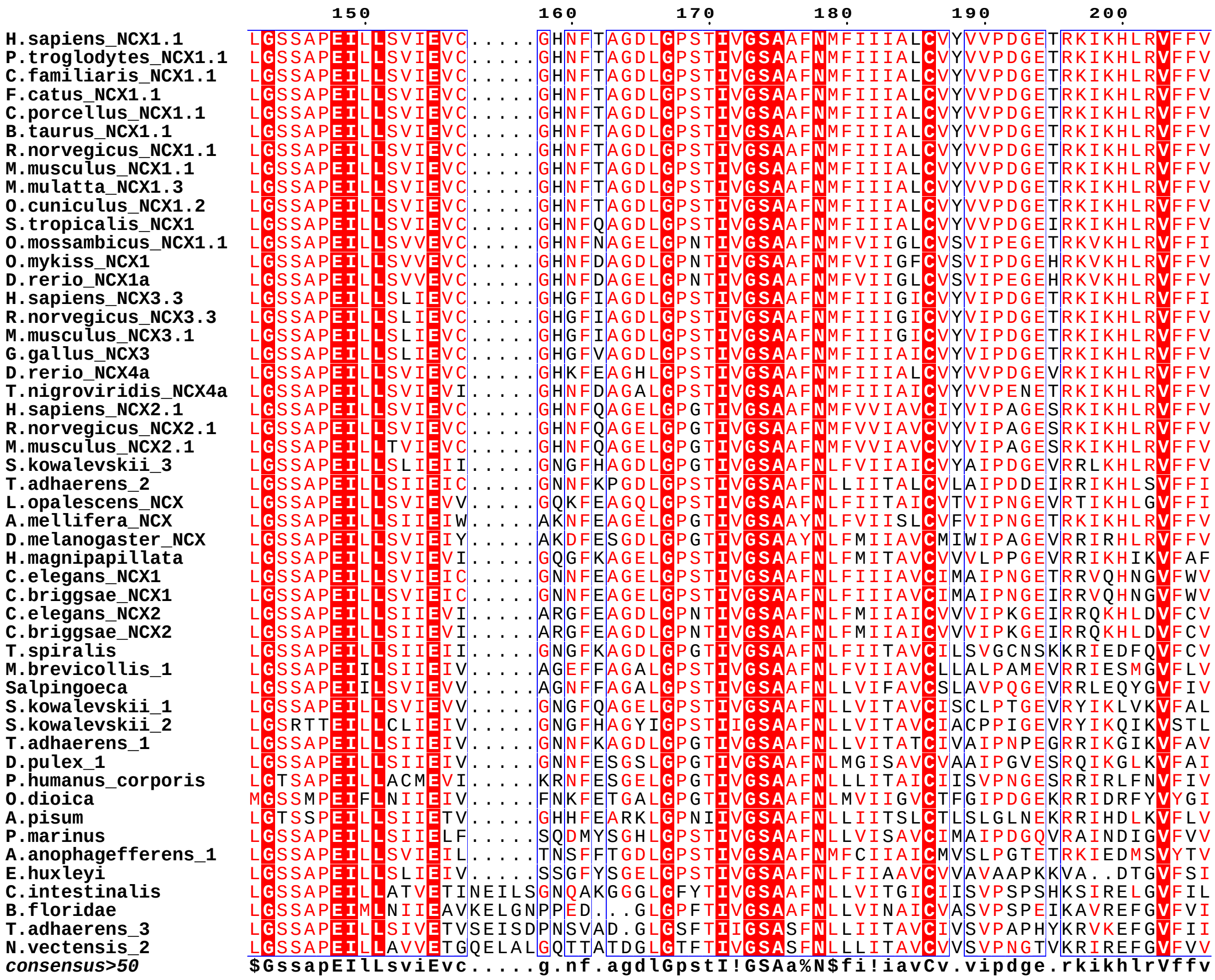
150 160 170 180 190 200
H.sapiens_NCX1.1 LGSSAPEILLSVIEVC.....GHNFTAGDLGPSTIVGSAAFNMFIIIALCVYVVPDGETRKIKHLRVFFV
P.troglodytes_NCX1.1 LGSSAPEILLSVIEVC.....GHNFTAGDLGPSTIVGSAAFNMFIIIALCVYVVPDGETRKIKHLRVFFV
C.familiaris_NCX1.1 LGSSAPEILLSVIEVC.....GHNFTAGDLGPSTIVGSAAFNMFIIIALCVYVVPDGETRKIKHLRVFFV
F.catus_NCX1.1 LGSSAPEILLSVIEVC.....GHNFTAGDLGPSTIVGSAAFNMFIIIALCVYVVPDGETRKIKHLRVFFV
C.porcellus_NCX1.1 LGSSAPEILLSVIEVC.....GHNFTAGDLGPSTIVGSAAFNMFIIIALCVYVVPDGETRKIKHLRVFFV
B.taurus_NCX1.1 LGSSAPEILLSVIEVC.....GHNFTAGDLGPSTIVGSAAFNMFIIIALCVYVVPDGETRKIKHLRVFFV
R.norvegicus_NCX1.1 LGSSAPEILLSVIEVC.....GHNFTAGDLGPSTIVGSAAFNMFIIIALCVYVVPDGETRKIKHLRVFFV
M.musculus_NCX1.1 LGSSAPEILLSVIEVC.....GHNFTAGDLGPSTIVGSAAFNMFIIIALCVYVVPDGETRKIKHLRVFFV
M.mulatta_NCX1.3 LGSSAPEILLSVIEVC.....GHNFTAGDLGPSTIVGSAAFNMFIIIALCVYVVPDGETRKIKHLRVFFV
O.cuniculus_NCX1.2 LGSSAPEILLSVIEVC.....GHNFTAGDLGPSTIVGSAAFNMFIIIALCVYVVPDGETRKIKHLRVFFV
S.tropicalis_NCX1 LGSSAPEILLSVIEVC.....GHNFQAGDLGPSTIVGSAAFNMFIIIALCVYVVPDGEIRKIKHLRVFFV
O.mossambicus_NCX1.1 LGSSAPEILLSVVEVC.....GHNFNAGELGPNTIVGSAAFNMFVIIGLCVSVIPEGETRKVKHLRVFFI
O.mykiss_NCX1 LGSSAPEILLSVIEVC.....GHNFDAGDLGPNTIVGSAAFNMFVIIGFCVSVIPDGEHRKVKHLRVFFV
D.rerio_NCX1a LGSSAPEILLSVVEVC.....GHNFDAGELGPNTIVGSAAFNMFVIIGLCVSVIPEGEHRKVKHLRVFFV
H.sapiens_NCX3.3 LGSSAPEILLSLIEVC.....GHGFIAGDLGPSTIVGSAAFNMFIIIGICVYVIPDGETRKIKHLRVFFI
R.norvegicus_NCX3.3 LGSSAPEILLSLIEVC.....GHGFIAGDLGPSTIVGSAAFNMFIIIGICVYVIPDGETRKIKHLRVFFV
M.musculus_NCX3.1 LGSSAPEILLSLIEVC.....GHGFIAGDLGPSTIVGSAAFNMFIIIGICVYVIPDGETRKIKHLRVFFV
G.gallus_NCX3 LGSSAPEILLSLIEVC.....GHGFVAGDLGPSTIVGSAAFNMFIIIAICVYVIPDGETRKIKHLRVFFV
D.rerio_NCX4a LGSSAPEILLSVIEVC.....GHKFEAGHLGPSTIVGSAAFNMFIIIALCVYVVPDGEVRKIKHLRVFFV
T.nigroviridis_NCX4a LGSSAPEILLSVIEVI.....GHNFDAGALGPSTIVGSAAFNMFIIIAICVYVVPENETRKIKHLRVFFV
H.sapiens_NCX2.1 LGSSAPEILLSVIEVC.....GHNFQAGELGPGTIVGSAAFNMFVVIAVCIYVIPAGESRKIKHLRVFFV
R.norvegicus_NCX2.1 LGSSAPEILLSVIEVC.....GHNFQAGELGPGTIVGSAAFNMFVVIAVCVYVIPAGESRKIKHLRVFFV
M.musculus_NCX2.1 LGSSAPEILLTVIEVC.....GHNFQAGELGPGTIVGSAAFNMFVVIAVCVYVIPAGESRKIKHLRVFFV
S.kowalevskii_3 LGSSAPEILLSLIEII.....GNGFHAGDLGPGTIVGSAAFNLFVIIAICVYAIPDGEVRRLKHLRVFFV
T.adhaerens_2 LGSSAPEILLSIIEIC.....GNNFKPGDLGPSTIVGSAAFNLLIITALCVLAIPDDEIRRIKHLSVFFI
L.opalescens_NCX LGSSAPEILLSVIEVV.....GQKFEAGQLGPSTIVGSAAFNLFIITAICVTVIPNGEVRTIKHLGVFFI
A.mellifera_NCX LGSSAPEILLSIIEIW.....AKNFEAGELGPGTIVGSAAYNLFVIISLCVFVIPNGETRKIKHLRVFFV
D.melanogaster_NCX LGSSAPEILLSVIEIY.....AKDFESGDLGPGTIVGSAAYNLFMIIAVCMIWIPAGEVRRIRHLRVFFV
H.magnipapillata LGSSAPEILLSVIEVI.....GQGFKAGELGPSTIVGSAAFNLFMITAVCVVVLPPGEVRRIKHIKVFAF
C.elegans_NCX1 LGSSAPEILLSVIEIC.....GNNFEAGELGPSTIVGSAAFNLFIIIAVCIMAIPNGETRRVQHNGVFWV
C.briggsae_NCX1 LGSSAPEILLSVIEIC.....GNNFEAGELGPSTIVGSAAFNLFIIIAVCIMAIPNGEIRRVQHNGVFWV
C.elegans_NCX2 LGSSAPEILLSIIEVI.....ARGFEAGDLGPNTIVGSAAFNLFMIIAICVVVIPKGEIRRQKHLDVFCV
C.briggsae_NCX2 LGSSAPEILLSIIEVI.....ARGFEAGDLGPNTIVGSAAFNLFMIIAICVVVIPKGEIRRQKHLDVFCV
T.spiralis LGSSAPEILLSIIEII.....GNGFKAGDLGPGTIVGSAAFNLFIITAVCILSVGCNSKKRIEDFQVFCV
M.brevicollis_1 LGSSAPEIILSIIEIV.....AGEFFAGALGPSTIVGSAAFNLFVIIAVCLLALPAMEVRRIESMGVFLV
Salpingoeca LGSSAPEIILSVIEVV.....AGNFFAGALGPSTIVGSAAFNLLVIFAVCSLAVPQGEVRRLEQYGVFIV
S.kowalevskii_1 LGSSAPEILLSVIEVV.....GNGFQAGELGPSTIVGSAAFNLLVITAVCISCLPTGEVRYIKLVKVFAL
S.kowalevskii_2 LGSRTTEILLCLIEIV.....GNGFHAGYIGPSTIIGSAAFNLLVITAVCIACPPIGEVRYIKQIKVSTL
T.adhaerens_1 LGSSAPEILLSIIEIV.....GNNFKAGDLGPGTIVGSAAFNLLVITATCIVAIPNPEGRRIKGIKVFAV
D.pulex_1 LGSSAPEILLSIIEIV.....GNNFESGSLGPGTIVGSAAFNLMGISAVCVAAIPGVESRQIKGLKVFAI
P.humanus_corporis LGTSAPEILLACMEVI.....KRNFESGELGPGTIVGSAAFNLLLITAICIISVPNGESRRIRLFNVFIV
O.dioica MGSSMPEIFLNIIEIV.....FNKFETGALGPGTIVGSAAFNLMVIIGVCTFGIPDGEKRRIDRFYVYGI
A.pisum LGTSSPEILLSIIETV.....GHHFEARKLGPNIIVGSAAFNLLIITSLCTLSLGLNEKRRIHDLKVFLV
P.marinus LGSSAPEILLSIIELF.....SQDMYSGHLGPSTIVGSAAFNLLVISAVCIMAIPDGQVRAINDIGVFVV
A.anophagefferens_1 LGSSAPEILLSVIEIL.....TNSFFTGDLGPSTIVGSAAFNMFCIIAICMVSLPGTETRKIEDMSVYTV
E.huxleyi LGSSAPEILLSLIEIV.....SSGFYSGELGPSTIVGSAAFNLFIIAAVCVVAVAAPKKVA..DTGVFSI
C.intestinalis LGSSAPEILLATVETINEILSGNQAKGGGLGFYTIVGSAAFNLLVITGICIISVPSPSHKSIRELGVFIL
B.floridae LGSSAPEIMLNIIEAVKELGNPPED...GLGPFTIVGSAAFNLLVINAICVASVPSPEIKAVREFGVFVI
T.adhaerens_3 LGSSAPEILLSIVETVSEISDPNSVAD.GLGSFTIIGSASFNLLIITAVCIVSVPAPHYKRVKEFGVFII
N.vectensis_2 LGSSAPEILLAVVETGQELALGQTTATDGLGTFTIVGSASFNLLLITAVCVVSVPNGTVKRIREFGVFVV
consensus>50 $GssapEIlLsviEvc.....g.nf.agdlGpstI!GSAa%N$fi!iavCv.vipdge.rkikhlrVffv

```
                          210       220       230       240       250       260       270
H.sapiens_NCX1.1         TAAWSIFAYTWLYIILSVISPGVVEVWEGLLTFFFFPICVVFAWVADRRLLFYKYVYKRYRAGKQRGMII
P.troglodytes_NCX1.1     TAAWSIFAYTWLYIILSVISPGVVEVWEGLLTFFFFPICVVFAWVADRRLLFYKYVYKRYRAGKQRGMII
C.familiaris_NCX1.1      TAAWSIFAYTWLYIILSVISPGVVEVWEGLLTFFFFPICVVFAWVADRRLLFYKYVYKRYRAGKQRGMII
F.catus_NCX1.1           TAAWSIFAYTWLYIILSVISPGVVEVWEGLLTFFFFPICVVFAWVADRRLLFYKYVYKRYRAGKQRGMII
C.porcellus_NCX1.1       TAAWSIFAYTWLYIILSVISPGVVEVWEGLLTFFFFPICVVFAWVADRRLLFYKYVYKRYRAGKQRGMII
B.taurus_NCX1.1          TAAWSIFAYTWLYIILSVSSPGVVEVWEGLLTFFFFPICVVFAWVADRRLLFYKYVYKRYRAGKQRGMII
R.norvegicus_NCX1.1      TAAWSIFAYTWLYIILSVSSPGVVEVWEGLLTFFFFPICVVFAWVADRRLLFYKYVYKRYRAGKQRGMII
M.musculus_NCX1.1        TAAWSIFAYTWLYIILSVSSPGVVEVWEGLLTFFFFPICVVFAWVADRRLLFYKYVYKRYRAGKQRGMII
M.mulatta_NCX1.3         TAAWSIFAYTWLYIILSVISPGVVEVWEGLLTFFFFPICVVFAWVADRRLLFYKYVYKRYRAGKQRGMII
O.cuniculus_NCX1.2       TAAWSIFAYTWLYIILSVISPGIVEVWEGLLTFFFFPICVVFAWVADRRLLFYKYVYKRYRAGKQRGMII
S.tropicalis_NCX1        TAAWSIFAYTWLYMILSVFSPGVVEVWEGLLTFFFFPICVVFAWVADRRLLFYKYVYKRYRAGKQRGMII
O.mossambicus_NCX1.1     TAAWSVFAYTWLYLILAVFSPGVVEIWEGLLTLFFFPLCVGLAYIADRRLLFYKYMYKRYRAGKRKGVII
O.mykiss_NCX1            TATWSIFAYTWLYLILAVISPGIVQVWEGLVTLFFFPLCVGMAYVADRRLLVYKYMYKRYRAGKRRGVII
D.rerio_NCX1a            TATWSIFAYTWLYLILAVISPGIVEVWEGLLTLFFFPICVLFAWVADRRLLFYKYVYKRYRVGKKRGIII
H.sapiens_NCX3.3         TAAWSIFAYIWLYMILAVFSPGVVQVWEGLLTLFFFPVCVLLAWVADKRLLFYKYMHKKYRTDKHRGIII
R.norvegicus_NCX3.3      TAAWSVFAYIWLYMILAVFSPGVVQVWEGLLTLFFFPVCVLLAWVADKRLLFYKYMHKRYRTDKHRGIII
M.musculus_NCX3.1        TAAWSIFAYIWLYMILAVFSPGVVQVWEGLLTLFFFPVCVLLAWVADKRLLFYKYMHKKYRTDKHRGIII
G.gallus_NCX3            TAAWSIFAYIWLYMILAVFSPGVVQVWEGLLTLFFFPLCVLLAWIADRRLLFYKYMHKKYRTDKHRGIII
D.rerio_NCX4a            TAAWSIFAYIWLYLILSVFSPGVVEVWEAVLTFLFFPLCVVQAWIADRRLLFYKYVHKRYRTDKNRGIII
T.nigroviridis_NCX4a     TAAWSIFAYIWLYLILSVFSPGEVQVWEAVLTFCFFPLCVLQAWIADRRLLFYKYVHKRYRADKTRGIII
H.sapiens_NCX2.1         TASWSIFAYVWLYLILAVFSPGVVQVWEALLTLVFFPVCVVFAWMADKRLLFYKYVYKRYRTDPRSGIII
R.norvegicus_NCX2.1      TASWSIFAYVWLYLILAVFSPGVVQVWEALLTLVFFPVCVVFAWMADKRLLFYKYVYKRYRTDPRSGIII
M.musculus_NCX2.1        TASWSIFAYVWLYLILAVFSPGVVQVWEALLTLIFFPVCVVFAWMADKRLLFYKYVYKRYRTDPRSGIII
S.kowalevskii_3          TATCSLFAYIWLYMILAVFSAGIIEVWEALLTFMFFPLLVVVAYLVDTRVFFMKFLRKKYKAKTMPGESR
T.adhaerens_2            TASCSVFAYLWLLLILKGISRDVIDIWEAVVTLLFFPILVTIAWVADRQLLSFKKLSKKYRADGKNRAVI
L.opalescens_NCX         TATWSVFAYLWLYFILAVSSYGVVDVWEGLLTFMFFPATVVTAYIADRRLLNKYLSKKYRASKQKGVIVQ
A.mellifera_NCX          TATWSIFAYVWLYLILAVSSPGVLEVWEGILTFSFFPATVLTAYVADRRLLIYKYLHKGYRMNKRGVIVQ
D.melanogaster_NCX       TALFSVFAYVWLWLILSVFTPGVILVWEAIVTLLFFPLTVLWAYIAERRLLVYKYMDKNYRVNKRGTVVA
H.magnipapillata         TASCSMFAYIWLYLILSVFSPGVIEIWEGIITFLCFPAMVIAAWAIDRKINFYHLLRKKIRKVKKRGQAF
C.elegans_NCX1           TVVWSTFAYVWLYLILSVFSPGEVEVWEGVLTFVFFPLTVGSAYFADAHAGQFGQRLISGPLSSFVRRSP
C.briggsae_NCX1          TVVWSTFAYVWLYLILSVFSPGEVEVWEGVLTFVFFPLTVASAYFADAHAGQFGQRLISGPLSSFVRRSP
C.elegans_NCX2           TATWSVFAYVWLYLILAFFSPGEIEIWEGALTFIFFPLTVFTAYMADIKLIQNKFLPHRYRRGSHGQMIA
C.briggsae_NCX2          TATWSIFAYVWLYLILAFFSPGEIEIWEGLLTFVFFPLTVGTAYMADIKIIQNRFLPHRYRRGSHGQMIA
T.spiralis               TTGWSVFAYIWLIIVLVLVSPNRIEVWEGLLTLLFFGALLLHAYLVSKKLCSGGRGRGRPTTERDRTPKA
M.brevicollis_1          TAIFSIFAYIWLLIILIASSPDVIEIWEGVVTLLFFPILLGLSLMADRGYFDFSRDQKPHPNEEELQVIN
Salpingoeca              TAIFSVFAYVWLIIILVVSSPNVVEVWEGVLTLLFFPMLLGLSFMADRGYFDFGKRHPASVDNVLR..ID
S.kowalevskii_1          TSFFCIFAYIWLFLVLVVSSPDVVELWEAIFTFLCFPILVILAFVADKDYCGKKEVEDAELARLGIGHPG
S.kowalevskii_2          TAAFSIIAYIWLLLVLLVITPNIVELWEAICTFICFPILVVIAFMADKGYRGRNVAKDDDKQAL......
T.adhaerens_1            TATFSIFAYLWLYIVLVARTKNIVDLTEAIITLCFFFVLVLISYFVDRNFFCPR..........KARRIS
D.pulex_1                TAVFSILAYLWLIFVLMGVSKNVVEVWEAVVTFLLFPILVYLAYLADKGFPWNKSRVSATTANGKQIELG
P.humanus_corporis       CSIFSILAYVWLWIILSLFSPHVVELWEAIATFLMFPALIFFAFATDKKWLDIRLLMSKKKRLTRAHEIK
O.dioica                 TAFFSIFAYVWLLIILQVTSPGVIDLWEALVTLLMFPLLVGVAYLADRNMLCGQTRTDNDDEGQKQIMEY
A.pisum                  IGAFSFFAYIWLLLIIEVISPNVIELWEAAATFLLFPILMFFAYATDQNWCGKST...............
P.marinus                TAGCSIFAYIWLLLVLQVSSPDVVDIWEAVVTLVLFPALVVM.....................AFAADKG
A.anophagefferens_1      TAFFSVFAYVWLLFILMVTSPDVVEIWEAIFTFLFFPILVAL.....................AYAADRG
E.huxleyi                TAAASVFAYVWLAYILLVSSPNVIEVWEGAATLAFFPLLVWLSHRCGIRVPPPTLSAQLRTMLAYLADVG
C.intestinalis           TSMWSLLAYVWLLCVMLWTSPNVVEPWEAWLTLIFFPLLVITAYAQDNGWWMYKLRGQNSVQDASKPRDE
B.floridae               TSLWSMFAYVWMLVVVLWVTPGVIDVWEAWVTLGFTPLLVLTAYAQDSGWWCKRQHSRIHNSEPSIQEDM
T.adhaerens_3            TAIWSVFAYVWMLIVTTVWTPNKIDIFEAWITLAFFPLLILTAWAQDNGWWWKRHSQILPNEDKSLTVRV
N.vectensis_2            TAVWSMFAYVWMLFVIQWNSPEVIELWEAFMTLAFFPLLVLTAWCTDHGWWCRNRGKILSVDPPQVIVII
consensus>50             ta.wsifAYvW$yl!l.v.spgvvevwEgllTf.ffpi.v..awvadrrll.yky..k.yr.....g.ii
```

```
                         280
H.sapiens_NCX1.1         EHEGDRPS.S............................................................
P.troglodytes_NCX1.1     EHEGDRPS.S............................................................
C.familiaris_NCX1.1      EHEGDRPS.S............................................................
F.catus_NCX1.1           EHEGDRPS.S............................................................
C.porcellus_NCX1.1       EHEGDRPS.S............................................................
B.taurus_NCX1.1          EHEGDRPS.S............................................................
R.norvegicus_NCX1.1      EHEGDRPA.S............................................................
M.musculus_NCX1.1        EHEGDRPA.S............................................................
M.mulatta_NCX1.3         EHEGDRPS.S............................................................
O.cuniculus_NCX1.2       EHEGDRPS.S............................................................
S.tropicalis_NCX1        ETEGDRPS.S............................................................
O.mossambicus_NCX1.1     ETEGEPELPS............................................................
O.mykiss_NCX1            ETEGEAQIPS............................................................
D.rerio_NCX1a            ETEGEPELQS............................................................
H.sapiens_NCX3.3         ETEGDHPKG.............................................................
R.norvegicus_NCX3.3      ETEGEHPKG.............................................................
M.musculus_NCX3.1        ETEGDHPKG.............................................................
G.gallus_NCX3            ESEGDHPKG.............................................................
D.rerio_NCX4a            ETEGDGMFT.............................................................
T.nigroviridis_NCX4a     ESEGDAMFT.............................................................
H.sapiens_NCX2.1         GAEGDPPKSI............................................................
R.norvegicus_NCX2.1      GAEGDPPKSI............................................................
M.musculus_NCX2.1        GAEGDPPKSI............................................................
S.kowalevskii_3          GNHYP.................................................................
T.adhaerens_2            VEPDSI................................................................
L.opalescens_NCX         CEGQDAEAGE............................................................
A.mellifera_NCX          AEAGDSDGGV............................................................
D.melanogaster_NCX       GEHDQVEMDA............................................................
H.magnipapillata         IQTGDGDVVA............................................................
C.elegans_NCX1           R.....................................................................
C.briggsae_NCX1          R.....................................................................
C.elegans_NCX2           T.....................................................................
C.briggsae_NCX2          T.....................................................................
T.spiralis               LRELNRRSWT............................................................
M.brevicollis_1          IRT...................................................................
Salpingoeca              NDV...................................................................
S.kowalevskii_1          DRY...................................................................
S.kowalevskii_2          ......................................................................
T.adhaerens_1            DTE...................................................................
D.pulex_1                SIQ...................................................................
P.humanus_corporis       MEE...................................................................
O.dioica                 NPD...................................................................
A.pisum                  ......................................................................
P.marinus                MFS...................................................................
A.anophagefferens_1      YFD...................................................................
E.huxleyi                AFS...................................................................
C.intestinalis           DPAMVNGLSSRRGSAVGVKSPEKSRKMNWTNEGVCSVSNGDICGGTPNMGSPALKRHIRRVSIAAPTNPA
B.floridae               NVRVIGSPQGRASVLNTR...................................................A
T.adhaerens_3            VGGKSRHPSASIIRSTSRELSNVHRESVASAHNSTYDLESVTKLNPGENRSRANSARTSIS.........
N.vectensis_2            FTKGLVDRNSLALLRFVRFVKQARNCDVVSKTSRFSHLQVRVLGFTDAPSSHAMIHRSMEL.........
consensus>50             e.egd.................................................................
```

```
                                            290          300       310                320          330
                                              .            .         .                  .            .
H.sapiens_NCX1.1        ..................KTEIEMDGKVVNSHVENFLDGALVLEVDERDQD.......DEEARREMARIL
P.troglodytes_NCX1.1    ..................KTEIEMDGKVVNSHVENFLDGALVLEVDERDQD.......DEEARREMARIL
C.familiaris_NCX1.1     ..................KTEIEMDGKVVNSHVDNFLDGALVLEVDERDQD.......DEEARREMARIL
F.catus_NCX1.1          ..................KTEIEMDGKVVNSHVDNFLDGALVLEVDERDQD.......DEEARREMARIL
C.porcellus_NCX1.1      ..................KTEIEMDGKVVNSHVENFLDGALVLEVDERDQD.......DEEARREMARIL
B.taurus_NCX1.1         ..................KTEIEMDGKVVNSHVDSFLDGALVLEVDERDQD.......DEEARREMARIL
R.norvegicus_NCX1.1     ..................KTEIEMDGKVVNSHVDNFLDGALVLEVDERDQD.......DEEARREMARIL
M.musculus_NCX1.1       ..................KTEIEMDGKVVNSHVDNFLDGALVLEVDERDQD.......DEEARREMARIL
M.mulatta_NCX1.3        ..................KTEIEMDGKVVNSHVENFLDGALVLEVDERDQD.......DEEARREMARIL
O.cuniculus_NCX1.2      ..................KTEIEMDGKVVNSHVDNFLDGALVLDVDERDQD.......DEEARREMARIL
S.tropicalis_NCX1       ..................KADIEMDGKVLNSHTETFLDGSLVLEVDEKDQD.......EEEARRDMAKIL
O.mossambicus_NCX1.1    ..................KVDIEMDGKMLNSHGEEFTEELE..G...KELD.......EEEARREVARIL
O.mykiss_NCX1           ..................KMDIEMDGKMLNS..ESFMDGAM..GFDEKDLD.......EEEARREMVRIL
D.rerio_NCX1a           ..................KADIEMDGGMLNSHAEEFLDGAV..DNEDKDAD.......EDEARREMAKIL
H.sapiens_NCX3.3        .....................IEMDGKMMNSHFLDGNLVPLEGKEVD.............ESRREMIRIL
R.norvegicus_NCX3.3     .....................IEMDGKMMNSHFLDGNLIPLEGKEVD.............ESRREMIRIL
M.musculus_NCX3.1       .....................IEMDGKMMNSHFLDGNFTPLEGKEVD.............ESRREMIRIL
G.gallus_NCX3           .....................IEMDGKMMNSHFLDGNLVTVEGKEVD.............ESRKEMIRIL
D.rerio_NCX4a           ..................KMDMEMDGQGANSHH..KEALDGMLAGVEEGGGGGEE....EEARREMARTL
T.nigroviridis_NCX4a    ..................KMDMEMDGQGVNSHTHPKEALDGMLEGMEEGGGMISEQDQEEEARREMARTL
H.sapiens_NCX2.1        ..................ELDGTFVGAEAPGELGGLGPGPAEARELDA.............SRREVIQIL
R.norvegicus_NCX2.1     ..................ELDGTFVGTEVPGELGALGTGPAEARELDA.............SRREVIQIL
M.musculus_NCX2.1       ..................ELDGTFVGTEVPGELGALGTGPAEARELDA.............SRREVIQIL
S.kowalevskii_3         ................................LSHEYDDVEYRGRFEDDELDFEDIKDLDESRQEAIRIL
T.adhaerens_2           .....................IMRSPKEERDSEGFNSVYKNNPMYDVDSIDDVDSEKNDEMRRKKAMQIV
L.opalescens_NCX        ..................GKSEDG.....ALKEGGDDVEVREF................EQHRKEYIEIL
A.mellifera_NCX         ..................ELEIKPQQDSFHNMMDADTPEAKEF................EQTRRDYINTL
D.melanogaster_NCX      ..................EKGPKQPMVTSARGNDAEA.....F................DEARREYITLL
H.magnipapillata        ..................VSLKSTIKHDSSNDNVDEKDIELLPLEEDDDPLEIINDKK.....KKAMEAF
C.elegans_NCX1          ......................RSPSKKTRENVENGA..GLPGDATQNLIGGDADALAFEIHRRHYLDIF
C.briggsae_NCX1         ......................RSPSKKTHENALESGNGGIPGDQNQSLIGGDADALAFEIHRRHYLDIF
C.elegans_NCX2          ......................EAEEMKMLENGTQG................DPALKAFEEHRQEFIELM
C.briggsae_NCX2         ......................EAEEMKMLENGSPQ..............GADPALKAFEEHRQEFIELM
T.spiralis              ..................GPALNGKEKLLDVVEQNRVD.............................RLS
M.brevicollis_1         ..............................EDGRNIAFDPYDM.........................AV
Salpingoeca             ..............................EHGHQFHDPYEAD.........................TI
S.kowalevskii_1         ..............................LTAENVTKEGVADVM............KEIGKHPDITPEQ
S.kowalevskii_2         ..............................LGIGNVTREGVADVM............KEMGKHPDITQEQ
T.adhaerens_1           ..............................NGHLAFDDSTGRLVPDSFDKSSANSFLKAVKTRTDITAEE
D.pulex_1               ..............................PGELEAMVDGLTLTKDGQVDPNELAKWVRQASRMGLTGED
P.humanus_corporis      ..............................SKKIFVERKFIKDGKI..DKETLYSFFKELKKYPGISNED
O.dioica                ..............................TPEGIELLGKQRPNSMALVDEFMKDFEGDPLTIDEQAAAI
A.pisum                 ...................................EHNTHQLDLDLGQNKENLIKFVKNVKKYSGITDAD
P.marinus               ..............................IKTNVASAHRDHIVGLEDLSPDELVD............LM
A.anophagefferens_1     ..............................TAKISPVSSAHVIQVGSTPFRPYEFS............DL
E.huxleyi               ..............................CPDKARPATHVVVAFTRDGKPIRHRSFAREAATQWARGDL
C.intestinalis          AIELASISKKDNGRKESERRQVEPEPAYRAFARARFRHAAIWSIGGRKRKPINLNLQPSPKLPVNDPKLL
B.floridae              PTELLALEGRNRNLNDMDRPSQQGQQPTNALSRARFRHAVVRSMLYQK............KAPKPKARFA
T.adhaerens_3           .........RSSFVYEDHGKIQVDIKPSNSFARARFRHAAIRSLMGGKRHRRSKSVTDKMVELESGLFNS
N.vectensis_2           ..................RALEQHRGSVRNSSFDRFRHAALRSVTRSKKLIKINEGEDVE..........
consensus>50            ......................e.d.........n...........e............ee.rre...il
```

```
                                340       350       360       370       380                 390
                                  .         .         .         .         .                   .
H.sapiens_NCX1.1         KELKQKHPDKEIEQLIELANYQVLSQQQKSRAFYRIQATRLMTGAGNILKRHAADQA......RKAVSMH
P.troglodytes_NCX1.1     KELKQKHPDKEIEQLIELANYQVLSQQQKSRAFYRIQATRLMTGAGNILKRHAADQA......RKAVSMH
C.familiaris_NCX1.1      KELKQKHPEKEIEQLIELANYQVLSQQQKSRAFYRIQATRLMTGAGNILKRHAADQA......RKAVSMH
F.catus_NCX1.1           KELKQKHPEKEIEQLIELANYQVLSQQQKSRAFYRIQATRLMTGAGNILKRHAADQA......RKAVSMH
C.porcellus_NCX1.1       KELKQKHPEKEIEQLIELANYQVLSQQQKSRAFYRIQATRLMTGAGNILKRHAADQA......RKAVSMH
B.taurus_NCX1.1          KELKQKHPEKEIEQLIELANYQVLSQQQKSRAFYRIQATRLMTGAGNILKRHAADQA......RKAVSMH
R.norvegicus_NCX1.1      KELKQKHPDKEIEQLIELANYQVLSQQQKSRAFYRIQATRLMTGAGNILKRHAADQA......RKAVSMH
M.musculus_NCX1.1        KELKQKHPEKEIEQLIELANYQVLSQQQKSRAFYRIQATRLMTGAGNILKRHAADQA......RKAVSMH
M.mulatta_NCX1.3         KELKQKHPEKEIEQLIELANYQVLSQQQKSRAFYRIQATRLMTGAGNILKRHAADQA......RKAVSMH
O.cuniculus_NCX1.2       KELKQKHPEKEIEQLIELANYQVLSQQQKSRAFYRIQATRLMTGAGNILKRHAADQA......RKAVSMH
S.tropicalis_NCX1        KELKQKHPEKEIEQLIELANYQVLSQQQKSRAFYRIQATRLMTGAGNILKRHAADQA......RKAVSMH
O.mossambicus_NCX1.1     KELKQKHPEKEMEQLMELANYQVLTQQQKSRAFYRCQATRIMTGAGNVLKKHACDQA......KRAT..H
O.mykiss_NCX1            KELKQKHPEKETEQLIELANYQVLTQQQKSRAFYRCQATRIMTGAGNVLKKHAADQA......RKAVGAY
D.rerio_NCX1a            KELKQKHPEKEMEQLVELANYHVLSQQQKSRAFYRCQATRLMTGAGNILKKHAADQA......RKALGIH
H.sapiens_NCX3.3         KDLKQKHPEKDLDQLVEMANYYALSHQQKSRAFYRIQATRMMTGAGNILKKHAAEQA......KKASSMS
R.norvegicus_NCX3.3      KDLKQKHPEKDLDQLVEMANYYALSHQQKSRAFYRIQATRMMTGAGNILKKHAAEQA......KKTASMS
M.musculus_NCX3.1        KDLKQKHPEKDLDQLVEMANYYALSHQQKSRAFYRIQATRMMTGAGNILKKHAAEQA......KKTSSMS
G.gallus_NCX3            KDLKQKHPEKDLDQLVEMANYYALSHQQKSRAFYRIQATRMMTGAGNILKKHAAEQA......KKSTSLH
D.rerio_NCX4a            KELKQRHPEKDMEQLIEMANYQVLVQQQKSRAFYRIQATRMMIGAGNILKKHAADQA......RKVVSCH
T.nigroviridis_NCX4a     KELKQRHPEKDIEQLIEMANYQVLIQQQKSRAFYRIQATRMMIGAGNILKKHAADQA......RKVVSCH
H.sapiens_NCX2.1         KDLKQKHPDKDLEQLVGIANYYALLHQQKSRAFYRIQATRLMTGAGNVLRRHAADAS......RRAAPAE
R.norvegicus_NCX2.1      KDLKQKHPDKDLEQLVGIAKYYALLHQQKSRAFYRIQATRLMTGAGNVLRRHAADAA......RRPGAND
M.musculus_NCX2.1        KDLKQKHPDKDLEQLMGIAKYYALLHQQKSRAFYRIQATRLMTGAGNVLRRHAADAA......RRPGATD
S.kowalevskii_3          RDLKKKHPEADHETLEQMANYEALNRQTKSRAFYRIQATRKMVGAGNILKKNLD.........KKRTSIA
T.adhaerens_2            KEIKQNHPNATAKEIEELASYEALKYQPKSRAYYRIQATRKLTGSGNVIKQRKM..S......LPANPDG
L.opalescens_NCX         REMRKKNPTLDMKTLEDMAESEAVNRGPKSRAFYRIQATRKLTGSGNIIKKAKAQAGVAQPIVIDQKPED
A.mellifera_NCX          RELRKKYPALPLEQLEVMAHEEVLGKGPKSRAFYRVQATRKMVGAGNLSKKISERAQSDLSEVKAELQKQ
D.melanogaster_NCX       TELRQKYPDADLEQLEMMAQEQVLARSGRSRAFYRIQATRKMVGSGNLMRKIQERAHSDLTGVKAQLHAG
H.magnipapillata         HRARTKYPNADSITLKKLIEQENIKLQHKSRAFYRVQATHALTGQGNLLRLKDRDNSIELN.....NKME
C.elegans_NCX1           KQLRSEHPDAPVVELEKHAMEKVVGEQKKSRAFYRIQTTRKMIGSGDIQKKLK.KSN......KLEPMVV
C.briggsae_NCX1          KQLRSEHPDAPVDELEKHAMEKVVGEQKKSRAFYRIQTTRKMIGSGDIQKKMKNKKG......LLEPLVV
C.elegans_NCX2           REIRKQNPHITPTELQKQAEYEMISRGPKSRAFYRVQATRRLIGGGDIVKKRIDKEH......NKALDA.
C.briggsae_NCX2          REIRKQNPHITPTELQKQAEYEMISRGPKSRAFYRVQATRRLIGGGDIVKKRIDKEH......NKALDAL
T.spiralis               RELRRKYPHLTDDSLAEVVAKQIEKEAPHDHLWYRIMAVRRLASSMRSRVRAKPVSEQVCQKLETLSRTG
M.brevicollis_1          VLKDVETEALTEDEIANIAAARKIDGTKVSRARFRIDANRRLRGAAPLLPDWQRALLD.....TQKSRAI
Salpingoeca              IKRIARSNSMTMEDKVNLALAKKLKGERVSRAQFRIDATRKLVGSGGILPDYNKAKLQ.....EALLR..
S.kowalevskii_1          AATIAAIEAEKNSPH..............TKAWYRVNATRNMTGGQKLIPAVDPKLLSAYDQAKMGSAIS
S.kowalevskii_2          AATIAAIEAEKNSPH..............TRAWYRVSETRNMTGGQKLIPTVDPKLLLAYDQVKMGSSIS
T.adhaerens_1            AAKLAAAKLQEQEKK..............SYIWYRVGAARLVSGSKRVTPLLDARLQKIFDDIEMKNKKF
D.pulex_1                AAKLAAYKLITSKEH..............SRAWYRIGATRIFSGSKKIQPQLSIKLREVYDAINEHPDAV
P.humanus_corporis       AAILAASKIIDSEPQ..............SAATYRMSAIRDLTGGRKLRPRLSLKLQEVYDTINQYPLGS
O.dioica                 AVASKMQGSFCVVPSFHVTKNLQCPTQGRSRAWYRVQATRTLTAGARIEPN...........KALSRMA
A.pisum                  AAKLAATKLIDSTH...............RGAMWYRIGAVRRFTGGKQIEPVLEDHLRQ...........
P.marinus                AEVTRRRRLQERCPSQIRQRHGSSLTDDAVMRIIQAERPPRTTRAQHRIQASRMLTRRSGASKRSSVWNS
A.anophagefferens_1      LKKLQDPTLEESERHDLVKKLAMGQKAKPS.............RAVLRMNAVRAATGQKVIKADGPDPKV
E.huxleyi                ARELQRMDPDAADGTSDAQGLASYLASSLGTLRSRAAYRVATTRGATGVAAPQARAAAEQEVAPPPSAAV
C.intestinalis           SVVSKIASIRKLYSNNNAIQDES...SGN.VAFASSAYSTLESAGELTIDVLLHRRKRPKG.........
B.floridae               EVVTKMVSIKALTSPAMRRQLAANDMSGK.FTFGTHTYSVLESSGAIEVDVLFHRRRLSKL.........
T.adhaerens_3            SEPKMAHFVNIITHRLNEQSNASSRNIAATFVFEIAAYSVREDSGSIDLIVLLNRHRALIKKSQENKNNK
N.vectensis_2            ..........................DDNLNRFVFGTSSYSVLKSAKHIDLEIILLQN.............
consensus>50             kelkqkhpe.e.eql.elanyq.l..q.ksrafyriqatrlmtgagnilk...ad...............
```

```
                          400       410       420       430       440       450
H.sapiens_NCX1.1        EVNTEVTENDPVSK............IFFEQGTYQCLENCGTVALTIIRRGGDL..TNTVFVDFRTEDGT
P.troglodytes_NCX1.1    EVNTEVTENDPVSK............IFFEQGTYQCLENCGTVALTIIRRGGDL..TNTVFVDFRTEDGT
C.familiaris_NCX1.1     EVNTEVAENDPVSK............IFFEQGTYQCLENCGTVALTIIRRGGDL..TNTVFVDFRTEDGT
F.catus_NCX1.1          EVNTEVAENDPVSK............IFFEQGTYQCLENCGTVALTILRRGGDL..TNTVFVDFRTEDGT
C.porcellus_NCX1.1      EVNTEVAENDPVSK............IFFEQGTYQCLENCGTVALTIIRRGGDL..TNTVFVDFRTEDGT
B.taurus_NCX1.1         EVNTEVAENDPVSK............IFFEQGTYQCLENCGTVALTIIRRGGDL..TNTVFVDFRTEDGT
R.norvegicus_NCX1.1     EVNMDVVENDPVSK............VFFEQGTYQCLENCGTVALTIIRRGGDL..TNTVFVDFRTEDGT
M.musculus_NCX1.1       EVNMEMAENDPVSK............IFFEQGTYQCLENCGTVALTIMRRGGDL..STTVFVDFRTEDGT
M.mulatta_NCX1.3        EVNTEVTENDPVSK............IFFEQGTYQCLENCGTVALTIIRRGGDL..TNTVFVDFRTEDGT
O.cuniculus_NCX1.2      EVNTEMAENDPVSK............IFFEQGTYQCLENCGTVALTIIRRGGDL..TNTVFVDFRTEDGT
S.tropicalis_NCX1       EVNTDVVENDPVSK............IYFEQATYQCLENCGTVALTIVRRGGDL..TNTVYVDFRTEDGS
O.mossambicus_NCX1.1    DIFSEVSVNDFSSK............VFFDPGTYQCLENCGSVALNVVRRGGDL..TSTVSVDYRTEDGT
O.mykiss_NCX1           EIRSEVSENDFSSK............VFFDPGTYQCLENCGTVALNVVRLGGDL..TNTVSVEYRTEDGT
D.rerio_NCX1a           ELRSEVSDNDISSK............IFFDPGTYQCLENCGTVALNVVRRGGDL..TSTVSVEYRTEDGT
H.sapiens_NCX3.3        EVHTDEPE.DFISK............VFFDPCSYQCLENCGAVLLTVVRKGGDM..SKTMYVDYKTEDGS
R.norvegicus_NCX3.3     EVHTDEPE.DFASK............VFFDPCSYQCLENCGAVLLTVVRKGGDI..SKTMYVDYKTEDGS
M.musculus_NCX3.1       EVHTDEPE.DFASK............VFFDPCSYQCLENCGAVLLTVVRKGGDI..SKTMYVDYKTEDGS
G.gallus_NCX3           EVRPDEPE.EFISK............IYFDPCSYQCLENCGAVLLTVVRKGGDV..SKTIYVDYKTEDGS
D.rerio_NCX4a           EANPQE.EDPHTIY............LEFEPSHYQCFENCGSLKLSVTRHGGDS..GCTVKVDYRTEDGT
T.nigroviridis_NCX4a    EASGQE.EDPHTIY............LQFEPSHYQCFENCGSLKLSVSRYGGES..GCTVKVDYRTEDAT
H.sapiens_NCX2.1        GAGED..EDDGASR............IFFEPSLYHCLENCGSVLLSVTCQGGEG..NSTFYVDYRTEDGS
R.norvegicus_NCX2.1     GAPDD..EDDGASR............IFFEPSLYHCLENCGSVLLSVACQGGEG..NSTFYVDYRTEDGS
M.musculus_NCX2.1       GAPDD..EDDGASR............IFFEPSLYHCLENCGSVLLSVACQGGEG..NSTFYVDYRTEDGS
S.kowalevskii_3         EVKIEVPQDQNFTR............VFFEPAEYTVFENVGTFNATVVRRGGDM..NSTIYVDYRSEDGT
T.adhaerens_2           NVDPEDIEKCSLTQ............IYFSPEKYTVLENCGTVGVTVERTGN.L..NNVLTVDYKTQDGT
L.opalescens_NCX        EIT..........R............VSFDPGHYTVMENVGTFYGTVTREGGDL..TKTLYVDYKTEDGT
A.mellifera_NCX         EAESIEIENVNAMR............IFFEPGHYTVMENVGSFEVGVTRAGGDL..SKPCTVDYCTEDGS
D.melanogaster_NCX      DDE....EADDPIR............MYFEPGHYTVMENCGEFEVRVVRRG.DI..STYASVEYETQDGT
H.magnipapillata        EVSASQKDTVLENC............VQFEPDQYTVVESCGQCFLTVKRFGNDL..SDTLYVDYETSDGT
C.elegans_NCX1          QKTMATVEFD....................PPHYTCLENVGDVYLTVKCDRGSVPEDTTVTVHYRTIADT
C.briggsae_NCX1         KQTMATVEFD....................PPHYTCLENVGDVYLTVKCDRGNVPEDTTVTVHYRTIADT
C.elegans_NCX2          ..AQEKQSRDNTCK............IFLDPAHYTVLESVGSFDVVVGRDGG..PDGLTVMVDYFTEDGS
C.briggsae_NCX2         VQAQEKQLRDNTCK............IFLDPAHYTVLESVGSFDVVVGRDGG..PDGLTVMVDYFTEDGT
T.spiralis              MLRRTQTETGVRSV............TNVEFAANAYAVDPTGERRVRLKVVRRGSARKQLVFNYRTMSGS
M.brevicollis_1         KDSYYAHRATALRQEQTFGFMTIEYHVREDAGA............VTLTIVRTGDLSQSASIGIETQEGT
Salpingoeca             .DDFFEAHKTKLAKKNFVGLESLQYSIGEGDGH............VNVKILRTGDLSLPLTIEYYTEGDT
S.kowalevskii_1         VGSTLISPE.....IPIVEFAASSCAVYENEKR............VRVYVNRRGNIQPQVIFKFETLDGT
S.kowalevskii_2         IGSTLF.PE.....IPIVEFAAASCAVYEHEKR............VRVWINRKGNIHPQIVFKFETLDGT
T.adhaerens_1           SSTADVRDDPETTTRTTIEFTTAACAVMENEKM............VKVGISRTGKLNNTIKVRYETLPGT
D.pulex_1               NLSPLIETVPQNKAI..IEFHAIACAVPESVGR............FPITIVRSGKTDNSVSVRVETIDGS
P.humanus_corporis      ALSLLPEISMKNAIV...EFHSATCAVRENIGS............FFITVCRHGNLEPSVKVRVESVDGS
O.dioica                RSFSNLPNKNNTSDYAMFNFACTSSSVMENAKQ............IELKVMRTGNIMMPATCSYETIDGT
A.pisum                 ...PPTLSQNDETKYAIIEFHAPTIAVNENIGR............FPVTIWRHGNLNTKATVRVDSINGT
P.marinus               LPAHLVALKDTVEADEEAVRSESTIPVINFKCSRFAVMEGAGRIVLPLVTSRPLERDVVVRYETLQGTAT
A.anophagefferens_1     LAKYLEGAAGPKHRPEDVVAFFSDVNGKINTKYALLESDKAVTLHVMRSPAEGPMTIKWATRDGTAKGSE
E.huxleyi               LSFAVSGAAFSEGAKE........................ASLVVERSGEELARELDWEGSVEYESRDGT
C.intestinalis          ............QTVIPTTNANGKFDTQIMPGVI..........................SVDYETREGT
B.floridae              ............TLLNSMSKLNLVNKSNNGPNNGPVVNGTTTDSGQSSAPSKQDEAKEDVSVEFETRDGT
T.adhaerens_3           TVTFGLGKSTSETIIKSLSSSFKTDKSIKSCDPITEESIQDLESSRYSDEEGDDATQNNFSVDFETRDGG
N.vectensis_2           ...................NNNNKNKEMIYAHNDSIADTISENSFEDGVTPSSPGSSQRFSVDYETHDGS
consensus>50            e...e..end................iffe...y.clencg...l.vvr.ggd.....tv.vdy.tedgt
```

```
                                   460       470       480       490       500       510
H.sapiens_NCX1.1        ANAGSDYEFTEGTVVFKPGDTQKEIRVGIIDDDIFEEDENFLVHLSNVKVSSEASEDGILEA........
P.troglodytes_NCX1.1    ANAGSDYEFTEGTVVFKPGETQKEIRVGIIDDDIFEEDENFLVHLSNVKVSSEASEDGILEA........
C.familiaris_NCX1.1     ANAGSDYEFTEGTVVFKPGETQKEIRVGIIDDDIFEEDENFLVHLSNVKVSSEASEDGILEA........
F.catus_NCX1.1          ANAGSDYEFTEGTVVFKPGETQKEIRVGIIDDDIFEEDENFLVHLSNVKVSSEASEDGILEA........
C.porcellus_NCX1.1      ANAGSDYEFTEGTVVFKPGETQKEIRVGIIDDDIFEEDENFLVHLSNVKVSSEASEDGILEA........
B.taurus_NCX1.1         ANAGSDYEFTEGTVVFKPGETQKEIRVGIIDDDIFEEDENFLVHLSNVKVSLEASEDGILEA........
R.norvegicus_NCX1.1     ANAGSDYEFTEGTVIFKPGETQKEIRVGIIDDDIFEEDENFLVHLSNVRVSSEVSEDGILDS........
M.musculus_NCX1.1       ANAASDYEFTEGTVIFKPGETQKEIRVGIIDDDIFEEDENFLVHLSNVRVSSDVSEDGILES........
M.mulatta_NCX1.3        ANAGSDYEFTEGTVVFKPGETQKEIRVGIIDDDIFEEDENFLVHLSNVKVSSEASEDGILEA........
O.cuniculus_NCX1.2      ANAGSDYEFTEGTVVFKPGETQKEIRVGIIDDDIFEEDENFLVHLSNVKVSSETSEDGILEA........
S.tropicalis_NCX1       ANAGSDYEFTEGTIIFKPGETQKELRVGIIDDDIFEEDENFLVHLSNVRVNAENTEAN.LES........
O.mossambicus_NCX1.1    ANASSDYEFTEGTVVFKPGETEKEIRINIIDDDIFEEDEHFLVHLSNVRVRAEGSNSHE..A........
O.mykiss_NCX1           ANAGSDYQFTEGVVVFNPGETEKEIRIDIIDDDIFEEDEHFLVHLSNVKVISEGTGYVQPRA........
D.rerio_NCX1a           ANAGSDYQFTEGVIIFKPGETEKEIRVDIIDDDIFEEDEHFLVHLSNVKVISEGANNGNPGT........
H.sapiens_NCX3.3        ANAGADYEFTEGTVVLKPGETQKEFSVGIIDDDIFEEDEHFFVRLSNVRIEEEQPEEGMPPA........
R.norvegicus_NCX3.3     ANAGADYEFTEGTVVLKPGETQKEFSVGIIDDDIFEEDEHFFVRLSNVRVEEEQLEEGMTPA........
M.musculus_NCX3.1       ANAGADYEFTEGTVVLKPGETQKEFSVGIIDDDIFEEDEHFFVRLSNVRVEEEQLAEGMLPA........
G.gallus_NCX3           ANAGADYEFTEGTVVLKSGETQKDFAVGIIDDDIFEEDEHFFVRLSNLRVVDNEE.....PP........
D.rerio_NCX4a           ANAGSDYEFAEGTLVFKPGETVKELTVGVIDDDIFEEDEHFYVHLSNPRVVHRAEVSILDPNAVSPGNSI
T.nigroviridis_NCX4a    ASAGSDYEFAEGTLVFKPGETTKEFTVGIIDDDIFEEDEHFYQQHGGSFLSR..................
H.sapiens_NCX2.1        AKAGSDYEYSEGTLVFKPGETQKELRIGIIDDDIFEEDEHFFVRLLNLRVGDAQGMFEPDGGGR......
R.norvegicus_NCX2.1     AKAGSDYEYSEGTLVFKPGETQKELRIGIIDDDIFEEDEHFFVRLLNLRVGDAQGMFEPDGGGR......
M.musculus_NCX2.1       AKAGSDYEYSEGTLVFKPGETQKDLRIGIIDDDIFEEDEHFFVRLLNLRVGDAQGMFEPDGGGR......
S.kowalevskii_3         ANAGSDFHYAEGTLVFKPGETQKNIQLTIIDDDIFEEDEHFYIRLSNVRVGTADGMFESNHAG.......
T.adhaerens_2           ANSGEDYVTTNGTLRFKAGESRKDIKITIIDDDIYEEDEYFYVLLSNAKFVSTTDNEQ............
L.opalescens_NCX        ANAGSDYVYAEGTLVFYPMETHKQFPISIIDDDIFEEDEHFYIRLSNLRVGDSNGLFESGQAE.......
A.mellifera_NCX         AEAGSDYISAKGTLTFDSGETKKTIKLSVIDDELFEEDEHFYVRLSNASQP...................
D.melanogaster_NCX      ASAGTDFVGRKGLLSFPPGVDEQRFRIEVIDDDVFEEDECFYIRLFNPSEG...................
H.magnipapillata        ANEGDDYLPVKGTLEFKPNETTKKISITIIDDDLFELDEHFYCKLTAIRYYHVADNTQNKS.........
C.elegans_NCX1          AQAESDFVHTEGTITFEPGQTEQKIKVGIVDNDIYEDDEQFMVRLSQVRAFRSEHFSSVPARLG......
C.briggsae_NCX1         AQENSDFMPAEGTITFEPGQTEQKIKVGIVDNDIYEDDEQFMVRLSQVRAFRSEHFSAVPCRLG......
C.elegans_NCX2          ANAGSDYIPVKGTLTFYPEDKHQKVTIEVVDDDVFEEDEHFYLRLCNLRVRTKDGIIIDPTRIG......
C.briggsae_NCX2         ANAGSDYIPVKGTLTFYPEDKHQKVTIEVVDDDVFEEDEHFYLRLTNLRVRTKDGIIIDPTRIG......
T.spiralis              AVQDVHFLKKSETVVFAPQEREKTIRIEVCNPAQWRPGITFWVKLELIPGDKDDRITLGLASAAKIIYPK
M.brevicollis_1         AKAGSDYVALDRRVEFAAGQAEARVSIEIVDDDEPEDDEFFLARLKQPQP.......D............
Salpingoeca             ATMGVDFEETKGVAEFSAGEEEKVIPIKIIDDDEEEDDEHFYFYIKNPSP.......E............
S.kowalevskii_1         AEAGSDYIAQRDTMVFGSNETQKFIDIDIIDDNEWEPDEVFFVKLSVDSKSG...AK.............
S.kowalevskii_2         AEAGSDYIAQRDTMVFGSNETQKFIDIDIIDDNEWEPDEVFFVKLSVDSKSG...AK.............
T.adhaerens_1           AEPEKDYIPQNDVLVFEPNEKIKYVTIPIVDDNEWEPDENFFIQIWLEGSYD..................
D.pulex_1               AVGGQDFVTFNQVIQFEAGELEKIVTMEIIDDNQYEPDEQFYIKLSLTGTKA...IKQ............
P.humanus_corporis      AKAGDDYVKVDEILVFEPEETEKLVLVKIVDDTQWEPNEEFFLKLSLTEHSG...NAN............
O.dioica                ATKGEDYVEAKGVLEFREKETQKSITVEIIDDDQWEPDETFFVKLFLCVSRLVPNAAD............
A.pisum                 AKEGEDFVKVKEIVEFKENEEEKEIYIEILDDNKWEPDEEFFLRLSVVHH......KD............
P.marinus               AGEDYVPVIDGKITIRAGESSSDEVTIAIMDDDIVEEDENFWVRVTHVSEGAVI................
A.anophagefferens_1     VGGTGDYETCSGELTFADKENFKTIVIKVFDDNETEDDEVFYVDLLEVAHGGKAYAPG............
E.huxleyi               AHAGQDYTAVSGRLCFARGQRSAVLAVPLLDDEVAEEDETFFHRPSAGARL...................
C.intestinalis          AKINKDFKYTSGTLIFPEDTYVQSISIPIINDDQYYPDTDFYVILKNPSG....................
B.floridae              GKTGKDYTHTQGRLVFKETEYRKKIKIPIINDQQYQSDKDFYVILKSPAPRE..................
T.adhaerens_3           AKQGVNFKYSAGRLNFTKSEYQKTITVDILNDGQYHPNLSFYVILKNPS..E..................
N.vectensis_2           AKNGKEYQTVKGTMAFRAGINKHILRIPILPCEDFSKNKDFYVLLKNPSPEG..................
consensus>50            anagsdyef.egt.vfkpgetqkei.v.iidddifeede.Fyv.lsnv.v....................
```

```
                                   520       530       540                 550       560
H.sapiens_NCX1.1        NHVS...TLACLGSPSTATVTIFDDDHAGIFTFEEPVTHV.................SESIGIMEVKVLRT
P.troglodytes_NCX1.1    NHVS...TLACLGSPSTATVTIFDDDHAGIFTFEEPVTHV.................SESIGIMEVKVLRT
C.familiaris_NCX1.1     NHVS...ALACLGSPSTATVTIFDDDHAGIFTFEEPVTHV.................SESIGIMEVKVLRT
F.catus_NCX1.1          NHVS...TLACLGSPSTATVTIFDDDHAGIFTFEEPVTHV.................SESIGIMEVKVLRT
C.porcellus_NCX1.1      NHIS...TLACLGSPSTATVTIFDDDHAGIFTFEEPVTHV.................SESIGIMEVKVLRT
B.taurus_NCX1.1         SHVS...TLACLGSPSTATVTIFDDDHAGIFTFEEPVTHV.................SESIGIMEVKVLRT
R.norvegicus_NCX1.1     NHVS...AIACLGSPNTATITIFDDDHAGIFTFEEPVTHV.................SESIGIMEVKVLRT
M.musculus_NCX1.1       NHAS...SIACLGSPSTATITIFDDDHAGIFTFEEPVTHV.................SESIGIMEVKVLRT
M.mulatta_NCX1.3        NHVS...TLACLGSPSTATVTIFDDDHAGIFTFEEPVTHV.................SESIGIMEVKVLRT
O.cuniculus_NCX1.2      NHIS...TLACLGSPCTATVTIFDDDHAGIFTFEESVTHV.................SESIGIMEVKVLRT
S.tropicalis_NCX1       NHVT...PLACLGATCTATVTIFDDDHAGIFTFEETVTHV.................SESVGIMEVKVLRT
O.mossambicus_NCX1.1    NHVD...TLAGLGLPCTATVTIFDDDHAGIFTFEEPVMTV.................SESVGVMEVKVIRT
O.mykiss_NCX1           NHLD...TLAGLGLPCSATVTIFDDDHAGIFTFEEPVMTI.................SESIGMMEVKVLRT
D.rerio_NCX1a           NHVD...ALAGLGLPSTATVTIFDDDHAGIFLFEEPIVHI.................SESIGMMEVKVVRT
H.sapiens_NCX3.3        IFNSLPLPRAVLASPCVATVTILDDDHAGIFTFECDTIHV.................SESIGVMEVKVLRT
R.norvegicus_NCX3.3     ILNSLPLPRAVLASPCVATVTILDDDHAGIFTFECDTIHV.................SESIGVMEVKVLRT
M.musculus_NCX3.1       ILNSLPLPRAVLASPCVATVTILDDDHAGIFTFECDTIHV.................SESIGVMEVKVLRT
G.gallus_NCX3           ELGNSPYPKAILASPCVATVTILDDDHAGIFTFECDVIHV.................SESIGVMEVKVLRT
D.rerio_NCX4a           IGSSHVPPKAALGNAHSATVTIYDDDHAGIFTFESNSTRV.................SESVGIMQVKVHRT
T.nigroviridis_NCX4a    ......PPEAALGKAHTATVTIYDDDHAGIFTFESDSTKV.................SESIGNMQVKVHRT
H.sapiens_NCX2.1        .......PKGRLVAPLLATVTILDDDHAGIFSFQDRLLHV.................SECMGTVDVRVVRS
R.norvegicus_NCX2.1     .......PKGRLVAPLLATVTILDDDHAGIFSFQDRLLHV.................SECMGTVDVRVVRS
M.musculus_NCX2.1       .......PKGRLVAPLLATVTILDDDHAGIFSFQDRLLHV.................SECMGTVDVRVVRS
S.kowalevskii_3         .......PQARLVEPAFATVVILDDDHAGIFHFEEKLVKV.................AETAGELEIKVVRS
T.adhaerens_2           ........KCVIESPSTATVVILDDDHPGVFSFSESKYSV.................IETAGTLSLTVERK
L.opalescens_NCX        .......AKAQLANPFLATVMILDDDHPGIFQIDEKEMSL.................TESSGEVEVRIIRT
A.mellifera_NCX         .........AMLVSPSLATVMILDDDHSGVFGFPERDMEL.................VESVGQYPLRVMRY
D.melanogaster_NCX      .........VKLAVPMIATVMILDDDHAGIFAFTDSVFEI.................TESVGRFELKVMRY
H.magnipapillata        .......TKTTIGKANTATIIILDDDYPGIFTFEHSNFEV.................MESVGVLTLRVLRL
C.elegans_NCX1          .....LAA........TATVIIVDDDHAGSFGFLSEKFKC.................TESCGSFVAEVIRS
C.briggsae_NCX1         .....PAA........TATVIIVDDDHAGCFGFASEKFKC.................VESCGSFVAEVIRS
C.elegans_NCX2          .....GLPVAQLEMPNTATIMILDDDHAGVFGFEHDHFQV.................VENCGHLSLQMKRH
C.briggsae_NCX2         .....GLPVAQLEMPNTATIMILDDDHAGVFGFDHDHFQV.................VENCGHLSLQMKRH
T.spiralis              AAVGESVEFA...........ISDLRVGENEGFARIPISR.................RASLSTDPRSVFVM
M.brevicollis_1         .......NYRLIPARELAKIIIVDDDKPGEIEFEQTRMEVE.................EDCGCCEVVVRRR
Salpingoeca             .......QYLIVPGRNRARITIIDDDHPGVVSLELSKFSCL.................ENVGTIFLSVLRQ
S.kowalevskii_1         ...........LGKKNTMQITIINDDEPGTFEFTMPSYLVK.................ESIGVLQVPVSRN
S.kowalevskii_2         ...........LGKKHIMQITIINDEEPGTFEFTMPSYLVK.................ESIGVLQVPVSRN
T.adhaerens_1           ........DAIIGEKNSTEITIINDDEPGEIAFNKASYLFK.................ESCGTAKVEIVRK
D.pulex_1               .......KDVMLGRISIMEVTVLNDDDPGTITFEERGILVK.................ESVGVAQVPVLRK
P.humanus_corporis      .........VQIGRISIMEITILDDDKPGVISFEKTGILVK.................ESAECVVINVRRK
O.dioica                .......PRCKLGDRSINQVTIINDDDPGTFQFKEPAFMCK.................ESQGILKLPVCRT
A.pisum                 .......LSVKLGCISIMEVTIIDNDEPGIISFGKRGLLVK.................ESTGFVEVPIVRT
P.marinus               ............GKLSVAEVTIIDDDEIGELVLEKEELVVEGDVSPSPWPVVDPSEARLWQEAQVTVLRT
A.anophagefferens_1     .......AF...GDASTCEVTIIDDDEPGELSFEPPKEGAEGGPVLTM.........AETCGKARVKVGRY
E.huxleyi               ............GRRSTMAVTIIDDDGPGEVTFAVAETRVR.................ESQRVAVLEVERK
C.intestinalis          ........DATLGDPSVSRVTIIDDDKPGDFVLETSQLYADME.................KGEVSATVLRQ
B.floridae              ........DAALGDPSVARVTIIDDDKPGNFVFEQPIYYGDSR.................TFKVTATVLRQ
T.adhaerens_3           ........YSKLADPSVALVTIIDTNVPGEFQFEKAKYHVDKD.................SNQISLKVLRK
N.vectensis_2           ........ITDLGEPNLTRITIIDDDMPGEFSFEQASYHANFV.................TGEATCTVVRR
consensus>50            .........a.lg.p..atvt!.#ddhagif.fee....v..................esig.mev.vlr.
```

```
                          570       580                 590       600       610       620
H.sapiens_NCX1.1          SGARGNVIVPYKTIEGTARGG........GEDFEDTCGELEFQNDEIVKTISVKVIDDEEYEKNKTFFLE
P.troglodytes_NCX1.1      SGARGNVIVPYKTIEGTARGG........GEDFEDTCGELEFQNDEIVKTISVKVIDDEEYEKNKTFFLE
C.familiaris_NCX1.1       SGARGNVIVPYKTIEGTARGG........GEDFEDTCGELEFQNDEIVKTISVKVIDDEEYEKNKTFFLE
F.catus_NCX1.1            SGARGNVIVPYKTIEGTARGG........GEDFEDTCGELEFQNDEIVKTISVKVIDDEEYEKNKTFFLE
C.porcellus_NCX1.1        SGARGNVIVPYKTIEGTARGG........GEDFEDTCGELEFQNDEIVKTISVKVIDDEEYEKNKTFFLE
B.taurus_NCX1.1           SGARGNVIVPYKTIEGTARGG........GEDFEDTCGELEFQNDEIVKTISVKVIDDEEYEKNKTFFLE
R.norvegicus_NCX1.1       SGARGNVIIPYKTIEGTARGG........GEDFEDTCGELEFQNDEIVKTISVKVIDDEEYEKNKTFFIE
M.musculus_NCX1.1         SGARGNVIIPYKTIEGTARGG........GEDFEDTCGEPEFQNDEIVKTISVKVIDDEEYEKNKTFFIE
M.mulatta_NCX1.3          SGARGNVIVPYKTIEGTARGG........GEDFEDTCGELEFQNDEIVKIITIRIFDREEYEKECSFSLV
O.cuniculus_NCX1.2        SGARGNVIVPYKTIEGTARGG........GEDFEDTCGELEFQNDEIVKIITIRIFDREEYEKECSLSLV
S.tropicalis_NCX1         SGARGTVIVPYKTVEGTAKGG........GEDFEDTCGQLEFQNDEIVKIISVKIIDDEEYEKNKTFFLE
O.mossambicus_NCX1.1      SGARGVVVVPYKTIQGTAKGG........GEDFEDTHGVLEFENDEISKTIRINIIDDEEYEKNKNFFLE
O.mykiss_NCX1             SGARGLVVVPYKTMEGTAKGG........GEDFEDTHGALEFQNDEIFKSIQINIIDDEEYEKNKNFFLE
D.rerio_NCX1a             SGARGVVVIPYKTIEGTAKGG........GEDFEDTHGVLEFQNDEISKTIQINIIDDEEYEKNKNFFLE
H.sapiens_NCX3.3          SGARGTVIVPFRTVEGTAKGG........GEDFEDTYGELEFKNDETVKTIRVKIVDEEEYERQENFFIA
R.norvegicus_NCX3.3       SGARGTVIVPFRTVEGTAKGG........GEDFEDTYGELEFKNDETVKTIRVKIVDEEEYERQENFFIA
M.musculus_NCX3.1         SGARGTVIVPFRTVEGTAKGG........GEDFEDAYGELEFKNDETVKTIHIKVIDDKAYEKNKNYVIE
G.gallus_NCX3             SGARGTVIVPFRTVEGTAKGG........GEDFEDAYGELEFKNDETVKTIHIKVIDDEEYEKNKSFFIE
D.rerio_NCX4a             SGARGKVAVPYHTTEGTAKAG.........EDYDEVAGKLEFLNDETMKILEVKIIDDEEYEKNKTFTIH
T.nigroviridis_NCX4a      SGARGKVAIPYHTVEATAKAG.........EDYEEVSGKLEFQNDETMKLLNVKIIDDEEYEKNKTFTIV
H.sapiens_NCX2.1          SGARGTVRLPYRTVDGTARGG........GVHYEDACGELEFGDDETMKTLQVKIVDDEEYEKKDNFFIE
R.norvegicus_NCX2.1       SGARGTVRLPYRTVDGTARGG........GVHYEDACGELEFGDDETMKTLQVKIVDDEEYEKKDNFFIE
M.musculus_NCX2.1         SGARGTVRLPYRTVDGTARGG........GVHYEDACGELEFGDDETMKTLQVKIVDDEEYEKKDNFFIE
S.kowalevskii_3           SGARGTVIVPYHSIEGTAKGH........GDDFEDADGELEFHNDETSKFINVKIIDDEEYEKNENFYVE
T.adhaerens_2             SGARGTIRVPYHTKEGTAKGG........GDDYEDAVGELEFKNDETSKTIEVHIVDDEEYEKNEIFYVL
L.opalescens_NCX          SGARGCVKVPFHSVDGTATYG........K.DYELVDKDVIFDNDETEKFLRVRVVDDEEYEKNETFFIW
A.mellifera_NCX           SGARGRVVVPYRTIEGTAKPG........K.QYMHTESSLTFEDNQTEKEITLEIIEEDSYEKDALFYVE
D.melanogaster_NCX        SGARGTVIVPYWTENDTATES........K.DYEGARGELVFENNESEKFIDLFILEESSYEKDVSFKVH
H.magnipapillata          IGARGKVSIPYQTVEGSALGG........GADYEDSKGEIEFFDDETIKTVDIPIVNREEYEKTKTFTVI
C.elegans_NCX1            RGARGKVSIPYKTVDGAAKSP........Q.DYEHQEGVLKFADEQSKAEIYIPIVNDDEYEKHEDFYIE
C.briggsae_NCX1           RGARGEVSIPYKTIDGAAKSP........Q.DYVHQEGVLKFDDEQSKAEIYIPITNDDEYEKHEDFFIE
C.elegans_NCX2            SGARGKVIIPFRTVEGTASAD........K.HFEMKEGEIVFEDNQTEALVEIGIVDTEQYERSDYFYIE
C.briggsae_NCX2           SGARGKVVIPFRTIEGTAQAD........K.HFEMKEGEIVFEDNQTEATIDIGIIDTEQYERSDYFYIE
T.spiralis                TTMFSSASVEWKTENGTAKQG.........EDFVGEKGELILEPGVVEAYIDIPIVNDYEPEKDETFTVK
M.brevicollis_1           NGTKGAVACRYFMRNGSAIDG........H.DYEGSSGELQFQDGQATSIIRIPIIKDNVCEP.DEFFTV
Salpingoeca               NGSKGELKVNYATQDGTAHAP........H.DYEETKGTLVFEDGETEKVIAINIVDDEEYEL.DEHFFL
S.kowalevskii_1           NGTDGKVVLKWKTTDMTAISG........K.DYEGGEGELVFEHGEKQKSIDIPIVDDQEYEK.DESFKL
S.kowalevskii_2           NGTDGKVVLKWKTTDMTAISG........K.DYEGGKGELVFEHGEKQKSIDIPIVDDQEYEK.DESFKL
T.adhaerens_1             NGSDGTISVNWKTIDGSAING........K.DYRGGTGKIEFQHGEMSKLIEIPLIDDDIFEN.DETFHI
D.pulex_1                 NGADGEVSVRWRTIDKNAVSG........K.AYIGGEGCLVFKHSEMKRVIEIPISNDMMPEK.DECFEV
P.humanus_corporis        YGADGEVSVKWRTNNDTAISG........K.DFLGGEGELIFKHREILQTIEIPLLNEIRPEK.DSYFFV
O.dioica                  NGADGQVSVEWRVTPITAQHG........S.DYKEDCGTLVFNHGETQNDITITIIQTPEKEP.DETFKV
A.pisum                   HGSDSEVSVKWKTIDESAISG........R.DYVGGEGEIIFKDKEVAKILRVEIINDMCPEK.DECFEI
P.marinus                 KGCSGEVRVLYRTESGNAVAP........N.DYIHTEG...ELIMDHNQAAGVIKIPLRSTSTRTPHFRL
A.anophagefferens_1       NGSAGQVQCDYELVDGTACCG........K.DFGGTVGFEREGTFVFENTEVEKFIEIPVINTNRYEGES
E.huxleyi                 RGVQGAVSVGWRTADGTALAS........R.DYVPGRGRLTFAPGVTRAAIEVEIIDDGAYDLADESFAV
C.intestinalis            CGSDGVVFVHYATIDGTAHGG...ETMDLGVDYVHTSGKLEFKHQETSKVITIEINKDITETKNFVIILR
B.floridae                NGTDGNTSVEYTTMDGTAKGG...PFSSPTLDYISNSGVLYFAHGETSKQISINLNKEKMGHRNFVILLR
T.adhaerens_3             KGVDGTVHLEFATIDGTAHGGIIPEVEENHKDYESKTDMLIFANGETEKEIVINVASHVKLDKNFLVALR
N.vectensis_2             KGCDGTVTLKYHTMNGTAKGGTKNELEAKTCDYEQVTDGLLTFHHGEMSKHITVKINTETQDTNFVVLLH
consensus>50              sgarg.vivpy.tiegtA.gg........g.d%ed..gelefqnde..k.i.i.iiddeeyekn..ff..
```

```
                        630                                            640       650
H.sapiens_NCX1.1        IGEPRLVEMS......................................EKKALLLNELGGFTIT.GKYLF
P.troglodytes_NCX1.1    IGEPRLVEMS......................................EKKALLLNELGGFTIT.GKYLF
C.familiaris_NCX1.1     IGEPRLVEMS......................................EKKALLLNELGGFTIT.GKYLY
F.catus_NCX1.1          IGEPRLVEMS......................................EKKALLLNELGGFTIT.GKYLY
C.porcellus_NCX1.1      IGEPRLVEMS......................................EKKALLLNELGGFTIT.GKHLY
B.taurus_NCX1.1         IGEPRLVEMS......................................EKKALLLNELGGFTIT.GKYLY
R.norvegicus_NCX1.1     IGEPRLVEMS......................................EKKALLLNELGGFTLTEGKKMY
M.musculus_NCX1.1       IGEPRLVEMS......................................EKKALLLNELGGFTLT.GKEMY
M.mulatta_NCX1.3        LEEPKWIRRG......................................MK........GGFTIT......
O.cuniculus_NCX1.2      LEEPKWIRRG......................................MK.ALLLNELGGFTIT......
S.tropicalis_NCX1       VGEPRLVEMS......................................EKKALLLNELGDFTIT.GKILY
O.mossambicus_NCX1.1    IGEPRLLEMS......................................ERKAVLLQEVGGFVKT......
O.mykiss_NCX1           MGEPQLLEMS......................................ERKAVLLQEIGGFVKT......
D.rerio_NCX1a           IGEPQLVEMS......................................ERKAMLLHECGGFVKTD.KQLY
H.sapiens_NCX3.3        LGEPKWMERG......................................IS.ALLLSPDVTDRK.......
R.norvegicus_NCX3.3     LGEPKWMERG......................................IS.ALLLSPEVTDRK.......
M.musculus_NCX3.1       MMGPRMVDMS......................................VQKALLLSPEVTDRK.......
G.gallus_NCX3           LMSPRMVDMS......................................LQKALILLAE...RK.......
D.rerio_NCX4a           LGEPVLLEIG......................................QKHG..................
T.nigroviridis_NCX4a    LEEPILLEVG......................................QKHG..................
H.sapiens_NCX2.1        LGQPQWLKRG......................................ISALLLNQGDGDRK........
R.norvegicus_NCX2.1     LGQPQWLKRG......................................ISALLLNQGDGDRK........
M.musculus_NCX2.1       LGQPQWLKRG......................................ISALLLNQGNGDKK........
S.kowalevskii_3         LGEPRLVKRG......................................QGSDDEDSAYGDK.........
T.adhaerens_2           LGEPYFK...............................................................
L.opalescens_NCX        LDEPYLVKKP......................................TGSSSGSVVEDDDP........
A.mellifera_NCX         LGEPQLQGGD......................................GLAAEMKQPEER..........
D.melanogaster_NCX      IGEPRLAPDD......................................ELAAKIKEVEKKPV........
H.magnipapillata        LGEPKIVGKL......................................NSTITNLSSI............
C.elegans_NCX1          LGEPIWHRELADDEEGIEGKPILG..............................................
C.briggsae_NCX1         LGDPIWHKEIKDDEEGAEGKPILG..............................................
C.elegans_NCX2          LSPPIWAKKMNDLSRIQERFQRRMERKRGSSVASESKDSNTENALAPAEKSTRAASVDLLQPSSDPRRSS
C.briggsae_NCX2         LSPPIWAKKMNDLSRIQERFQRRMERKRGSSVASESKDSNTETALAPTDKSTRAASVDLLQPCSDPRRSS
T.spiralis              LLP...................................................................
M.brevicollis_1         HLEEVS................................................................
Salpingoeca             TLEESE................................................................
S.kowalevskii_1         ELLEVS................................................................
S.kowalevskii_2         ELLEVS................................................................
T.adhaerens_1           ELFDPN................................................................
D.pulex_1               ELSDPT................................................................
P.humanus_corporis      ELFSPS................................................................
O.dioica                SIVKVS................................................................
A.pisum                 KLFETT................................................................
P.marinus               ILSKPS................................................................
A.anophagefferens_1     EFTIVL................................................................
E.huxleyi               VLDEPG................................................................
C.intestinalis          ......................................................................
B.floridae              ......................................................................
T.adhaerens_3           ......................................................................
N.vectensis_2           ......................................................................
consensus>50            .gep..................................................................
```

```
                        660       670       680       690       700       710       720
H.sapiens_NCX1.1        GQPVFRKVHAREHPILSTVITIADEYDDKQPLTSKEEEERRIAEMGRPILGEHTKLEVIIEESYEFKSTV
P.troglodytes_NCX1.1    GQPVFRKVHAREHPILSTVITIADEYDDKQPLTSKEEEERRIAEMGRPILGEHTKLEVIIEESYEFKSTV
C.familiaris_NCX1.1     GQPVFRKVHAREHPIPSTVITIAEEYDDKQPLTSKEEEERRIAEMGRPILGEHTKLEVIIEESYEFKSTV
F.catus_NCX1.1          GQPVFRKVHAREHPIPSTVITIAEECDAKQPLTSKEEEERRIAEMGRPILGEHTKLEVIIEESYEFKSTV
C.porcellus_NCX1.1      GQPVLRKVHARDHPIPSTVITIADEYDDKQPLTSKEEEERRIAELGRPILGEHTKLEVIIEESYEFKSTV
B.taurus_NCX1.1         GQPVFRKVHAREHPLPSTIITIADEYDDKQPLTSKEEEERRIAEMGRPILGEHTRLEVIIEESYEFKSTV
R.norvegicus_NCX1.1     GQPVFRKVHARDHPIPSTVISISEEYDDKQPLTSKEEEERRIAEMGRPILGEHTKLEVIIEESYEFKSTV
M.musculus_NCX1.1       GQPIFRKVHARDHPIPSTVITISEEYDDKQPLTSKEEEERRIAEMGRPILGEHTKLEVIIQESYEFKSTV
M.mulatta_NCX1.3        .......................DEYDDKQPLTSKEEEERRIAEMGRPILGEHTKLEVIIEESYEFKSTV
O.cuniculus_NCX1.2      .......................EEYDDKQPLTSKEEEERRIAEMGRPILGEHTKLEVIIEESYEFKSTV
S.tropicalis_NCX1       GKPVLRKVQVRDHPIPSTVIILTEENEEKQPLTSKEEEERRIAEMGRPVLGEHTRLEIIIEESYEFKSTV
O.mossambicus_NCX1.1    GRDIYRKVQGREHPVPSNIISIA.EDGAEEPLTKKEEEERRIAEMGRPMLGEHVKLEVIIEESYEFKSTV
O.mykiss_NCX1           GRDVYRKVQGRDNPVPATIISLA.EEGDEEALSKKEEEERRIAEMGRPTLGEHVKLEVVIEESYEFKNTV
D.rerio_NCX1a           GRDVYRKVQGRDKPIPSTIISIS.EDGEEETLTKKEKDERRIAEMGRPTLGEHVKLEVIIEESYEFKSTV
H.sapiens_NCX3.3        ...............................LTMEEEEAKRIAEMGKPVLGEHPKLEVIIEESYEFKTTV
R.norvegicus_NCX3.3     ...............................LTMEEEEAKRIAEMGKPVLGEHPKLEVIIEESYEFKSTV
M.musculus_NCX3.1       ...............................LTVEEEEAKRIAEMGKPVLGEHPKLEVIIEESYEFKSTV
G.gallus_NCX3           ...............................LTVEEEEAKRIAEMGKPILGEHPKLEVIIEESYEFKSTV
D.rerio_NCX4a           ..........................DSNDNKPAVGAEEEEVAKMGCPSLGEHTKLEVVIEESYEFKNTV
T.nigroviridis_NCX4a    ..........................DTNDNKTSVGPED..VSKMGCPCLGEHTKLEVVIEESYEFKSTV
H.sapiens_NCX2.1        ...............................LTAEEEEARRIAEMGKPVLGENCRLEVIIEESYDFKNTV
R.norvegicus_NCX2.1     ...............................LTAEEEEAQRIAEMGKPVLGENCRLEVIIEESYDFKNTV
M.musculus_NCX2.1       ...............................ITAEQEEAQRIAEMGKPVLGENNRLEVIIEESYDFKNTV
S.kowalevskii_3         ...........................ENSGSNHMTEDEKRVAEMGKPRLGDLIKCEVKILESTEFKNTV
T.adhaerens_2           .......................DKKRIEKERNHVAKTTGNQPIDVNPSLGPYKEAEVTIIESHEFKNTV
L.opalescens_NCX        .................................VL........AELGKPRRGENIKITVHIIESTEFKSVV
A.mellifera_NCX         ....................................TAEEKMALLGKPKLGEVFRAQIRIKESKEFKNTV
D.melanogaster_NCX      .................................QDLTELDRILLLSKPRNGELTTAYVRIRESQEFKATV
H.magnipapillata        ..................................KDDDLRKVLEVGKPTLGDHKACEIKIVESKEFRKTI
C.elegans_NCX1          ....................................................FSRCKVVITEDREFKNFM
C.briggsae_NCX1         ....................................................FARCKVVITEDRDFKNFV
C.elegans_NCX2          QTNSPHLTSRFRNRLGSWIAGMKGGNGDDEVTTSLTPSQLEIAEMGKPRLGEFTKCQITIRESKEFQGIV
C.briggsae_NCX2         QSNSPHLTSRFRNRLGSWIAGMKGGNGDDEVTTALTPSQLEIAEMGKPRLGEFTKCQITIRESKEFQGIV
T.spiralis              .............................................GKASLGACQSIVVTIVNDDNISRYY
M.brevicollis_1         ..............................................GSTLGKVTVCRVVILNDDKAQRLL
Salpingoeca             ..............................................DVTFGEVTKCKITILNDDEVTTLA
S.kowalevskii_1         .............................................DGAKLGKTRTSVITIVNDDEYNSLL
S.kowalevskii_2         .............................................DGAKLGKTRTSVITIVNDDELNNLL
T.adhaerens_1           .............................................GGAKLGSRTRTALTIVNDDVYNSLL
D.pulex_1               .............................................GGAVLGSITKLAVTITNDEEFNSVL
P.humanus_corporis      .............................................NDASLGDIKVICVTITNDEDFANII
O.dioica                .............................................TGAAMGHTRETVVTVIGDEEYKNLV
A.pisum                 .............................................GGARIGNVNRIAITISSDDDFDSVV
P.marinus               .............................................GGAKFCS............KTDGGH
A.anophagefferens_1     .............................................KNFKCAV............ERAKFG
E.huxleyi               .............................................GGASLGEIGRAVVVIESDDERRALV
C.intestinalis          ..............................................NPSQGSHLGEHSAAVVN..IN...
B.floridae              ..............................................NPSLGSKIGEPGAAVGF..LCKDE
T.adhaerens_3           ..............................................NASMGARIGEIGATIVSFDIERDA
N.vectensis_2           ..............................................DPSTGARIGNRTATVVHISSGI..
consensus>50            .....................................e...iaemg.p.lge....evii.es.efk.tv
```

```
                     730        740       750          760          770       780
H.sapiens_NCX1.1     DKLIKKTNLALV...VGTNSWREQFIEAITVSAG.E.....DDDDDECG......EEKLPSCFDYVMHFL
P.troglodytes_NCX1.1 DKLIKKTNLALV...VGTNSWREQFIEAITVSAG.E.....DDDDDECG......EEKLPSCFDYVMHFL
C.familiaris_NCX1.1  DKLIKKTNLALV...VGTNSWREQFIEAITVSAG.E.....DDDDDECG......EEKLPSCFDYVMHFL
F.catus_NCX1.1       DKLIKKTNLALV...VGTNSWREQFIEAITVSAG.E.....DDDDDECG......EEKLPSCFDYVMHFL
C.porcellus_NCX1.1   DKLIKKTNLALV...VGTNSWREQFIEAITVSAG.E.....DDDDDECG......EEKLPSCFDYVMHFL
B.taurus_NCX1.1      DKLIKKTNLALV...VGTNSWREQFIEAITVSAG.E.....DDDDDECG......EEKLPSCFDYVMHFL
R.norvegicus_NCX1.1  DKLIKKTNLALV...VGTNSWREQFIEAITVSAG.E.....DDDDDECG......EEKLPSCFDYVMHFL
M.musculus_NCX1.1    DKLIKKTNLALV...VGTNSWREQFIEAITVSAG.E.....DDDDDECG......EEKLPSCFDYVMHFL
M.mulatta_NCX1.3     DKLIKKTNLALV...VGTNSWREQFIEAITVSAG.E.....DDDDDECG......EEKLPSCFDYVMHFL
O.cuniculus_NCX1.2   DKLIKKTNLALV...VGTNSWREQFIEAITVSAG.E.....DDDDDECG......EEKLPSCFDYVMHFL
S.tropicalis_NCX1    DKLIKKTNLALV...VGTNSWREQFIEAITVSAG.E.....DDDDDECG......EEKLPSCFDYVMHFL
O.mossambicus_NCX1.1 DKLIKKTNLALL...IGTNSWREQFVEAITVSSG.......DDD..ECR......EEKLPSCFDYVMHFL
O.mykiss_NCX1        DKLIKKTNLALL...IGTNSWRQQFMEAITVSSG.......DDDEDECG......EEKLPSCFDYVMHFL
D.rerio_NCX1a        DKLIKKTNLALL...VGTNSWRDQFVEAITVNSG.......DDDDEECG......QEKMPSCFDYVMHFL
H.sapiens_NCX3.3     DKLIKKTNLALV...VGTHSWRDQFMEAITVSAA.G.....DEDEDESG......EERLPSCFDYVMHFL
R.norvegicus_NCX3.3  DKLIKKTNLALV...VGTHSWRDQFMEAITVSAA.G.....DEEEDESG......EERLPSCFDYVMHFL
M.musculus_NCX3.1    DKLIKKTNLALV...VGTHSWRDQFMEAITVSAG.G.....DEDEDESG......EERLPSCFDYVMHFL
G.gallus_NCX3        DKLIKKTNLALV...VGTHSWRDQFLEAITVSAA.G.....DEDEDESG......EERLPSCFDYVMHFL
D.rerio_NCX4a        DKLIKKTNLALV...VGSSSWREQFVSAVTVSAG.......DDDEEESG......EERLPSCFDYIMHFL
T.nigroviridis_NCX4a DKLIKKTNLALV...VGSSSWREQFVSAVTVSAG.......DDDEEESG......EERLPSCFDYIMHFL
H.sapiens_NCX2.1     DKLIKKTNLALV...IGTHSWREQFLEAITVSAGDE.....EEEEDGSR......EERLPSCFDYVMHFL
R.norvegicus_NCX2.1  DKLIKKTNLALV...IGTHSWREQFLEAVTVSAGDE.....EEDEDGSR......EERLPSCFDYVMHFL
M.musculus_NCX2.1    DKLIKKTNLALV...IGTHSWREQFIEAVTVSAGDE.....EEDEDGPR......EERLPSCFDYVMHFL
S.kowalevskii_3      DKLIKKANLSLV...VGTSSWREQFVEAVTVSSG.........DEDEDG......EEKLPSCSDYVMHFL
T.adhaerens_2        DKLLKKANLAMA...IGTSTWREQFRAALTVNGGDN.....DDDDD.........EETKPTCTDYIMHYL
L.opalescens_NCX     DKLLKKANLSLV...VGTSSWREQFIEAITVN.AEG.....DDDDEEGE......EEKLPSCMDYIMHFV
A.mellifera_NCX      DKLVQRANASIL...LGTSSWKEQFTEALTVSGGDE.....D..DEGGG......EPAAPSTLDYLMHGV
D.melanogaster_NCX   DKLVAKANVSAV...LGTSSWKEQFKDALTVIPADE.....SEFDNDDE......EEEVPSCFSYVSHFV
H.magnipapillata     DQMLSAKNKSIL....GSSSWAQQFKDAFQVEYGG.........DEEEG......DDVEPTYGDYVMHYL
C.elegans_NCX1       DRALVTANTSIM...VGTSSWKQQFTEAWTLEP....................EEEDGEVTTMEKVMHYI
C.briggsae_NCX1      DKVLVTANTSIM...VGTSSWKQQFTEALTLEP....................EEEGGDVSLQEKIMHYV
C.elegans_NCX2       DRMIKNANTRIM...LGTHSWREQFMEALVVSAGDD.....DDDEGEDGEDGEEKEPEEPGCMDYVMHVL
C.briggsae_NCX2      DRMIKNANTRIM...LGTHSWREQFMEALVVSAGDD.....DDDDEE.GDDGEEKEPEEPGCMDYFMHVL
T.spiralis           AKFEEKVRQSLLTCKLESSTWKEQFLKAVSVNGG.....................ETSDASMVDCFAHII
M.brevicollis_1      EKVSRRMKINMDRFRVGSSDWKQQFRDAV.....................AWPE...REAGVLGIVMHLI
Salpingoeca          DKVTALLNLNLDKFRIGGTDWKSQFVDAL.....................AWPE...EGSGTFAIVMHLI
S.kowalevskii_1      DRVVSITNVNLDRLRIGNASWADQFKEAM.....................NVNGGDIESATWVDYVMHFL
S.kowalevskii_2      DRVVSITNVNLDRLRIGNASWADQFKEAM.....................NVNGGDIESATWVDYVMHFL
T.adhaerens_1        DKVVALTRINVHRLKLGSQSWGEQFTNAM.....................TVNGGDVENATGMDYFLHFL
D.pulex_1            NRLADMTTANVDSMEVHHSTWTSQIKDAM.....................NVNGGDIENATTSDYVMHFL
P.humanus_corporis   DKMLAMTNLKLKTLSIYRKTWASQIKDAM.....................NVNGGDVKNATATDYVLHFI
O.dioica             KRVAAQTHVALSKMEIGGDSWYEQFVEAM.....................NVNGGDIENATGFDYIMHLL
A.pisum              DRLMLLTNINIHALELHRQTWAEQIKTAM.....................TVNGGDLTNATTLTYVLHFF
P.marinus            DSLICTIWIKPKSGISQGRTTVTDLLNVHWGKMSLGATNWREQFNEALWVNGSREEQSEAGVGDWVMHVI
A.anophagefferens_1  KHTELKVVIVADTETKDMIDNVQKYLESNDASYNVGSTWGQQFSDALKVGGGDADDDFKASAGDWVMHII
E.huxleyi            KQVASLMSLNVDAMQLAGASWREQLAEAVTFEPPRQATTARALDGPLLGGASPPAALSLSCPFAFAGFAL
C.intestinalis       LEACGINSMSRVLIDENDTTWVGQIRNAMIV................GGEMDDYGNESPPSNTDFLMHAI
B.floridae           LAERVANVVPMGDTEEEDTSWGGQFRSAMCL................ESEEDEDGKKIPPSVGQLIMHFV
T.adhaerens_3        LGDRVAEFAALNGVEGEDVSWAEQFTNALTV................GSDIDEFGEESPPSNLDYILHFI
N.vectensis_2        ...........................AMII................QGDEDKDGNELPLNSIDFIMHFL
consensus>50         dklikktnlal....vgt.swreqf.eaitvs.g.......ddddde.g......ee.lpscfdyvmhfl
```

```
                          790       800       810       820       830       840
H.sapiens_NCX1.1          TVFWKVLFAFVPPTEYW..NGWACFIVSILMIGLLTAFIGDLASHFGCTIGLKDSVTAVVFVALGTSVPD
P.troglodytes_NCX1.1      TVFWKVLFAFVPPTEYW..NGWACFIVSILMIGLLTAFIGDLASHFGCTIGLKDSVTAVVFVALGTSVPD
C.familiaris_NCX1.1       TVFWKVLFAFVPPTEYW..NGWACFIVSILMIGILTAFIGDLASHFGCTIGLKDSVTAVVFVALGTSVPD
F.catus_NCX1.1            TVFWKVLFAFVPPTEYW..NGWACFIVSILMIGILTAFIGDLASHFGCTIGLKDSVTAVVFVALGTSVPD
C.porcellus_NCX1.1        TVFWKVLFAFVPPTEYW..NGWACFIVSILMIGLLTAFIGDLASHFGCTIGLKDSVTAVVFVALGTSVPD
B.taurus_NCX1.1           TVFWKVLFAFVPPTEYW..NGWACFIVSILMIGLLTAFIGDLASHFACTIALKDSVTAVVFVALGTSVPD
R.norvegicus_NCX1.1       TVFWKVLFAFVPPTEYW..NGWACFIVSILMIGLLTAFIGDLASHFGCTIGLKDSVTAVVFVALGTSVPD
M.musculus_NCX1.1         TVFWKVLFAFVPPTEYW..NGWACFIVSILMIGLLTAFIGDLASHFGCTIGLKDSVTAVVFVALGTSVPD
M.mulatta_NCX1.3          TVFWKVLFAFVPPTEYW..NGWACFIVSILMIGLLTAFIGDLASHFGCTIGLKDSVTAVVFVALGTSVPD
O.cuniculus_NCX1.2        TVFWKVLFAFVPPTEYW..NGWACFIVSILMIGLLTAFIGDLASHFGCTIGLKDSVTAVVFVALGTSVPD
S.tropicalis_NCX1         TVFWKVLFAFVPPTEYW..NGWACFIVSISMIGLLTAFIGDLASHFGCTIGLKDSVTAVVFVALGTSVPD
O.mossambicus_NCX1.1      TIFWKLLFAFVPPTDYW..NGWACFVVSITVIGMLTAVIGDLASHFGCTVGLKDSVTAVVFVALGTSVPD
O.mykiss_NCX1             TVFWKLLFAFVPPTDYW..NGWACFVVSISMIGLLTAFIGDLASHFGCTVGLKDSVTAVVFVALGTSVPD
D.rerio_NCX1a             TVFWKVLFAFVPPTDYW..NGWACFIVSIMMIGVLTAFIGDIASHFGCTIGLKDSVTAVVFVALGTSVPD
H.sapiens_NCX3.3          TVFWKVLFACVPPTEYC..HGWACFAVSILIIGMLTAIIGDLASHFGCTIGLKDSVTAVVFVAFGTSVPD
R.norvegicus_NCX3.3       TVFWKVLFACVPPTEYC..HGWACFVVSILIIGMLTAIIGDLASHFGCTIGLKDSVTAVVFVAFGTSVPD
M.musculus_NCX3.1         TVFWKVLFACVPPTEYC..PGWACFVVSILIIGMLTAIIGDLASHFGCTIGLKDSVTAVVFVAFGTSVPD
G.gallus_NCX3             TVFWKVLFACVPPTEYC..NGWACFVVSILIIGMLTAVIGDLASHFGCTIGLKDSVTAVVFVAFGTSVPD
D.rerio_NCX4a             TVFWKVLFAFVPPTEYW..NGWACFIVSITLIGVLTAVTGDLASHFGCTVGLKDTVTAVVFVALGTSVPD
T.nigroviridis_NCX4a      TVFWKVLFAFVPPTEYW..NGWACFFVSISLIGVLTAVTGDLASHFGCTIGLKDSVTAVVFVALGTSVPD
H.sapiens_NCX2.1          TVFWKVLFACVPPTEYC..HGWACFGVSILVIGLLTALIGDLASHFGCTVGLKDSVNAVVFVALGTSIPD
R.norvegicus_NCX2.1       TVFWKVLFACLPPTEYC..HGWACFGVCILVIGLLTALIGDLASHFGCTVGLKDSVNAVVFVALGTSIPD
M.musculus_NCX2.1         TVFWKVLFACVPPTEYC..NGWACFGVCILVIGVLTALIGDLASHFGCTVGLKDSVNAVVFVALGTSIPD
S.kowalevskii_3           SIFWKILFAFVPPTDIW..GGWACFTVSILMIGLLTAVIGDLASHFGCTVGLKDTVTAISFVALGTSVPD
T.adhaerens_2             TIFWKLIFALVPPTEIW..GGWACFCVSIIMIGILTTFIGDLASHFGCTVGLKDSVTAITFVALGTSLPD
L.opalescens_NCX          CLFWKVLFAFVPPTDYW..GGWACFTVSIILIGVLTAFIGDLATYFGCTIGLKDAVTAVSFVALGTSVPD
A.mellifera_NCX           TILWKVLFAFVPPTDIA..GGYLCFVVSIFGIGVVTAVIGDVASYFGCTLGIKDSVTAVAFVALGTSIPD
D.melanogaster_NCX        CLFWKVLFAFVPPTDIC..GGYVTFVVSIFVIGVITAIIGDAASYFGCALNIKDSVTAILFVALGTSIPD
H.magnipapillata          SFFWKVLFACVPPTDIW..GGWACFTASITMIGLLTAITGDIASHFGCTVGLADSVVAISFVALGTSLPD
C.elegans_NCX1            ALPWKLLFALIPPTDYF..NGWLCFVVAIAMIGLLTAFIGDIAAAFGCTVGLKDSVTALTLVAMGTSLPD
C.briggsae_NCX1           ALPWKLLFALIPPTDYF..NGWCCFVVAIIMIGVLTAFIGDIAAAFGCTVGLKDSVTALTLVAMGTSLPD
C.elegans_NCX2            TVPWKLTFATIPPTDYF..GGWATFVVAIFMIGVLTAVVGDLASQFGCWVGLKDAVTAISFVALGTSVPD
C.briggsae_NCX2           TVPWKLTFATIPPTDYI..GGWATFVVAIFMIGVLTAVVGDLASQFGCWVGLKDAVTAISFVALGTSVPD
T.spiralis                AFPWKVIAAFVPPTTIF..GGWLAFIVALALIGLLTAIIGDLAAIFGCLVGLKDSVTAITFVALGTSLPD
M.brevicollis_1           TLPWKVLAACIPPTVFVH..GWVAFGVALCFIGLVTALIGDLASLFGCSLGLADTITAITFVALGTSLPD
Salpingoeca               NIPWKIIAACLPPTLFLN..GWVTFGSALIMIGIVTATIGDLASLMGCAMGLEDAVTAITFVALGTSLPD
S.kowalevskii_1           TFGWKVIFALVPPPSFLNSGGWICFTVAISFIGLLTAIIGDLAAIFGCLIDLRESVVAITFVALGTSLPD
S.kowalevskii_2           SFGWKVIFALVPPPSFLDSGGWISFCVALTFIGVLTTIIGSLGSIFGCLINMKDSVTGLTFVTLGTCIVD
T.adhaerens_1             TFGWKVIFAIIPPPSML..GGWLTFIASLGMIGFLTAIVGDLAGIFGCIVGLRDAVTAITFVALGTSLPD
D.pulex_1                 TFGWKLLFALVPPAGIW..GGWLSFFVSLVVIGALTAIVGDLASIFGCLVGLDDAVTAITFVALGTSLPD
P.humanus_corporis        SFNWKVLFSFIPPPSMF..RGWLCFIVSLCAIGFVTAVIEDIASLFGCVVGLKDTITAITLVAMGTSLPD
O.dioica                  TFGFKTIFAIIPPSNMG..GGFPCFFGSLTMIGVMTVIISDLANIFGCLVGLKPAVNSITLVALGTSLPD
A.pisum                   SFFWKVVFAFIPPAAVFS..GWLRFFTSLTLIGVMATIIGDLATIFGCLIGLDDAVTAITIVALGMSLPD
P.marinus                 TFPWKIVFAFCPPLEYA..DGWLTFFISLSMIGGVTAIIADMASLLGCTMDIPDSVTAITLVALGTSLPD
A.anophagefferens_1       TLPWKVAFAFVPPTSYG..GGWVCFYVALTFIGIVTAFIGDLAALMGCCLGLKDTVTAITFVALGTSLPD
E.huxleyi                 ALPWRLLFALTPPPRVA..GGWACFFVALVGIGVITALVGDLAAHMGCCLGMKPAVTAITFVALGTSLPD
C.intestinalis            SMPWKIVFALAPTRHAY..GGFPAFIISLLMIGCLTAIIEQLGHLLGCVATLKTQVTGITIIALGTSLPD
B.floridae                TFFWKVLFAFIPPRSML..GGWPAFVMSLGFITMLTAFIETLGHLLGCVFGVRTSVTGITIIALGTSVPD
T.adhaerens_3             AFLWKIIAATIPPRTVL..GGWPAFVLSLVYIGFLTAIIEQFGNLLGCVILLKPSVTGITIVALGTSLPD
N.vectensis_2             TFFWKVLFAFIPPRPLL..GGWPAFVVSLLFIAALTAVIEQLGKLLGCVVDLRNSVTGITIIAIGTSLPD
consensus>50              tvfwkvlfafvPptey....GwacFivsi.mIg.ltaiigdlashfgCtiglkds!tav.f!alGtsvpD
```

```
                         850       860       870       880       890
H.sapiens_NCX1.1         TFASKVAATQDQY..ADASIGNVTGSNAVNVFLGIGVAWSIAAIYHAA......................
P.troglodytes_NCX1.1     TFASKVAATQDQY..ADASIGNVTGSNAVNVFLGIGVAWSIAAIYHAA......................
C.familiaris_NCX1.1      TFASKVAATQDQY..ADASIGNVTGSNAVNVFLGIGVAWSIAAIYHAA......................
F.catus_NCX1.1           TFASKVAATQDQY..ADASIGNVTGSNAVNVFLGIGVAWSIAAIYHAA......................
C.porcellus_NCX1.1       TFASKVAATQDQY..ADASIGNVTGSNAVNVFLGIGVAWSIAAIYHAA......................
B.taurus_NCX1.1          TFASKVAATQDQY..ADASIGNVTGSNAVNVFLGIGVAWSIAAIYHAA......................
R.norvegicus_NCX1.1      TFASKVAATQDQY..ADASIGNVTGSNAVNVFLGIGVAWSIAAIYHAA......................
M.musculus_NCX1.1        TFASKVAATQDQY..ADASIGNVTGSNAVNVFLGIGVAWSIAAIYHAA......................
M.mulatta_NCX1.3         TFASKVAATQDQY..ADASIGNVTGSNAVNVFLGIGVAWSIAAIYHAA......................
O.cuniculus_NCX1.2       TFASKVAATQDQY..ADASIGNVTGSNAVNVFLGIGVAWSIAAIYHAA......................
S.tropicalis_NCX1        TFASKVAATQDQY..ADASIGNVTGSNAVNVFLGIGVAWSIAAIYHAA......................
O.mossambicus_NCX1.1     TFASKVAAIQDQY..ADASIGNVTGSNAVNVFLGIGVAWSIAAVYHYT......................
O.mykiss_NCX1            TFASKVAAIQDQY..ADAFIGNVTGSNAVNVFLGIGVAWSIAAIYHNS......................
D.rerio_NCX1a            TFASKVAAIQDQY..ADASIGNVTGSNAVNVFLGIGVAWSIAAIFHQS......................
H.sapiens_NCX3.3         TFASKAAALQDVY..ADASIGNVTGSNAVNVFLGIGLAWSVAAIYWAL......................
R.norvegicus_NCX3.3      TFASKAAALQDVY..ADASIGNVTGSNAVNVFLGIGLAWSVAAIYWAM......................
M.musculus_NCX3.1        TFASKAAALQDVY..ADASIGNVTGSNAVNVFLGIGLAWSVAAIYWAM......................
G.gallus_NCX3            TFASKAAAIQDVY..ADASITNVTGSNAVNVFLGIGLAWSVAAIYWAS......................
D.rerio_NCX4a            TFASKVAAIQDQY..ADASIGNVTGSNAVNVFLGIGVAWTIAAVYWHS......................
T.nigroviridis_NCX4a     TFASKVAAIQDQY..ADASIGNVTGSNAVNVFLGIGVAWTIAAVYWHS......................
H.sapiens_NCX2.1         TFASKVAALQDQC..ADASIGNVTGSNAVNVFLGLGVAWSVAAVYWAV......................
R.norvegicus_NCX2.1      TFASKVAALQDQC..ADASIGNVTGSNAVNVFLGLGVAWSVAAVYWAV......................
M.musculus_NCX2.1        TFASKVAALQDQC..ADASIGNVTGSNAVNVFLGLGVAWSVAAVYWAV......................
S.kowalevskii_3          TFASKTAAVGDKY..ADASVGNVTGSNAVNVFLGIGLAWSAAAIYWAA......................
T.adhaerens_2            TFASKTAAVNDKY..ADSSIGNVTGSNSVNVFLGLGLAWCVAAVANAI......................
L.opalescens_NCX         TFASKVAAINDKY..ADSSIGNVTGSNAVNVFLGIGIAWSIAAIYHAA......................
A.mellifera_NCX          TFASKVAACQDKY..ADASVGNVTGSNAVNVFLGIGVAWSIAAIYHAL......................
D.melanogaster_NCX       TFASMIAAKHDEG..ADNCIGNVTGSNAVNVFLGIGLAWTIAAVYHSS......................
H.magnipapillata         TFASKVATINDEH..ADGSIGNVTGSNSVNVFLGIGLAWSIAAIYHAS......................
C.elegans_NCX1           TFASRTAAVGDQW..ADGSIGNVTGSNAVNVFLGIGIAWMIAACVHAY......................
C.briggsae_NCX1          TFASRTAAVGDQW..ADGSIGNVTGSNAVNVFLGIGIAWMIAACVHAY......................
C.elegans_NCX2           TFASKVSAVQDKY..ADNAVGNVTGSNAVNVFLGIGIAWSMAAIYHWN......................
C.briggsae_NCX2          TFASKVSAVQDKY..ADNAVGNVTGSNAVNVFLGIGIAWSMAAIYHWN......................
T.spiralis               TFASRLAALQDKT..ADNAIGNINGSNSVNVFLGLGLPWFIASVYWAA......................
M.brevicollis_1          TFASRSATLASKT..ADAAVTNVTGSNSVNVFLGLGLSWFIAAVYWPA....MGATDQWRARVPTCLQQK
Salpingoeca              TFASMSATIASDT..ADAAITNVTGSNSVNVFLGLGLSWIIAAIYWPV....VGKTDEWIATVPPCINAK
S.kowalevskii_1          LFASKTAAVNEKY..ADASIGNVTGSNSVNVFLGLGLSWLIASIYWTS....KGL...............
S.kowalevskii_2          LCVSKKASVGEKY..ADCCM...TGSYSVYVFFGLGLSWVTASIYWTS....KGL...............
T.adhaerens_1            LFASKTAALNEKY..ADNSIGNVTGSNSVNVFLGLGLPWLIATIYWNS....QNK...............
D.pulex_1                LFASRAAAMNEKY..ADNAIGNVTGSNSVNVFLGLGLPWLIASIYHAS....NGN...............
P.humanus_corporis       TFASRTAALKEQY..ADTSIGNITGSNSVNVFLGLGLPWLMATIYHTH....NGT...............
O.dioica                 LFASKIAATNEPN..ADDAVGNVTGSNSVNVFLGLGLPWSMAAIYHYC....NGS...............
A.pisum                  ILGACMVTRAETH..ADEAMIHIAGSIAVKVLMGVGLPWFISALYHYS....HGN...............
P.marinus                TFASRLAAMQDPY..ADASIGNVTGSNSVNVFLGLGLPWVIGSLYWS....AKGATQEWIARVGLDIYTK
A.anophagefferens_1      AFASKAATINDDS..ADAAVGNVTGSNSVNVFLGLGLPWSIAAIYWS....GGFAGGS..AEKQWRIKYG
E.huxleyi                TFASRTAAVKERERHADSSIGNITGSNSVNVFLGLGLPWLAAALFWASPRGAAQEASWRDRYRGEAWYSE
C.intestinalis           TIASRTAALQDDY..ADASIGNITGSNSVNVFLGLGLPWVIRTMYFAV.....................K
B.floridae               TFASRTVAIHDVH..ADAAIGNVTGSNSVNVFLGLGLPWVISTMYHLI.....................N
T.adhaerens_3            TFASRNAAVHDHS..ADASIGNVTGSNSVNVFLGLGLPWVLVTTYRQI.....................M
N.vectensis_2            TMASRSAALQDTG..ADAAIGNITGYNSVNVFLGLGLPWVMSTCYHAA.....................R
consensus>50             tfaskvaa.qdqy..ADasignvtGsnaVnVflGiGvaWsiaaiyha.......................
```

```
                              900       910       920       930       940       950       960
H.sapiens_NCX1.1          .NGEQFKVSPGTLAFSVTLFTIFAFINVGVLLYRRRPEI.G.GELGGPRTAKLLTSCLFVLLWLLYIFFS
P.troglodytes_NCX1.1      .NGEQFKVSPGTLAFSVTLFTIFAFINVGVLLYRRRPEI.G.GELGGPRTAKLLTSCLFVLLWLLYIFFS
C.familiaris_NCX1.1       .NGEQFKVSPGTLAFSVTLFTIFAFINVGVLLYRRRPEI.G.GELGGPRTAKLLTSCLFVLLWLLYIFFS
F.catus_NCX1.1            .NGEQFKVSPGTLAFSVTLFTIFAFINVGVLLYRRRPEI.G.GELGGPRTAKLLTSCLFVLLWLLYIFFS
C.porcellus_NCX1.1        .NGEQFKVSPGTLAFSVTLFTIFAFINVGVLLYRRRPEI.G.GELGGPRTAKLLTSCLFVLLWLLYIFFS
B.taurus_NCX1.1           .NGEQFKVSPGTLAFSVTLFTIFAFINVGVLLYRRRPEI.G.GELGGPRTAKLLTSCLFVLLWLLYIFFS
R.norvegicus_NCX1.1       .NGEQFKVSPGTLAFSVTLFTIFAFINVGVLLYRRRPEI.G.GELGGPRTAKLLTSSLFVLLWLLYIFFS
M.musculus_NCX1.1         .NGEQFKVSPGTLAFSVTLFTIFAFINVGVLLYRRRPEI.G.GELGGPRTAKLLTSSLFVLLWLLYIFFS
M.mulatta_NCX1.3          .NGEQFKVSPGTLAFSVTLFTIFAFINVGVLLYRRRPEI.G.GELGGPRTAKLLTSCLFVLLWLLYIFFS
O.cuniculus_NCX1.2        .NGEHFKVSPGTLAFSVTLFTIFAFINVGVLLYRRRPEI.G.GELGGPRTAKLLTSCLFVLLWLLYIFFS
S.tropicalis_NCX1         .NGDVFRVQPGTLAFSVTLFTIFAFINVGVLLYRRRPEI.G.GELGGPRTAKLLTTALFTLLWLLYIFFS
O.mossambicus_NCX1.1      .QGQEFKVNPGTLAFSVTLFTIFAFICIAVLIYRRRPEI.G.GELGGPRIPKILTTCLFFSLWLMYIVFS
O.mykiss_NCX1             .KGNDFRVDPGTLAFSVTLFTIFAFVAVAVLMYRRRPEI.G.GELGGPRGPKIATTCLFFSLWLMYIVFS
D.rerio_NCX1a             .QGRQFRVDPGTLAFSVTLFTIFAFVCIAVLMYRRRPEI.G.GELGGPRHPKIITTTLFFSLWLMYIILS
H.sapiens_NCX3.3          .QGQEFHVSAGTLAFSVTLFTIFAFVCISVLLYRRRPHL.G.GELGGPRGCKLATTWLFVSLWLLYILFA
R.norvegicus_NCX3.3       .QGQEFHVSAGTLAFSVTLFTIFAFVCLSVLLYRRRPHL.G.GELGGPRGCKLATTWLFVSLWLLYVLFA
M.musculus_NCX3.1         .QGQEFHVSAGTLAFSVTLFTIFAFVCLSVLLYRRRPHL.G.GELGGPRGCKLATTWLFVSLWLLYILFA
G.gallus_NCX3             .QGQEFQVSAGTLAFSVTLFTIFAFICISVLLYRRRPHL.G.GELGGPRGCKLATTLLFVSLWLLYILFA
D.rerio_NCX4a             .QGKKFQVPPGSLAFSVTLFTILALVCVVTLLYRRRPSVSG.GELGGPRTPKLLTAFLFIVLWLIYIMLA
T.nigroviridis_NCX4a      .KGKPFKVNPGSLAFSVTLFTIMALVCVLVLLYRRRPSVSG.GELGGPRTAKLVTFFLFISLWFVYILLA
H.sapiens_NCX2.1          .QGRPFEVRTGTLAFSVTLFTVFAFVGIAVLLYRRRPHI.G.GELGGPRGPKLATTALFLGLWLLYILFA
R.norvegicus_NCX2.1       .QGRPFEVRTGTLAFSVTLFTVFAFVGIAVLLYRRRPHI.G.GELGGPRGPKLATTALFLGLWFLYILFA
M.musculus_NCX2.1         .QGRPFEVRAGTLAFSVTLFTVFAFVCIAVLLYRRRPQI.G.GELGGPRGPKLATTALFLGLWFLYILFS
S.kowalevskii_3           .KGEVFEVKPGSLAFSVTIYSIFAVIAITVLMIRRKLPNTL.GELGGPKTTKIITSCFLVGLWLLYLLLC
T.adhaerens_2             .KGEKFYVPSGSLAFSVTIFCAVAIVCIAVLMLRRTKSV.G.GELGGPRGPKYATAVFFIFLWLLYIILS
L.opalescens_NCX          .NGTVFRVDPGTLAFSVTIFCVFAVCTIVLLVCRRHHLV.G.GELGGPPRCKYITSGILGSFWVSYLLLT
A.mellifera_NCX           .HGDKFRVKPGNLAFCVTLFCTEACLVILVLLLRRT.KSIG.GELGGPFVPKVITSLILFFLWVFYLIMS
D.melanogaster_NCX        .HGMTFNVEPGTIGFAVALFCGEALIAIMLIMFRRWHKGIG.AELGGPKVSKYISAAILVFLWVFYVVIC
H.magnipapillata          .NGNKFEVDPGSLGFSVMLFCLEASVCIGILMYRRSSKNIR.AELGGPSISRYITFTTFLLLWIIYLLVS
C.elegans_NCX1            .RGTKFLVATGSLAFSVTMFLIGSVVCVALLQYRRFNRKVN.GELGGPMGWRIISAGIFVSVWLLYILLS
C.briggsae_NCX1           .RGTKFLVSTGSLAFSVTMFLIGSVICVALLQYRRFNRKIN.GELGGPTSWRVISAGIFVSVWLLYILLS
C.elegans_NCX2            .QGTKFLVDPGNLGFSVLIFCTEAVLCIIVLVLRR.NKKVG.GELGGPIALRWMTAMFFASLWFMYLLLS
C.briggsae_NCX2           .KGTKFLVDPGNLGFSVLIFCVEALLCIAVLVLRR.SKKVG.GELGGPTALRWMTSMFFCSLWLMYLLLS
T.spiralis                .RGEAFVVHSGDVGFSVTIYTVLSLICIVMMMLRRFLPALGEAELGGPTIPKWICSVTLILFWFLYILLS
M.brevicollis_1           HDGAFFYVPAGDLAFSVVIFTCCCLVCLGSLLFRR...YKFGAELGGPYSRQ..FAFFFIGLWIFYVLMS
Salpingoeca               YSNAVFWVPSGDLAFSVIIFSLCCIICLGTLVVRR...YTVGAELGGAYAKP..TAALFLTLWLMYILLS
S.kowalevskii_1           ....VFEMEAGALSFSVMIYTICAIMCLSLLVLRRFIPFFGKAELGGPTAGRYSSSIFLILLWIIYILLC
S.kowalevskii_2           ....VFVVDSSSFSFSVMMYIICAIMCLSLLVLRRFTPFFGKAELGGPTAGRYLSSIFLLLLWCLYILLC
T.adhaerens_1             ....EFAVPAGNLASSVILFTICAVICIALLMLRRIIPSFGGSELGGPTKSKYISGLLLVTLWIVYVVIS
D.pulex_1                 ....EFKVEAGSLGFSITVFTITAVLSLALLVTRRCSAACGRGELGGPLGTRYASAIFLLVLWMAYVLLS
P.humanus_corporis        ....VFKVPAGTLGFSVLLFTISAVIAIALLIGRRNSSFCGKAELGGSVFFKLLSGGLLIILWVFYIVMS
O.dioica                  ....TFNVPPGSLVFSVTIFSICAVCTIAFLFLRRTLSYFGNAELGGPQKQKWFSGIFLICLWLFYVLMS
A.pisum                   ....LFEIPSTRIGFNVLLYSVMAILAVTVLMVRRNVEFFGKAELGGPTKGRYITVAILISLWVIYLSLT
P.marinus                 YPDGGFVVEAGDLSFSVMIFTICAFLCLTVLVARR...AKYGGELGGTKHGKRISAAILTYALEVDVTSN
A.anophagefferens_1       GKDVAFIVPAGNLGLSVGVFVFCACTCLGVLAYRR...KAYGAELGGDRTMANRHATLFVMLWFLYVAMS
E.huxleyi                 AMPVAFVVASGDLGFSVAVFTVLALVCLAVLVARR...ATVGGELGGGAASKYSTATLFTALWLGYIWLS
C.intestinalis            GGQ..YIVDTDGLDFAVTLFDSFGAVCVVLLILRR...YVLGGELGGSTRVKWASGIFLFTLWFIFIIVS
B.floridae                GTQ..YRVLPGNLRFAVFTFLAVGSLCLLMLFVRR...KCFGGELGGKKVPKWISALLMFSFWVVFIVLC
T.adhaerens_3             FSNSDYAISPGDMQFSVILFTVVAGAGVAILLFRR...FVLGGELGGDIKWQYITAATLVLLWAVYITLA
N.vectensis_2             GTV..YKVSSGNLAFSVLVFALCGGVCLFLLVLRR...YLYKGELGGPTLSKWMTGLLLLFLWFIYVVLS
consensus>50              .ng..%.v.pg.lafs!tl%ti.a.icv.vllyRR.....g.gELGGpr..k..t..lfv.lwllyi.ls
```

```
                                970
H.sapiens_NCX1.1       SLEAYCHIKGF................
P.troglodytes_NCX1.1   SLEAYCHIKGF................
C.familiaris_NCX1.1    SLEAYCHIKGF................
F.catus_NCX1.1         SLEAYCHIKGF................
C.porcellus_NCX1.1     SLEAYCHIKGF................
B.taurus_NCX1.1        SLEAYCHIKGF................
R.norvegicus_NCX1.1    SLEAYCHIKGF................
M.musculus_NCX1.1      SLEAYCHIKGF................
M.mulatta_NCX1.3       SLEAYCHIKGF................
O.cuniculus_NCX1.2     SLEAYCHIKGF................
S.tropicalis_NCX1      SLEAYCHIKGF................
O.mossambicus_NCX1.1   SLEAYCHVKGF................
O.mykiss_NCX1          SLEAYCHVKGF................
D.rerio_NCX1a          SMEAYCIIKGF................
H.sapiens_NCX3.3       TLEAYCYIKGF................
R.norvegicus_NCX3.3    TLEAYCYIKGF................
M.musculus_NCX3.1      TLEAYCYIKGF................
G.gallus_NCX3          TLEAYCYIKGF................
D.rerio_NCX4a          SLEAYCHVPGF................
T.nigroviridis_NCX4a   SLEAYCHVHSF................
H.sapiens_NCX2.1       SLEAYCHIRGF................
R.norvegicus_NCX2.1    SLEAYCHIRGF................
M.musculus_NCX2.1      SLEAYCHIRGF................
S.kowalevskii_3        SFESYCYIEGF................
T.adhaerens_2          SLESYCHIPGF................
L.opalescens_NCX       GLMSYCHIPGF................
A.mellifera_NCX        ILEAYGYIEGF................
D.melanogaster_NCX     ILEAYDVIRV.................
H.magnipapillata       GLESYCYFTLKF...............
C.elegans_NCX1         TLEAYCIIKGF................
C.briggsae_NCX1        TLEAYCVIKGF................
C.elegans_NCX2         ALEAYCIIPGF................
C.briggsae_NCX2        ALEAYCIIPGF................
T.spiralis             ALQAYGHISSF................
M.brevicollis_1        SLQVEGYIASI................
Salpingoeca            SLSTYEHISL.................
S.kowalevskii_1        SLQAYGYIHF.................
S.kowalevskii_2        SFQSYDYIDF.................
T.adhaerens_1          SLQVYRIIPPIVKT.............
D.pulex_1              SLETYGHITGF................
P.humanus_corporis     CLKEYRVIDVNF...............
O.dioica               TLQAYEFIPGF................
A.pisum                CFQPF......................
P.marinus              GVALNSNLHLNYRDASDVDRGEY....
A.anophagefferens_1    ILASEGIV...................
E.huxleyi              IAKTEGDPRLARLLQQLPPQARALLGA
C.intestinalis         SLKAYNYF...................
B.floridae             SLKAYDLLPF.................
T.adhaerens_3          SLRAYDVIRFQ................
N.vectensis_2          SLMAYGHISGSL...............
consensus>50           sleayc.i.gf................
```
