# Supplementary material for: Phylogeny and a structural model of plant MHX transporters
Source: BMC Plant Biol. 2013 May 2;13:75. doi: 10.1186/1471-2229-13-75 (PMC3679957; doi:10.1186/1471-2229-13-75)
Supplement: Additional file 8 — Alignment of angiosperm MHX proteins. [file 1471-2229-13-75-S8.pdf]

Additional file 8. Alignment of angiosperm MHX proteins

The file presents the alignment of all currently identified angiosperm MHX proteins. See the legends of Additional file 5 for details of sequence alignment and color annotation.

```
                                                1         10        20        30        40
A.thaliana      .......................MASILNQTQELQESSKVLGHLRCENFFLFPGENTLSDGLRGVLYFLG
A.halleri       .......................MASILNQTQELQEASKVLGHVRCENFFIFPGENTLSDGLRGVLYFLG
A.lyrata        .......................MASILNQTQELQEASKVLGHVRCENFFIFPGENTLSDGLRGVLYFLG
C.clementina    ......................MASIHTQAIESISRAFNILHYEKCESYLLFRGETSLGDGFRAFLYFLG
R.communis      .....................MALETNYQSGWSRFGLLNILASEKCESYLVFRGETGLSVGFRTFLYFLG
M.esculenta     .....................MALETQNQSAGSKFGLLNILGRERCESYLLFHGETALDNGFRTFLYFLA
P.trichocarpa   ......................MASAYNQSDGGRFGDTNIVGHEKCESYFLFRGETTLGEGFRTFLYFLG
P.persica       .................................MPGNSSTMGQENCESYFIFQGETGLGVGFRTFLYFLG
C.papaya        ......................MTWAHWRNAESIIQNFNVLEQEKCESYLIFRSETALSNGFRAFLYFLG
S.tuberosum     ......................MTSLGNYTTDSTNGHSNILRHEKCDAYLLIHLETALGEGFRTFLYFLG
S.lycopersicum  ......................MTSLGNYTTHSTNGHSNILRHEKCDAYLLFHLETALGGGFRTFLYFLG
M.guttatus_1    ......................MDPLNNSTAEIIDGGSNILGVEKCQLYLLFHDETVLSDGFRGFLYFMA
C.sativus       ......................MASFAIENVESGQAISSISGTGKCESYFIFSIETSLGDALRIFLYFMG
G.max           .................................MVPFSNILGHEKCESYLIFGGETTLGDNFRAFFYFLC
M.truncatula    ............................MYKMIGYLSSFLAHDEKCESYLIFSGETNLGNSVRILLYFLG
A.coerulea      ............................................KCESYFLFQGEMALGNGLRAFLYLLG
V.vinifera      ......................MAWVHEQLPGNILEAYNISVREKCESYLLFSGETSLGDGFRTFLYFLG
E.grandis       MQACFASVRLSLSSSLDSLPIFSVSFRRAGTPGDISRSHMMGHGVCESYLLFPGETALGDAFRTFLYFLG
M.guttatus_2    ...........................MASLNSSSTWDSNLGHERCLFYFIFHFETKLSHGLRGFLYFLA
O.sativa_J1     .................................MANINMADTVPSCDTYLLFNGETLLPIGVRAFIYTAV
O.sativa_I1     .................................MANINMADTAPSCDTYLLFNGETLLPIGVRAFIYTAV
O.sativa_J2     .................................MANINMADTAPSCDTYLLFNGETLLPNGVRAFIYTVV
T.aestivum      ......................................MGSTTRSCDAYLLFNGETLLPNGVRAFLCTVA
B.distachyon    .................................MANISMGSNAPSCNAYLLFYAEKMLPNGVRAFAYTVA
Z.mays          ..........................MAGAATPPPSSSSSASCGTDAAYLLFRGETLLPDGVHASLYAVA
S.bicolor       .......................MSNIAMVAAPPSSSCDSDT......YLLFHGETLLSTGARASLYTVA
S.italica       .....................................MSGPSSACEGTYLLFHGETLLSGGVRASLYTVA
consensus>50    .......................................il....ce.%llf.gEt.L.dg.r.flyflg
```

```
                50        60        70        80        90        100       110
A.thaliana      LAYCFIGLSAITARFFKSMENVVKHSRKVVT.IDPITKAEVITYKKVWNFTIADISLLAFGTSFPQISLA
A.halleri       LAYCFIGLSAITARFFKSMENVVKHSRKVVA.IDPITKAEIITYKKVWNFTIADISLLAFGTSFPQISLA
A.lyrata        LAYCFIGLSAITARFFKSMENVVKHSRKVVA.VDPITKAEVITYKKVWNFTIADISLLAFGTSFPQISLA
C.clementina    LAYCFIGLSAITARFFRSMENVVKHSRKVVE.IDPVTKAEVVRYEKVWNYAIADIALLAFGTSFPQISLA
R.communis      LAYCFIGLSAITGRFFRSMENVVKHSRKVVE.IDPYSNTEVIRYEKVWNYTIADISLLAFGTSFPQISLA
M.esculenta     LAYCFFGLSAITARFFRSMENVVKHSRKIVE.VDPDTNTEVIRYEKVWNYAIADISLLAFGTSFPQISLA
P.trichocarpa   LAYCFIGLSAITARFFRSMENVVKQSRKVVE.IDPYTNAEVIRYEKVWNYAIADISLLAFGTSFPQISLA
P.persica       LAYCFVGLSAITARFFQSMESVVSHTRKVVD.INPYTGAEIIRYEKVWNFTIADISLLAFGTSFPQISLA
C.papaya        IAYCFLGLSAITGRFFRSMEHVVKHTREVVE.IDPITNQEITRHEKVWNYTIADISLLAFGTSFPQISLA
S.tuberosum     LAYCFIGLSAITARFFRSMESVVKHSRTVET.IDPLTNTKIVKNEKVWNYTIADITLLAFGTSFPQISLA
S.lycopersicum  LAYCFIGLSAITARFFRSMESVVKHSRTVET.IDPLTNTKAVKNEKVWNYTIADITLLAFGTSFPQISLA
M.guttatus_1    LAYCFIGLSAITSRFFQSMENVVKHTRTVEE.IDPCTNTKTVRHEKVWNYTIADITLLAFGTSFPQISLA
C.sativus       LAYCFVGLSAITARFFRSMENVVKHSRKVVE.IDPHTNTEIIRYEKVWNFTIADISLLAFGTSFPQISLA
G.max           LAYCFIGLSAITARFFQSMENVVKHTRKVVE.VDPVTKTETIRHEKVWNYTIADISLLTFGTSFPQISLA
M.truncatula    LAYCFIGLSAITSRFFQSMENVVKHSREVVV.IDPVTKAETIRHEKVWNYTIADISLLAFGTSFPQISLA
A.coerulea      LAYCFFGLSAITARFFRSMENVVKHSRKVVE.IDHNTNTEVIRHEKVWNYAIADITLLAFGTSFPQISLA
V.vinifera      LAYCFIGLSAITARFFQSMENVVKHKRKVVK.IDPRSNTE.IRHEKVWNYTIADITLLAFGTSFPQISLA
E.grandis       LAYCFIGLSAITARFFSSMENVVKQTREVVE.IDPQTNSKVVRQEKVWNYTIADITLLAFGTSFPQISLA
M.guttatus_2    LAYCFVGLSAITDRFFRSMENVVKQTRAVEE.IDPLTNTKVIKYEKVWNYTIADITLLAFGTSFPQISLA
O.sativa_J1     LAYCFIGLSAITGRFFKSMESIMRHSREVVT.VDPHTNATIVKHEKVWNYTIADVALLAFGTSFPQISLA
O.sativa_I1     LAYCFIGLSAITGRFFKSMESIMRHSREVVT.VDPHTNATIVKHEKVWNYTIADVALLAFGTSFPQISLA
O.sativa_J2     LAYCFIGLSAISGRFFKSMESIMRHSREVVT.IDPHTNATIVKHEKVWNYTIADVALLAFGTSFPQISLA
T.aestivum      LAYCFIGLSAITARFFKSMESITNHSREVVT.VDTETNTPIVKHEKVWNYTIADIALLAFGTSFPQISLA
B.distachyon    LGYCFIGLSAITARFFKSMESITNHSREVVT.IDPHTNTPIVKHEKVWNYTIADIALLAFGTSFPQISLA
Z.mays          LAYCFVGLSAVTARFFKSMEQIMRHSREVVVGVDPRTGAPVVRRDKVWNYTVADIALLAFGTSFPQISLA
S.bicolor       LAYCFIGLSAITARFFKSMEQIMKHSREVVVI.DPHTKEPVVKHEKVWNYTVADIALLAFGTSFPQISLA
S.italica       LAYCFIGLSAITARFFKSMEQIMKHSREVVS.VDPHTNAPVVKQEKVWNYTIADIALLAFGTSFPQISLA
consensus>50    laYCFiGLSA!taRFF.SMEn!vkhsR.!v..i#p.tn.evir.eKVWN%t!AD!.LLaFGTSFPQISLA
```

```
                120       130       140       150       160       170       180
A.thaliana      TIDAIRNMGERYAGGLGPGTLVGSAAFDLFPIHAVCVVVPKAGELKKISDLGVWLVELVWSFWAYIWLYI
A.halleri       TIDAIRNIGERYAGGLGPGTLVGSAAFDLFPIHAVCVVVPKAGELKKISDLGVWLVELVWSFWAYIWLYI
A.lyrata        TIDAIRNIGERYAGGLGPGTLVGSAAFDLFPIHAVCVVVPKAGELKKISDLGVWLVELVWSFWAYIWLYI
C.clementina    TIDAIRSIGNLYAGGLGPGTLVGSAAFDLFPIHAVCVVVPKAGELKKISDIGVWLVELFWSFWAYVWLYI
R.communis      TIDAIRNIGNLYAGGLGPGTLVGSAAFDLFPIHAVCVVVPKAGELKKISDLGVWLVELFWSFWAYVWLYI
M.esculenta     TIDAIRNIGNLYAGGLGPGTLVGSAAFDLFPIHAVCVVAPKAGELKKIADLGVWIVELFWSFWAYIWLYI
P.trichocarpa   TIDAIQNIGNLYAGGMGPGTLVGSAAFDLFPIHAVCVVVPKAGELKKIADIGVWLVELFWSFWAYIWLYI
P.persica       TIDAIRNLGNLYAGGLGPGTLVGSAAFDLFPIHAVCVVVPRAGELKKIADVGVWLVELFWSFWAYIWLYI
C.papaya        TIDAIRNLGNLNAGGLGPGTLVGSAAFDLFPIHAVCVVVPKGGEMKKIADIGVWLVELFWSFWAYIWLYI
S.tuberosum     TIDAIRNIGKLYAGGLGPGTLVGSAAFDLFPIHAVCVVVPKAGELKKISDIGVWLVELFWSFWAYIWLYI
S.lycopersicum  TIDAIRNIGKLYAGGLGPGTLVGSAAFDLFPIHAVCVVVPKAGELKKISDIGVWLVELFWSFWAYIWLYI
M.guttatus_1    TIDAIRNLGSLYAGGLGPGTLVGSAAFDLFPIHAVCVVVPKAGELKKISDVGVWLVELFWSFWAYIWLYI
C.sativus       TIDAIRNIGNLYAGGLGPGTLVGSAAFDLFPIHAVCVVVPKAGELKKISDIGVWLVELVWSFWAYIWLYI
G.max           TIDAIQNIGKLYAGGLGPGTLVGSAAFDLFPIHAVCVVVPKAGELKKIADLGVWLVELFWSFWAYIWLYI
M.truncatula    TIDAIRNLGDLYAGGLGPGTLVGSAAFDLFPIHAVCVVIPKAGELKKIADLGVWIVELFWSFWAYIWLYI
A.coerulea      TIDAIRNLGQIYAGGLGPGTLVGSAAFDLFPIHAVCVVVPKAGELKKISDIGVWLVELFWSFWAYIWLYI
V.vinifera      IIDSIQNLGSRYAGGLGPGTLVGSAAFDLFPIHAVCVVVPKAGELKKISDVGVWLVELFWSFWAYVWLYI
E.grandis       TIDALRNLGELYAGGLGPGTLVGSAAFDLFPIHAVCVVVPKSGELKKISDLAVWAVELFWSFWAYIWLYI
M.guttatus_2    TIDAIRNIGSLTAGGLGPGTLVGSAAFDLFPIHAVCVVVPKAGELKRISDIGVWLVELFWSFWAYIWLYI
O.sativa_J1     TIDAIRNLGQLTAGGLGPGTLVGSAAFDLFPIHAVCVVMPRAGSKKKISDLGVWLVELFWSFWAYIWLYI
O.sativa_I1     TIDAIRNLGQLTAGGLGPGTLVGSAAFDLFPIHAVCVVMPRAGSKKKISDLGVWLVELFWSFWAYIWLYI
O.sativa_J2     TIDAIRNLGQLTAGGLGPGTLVGSAAFDLFPIHAVCVVMPRAGSKKKISDLGVWLVELFWSFWAYIWLYI
T.aestivum      TIDAIRNLGQLTAGGLGPGTLVGSAAFDLFPIHAVCVVMPRAGSMKKISDLGVWLVELFWSFWAYIWLYI
B.distachyon    TIDAIRNLGQLTAGGLGPGTLVGSAAFDLFPIHAVCVIMPRAGSMKKISDLGVWLVELFWSFWAYIWLYI
Z.mays          TIDAIRNLGQLTAGGLGPGTLVGSAAFDLFPIHAVCVLMPRAGSKKKISDLGVWLVELFWSFWAYIWLYV
S.bicolor       IIDAIRNLGQLTAGGLGPGTLVGSAAFDLFPIHAVCVVMPRAGSKKKISDLGVWLVELVWSFWAYIWLYV
S.italica       TIDAIRNLGQLTAGGLGPGTLVGSAAFDMFPIHAVCVIMPKAGSKKKITDLGVWLVELFWSFWAYIWLYV
consensus>50    tIDairnlGqlyAGG$GPGTLVGSAAFD$FPIHAVCVvvPkaGelKkIsDlgVWlVELfWSFWAY!WLY!
```

```
                  190       200       210       220       230       240       250
A.thaliana        ILEVWSPNVITLVEALLTVLQYGLLLVHAYAQDKRWPYLSLPMSRGDRPEEWVPEEIDTSKDDNDNDVHD
A.halleri         ILEVWSPNVITLVEALLTVLQYGLLLVHAYAQDKRWPYLSLPMSRGDRPEEWVPEEIDTSKDDNDNDVHD
A.lyrata          ILEVWSPNVITLVEALLTVLQYGLLLVHAYAQDKRWPYLSLPMSRGDRPEEWVPEEIDTSKDENDNDVHD
C.clementina      ILEVWTPNVITFWEALLTVLQYGLLLIHAYAQDKRWPYLSLPIARTERPEDWVPQETTSCKDENCDYDEC
R.communis        ILEVWTPNVITLWEALLTVLQYGLLLTHAYAQDKRWPYLSLPIERTERPEEWVPEEATSDKHQHNAYEEY
M.esculenta       ILEVWTPNVITLWEALVTVLQYGLLLIHAYAQDKRWSYLSLPLGRTERPKEWVPEEITSSKHQPIDYEEY
P.trichocarpa     ILEVWTPNVITLWEALLTVLQYGLLLMHAYAQDKRWPYLSLPIPRTERPEEWVPGEVPSPTHENNVYGE.
P.persica         ILEVWTPNVITIWEALLTVLQYGLLLMHAYAQDKRWPYFSLPLPRSERPEDWVPAEVVTCKSDSGPCNNY
C.papaya          ILEVWTPNVVTLWEALLTVLQYGLLLTHAYAQDKRWPYLSLPIGRTERPEDWVPEEVASVDHDNNDKF..
S.tuberosum       ILEVWTPNVVTLWESILTVLQFGLLLIHAYAQDKRWPYLSLPLERAERPEEWVPAEVVKHRPLDKVHEPH
S.lycopersicum    ILEVWTPNVVTLWESILTVLQFGLLLIHAYAQDKRWPYLSLPLERAERPEEWVPAEVVKYRPLDKVHEPH
M.guttatus_1      ILEVWTPNIITLLEALLTVLQFGLLLIHAYAQDKRWPYLSLPIGRTERPEEWVPVETAPYKDRDKYSEIP
C.sativus         ILEVWTPKVITLWEALLTVLQYGLLLTHAYAQDKRWPYLSLPLARTERPEEWVPPEIDICKQDNPCREEF
G.max             ILEVWTPNVVTLWEALLTVLQYGLLLTHAYAQDKRWPYISLPIARDERPEDWVPEETPYFQHEAHAEVDF
M.truncatula      ILEVWTPNVITLWEALLTVLQYGLLLIHAYAQDKRWPYISLPIARDERPEDWVPEETPKQKS..HERVEC
A.coerulea        ILEVWTPNVVTLWEALITVLQFGLLLIHTYAQDKRWPYLSLPLARTERPEDWVPVEASLDKLDNNDYSEI
V.vinifera        ILEVWTPNVITIWEALLTVLQFGLLLIHAYAQDKQWPYLSLPLPRTERPEDWVPDEAASRKQDKIAGDEC
E.grandis         ILKVWTPDIVTLWEALLTVLQFGLLLVHAYAQDKRWPYLSIPLARGERPEEWVPEEAASYKHVDN.....
M.guttatus_2      ILKIWTPNVITLWEALLTVAQFGLLLIHAYAQDKRWPYVSLPMTRSERPEDWVPAEKNAPYRDGNKTR..
O.sativa_J1       ILEVWTPRVITLWEALLTVLQYGLLLLHAYAQDKRWPYVSIPLARGERPEDWVPAEDASVDYDDNYD...
O.sativa_I1       ILEVWTPRVITLWEALLTVLQYGLLLLHAYAQDKRWPYVSIPLARGERPEDWVPAEDASVDYDDNYD...
O.sativa_J2       ILEVWTPRVITLWEALLTVLQYGLLLLHAYAQDKRWPYVSIPLARGDRPEDWVPTEDASVDYDDNYD...
T.aestivum        ILEVWTPNVITLWEALLTVLQYGLLLVHAYAQDKRWPYVSIPLVRGERPEDWVPAEDTSLHHDKNCD...
B.distachyon      ILEVWTPNVITLWEALLTVLQYGLLLVHAYAQDKRWPYVSIPLVRGERPEDWVPAEDTSVDHDKNCG...
Z.mays            ILEVWTPRVITLWEASLTVLQYGLLLLHAYAQDKRCPFVSIPFARGERPEDWVPPEDVSAVYYAKCD...
S.bicolor         ILEVWTPKVITLWEALLTVLQYGLLLLHAYAQDKRWPFVSIPFVRGERPEDWVPPEDVS.VDYDNCD...
S.italica         ILEVWTPKVITVWEALLTVLQYGLLLLHAYAQDKRWPYVSIPLVRGDRPEDWVPEEGAS.VDYDNCN...
consensus>50      ILe!WtPn!!TlwEallTVlQ%GLLLiHaYAQDKrwp%lSlPl.R.#RPe#WVP.E..s...ddn.....

                  260       270       280                 290       300       310
A.thaliana        VYSDAAQDAVESGSRNIVDIFSIHSAN.......NDTGITYHTVADTPPDSATKKGKAKNSTVFDIWKHQ
A.halleri         VYSDAAQEAVESGSRNIVDIFSIHSAN.......NDTGITYHTVADTPPDSATKKGKAKNSSVFDIWKHQ
A.lyrata          VYSDAAQEAVESGSRNIVDIFSIHSAN.......NNTGITYHTVADTPPDSATKKGKAKNSTVFGIWKHQ
C.clementina      SEIL...QLGEDENRGVVDIFSIHSPNGAVTSPLYQKVPGSEDVAEISKESFREEINSELPHVHALWKQQ
R.communis        SEIV...QVSEEDSRNIVDIFSIHSPVG..TDPVYQKVPETDEAAESSNNYSLSEKDL...DVVALWKQQ
M.esculenta       SEIL...QVDKDESRNVVDIFSIHSNVE..TDQVYHKVPETDDAVEFSDKNFHSEQDF...NVAALWKRQ
P.trichocarpa     ............ENRNVVDIFSIHSNNG..T..VYQKVPVTDDAAESSNKHFHQEKDL...HVLSLWKEQ
P.persica         SEIL...EVGEDENRNIVDIFSFHSGSGLG..PVYQNVPGTDETPEYSNKDSPEKMSLEDYHVFAIWGQQ
C.papaya          ................VVDIFSIHSAP....STEYQKVPADENIPEPSGKSSRKEILLEDPNVLTLWKQQ
S.tuberosum       SEVS...QVGEEENSGIVDIFSIHSGEGTGHFYRNLAGEDVTESSTPNNGNIIPEESDI....LSIWKHQ
S.lycopersicum    SEVS...QVGEEENTGIVDIFSIHSGEGTGHFYQNLAGEDVTESSTPNNCYIIPEESDI....LSIWKHQ
M.guttatus_1      EDDH...ESGR.....IVDIFSVHSD....HVYQNLSGSDSGEPSGQSHEDIHVKDNLI.....SIWKQQ
C.sativus         .......QAHENEQRSIVDIFSIHDSDGKVYHEVPGHDIAE...SSNSNIPEEMDGKADHPHVLKIWKQQ
G.max             SDIK...HVNE.ENGDTVDIFSIHSENPTDPLYARVPSIND....EAEILDKAKETTLVDTHLLTIWRQQ
M.truncatula      SEIN...HFNE.ENGDTVDIFSIHSENPRDMSYVRVPQIDD.....AENSDKVIETRLEDTCLLTIWKQQ
A.coerulea        LQLS...E....ED.NIVDIFSIHSEKETGPLYQPVSNSDV...AESSNMNFHNKSVLQDFNVFTVWKHQ
V.vinifera        SELF...Q....ENGNIVDIFSIHSGD..GSVYHRISGSEV...AEPSNEHFQKNIILEDSHLLSLWKQQ
E.grandis         ........................................VDGPMYHNVPASDLAESSNELHVLALWKEQ
M.guttatus_2      ...DNYPEVDEDRNSIIVDIFSIHSANTGFVYE....NVADTDIHESSSEQCRVDTIPHKDDLLSVWKMQ
O.sativa_J1       .GIGDILPGQ...NEDIVDIFSAHSYSNEG.Y....HHVSEEDVEESS.TGLTLKNKWEDTHWFSIWWQQ
O.sativa_I1       .GIGDILPGQ...NEDIVDIFSAHSYSNEG.Y....HHVSEEDVEESS.TGLTLKNKWEDTHWFSIWWQQ
O.sativa_J2       .GIGDILPGQ...NEDIVDIFSARSYSNEG.Y....HHVSEKDVEESP.TGLTLKNKWEDTHWFSIWWQQ
T.aestivum        .ENSDILPSE...NDDVVDIFSIHSYSNAG.Y....HHVPEKDIEESSKTTLVVKNTQEDIYWLSVWRQQ
B.distachyon      .ESSEILPRE...NEDIVDIFSMHSYNNTG.Y....HHVPEKDIEESSKTTLVVKNTQEDTCWLSIWRQQ
Z.mays            .GIDETLPMGADGNDGIVDIFSAHSYYDAAEY....SQLAEEDMEGSSTMDQVVKNTQDNMSWLSIWWQQ
S.bicolor         .DTNETLPISANRNDGIVDVFSAHSYHIA.EY....SRVPEKDMEGSSTMDQVVKNTQEDISWLSIWWQQ
S.italica         .ETSEILPGSSDKD..IADIFSGHSYHNA.EY....HKVPENDMECSSTMNNFVKNTREDTSWRSLWRQQ
consensus>50      .e.....q.....n.nivdifsihs............v...d..e.s......k...ed...lsiWkqQ

                320       330       340       350       360       370       380
A.thaliana        FVDAITLETSESKKVDSIYLRIAKSFWHLLLAPWKLLFAFVPPCNIAHGWIAFICSLLFISGVAFVVTRF
A.halleri         FVDAITLETSESKKVDSIYLRIANSFWQLLLAPWKLLFAFVPPCNIAHGWIAFIFSLLFISGVAFVVTRF
A.lyrata          FVDAITLETSESKKVDSIYLRIAKSFWQLLLAPWKLLFAFVPPCNIAHGWIAFICSLLFISGVAFVVTRL
C.clementina      FVDSIALESPESRKMNNIYLRLARIFWQSLLVPWRVLFAFVPPYHIAHGWIAFICSLIFISGIAYIVTKL
R.communis        FVDAIMLERPESRKLINSHLRLARISWQLFLVPWRLLFAFVPPYHIAHGWIAFICSLLFISGIAYIVTQL
M.esculenta       FLDAITLESLESRNMFSIHIRLARTFWEILLAPWRLLFAFVPPYHIAHGWVAFICSLIFISGIAYIVTKI
P.trichocarpa     FVDALTLESLETRKLNSIHLRVARISWQLILAPWRLLFAFVPPYHFAHGWIAFIFSLLFISGIAYIVTKL
P.persica         FVDAVKLKSTESRQLNNLYLRLARISWQLLLVPWRLLFAFVPPYHIAHGWIAFICSLVFISAIAYIVTQL
C.papaya          FVDIFKVEKSDSRKLDNIYLQLARIVWQSLLAPWRFMFAFVPPYHISHGWIAFVCSLLFISGISYIVTKL
S.tuberosum       FVDALMLESTESRKLNNIYLRVARIFWQLLLLPWKLLFAFVPPYQIAHGWIAFICSLIFISGIAYVVTKI
S.lycopersicum    FVDALMLESTESRKLNNTYLRVARIFWQLLLLPWKLLFAFVPPYQIAHGWIAFICSLIFISGIAYVVTKI
M.guttatus_1      FVDAFVLEGQESRKLNNTYLRAAKVLWELLLAPWRILFAFVPPCQIAHGWISFISSLIFISGIAYVVTKL
C.sativus         FVDALSLETSESKQRNNIYLRSARLCWQLIVAPWRLLFAFVPPYHIAHGWVAFICSLMFISGIAYVLTKF
G.max             FLDALRLLRPESKKIKNACLRLACIFWQLLILPWRFLFAFVPPCQIAHGWISFICSLLFISGIAYVVTKI
M.truncatula      FVDALTVESQESKKMNNIYIRTARIFWQLLLLPWRFMFAFVPPCHIAHGWISFICSLLFISGIAYIVTKI
A.coerulea        FMDAVMLENPESRKLDNTYLRLARGTWQVILAPWRVLFAFVPPCHIAHGWIAFICSLIFISGIAYIVTKL
V.vinifera        FVDAFTLESSESRKLDNIYLWVARIFWQLLLLPWRFLFAFVPPPHIAHGWFAFICSLIFISGIAYIVTKL
E.grandis         FVDALMLESAETKKLYSIYVRALRMLWQSLLAPWRLMFALVPPCQIAHGWFAFICSLTFISGIAYVVTKL
M.guttatus_2      FVDALTLESPESKKLNSKSLRVAKALWKLVLAPWRLLFAFVPPYQIANGWIAFIFSLVFISGIAYIVTKI
O.sativa_J1       FVDAATLESSVSRKMDSTCLRVIGISWNLIIAPWKMLFAFVPPYEIAHGWIAFICSLIFISGIAYGVTKI
O.sativa_I1       FVDAATLESSVSRKMDSTCLRVIGISWNLIIAPWKMLFAFVPPYEIAHGWIAFICSLIFISGIAYGVTKI
O.sativa_J2       FVDAATLESSVSRKMDSTCLSVIGISWNLIIAPWKMLFAFIPPYEIAHGWIAFICSLIFISGIAYGVTKI
T.aestivum        FVDAVMLESPELKKMDPVCLRFIIICWNSIIAPWKLLFAFVPPYQIAHGWIAFIFSLIFISGIAYGVTNI
B.distachyon      FVDALMLESPEQRKMDSICLRFIIIFWNSIIAPWKLLFAFVPPYQIAHGWIAFICSLIFISGIAYGVTKI
Z.mays            FVDASMLESPESMKMDSVCLRYTRIFWNLIIAPWKFLFAFVPPYHIAHGWIAFICSLIFISGIAYGVTKL
S.bicolor         FVDASMLESPESRKMHSICLRFTTIFWNLIIAPWKFLFAFVPPYNIAHGWIAFICSLIFISGIAYGVTKL
S.italica         FVDAFMLESPESRKMASVCLRLIRIFWNLLIAPWKLFFAFVPPYHIAHGWVAFICSLIFISGIAYGVTKL
consensus>50      FvDa..les.esrkmdsiylr.arifWqlllaPWrllFAf!PPyqiahGWiaF!cSLiFISg!a%ivTk.
```

```
                390       400       410       420       430       440       450
A.thaliana      TDLISCVTGINPYVIAFTALASGTSWPDLVASKIAAERQLTADSAIANITCSNSVNIYVGIGVPWLINTV
A.halleri       TDLISCVTGINPYVIAFTALASGTSWPDLVASKIAAERQLTADSAIANITCSNSVNIYVGIGVPWLINTV
A.lyrata        TDLISCVTGINPYVIAFTALAGGTSWPDLVASKIAAERQLTADSAIANITCSNSVNIYVGIGVPWLINTV
C.clementina    TDIISCVTGINAYVIAFTALASGTSWPDLVASKIAAERQITADSAIANITCSNSVNIYVGIGIPWLIDTA
R.communis      TDLISCVTGINAYVIAFTALAAGTSWPDLVASKIAAERQITADSAIANITCSNSVNIYVGIGVPWLIDTA
M.esculenta     TDLISCVTGINAYVIAFTALASGSSWPDLVASKIAAERQTTADSAIANITCSNSVNIYVGIGVPWLIDTT
P.trichocarpa   TDVISCVTGINAYVIAFTALASGTSWPDLVASKIAAERQTTADSAIANITCSNSVNIYVGIGVPWLIDTA
P.persica       TDLISCVTGINPYVIAFTALASGTSWPDLVASKIAAERQITADSAIANITCSNSVNIYVGIGVPWLIDTA
C.papaya        TDLVSCVTGINPYVIAFTALACGTSWPDLVASKIAVERQITADSAIANITCSNSVNIYVGIGVPWLINTL
S.tuberosum     TDLISCVTGINPYVIAFTALASGTSWPDLVASKIAAERQLTADSAIANITCSNSVNIYIGIGVPWLINTL
S.lycopersicum  TDLISCVTGINPYVIAFTALASGTSWPDLVASKIAAERQLTADSAIANITCSNSVNIYIGIGVPWLIDTL
M.guttatus_1    TDIISCVTGINAYVIAFTALAAGTSWPDLVASKIAAERQTTADSAIANITCSNSVNIYVGIGVPWLINTM
C.sativus       TDLISCVSGINPYVIAFTALASGTSWPDLVASKIAAERQTTADSAIANITCSNSVNIYVGIGVPWLISTT
G.max           TDVISCVTGINAYVIAFTALASGTSWPDLVASKIAAERQKTADSAIANITCSNSVNIYVGIGVPWLIDTL
M.truncatula    TDLISCVTGINAYVIAFTALASGTSWPDLVASKIAAKRQKTADSAIANITCSNSVNIYVGIGVPWLIDTL
A.coerulea      TDMISCVTGINGYVIAFTALASGTSWPDLVASKIAAERQTTADSAIANITCSNSVNIYIGIGIPWLIDTT
V.vinifera      TDLISCTSGINSYVIAFTALAAGTSWPDLVASKIAAERQTTADSAIANIICSNSVNIYMGIGIPWLINTT
E.grandis       TDLISCVTGINAYVIAFTALATGTSWPDLVASKIAAERQTTADSAIANITCSNSVNIYVGIGVPWLINTA
M.guttatus_2    TDLISCVTGINAYIIALTALAAGTSWPDLVASKIAAERQITADSAIANITCSNSVNIYIGIGVPWLIDTL
O.sativa_J1     TDQISCVTGVSPYVIAFTALAAGTSWPDLVASKIAAERQITADSAITNITCSNSVNIYVGIGVPWLVDTM
O.sativa_I1     TDQISCVTGVSPYVIAFTALAAGTSWPDLVASKIAAERQITADSAITNITCSNSVNIYVGIGVPWLVDTM
O.sativa_J2     TDQISCVTGVSPYVIAFTALAAGTSWPDLVASKIAAERQITADSAIANITCSNSVNIYVGIGVPWLVDTM
T.aestivum      TDQISCVTGLNPYVIAFTALAAGTSWPDLVASKIAAERQVTADSAIANITCSNSVNIYVGIGVPWLINTV
B.distachyon    TDQISCVTGVNLYVIAFTALAAGTSWPDLVASKIAAERQVTADSAIANITCSNSVNIYVGIGVPWLIDTV
Z.mays          TDQISCVTGVSPYVIAFTALAAGTSWPDLVASKIAAERQVTADSAIANITCSNSVNIYVGIGVPWLIDTV
S.bicolor       TDQISCVTGVSPYVIAFTALAAGTSWPDLVASKIAAERQVTADSAIANITCSNSVNIYVGIGVPWLIDTV
S.italica       TDQISCVTGVSPYVIAFTALAAGTSWPDLVASKIAAERQVTADSAIANITCSNSVNIYVGIGVPWLIDTV
consensus>50    TDl!SCvtGinpY!IAfTALA.GtSWPDLVASKIAaeRQ.TADSAIaNItCSNSVNIYvGIG!PWL!dT.

                460       470       480       490       500       510       520
A.thaliana      YNYFAYREPLYIENAKGLSFSLLIFFATSVGCIVVLVLRRLIIGAELGGPRLWAWLTSAYFMMLWVVFVV
A.halleri       YNYFAYREPLYIENAKGLSFSLLIFFATSVGCIVVLVLRRLIIGAELGGPRLWAWLTSAYFMMLWVVFVV
A.lyrata        YNYFAYREPLYIENAKGLSFSLLIFFATSVGCIVVLVLRRLIIGAELGGPRLWAWLTSAYFMMLWVVFVV
C.clementina    YNFIVYKEPLRVQNAEGLSFSLLVFFCTSVGCIAVLVLRRLTLGAELGGPKLWAWVTSVYFMLLWIIFVV
R.communis      YNFFAYREPLRVQDAAGLSFSLLVFFSTSVGCIAVLVYRRLTLGAELGGPRIWAWVTSVYFMFLWLIFVV
M.esculenta     YNFFVYRQPLRIENAEGLSFSLLVFFATSVGCIGVLVYRRLTLGAELGGPRIWAWVTCVYFMLLWLIFVV
P.trichocarpa   YNFFMYNEPLRIQNAAGLSFSLLVFFCTSIGCIAVLVLRRLTLKAELGGPRIWAWVTFVYFMLLWIIFVV
P.persica       YNFFVYKEPLRIENAAGLSFSLLVFFSTSVGCIAVLVIRRRTLGAELGGPRLWAWITFVFFMLLWLIFVV
C.papaya        YNFFAYREPLGIQNAEGLSFSLLVFFSTSVGCIAVLVFRRLTLGAELGGPRFWAWVTCVYFMLLWIIFVV
S.tuberosum     YNYIAYNEPLRIDNAEGLSFSLLVFFSTSVACIGVLVFRRLTIGAELGGPRVWAWVTCIFFMLLWLIFVV
S.lycopersicum  YNYIAYNEPLRIENAEGLSFSLLVFFSTSVACIGVLVFRRLTIGAELGGPRVWAWVTCIFFMLLWLIFVV
M.guttatus_1    YNYFAYNEPLRIENAGGLSFSLLVFFATSIGCIGGLVFRRLTLGAELGGPKIWAWITCAYFMFLWLIFVV
C.sativus       YNFIAYKEPLKIKDAGGLSFSLLVFFSTSVACIVVLVFRRVTLGAELGGPKVWAWITCIFFMVLWVIFVV
G.max           YNFIAYREPLRIQNAGGLSFSLIVFFSTSVGCISVLVLRRIIFGAELGGPRLWAWITCAFFMLLWIIFVV
M.truncatula    YNFIAYRQPLRIQNAGGLSFSLIVFFATSVGCISVLVARRIVFGAELGGPRLWAWITCAFFMLLWIIFVV
A.coerulea      YNFIAYREPLRIENADGLSFSLLIFFLTSIGCISVLVLRRLTIGAELGGPRIWAWITSVYFMSLWIVFVV
V.vinifera      YNFIAYREPLGVQNAEGLSFSLLVFFCTSICCIGVLVLRRLTLGAELGGPKIWARLTCVYFMSLWIIFVV
E.grandis       YNFIMYREPLRIQNAAGLSFSLIVFFSTSVGCISVLVLRRLTLGAELGGPKPWAWLTSVYLMLLWVIFLV
M.guttatus_2    YNYIAYKKPLRIENAAGLSFSLLIFFATSVGCIGVLVFRRLTLGAELGGPRHWAWLTCFYLMLLWLVFVV
O.sativa_J1     YNYFVYQKPLYIDNAAGLSFSLLVFFATSFGCITVLVLRRVILGAELGGPRMWAWATSVYFMILWVVFVV
O.sativa_I1     YNYFVYQKPLYIDNAAGLSFSLLVFFATSFGCITVLVLRRVILGAELGGPRMWAWATSVYFMILWVVFVV
O.sativa_J2     YNYFVYKKPLYIDNAAGLSFSLLVFFATSFGCITVLVLRRVILGAELGGPRMWAWATSVYFMILWVVFVV
T.aestivum      YNFFAYQEPLYIDNAAGLSFSLLVFFATSFGCITVLVLRRIVFGAELGGPRLWAWATSAYFMVLWVVFVL
B.distachyon    YNYFVYQEPLYIDNAAGLSFSLLVFFATSFGCITVLVLRRIVLGAELGGPRLWAWVTSTYFMVLWVVFVV
Z.mays          YNFFVYREPLYIDNAAGLSFSLLVFFATSFGCITVLVLRRIILGAELGGPRLWAWVTSVYFMILWVVFVV
S.bicolor       YNFFVYQEPLYIDNAAGLSFSLLVFFATSFGCITVLVLRRIILGAELGGPRLWAWVTSVYFMILWVVFVV
S.italica       YNFFVYREPLYIDNAAGLSFSLLVFFATSFGCITVLVLRRIIIGAELGGPRLWAWITSVYFMILWIVFVV
consensus>50    YN%faY.ePL.!e#A.GLSFSLl!FFaTSvgCI.vLVlRRltlgAELGGPrlWAwvTsv%fMlLWv!Fvv

                530
A.thaliana      LSSLKVSGVI
A.halleri       LSSLKVSGVI
A.lyrata        LSSLKVSGII
C.clementina    LSSLKVSGII
R.communis      LSSLRVSGII
M.esculenta     LSSLRVSGFI
P.trichocarpa   LSSLRVSGII
P.persica       LSSLKVSGII
C.papaya        LSSLRVSGII
S.tuberosum     LSSLRVSGII
S.lycopersicum  LSSLRVSGII
M.guttatus_1    LSSLRVSGFI
C.sativus       LSSLKVSDII
G.max           LSSLKVSGFI
M.truncatula    LSSLKVSGFI
A.coerulea      LSSLKVSDII
V.vinifera      LSSLKIYGII
E.grandis       LSSLKVSGII
M.guttatus_2    LSSLQVSNII
O.sativa_J1     LSSLKISGVI
O.sativa_I1     LSSLKISGVI
O.sativa_J2     LSSLRISGVI
T.aestivum      LSSLRVSGVI
B.distachyon    LSSLRVSGVI
Z.mays          FSSLRVSGVI
S.bicolor       FSSLRVSGVI
S.italica       FSSLKVYGII
consensus>50    lSSLk!sgiI
```
