# Supplementary material for: Phylogeny and a structural model of plant MHX transporters
Source: BMC Plant Biol. 2013 May 2;13:75. doi: 10.1186/1471-2229-13-75 (PMC3679957; doi:10.1186/1471-2229-13-75)
Supplement: Additional file 9 — Alignment of MHX and NCX proteins. [file 1471-2229-13-75-S9.pdf]

Additional file 9. Alignment of MHX and NCX proteins

The file presents the alignment of all currently identified MHX proteins and the NCX proteins that were utilized as markers or identified in this work (excluding CrNCX). See the legends of Additional file 5 for details of sequence alignment and color annotation.

```
A.thaliana              ......................................................................
A.halleri               ......................................................................
A.lyrata                ......................................................................
C.sativus               ......................................................................
C.clementina            ......................................................................
R.communis              ......................................................................
M.esculenta             ......................................................................
P.trichocarpa           ......................................................................
P.persica               ......................................................................
C.papaya                ......................................................................
A.coerulea              ......................................................................
V.vinifera              ......................................................................
S.tuberosum             ......................................................................
S.lycopersicum          ......................................................................
M.guttatus_1            ......................................................................
M.guttatus_2            ......................................................................
G.max                   ......................................................................
M.truncatula            ......................................................................
E.grandis               ..................................................................MQAC
S.italica               ......................................................................
Z.mays                  ......................................................................
S.bicolor               ......................................................................
T.aestivum              ......................................................................
B.distachyon            ......................................................................
O.sativa_J1             ......................................................................
O.sativa_I1             ......................................................................
O.sativa_J2             ......................................................................
S.moellendorffii_1      ......................................................................
P.patens_1              ......................................................................
P.patens_2              ......................................................................
C.reinhardtii           .......................................................MAASATGLLPIADVL
C.intestinalis          ......................................................................
B.floridae              ......................................................................
T.adhaerens_3           ......................................................................
N.vectensis_2           ......................................................................
H.sapiens_NCX1.1        ...................................................MYNMRRLSLSPTFSMGFHL
P.troglodytes_NCX1.1    ...................................................MYNMRRLSLSPTFSMGFHL
C.familiaris_NCX1.1     ......................................................MLQLRLLPTFSMGCHL
F.catus_NCX1.1          ......................................................MLRLRLSPTFSVGFHL
C.porcellus_NCX1.1      ......................................................MLRLSLSPTYSLGFHL
B.taurus_NCX1.1         ......................................................MLQFSLSPTLSMGFHV
R.norvegicus_NCX1.1     ......................................................MLRLSLPPNVSMGFRL
M.musculus_NCX1.1       ......................................................MLRLSLPPNVSMGFRL
M.mulatta_NCX1.3        ......................................................MRRLSLSPTFSMGFHL
O.cuniculus_NCX1.2      ......................................................MPRFSLSPPFSMGFHL
S.tropicalis_NCX1       ......................................................MMKLGTSLAFFARFHF
O.mossambicus_NCX1.1    ...............................................MSPVRTVPMFFTYKLIFFATVIS
O.mykiss_NCX1           ..............................................MRRTGTSSFLFCALQLTVLLAVFS
D.rerio_NCX1a           ..............................................MGQSGTSSYFSLALNLSIFLLVFS
H.sapiens_NCX3.3        ....................................................MAWLRLQPLTSAFLHFGL
R.norvegicus_NCX3.3     ....................................................MAWLRLQPLTSAFLHFGL
M.musculus_NCX3.1       ....................................................MAWLRLQPLTSAFLHFGL
G.gallus_NCX3           ....................................................MAWLSLQPLTSAFLHFGL
D.rerio_NCX4a           ....................................................MFHLRLSRFSFSSITPCL
T.nigroviridis_NCX4a    ................................................................MPHTLI
H.sapiens_NCX2.1        .........................................................MAPLALVGVTLLL
R.norvegicus_NCX2.1     .........................................................MAPLALVGVALLL
M.musculus_NCX2.1       .........................................................MAPLALMGVVLLL
S.kowalevskii_3         ...................................MMKLLCSILSRTLSELTTVSRGVGASALLLVVAFL
T.adhaerens_2           .....................................MQLFSALLWVIAVVVLNFKVDPTLGSSIISERD
L.opalescens_NCX        MNPFKISFSWGVPLFLLGLFFDFAHASEDSNDTCTTEAET..............................
A.mellifera_NCX         ......................................................................
D.melanogaster_NCX      ......MQLLLKSIFTCALFVIFVYATAQSLLKVQETEARQAYLNVTSSSSSNLSQDDGHFLSRRLRQVS
H.magnipapillata        ................................................................MQFVLT
C.elegans_NCX1          .......................................................MTKLKIYLFLVVSLT
C.briggsae_NCX1         ............................................MRVSLKTITNAMTKLKIILFLVVSFG
C.elegans_NCX2          ......................................................................
C.briggsae_NCX2         ......................................................................
T.spiralis              ......................................................................
M.brevicollis_1         ......................................................MRPQTWPAWAVLAMLV
Salpingoeca             ...............................................................MVRGLSL
S.kowalevskii_1         ......................................................................
S.kowalevskii_2         ......................................................................
T.adhaerens_1           ......................................................................
D.pulex_1               ......................................................................
P.humanus_corporis      ......................................................................
O.dioica                ......................................................................
A.pisum                 ......................................................................
P.marinus               ..............................................................MIGLSLWG
A.anophagefferens_1     ......................................................................
E.huxleyi               ......................................................................
consensus>50            ......................................................................
```

```
                            1         10        20        30        40        50
A.thaliana             ..................MASILNQTQELQESSKVLGHLRCENFFLFPGENTLSDGLRGVLYFLGLAYC
A.halleri              ..................MASILNQTQELQEASKVLGHVRCENFFIFPGENTLSDGLRGVLYFLGLAYC
A.lyrata               ..................MASILNQTQELQEASKVLGHVRCENFFIFPGENTLSDGLRGVLYFLGLAYC
C.sativus              .................MASFAIENVESGQAISSISGTGKCESYFIFSIETSLGDALRIFLYFMGLAYC
C.clementina           .................MASIHTQAIESISRAFNILHYEKCESYLLFRGETSLGDGFRAFLYFLGLAYC
R.communis             ................MALETNYQSGWSRFGLLNILASEKCESYLVFRGETGLSVGFRTFLYFLGLAYC
M.esculenta            ................MALETQNQSAGSKFGLLNILGRERCESYLLFHGETALDNGFRTFLYFLALAYC
P.trichocarpa          .................MASAYNQSDGGRFGDTNIVGHEKCESYFLFRGETTLGEGFRTFLYFLGLAYC
P.persica              ............................MPGNSSTMGQENCESYFIFQGETGLGVGFRTFLYFLGLAYC
C.papaya               .................MTWAHWRNAESIIQNFNVLEQEKCESYLIFRSETALSNGFRAFLYFLGIAYC
A.coerulea             ....................................KCESYFLFQGEMALGNGLRAFLYLLGLAYC
V.vinifera             .................MAWVHEQLPGNILEAYNISVREKCESYLLFSGETSLGDGFRTFLYFLGLAYC
S.tuberosum            .................MTSLGNYTTDSTNGHSNILRHEKCDAYLLIHLETALGEGFRTFLYFLGLAYC
S.lycopersicum         .................MTSLGNYTTHSTNGHSNILRHEKCDAYLLFHLETALGGGFRTFLYFLGLAYC
M.guttatus_1           .................MDPLNNSTAEIIDGGSNILGVEKCQLYLLFHDETVLSDGFRGFLYFMALAYC
M.guttatus_2           .......................MASLNSSSTWDSNLGHERCLFYFIFHFETKLSHGLRGFLYFLALAYC
G.max                  ............................MVPFSNILGHEKCESYLIFGGETTLGDNFRAFFYFLCLAYC
M.truncatula           ........................MYKMIGYLSSFLAHDEKCESYLIFSGETNLGNSVRILLYFLGLAYC
E.grandis              FASVRLSLSSSLDSLPIFSVSFRRAGTPGDISRSHMMGHGVCESYLLFPGETALGDAFRTFLYFLGLAYC
S.italica              ...............................MSGPSSACEGTYLLFHGETLLSGGVRASLYTVALAYC
Z.mays                 ......................MAGAATPPPSSSSSASCGTDAAYLLFRGETLLPDGVHASLYAVALAYC
S.bicolor              ..................MSNIAMVAAPPSSSCDSDT......YLLFHGETLLSTGARASLYTVALAYC
T.aestivum             ..............................MGSTTRSCDAYLLFNGETLLPNGVRAFLCTVALAYC
B.distachyon           ............................MANISMGSNAPSCNAYLLFYAEKMLPNGVRAFAYTVALGYC
O.sativa_J1            ............................MANINMADTVPSCDTYLLFNGETLLPIGVRAFIYTAVLAYC
O.sativa_I1            ............................MANINMADTAPSCDTYLLFNGETLLPIGVRAFIYTAVLAYC
O.sativa_J2            ............................MANINMADTAPSCDTYLLFNGETLLPNGVRAFIYTVVLAYC
S.moellendorffii_1     ..................................CPSLLLTQEEQRWRFGTRAFAYCLGLAYC
P.patens_1             ........MAGFTNASISAHPSPGVWTNHFANAAVNATAELCSNHIIFKAEPSWWLSARAILYGLCLLYC
P.patens_2             .........AGLSSALVSAFSSPEKNTIHVVHASATSTAELCPNHIMFEAEPTWLHSSRAVLYAFCIIYC
C.reinhardtii          QQIEALVQVLPLCTDTQEAPCREDPTSARGVAIYTQNSDVVCTSDILLPGFNMLPRWLLGAVYLLFLLYL
C.intestinalis         .........MATTDVISYYSNGFVVEHVPIDETGNFTDAYCQSWLLIPAENLWPAWFRGFIYIIAIAYL
B.floridae             .........MDLIAWQTNYSAGYVVETLPNGTEP......CESWLLLPAENLWPSALRGILYFLAMAYI
T.adhaerens_3          .......MAAIPNGSTFYNFSNGFIVEY..........DGSCSSWLLIIGENFWHPAVRCILYIAAMIYL
N.vectensis_2          ............MAYRTNFSRGYVVEYSPPCFE.........SWLLLPAENLWNDGIRGFLYILGMLYL
H.sapiens_NCX1.1       LVTVSLLFSHVDHVIAETEMEGEGNETGECTGSYYCKKGVILPIWEPQ.DPSFGDKIARATVYFVAMVYM
P.troglodytes_NCX1.1   LVIVTLLFSHVDHVIAETEMEGEGNETGECTGSYYCKKGVILPIWEPQ.DPSFGDKIARATVYFVAMVYM
C.familiaris_NCX1.1    LAVVALLFSHVDLISAETEMEGEGNETGECTGSYYCKKGVILPIWEPQ.DPSFGDKIARATVYFVAMVYM
F.catus_NCX1.1         LAFVPLLFSHVDLISADTEMEGEGNETGECTGSYYCKKGVILPIWEPQ.DPSFGDKIARATVYFVAMVYM
C.porcellus_NCX1.1     LAMMTLLISHVDHITAETEMVEEGNETGECTGSYYCKKGVILPIWEPQ.DPSFGDKIARATVYFVAMVYM
B.taurus_NCX1.1        IAMVALLFSHVDHISAETEMEGEGNETGECTGSYYCKKGVILPIWEPQ.DPSFGDKIARATVYFVAMVYM
R.norvegicus_NCX1.1    VTLVALLFTHVDHITADTEAETGGNETTECTGSYYCKKGVILPIWEPQ.DPSFGDKIARATVYFVAMVYM
M.musculus_NCX1.1      VALVALLFSHVDHITADTEAETGGNETTECTGSYYCKKGVILPIWEPQ.DPSFGDKIARATVYFVAMVYM
M.mulatta_NCX1.3       LVIVALLFSHVDYVIAETEMEGEGNETGECTGSYYCKKGVILPIWEPQ.DPSFGDKIARATVYFVAMVYM
O.cuniculus_NCX1.2     LAIVALFFFRVDHVSAETEMEGEGNETGECTGSYYCKKGVILPIWEPQ.DPSFGDKIARATVYFVAMVYM
S.tropicalis_NCX1      LVLLLLLCNVGSIRSETTTVADFENHTDSCTGSYYCKEGVILPIWEPQ.NPSFGDKIARATVYFVAMVYM
O.mossambicus_NCX1.1   TEFPYSAAAGSTLTL...TSNQTATNHSKCGGSTDCIEGVILPLWKPE.NPAFTDRLARATIYFVGLVYM
O.mykiss_NCX1          SEIKFVTAGNSNPSLGTNSSIGNQTNKKCDSVDTECKVGVILPIWLPE.NTSFGDKLARATVYFVALFYM
D.rerio_NCX1a          YELTPVIAGSSKSSLDVDTSNANSSQETCGGSY.ECKEGVILPIWTPV.NPSFGDKLARATVYFVGLFYM
H.sapiens_NCX3.3       VTFVLFLNGLRAEAGGSGDVPSTGQNNESCSGSSDCKEGVILPIWYPE.NPSLGDKIARVIVYFVALIYM
R.norvegicus_NCX3.3    VTFVLFLNGLRAEAGDLRDVPSAGQNNESCSGSSDCKEGVILPIWYPE.NPSLGDKIARVIVYFVALIYM
M.musculus_NCX3.1      VTFVLFLNCLRAEAGDSGDVPSAGQNNESCSGSSDCKEGVILPIWYPE.NPSLGDKIARVIVYFVALIYM
G.gallus_NCX3          VTFVLFLHGLQVDAGLTGDSTSSVQNS.SCSGSFDCKEGVILPIWYPE.NPSLGDKIARVIVYFVALIYM
D.rerio_NCX4a          SSVLLLIFLSGLAHLSQASGDASHSGPGNCSGEDSCSEGVVLPIWNPQ.NPSVGDKVARAIVYFVALIYM
T.nigroviridis_NCX4a   SIVILLFPEVTRFSHGSVSHEDAGRTTGNCSSEDNCPEGVVLPLWNPQ.NPAVGDKVARAIVYFAALIYM
H.sapiens_NCX2.1       AAPPCSGAATPTPSLPPPPANDSDTSTGGCQGSYRCQPGVLLPVWEPD.DPSLGDKAARAVVYFVAMVYM
R.norvegicus_NCX2.1    GAPHCLGEATPTPSLPPPPANDSDASPGGCQGSYRCQPGVLLPVWEPD.DPSLGDKAARAVVYFVAMVYM
M.musculus_NCX2.1      GVPHCLGEATPTPSLPPPTANDSDASPEGCQGSYRCQPGVLLPVWEPE.DPSLGDKVARAVVYFVAMVYM
S.kowalevskii_3        NFHPTLTQETFRNSTYPPVWDGGEEEVCSRNATACKPGVLLPKWTPD.PVNVGDVAARATVYFAGLAYM
T.adhaerens_2          VSTINSTSTPTLSDAEKRLIACNTTTRCKNGLFLPHWPYPKDSKGNIL.PLSTGERVGRTIVYFFSLFFF
L.opalescens_NCX       .................................CRNGLIVPRWNPVGNLSVGDKLARATVYFVLMFYL
A.mellifera_NCX        ...............................MPGNYSTRCEPGLVVPVWRPQENLNLSDIIFRGFVYFSLLLYL
D.melanogaster_NCX     HGEEGDEGAPSQMDDELEQMTKVHGEAPDAEDVRECSEGLVLPLWMPQRNISVGDRLVRGFVYFVLLIYL
H.magnipapillata       FVFIWFVNAVFANETIIFTNITVKNTTCRTLENLKCKPGVVLPVWKSADSESKAVIAGKAFVYLVAMFYF
C.elegans_NCX1         TLGQYAAEPQNGEIIHVSSQRIPGPEP.ACAPAKPCSPGVIVPVWQPSENLSECKIWFRAIVYLIALAYL
C.briggsae_NCX1        QFQ..AQQQGNTEVVRVFSQRLPSPEDTACPPAKPCAPGVIVPVWQPSENLSECKIWFRAIVYLLVLAYL
C.elegans_NCX2         .MTRLGCWLAVAFLVALAGLADAGSNCSAADATRNCIDGLVIPIWRPFLDLTTGDRVLRGVLYFFVIAYM
C.briggsae_NCX2        .MTRLGCWLAVAALVALAGLADAGSNCSAADATRNCIDGLVVPIWRPFLDLTTGDRVLRGVLYFFVIAYM
T.spiralis             ..................MLQYNNGTTALIFVENAYPCSDRGLLLPLIDESRWPTATRAFLYLIGLLYS
M.brevicollis_1        AACVVPGLAQAPGPEPRPSDPPCSDTVLSQGTPICKGGSGTLLPAFNAYGEADWPEGLKIVLYFVGLLWT
Salpingoeca            ALVATTVIVSLSLVAGVHGEPCGDRIVNNTEKPICKGGSGTFLPAFNAYGEDNWSDGLKIFLYLAGLLWT
S.kowalevskii_1        ..........................MSNSTTDPGAYYCYDKGLILPIVNESTWSIGARTTIYLIGLLWS
S.kowalevskii_2        ..........................MSNSTTDPDAYLCYDKGLILPIVNESTWSTGARITVYLIGLIYC
T.adhaerens_1          ...................................MSYCNKTGLLLPILDESNWNQGFRGFLYLAGLLYS
D.pulex_1              ............................MTRNNGTLDDYTCSNALLLPLISEFTWSIEVRAFLYLFGLLYC
P.humanus_corporis     ......................MNVTGDLNELQNGEEDKCSESLFFPFLNDIHLNPIIRGILYFFALAYS
O.dioica               ........................MDGNVSVVDPGAYPQCAMGMLLPLVDESEMSMSLRIIMYLVGLFWF
A.pisum                ........................MGHGNTSSYPWGPNDVCPGGLILPILDERLWPIKVRVAVYLFTLLYF
P.marinus              VDSPRSLATPVIEQPIPICEHGGSGLVLEDRLMGYVEGSHPQILPLFGDSEQDWPNGLRVVLYFLGLCWC
A.anophagefferens_1    ...........................................................RAVLYLLGLLWF
E.huxleyi              ..MLNSTAVITNETAPLLNDTCEDGLLLAGPLRTHRN.....................ALIYMAALLWT
consensus>50           ......................................................e.......ra..yf..l.y.
```

```
                            60        70        80                            90        100
A.thaliana            FIGLSAITARFFK...SMENVVKHSRKVVT.IDPITKAEV............ITYKKVWNFTIADISLLA
A.halleri             FIGLSAITARFFK...SMENVVKHSRKVVA.IDPITKAEI............ITYKKVWNFTIADISLLA
A.lyrata              FIGLSAITARFFK...SMENVVKHSRKVVA.VDPITKAEV............ITYKKVWNFTIADISLLA
C.sativus             FVGLSAITARFFR...SMENVVKHSRKVVE.IDPHTNTEI............IRYEKVWNFTIADISLLA
C.clementina          FIGLSAITARFFR...SMENVVKHSRKVVE.IDPVTKAEV............VRYEKVWNYAIADIALLA
R.communis            FIGLSAITGRFFR...SMENVVKHSRKVVE.IDPYSNTEV............IRYEKVWNYTIADISLLA
M.esculenta           FFGLSAITARFFR...SMENVVKHSRKIVE.VDPDTNTEV............IRYEKVWNYAIADISLLA
P.trichocarpa         FIGLSAITARFFR...SMENVVKQSRKVVE.IDPYTNAEV............IRYEKVWNYAIADISLLA
P.persica             FVGLSAITARFFQ...SMESVVSHTRKVVD.INPYTGAEI............IRYEKVWNFTIADISLLA
C.papaya              FLGLSAITGRFFR...SMEHVVKHTREVVE.IDPITNQEI............TRHEKVWNYTIADISLLA
A.coerulea            FFGLSAITARFFR...SMENVVKQTRKVVE.IDHNTNTEV............IRHEKVWNYAIADITLLA
V.vinifera            FIGLSAITARFFQ...SMENVVKHKRKVVK.IDPRSNTE.............IRHEKVWNYTIADITLLA
S.tuberosum           FIGLSAITARFFR...SMESVVKHSRTVET.IDPLTNTKI............VKNEKVWNYTIADITLLA
S.lycopersicum        FIGLSAITARFFR...SMESVVKHSRTVET.IDPLTNTKA............VKNEKVWNYTIADITLLA
M.guttatus_1          FIGLSAITSRFFQ...SMENVVKHTRTVEE.IDPCTNTKT............VRHEKVWNYTIADITLLA
M.guttatus_2          FVGLSAITDRFFR...SMENVVKHSRAVEE.IDPLTNTKV............IKYEKVWNYTIADITLLA
G.max                 FIGLSAITARFFQ...SMENVVKHTRKVVE.VDPVTKTET............IRHEKVWNYTIADISLLT
M.truncatula          FIGLSAITSRFFQ...SMENVVKHSREVVV.IDPVTKAET............IRHEKVWNYTIADISLLA
E.grandis             FIGLSAITARFFS...SMENVVKQTREVVE.IDPQTNSKV............VRQEKVWNYTIADITLLA
S.italica             FIGLSAITARFFK...SMEQIMKHSREVVS.VDPHTNAPV............VKQEKVWNYTIADIALLA
Z.mays                FVGLSAVTARFFK...SMEQIMRHSREVVVGVDPRTGAPV............VRRDKVWNYTVADIALLA
S.bicolor             FIGLSAITARFFK...SMEQIMKHSREVVVI.DPHTKEPV............VKHEKVWNYTVADIALLA
T.aestivum            FIGLSAITARFFK...SMESITNHSREVVT.VDTETNTPI............VKHEKVWNYTIADIALLA
B.distachyon          FIGLSAITARFFK...SMESITNHSREVVT.IDPHTNTPI............VKHEKVWNYTIADIALLA
O.sativa_J1           FIGLSAITGRFFK...SMESIMRHSREVVT.VDPHTNATI............VKHEKVWNYTIADVALLA
O.sativa_I1           FIGLSAITGRFFK...SMESIMRHSREVVT.VDPHTNATI............VKHEKVWNYTIADVALLA
O.sativa_J2           FIGLSAISGRFFK...SMESIMRHSREVVT.IDPHTNATI............VKHEKVWNYTIADVALLA
S.moellendorffii_1    FLGLAVITRVYMRALDS....IVRHSRKSVHRDPITGLRI............FSERRIWNPTVADITLLA
P.patens_1            FVGLATITNLFMQAGGSAMGTIANRTRKIVRHNDESGSEE............VVHVRVWNPVIADITLLA
P.patens_2            FVGLATITNLYMEARSSAMEKIVHQTRKVVRHNYEIGSDE............IVHERIWNLVIADITLLA
C.reinhardtii         FAGVAIASDMFMDGIMNICAITKIYKRKN.EKGETIYV...............KEPVWNWVANITLMA
C.intestinalis        FIGVAIGSDVFMTSIEVITSKKRTIIIWDEELGENTKK...............EVLVWNETVANLTLLA
B.floridae            FIGITIGSDVFMCSIEVITSKKRKVVRWDEERQETVER...............EVLLWNETIANLTLLA
T.adhaerens_3         FVGVAIMSDVFMGAIEVITSKKKTVITYDRETGEQATK...............EVLIWNETVANLSLMA
N.vectensis_2         FLGIAIVADIFMSCIEVITSKKRKVTRYDHEKGESVEI...............EVFVWNETVANLTLMA
H.sapiens_NCX1.1      FLGVSIIADRFMSSIEVITSQEKEITIK.KPNGETTKTT...............VRIWNETVSNLTLMA
P.troglodytes_NCX1.1  FLGVSIIADRFMSSIEVITSQEKEITIK.KPNGETTKTT...............VRIWNETVSNLTLMA
C.familiaris_NCX1.1   FLGVSIIADRFMSSIEVITSQEKEITIK.KPNGETTKTT...............VRIWNETVSNLTLMA
F.catus_NCX1.1        FLGVSIIADRFMSSIEVITSQEKEITIK.KPNGETTKTT...............VRIWNETVSNLTLMA
C.porcellus_NCX1.1    FLGVSIIADRFMSSIEVITSQEKEITIK.KPNGETTKTT...............VRIWNETVSNLTLMA
B.taurus_NCX1.1       FLGVSIIADRFMSSIEVITSQEKEITIK.KPNGETTKTT...............VRIWNETVSNLTLMA
R.norvegicus_NCX1.1   FLGVSIIADRFMSSIEVITSQEKEITIK.KPNGETTKTT...............VRIWNETVSNLTLMA
M.musculus_NCX1.1     FLGVSIIADRFMSSIEVITSQEKEITIK.KPNGETTKTT...............VRIWNETVSNLTLMA
M.mulatta_NCX1.3      FLGVSIIADRFMSSIEVITSQEKEITIK.KPNGETTKTT...............VRIWNETVSNLTLMA
O.cuniculus_NCX1.2    FLGVSIIADRFMSSIEVITSQEKEITIK.KPNGETTKTT...............VRIWNETVSNLTLMA
S.tropicalis_NCX1     FLGVSIIADRFMSSIEVITSQEKEITIK.KPNGETTKTT...............VRIWNETVSNLTLMA
O.mossambicus_NCX1.1  FLGVSIIADRFMASIEVITSQERRITIK.KPNGEKITTT...............VRIWNETVSNLTLMA
O.mykiss_NCX1         FLGVSIIADRFMASIEVITSQEREITIK.KPNGEKVTTT...............VRIWNETVSNLTLMA
D.rerio_NCX1a         FLGVSIIADRFMASIEVITSQEKEITIK.KPNGETTTTT...............VRIWNETVSNLTLMA
H.sapiens_NCX3.3      FLGVSIIADRFMASIEVITSQEREVTIK.KPNGETSTTT...............IRVWNETVSNLTLMA
R.norvegicus_NCX3.3   FLGVSIIADRFMASIEVITSQEREVTIK.KPNGETSTTT...............IRVWNETVSNLTLMA
M.musculus_NCX3.1     FLGVSIIADRFMASIEVITSQEREVTIK.KPNGETSTTT...............IRVWNETVSNLTLMA
G.gallus_NCX3         FLGVSIIADRFMASIEVITSQEKEITIK.KPNGETTTTT...............IRIWNETVSNLTLMA
D.rerio_NCX4a         FLGMSIIADRFMSSIEVITSQEKEITIK.KPNGETTTAT...............VRIWNETVSNLTLMA
T.nigroviridis_NCX4a  FLGMSIIADRFMSSIEVITSQEKEITIK.KPNGETTTTT...............VRIWNETVSNLTLMA
H.sapiens_NCX2.1      FLGVSIIADRFMAAIEVITSKEKEITIT.KANGETSVGT...............VRIWNETVSNLTLMA
R.norvegicus_NCX2.1   FLGLSIIADRFMASIEVITSKEKEITIT.KANGETSVGT...............VRIWNETVSNLTLMA
M.musculus_NCX2.1     FLGVSIIADRFMASIEVITSKEKEITIT.KANGETSVGT...............VRIWNETVSNLTLMA
S.kowalevskii_3       FLGVSIVADRFMAAIEVITSKEKEVTVK.RPNGEKVVVS...............VRIWNETVSNLTLMA
T.adhaerens_2         FIGVSIISDRFMSAIEIITSKERFIKVK.MSNGEYKRIS...............VRVWNETVSNLTLMA
L.opalescens_NCX      FLGVSIIADRFMAAIEVITSKEKDVVVK.KPDGTTTVVN...............VRIWNETVSNLTLMA
A.mellifera_NCX       FIGVSIISDVFMAAIEVITSKEKELVVR.RQGKEPQIVV...............VRVWNETVANLTLMA
D.melanogaster_NCX    FVGVSIIADRFMAAIEAITSIERAVVVK.GPNNTKQVMH...............VRIWNETVANLTLMA
H.magnipapillata      FLGISIISDIFMSSIEIITSKEKEVLVRDRVTGKKHSVT...............VKIWNETVANLTLMA
C.elegans_NCX1        FFGVSIVADRFMASIEVITSQQKSVKMK.KITGEHFTIM...............VRVWNETVSNLTLMA
C.briggsae_NCX1       FFGVSIVADRFMASIEVITSQTKSVKMK.KITGEPFTVL...............VRVWNETVSNLTLMA
C.elegans_NCX2        FLGISIVADRFMSSIEVITSMERTIVVK.RPGLDPMAVQ...............VRIWNDTVSNLTLMA
C.briggsae_NCX2       FLGISIVADRFMSSIEVITSMERTIVVK.RPGLDPMEVQ...............VRIWNDTVSNLTLMA
T.spiralis            FVGVAILADSFMCAVERITSTKRTLKLQTAIDEETGSVLEQYQ...........EVLIWNPTVANLTLMA
M.brevicollis_1       FAGVGIITDVFMEAIEVITSRERHVMV.DGI.....................ETTVLIWNPTIANLSLMA
Salpingoeca           FSGIGIITDVFMEAIEVITSKEKEVKLKDGR.....................VVHVQVWNPTIANLSLMA
S.kowalevskii_1       FMGVSIIADIFMCSIEVITSKTKTVNIANPKAPGGTEE...............IKVRVWNSTVANLTLMA
S.kowalevskii_2       LYGVSIITGIFMSSIEVITSKTKTVNIANPKAPGGTQK...............LKVRVWNSTVANLTLVT
T.adhaerens_1         FLGVAIIADIFMCAIEVITSQTRRIKISDKDGNMKE.................VEVRIWNDTVANLTLMA
D.pulex_1             FTGVAIIADIFMGAIEKITSTTRKVFINHRSQEPEV.................IEVRIWNDTVANLTLMA
P.humanus_corporis    FVGVSVVTDIFMGAVVTITSQTKKVYLGKFKKKPEESESALNVTATSTHQLDFIEVRVWNDTVANLTLMA
O.dioica              FIGVAIVADIFMVSIEVITSRETHITILVDGQPEKQ.................KVRFWNATVANLTLMA
A.pisum               LLGISIVADVFMTAIDKITSHTKKVYLAKELKPNGSDVSLRPD......QPEVIEVRIWNPTIANLTLMA
P.marinus             FVGVAIISDFFMGAIEKITSKKKRARLTIQGETK...................LVTVRVWNDTVANLTLMA
A.anophagefferens_1   FLGVSIIADVFMAAIETITSKEKKVPGTD..........................VWNATVANLTLMA
E.huxleyi             FLGVAIAADAFMLGIETITSQEQAKTVVVDGRKR...................VYSLKIWNATVANLTLMA
consensus>50          f.Gvsii.dr%m..ievits..k.i.........t..................vrvWNet!a#ltLma
```

```
                            110       120                 130       140       150       160       170
A.thaliana                  FGTSFPQISLATIDAIRNMG...ERYAGGLGPGTLVGSAAFDLFPIHAVCVVVPKAGELKKISDLGVWLV
A.halleri                   FGTSFPQISLATIDAIRNIG...ERYAGGLGPGTLVGSAAFDLFPIHAVCVVVPKAGELKKISDLGVWLV
A.lyrata                    FGTSFPQISLATIDAIRNIG...ERYAGGLGPGTLVGSAAFDLFPIHAVCVVVPKAGELKKISDLGVWLV
C.sativus                   FGTSFPQISLATIDAIRNIG...NLYAGGLGPGTLVGSAAFDLFPIHAVCVVVPKAGELKKISDIGVWLV
C.clementina                FGTSFPQISLATIDAIRSIG...NLYAGGLGPGTLVGSAAFDLFPIHAVCVVVPKAGELKKISDIGVWLV
R.communis                  FGTSFPQISLATIDAIRNIG...NLYAGGLGPGTLVGSAAFDLFPIHAVCVVVPKAGELKKISDLGVWLV
M.esculenta                 FGTSFPQISLATIDAIRNIG...NLYAGGLGPGTLVGSAAFDLFPIHAVCVVAPKAGELKKIADLGVWIV
P.trichocarpa               FGTSFPQISLATIDAIQNIG...NLYAGGMGPGTLVGSAAFDLFPIHAVCVVVPKAGELKKIADIGVWLV
P.persica                   FGTSFPQISLATIDAIRNLG...NLYAGGLGPGTLVGSAAFDLFPIHAVCVVVPRAGELKKIADVGVWLV
C.papaya                    FGTSFPQISLATIDAIRNLG...NLNAGGLGPGTLVGSAAFDLFPIHAVCVVVPKGGEMKKIADIGVWLV
A.coerulea                  FGTSFPQISLATIDAIRNLG...QIYAGGLGPGTLVGSAAFDLFPIHAVCVVVPKAGELKKISDIGVWLV
V.vinifera                  FGTSFPQISLAIIDSIQNLG...SRYAGGLGPGTLVGSAAFDLFPIHAVCVVVPKAGELKKISDVGVWLV
S.tuberosum                 FGTSFPQISLATIDAIRNIG...KLYAGGLGPGTLVGSAAFDLFPIHAVCVVVPKAGELKKISDIGVWLV
S.lycopersicum              FGTSFPQISLATIDAIRNIG...KLYAGGLGPGTLVGSAAFDLFPIHAVCVVVPKAGELKKISDIGVWLV
M.guttatus_1                FGTSFPQISLATIDAIRNLG...SLYAGGLGPGTLVGSAAFDLFPIHAVCVVVPKAGELKKISDVGVWLV
M.guttatus_2                FGTSFPQISLATIDAIRNIG...SLTAGGLGPGTLVGSAAFDLFPIHAVCVVVPKAGELKRISDIGVWLV
G.max                       FGTSFPQISLATIDAIQNIG...KLYAGGLGPGTLVGSAAFDLFPIHAVCVVVPKAGELKKIADLGVWLV
M.truncatula                FGTSFPQISLATIDAIRNLG...DLYAGGLGPGTLVGSAAFDLFPIHAVCVVIPKAGELKKIADLGVWIV
E.grandis                   FGTSFPQISLATIDALRNLG...ELYAGGLGPGTLVGSAAFDLFPIHAVCVVVPKSGELKKISDLAVWAV
S.italica                   FGTSFPQISLATIDAIRNLG...QLTAGGLGPGTLVGSAAFDMFPIHAVCVIMPKAGSKKKITDLGVWLV
Z.mays                      FGTSFPQISLATIDAIRNLG...QLTAGGLGPGTLVGSAAFDLFPIHAVCVLMPRAGSKKKISDLGVWLV
S.bicolor                   FGTSFPQISLAIIDAIRNLG...QLTAGGLGPGTLVGSAAFDLFPIHAVCVVMPRAGSKKKISDLGVWLV
T.aestivum                  FGTSFPQISLATIDAIRNLG...QLTAGGLGPGTLVGSAAFDLFPIHAVCVVMPRAGSMKKISDLGVWLV
B.distachyon                FGTSFPQISLATIDAIRNLG...QLTAGGLGPGTLVGSAAFDLFPIHAVCVIMPRAGSMKKISDLGVWLV
O.sativa_J1                 FGTSFPQISLATIDAIRNLG...QLTAGGLGPGTLVGSAAFDLFPIHAVCVVMPRAGSKKKISDLGVWLV
O.sativa_I1                 FGTSFPQISLATIDAIRNLG...QLTAGGLGPGTLVGSAAFDLFPIHAVCVVMPRAGSKKKISDLGVWLV
O.sativa_J2                 FGTSFPQISLATIDAIRNLG...QLTAGGLGPGTLVGSAAFDLFPIHAVCVVMPRAGSKKKISDLGVWLV
S.moellendorffii_1          LGTCAPQVSLAVIDAFQHIG...Q.TSGKLGAGTLLGSTAFNLFLILAVCVVAPKRFQTKSIRNVGVYII
P.patens_1                  LGTSAPQISLSIIDAIQQIG...QKTNAGLGPETIVGSAAFNLYPILAVCVLVPKAGSVKRIQNVGVWIV
P.patens_2                  LGTSAPQISLAIIDAFQQLG...QKTEAGLGPGTIIGSAAFNLYIILAVCVLVPKAGSTKHIRSIGVWIV
C.reinhardtii               LGTSSPEIMLSLVEALLTLGKPA....GELGPSCIAGSAAYNFL.ISAVCTTALPDGQFKKISQLRVYVV
C.intestinalis              LGSSAPEILLATVETINEILSGNQAKGGGLGFYTIVGSAAFNLLVITGICIISVPSPSHKSIRELGVFIL
B.floridae                  LGSSAPEIMLNIIEAVKELGNPPED...GLGPFTIVGSAAFNLLVINAICVASVPSPEIKAVREFGVFVI
T.adhaerens_3               LGSSAPEILLSIVETVSEISDPNSVAD.GLGSFTIIGSASFNLLIITAVCIVSVPAPHYKRVKEFGVFII
N.vectensis_2               LGSSAPEILLAVVETGQELALGQTTATDGLGTFTIVGSASFNLLLITAVCVVSVPNGTVKRIREFGVFVV
H.sapiens_NCX1.1            LGSSAPEILLSVIEVC.....GHNFTAGDLGPSTIVGSAAFNMFIIIALCVYVVPDGETRKIKHLRVFFV
P.troglodytes_NCX1.1        LGSSAPEILLSVIEVC.....GHNFTAGDLGPSTIVGSAAFNMFIIIALCVYVVPDGETRKIKHLRVFFV
C.familiaris_NCX1.1         LGSSAPEILLSVIEVC.....GHNFTAGDLGPSTIVGSAAFNMFIIIALCVYVVPDGETRKIKHLRVFFV
F.catus_NCX1.1              LGSSAPEILLSVIEVC.....GHNFTAGDLGPSTIVGSAAFNMFIIIALCVYVVPDGETRKIKHLRVFFV
C.porcellus_NCX1.1          LGSSAPEILLSVIEVC.....GHNFTAGDLGPSTIVGSAAFNMFIIIALCVYVVPDGETRKIKHLRVFFV
B.taurus_NCX1.1             LGSSAPEILLSVIEVC.....GHNFTAGDLGPSTIVGSAAFNMFIIIALCVYVVPDGETRKIKHLRVFFV
R.norvegicus_NCX1.1         LGSSAPEILLSVIEVC.....GHNFTAGDLGPSTIVGSAAFNMFIIIALCVYVVPDGETRKIKHLRVFFV
M.musculus_NCX1.1           LGSSAPEILLSVIEVC.....GHNFTAGDLGPSTIVGSAAFNMFIIIALCVYVVPDGETRKIKHLRVFFV
M.mulatta_NCX1.3            LGSSAPEILLSVIEVC.....GHNFTAGDLGPSTIVGSAAFNMFIIIALCVYVVPDGETRKIKHLRVFFV
O.cuniculus_NCX1.2          LGSSAPEILLSVIEVC.....GHNFTAGDLGPSTIVGSAAFNMFIIIALCVYVVPDGETRKIKHLRVFFV
S.tropicalis_NCX1           LGSSAPEILLSVIEVC.....GHNFQAGDLGPSTIVGSAAFNMFIIIALCVYVVPDGEIRKIKHLRVFFV
O.mossambicus_NCX1.1        LGSSAPEILLSVVEVC.....GHNFNAGELGPNTIVGSAAFNMFVIIGLCVSVIPEGETRKVKHLRVFFI
O.mykiss_NCX1               LGSSAPEILLSVVEVC.....GHNFDAGDLGPNTIVGSAAFNMFVIIGFCVSVIPDGEHRKVKHLRVFFV
D.rerio_NCX1a               LGSSAPEILLSVVEVC.....GHNFDAGELGPNTIVGSAAFNMFVIIGLCVSVIPEGEHRKVKHLRVFFV
H.sapiens_NCX3.3            LGSSAPEILLSLIEVC.....GHGFIAGDLGPSTIVGSAAFNMFIIIGICVYVIPDGETRKIKHLRVFFI
R.norvegicus_NCX3.3         LGSSAPEILLSLIEVC.....GHGFIAGDLGPSTIVGSAAFNMFIIIGICVYVIPDGETRKIKHLRVFFV
M.musculus_NCX3.1           LGSSAPEILLSLIEVC.....GHGFIAGDLGPSTIVGSAAFNMFIIIGICVYVIPDGETRKIKHLRVFFV
G.gallus_NCX3               LGSSAPEILLSLIEVC.....GHGFVAGDLGPSTIVGSAAFNMFIIIAICVYVIPDGETRKIKHLRVFFV
D.rerio_NCX4a               LGSSAPEILLSVIEVC.....GHKFEAGHLGPSTIVGSAAFNMFIIIALCVYVVPDGEVRKIKHLRVFFV
T.nigroviridis_NCX4a        LGSSAPEILLSVIEVI.....GHNFDAGALGPSTIVGSAAFNMFIIIAICVYVVPENETRKIKHLRVFFV
H.sapiens_NCX2.1            LGSSAPEILLSVIEVC.....GHNFQAGELGPGTIVGSAAFNMFVVIAVCIYVIPAGESRKIKHLRVFFV
R.norvegicus_NCX2.1         LGSSAPEILLSVIEVC.....GHNFQAGELGPGTIVGSAAFNMFVVIAVCVYVIPAGESRKIKHLRVFFV
M.musculus_NCX2.1           LGSSAPEILLTVIEVC.....GHNFQAGELGPGTIVGSAAFNMFVVIAVCVYVIPAGESRKIKHLRVFFV
S.kowalevskii_3             LGSSAPEILLSLIEII.....GNGFHAGDLGPGTIVGSAAFNLFVIIAICVYAIPDGEVRRLKHLRVFFV
T.adhaerens_2               LGSSAPEILLSIIEIC.....GNNFKPGDLGPSTIVGSAAFNLLIITALCVLAIPDDEIRRIKHLSVFFI
L.opalescens_NCX            LGSSAPEILLSVIEVV.....GQKFEAGQLGPSTIVGSAAFNLFIITAICVTVIPNGEVRTIKHLGVFFI
A.mellifera_NCX             LGSSAPEILLSIIEIW.....AKNFEAGELGPGTIVGSAAYNLFVIISLCVFVIPNGETRKIKHLRVFFV
D.melanogaster_NCX          LGSSAPEILLSVIEIY.....AKDFESGDLGPGTIVGSAAYNLFMIIAVCMIWIPAGEVRRIRHLRVFFV
H.magnipapillata            LGSSAPEILLSVIEVI.....GQGFKAGELGPSTIVGSAAFNLFMITAVCVVVLPPGEVRRIKHIKVFAF
C.elegans_NCX1              LGSSAPEILLSVIEIC.....GNNFEAGELGPSTIVGSAAFNLFIIIAVCIMAIPNGETRRVQHNGVFWV
C.briggsae_NCX1             LGSSAPEILLSVIEIC.....GNNFEAGELGPSTIVGSAAFNLFIIIAVCIMAIPNGEIRRVQHNGVFWV
C.elegans_NCX2              LGSSAPEILLSIIEVI.....ARGFEAGDLGPNTIVGSAAFNLFMIIAICVVVIPKGEIRRQKHLDVFCV
C.briggsae_NCX2             LGSSAPEILLSIIEVI.....ARGFEAGDLGPNTIVGSAAFNLFMIIAICVVVIPKGEIRRQKHLDVFCV
T.spiralis                  LGSSAPEILLSIIEII.....GNGFKAGDLGPGTIVGSAAFNLFIITAVCILSVGCNSKKRIEDFQVFCV
M.brevicollis_1             LGSSAPEIILSIIEIV.....AGEFFAGALGPSTIVGSAAFNLFVIIAVCLLALPAMEVRRIESMGVFLV
Salpingoeca                 LGSSAPEIILSVIEVV.....AGNFFAGALGPSTIVGSAAFNLLVIFAVCSLAVPQGEVRRLEQYGVFIV
S.kowalevskii_1             LGSSAPEILLSVIEVV.....GNGFQAGELGPSTIVGSAAFNLLVITAVCISCLPTGEVRYIKLVKVFAL
S.kowalevskii_2             LGSRTTEILLCLIEIV.....GNGFHAGYIGPSTIIGSAAFNLLVITAVCIACPPIGEVRYIKQIKVSTL
T.adhaerens_1               LGSSAPEILLSIIEIV.....GNNFKAGDLGPGTIVGSAAFNLLVITATCIVAIPNPEGRRIKGIKVFAV
D.pulex_1                   LGSSAPEILLSIIEIV.....GNNFESGSLGPGTIVGSAAFNLMGISAVCVAAIPGVESRQIKGLKVFAI
P.humanus_corporis          LGTSAPEILLACMEVI.....KRNFESGELGPGTIVGSAAFNLLLITAICIISVPNGESRRIRLFNVFIV
O.dioica                    MGSSMPEIFLNIIEIV.....FNKFETGALGPGTIVGSAAFNLMVIIGVCTFGIPDGEKRRIDRFYVYGI
A.pisum                     LGTSSPEILLSIIETV.....GHHFEARKLGPNIIVGSAAFNLLIITSLCTLSLGLNEKRRIHDLKVFLV
P.marinus                   LGSSAPEILLSIIELF.....SQDMYSGHLGPSTIVGSAAFNLLVISAVCIMAIPDGQVRAINDIGVFVV
A.anophagefferens_1         LGSSAPEILLSVIEIL.....TNSFFTGDLGPSTIVGSAAFNMFCIIAICMVSLPGTETRKIEDMSVYTV
E.huxleyi                   LGSSAPEILLSLIEIV.....SSGFYSGELGPSTIVGSAAFNLFIIAAVCVVAVAAPKKVA..DTGVFSI
consensus>50                lGssap#!lLsvi#vi.......nf.ag.lGp.tivGSaa%#lfi!.avCv.vip.ge.rki.dl.Vf.v
```

```
                            180       190       200       210       220       230       240
A.thaliana            ELVWSFWAYIWLYIILEVWSPNVITLVEALLTVLQYGLLLVHAYAQDKRWPYLSLPMSRGDRPEEWVPEE
A.halleri             ELVWSFWAYIWLYIILEVWSPNVITLVEALLTVLQYGLLLVHAYAQDKRWPYLSLPMSRGDRPEEWVPEE
A.lyrata              ELVWSFWAYIWLYIILEVWSPNVITLVEALLTVLQYGLLLVHAYAQDKRWPYLSLPMSRGDRPEEWVPEE
C.sativus             ELVWSFWAYIWLYIILEVWTPKVITLWEALLTVLQYGLLLTHAYAQDKRWPYLSLPLARTERPEEWVPPE
C.clementina          ELFWSFWAYVWLYIILEVWTPNVITFWEALLTVLQYGLLLIHAYAQDKRWPYLSLPIARTERPEDWVPQE
R.communis            ELFWSFWAYVWLYIILEVWTPNVITLWEALLTVLQYGLLLTHAYAQDKRWPYLSLPIERTERPEEWVPEE
M.esculenta           ELFWSFWAYIWLYIILEVWTPNVITLWEALVTVLQYGLLLIHAYAQDKRWSYLSLPLGRTERPKEWVPEE
P.trichocarpa         ELFWSFWAYIWLYIILEVWTPNVITLWEALLTVLQYGLLLMHAYAQDKRWPYLSLPIPRTERPEEWVPGE
P.persica             ELFWSFWAYIWLYIILEVWTPNVITIWEALLTVLQYGLLLMHAYAQDKRWPYFSLPLPRSERPEDWVPAE
C.papaya              ELFWSFWAYIWLYIILEVWTPNVVTLWEALLTVLQYGLLLTHAYAQDKRWPYLSLPIGRTERPEDWVPEE
A.coerulea            ELFWSFWAYIWLYIILEVWTPNVVTLWEALITVLQFGLLLIHTYAQDKRWPYLSLPLARTERPEDWVPVE
V.vinifera            ELFWSFWAYVWLYIILEVWTPNVITIWEALLTVLQFGLLLIHAYAQDKQWPYLSLPLPRTERPEDWVPDE
S.tuberosum           ELFWSFWAYIWLYIILEVWTPNVVTLWESILTVLQFGLLLIHAYAQDKRWPYLSLPLERAERPEEWVPAE
S.lycopersicum        ELFWSFWAYIWLYIILEVWTPNVVTLWESILTVLQFGLLLIHAYAQDKRWPYLSLPLERAERPEEWVPAE
M.guttatus_1          ELFWSFWAYIWLYIILEVWTPNIITLLEALLTVLQFGLLLIHAYAQDKRWPYLSLPIGRTERPEEWVPVE
M.guttatus_2          ELFWSFWAYIWLYIILKIWTPNVITLWEALLTVAQFGLLLIHAYAQDKRWPYVSLPMTRSERPEDWVPAE
G.max                 ELFWSFWAYIWLYIILEVWTPNVVTLWEALLTVLQYGLLLTHAYAQDKRWPYISLPIARDERPEDWVPEE
M.truncatula          ELFWSFWAYIWLYIILEVWTPNVITLWEALLTVLQYGLLLIHAYAQDKRWPYISLPIARDERPEDWVPEE
E.grandis             ELFWSFWAYIWLYIILKVWTPDIVTLWEALLTVLQFGLLLVHAYAQDKRWPYLSIPLARGERPEEWVPEE
S.italica             ELFWSFWAYIWLYVILEVWTPKVITVWEALLTVLQYGLLLLHAYAQDKRWPYVSIPLVRGDRPEDWVPEE
Z.mays                ELFWSFWAYIWLYVILEVWTPRVITLWEASLTVLQYGLLLLHAYAQDKRCPFVSIPFARGERPEDWVPPE
S.bicolor             ELVWSFWAYIWLYVILEVWTPKVITLWEALLTVLQYGLLLLHAYAQDKRWPFVSIPFVRGERPEDWVPPE
T.aestivum            ELFWSFWAYIWLYIILEVWTPNVITLWEALLTVLQYGLLLVHAYAQDKRWPYVSIPLVRGERPEDWVPAE
B.distachyon          ELFWSFWAYIWLYIILEVWTPNVITLWEALLTVLQYGLLLVHAYAQDKRWPYVSIPLVRGERPEDWVPAE
O.sativa_J1           ELFWSFWAYIWLYIILEVWTPRVITLWEALLTVLQYGLLLLHAYAQDKRWPYVSIPLARGERPEDWVPAE
O.sativa_I1           ELFWSFWAYIWLYIILEVWTPRVITLWEALLTVLQYGLLLLHAYAQDKRWPYVSIPLARGERPEDWVPAE
O.sativa_J2           ELFWSFWAYIWLYIILEVWTPRVITLWEALLTVLQYGLLLLHAYAQDKRWPYVSIPLARGDRPEDWVPTE
S.moellendorffii_1    EVVWSFWAYVWLFLILEIWTPNEITLWEAVLTVAQFPLLVLHAYVQDRNWKYLSIPLRESDAIESSDMNN
P.patens_1            ELSWSIWAYVWLAIILQVSSPNVVEPWEAMCTVLQFPILMTHAYIQDKGWGNFCRPLWPCMHVSWSPLKD
P.patens_2            ELTWSMWAYIWLAIILQVSSPDVVEPWEAVCTVLQFPILMMHAYVQDVGLETLCCPS...MIVSWKPIRK
C.reinhardtii         TAAWSIWAYIWMLIVYVYWTPNEVTLAEAFLTLGFFVLMVLTAWIVDKQPWKKNNKIISRSDPEAPAALP
C.intestinalis        TSMWSLLAYVWLLCVMLWTSPNVVEPWEAWLTLIFFPLLVITAYAQDNGWWMYKLRGQNSVQDASKPRDE
B.floridae            TSLWSMFAYVWMLVVVLWVTPGVIDVWEAWVTLGFTPLLVLTAYAQDSGWWCKRQHSRIHNSEPSIQEDM
T.adhaerens_3         TAIWSVFAYVWMLIVTTVWTPNKIDIFEAWITLAFFPLLILTAWAQDNGWWWKRHSQILPNEDKSLTVRV
N.vectensis_2         TAVWSMFAYVWMLFVIQWNSPEVIELWEAFMTLAFFPLLVLTAWCTDHGWWCRNRGKILSVDPPQVIVII
H.sapiens_NCX1.1      TAAWSIFAYTWLYIILSVISPGVVEVWEGLLTFFFFPICVVFAWVADRRLLFYKYVYKRYRAGKQRGMII
P.troglodytes_NCX1.1  TAAWSIFAYTWLYIILSVISPGVVEVWEGLLTFFFFPICVVFAWVADRRLLFYKYVYKRYRAGKQRGMII
C.familiaris_NCX1.1   TAAWSIFAYTWLYIILSVISPGVVEVWEGLLTFFFFPICVVFAWVADRRLLFYKYVYKRYRAGKQRGMII
F.catus_NCX1.1        TAAWSIFAYTWLYIILSVISPGVVEVWEGLLTFFFFPICVVFAWVADRRLLFYKYVYKRYRAGKQRGMII
C.porcellus_NCX1.1    TAAWSIFAYTWLYIILSVISPGVVEVWEGLLTFFFFPICVVFAWVADRRLLFYKYVYKRYRAGKQRGMII
B.taurus_NCX1.1       TAAWSIFAYTWLYIILSVSSPGVVEVWEGLLTFFFFPICVVFAWVADRRLLFYKYVYKRYRAGKQRGMII
R.norvegicus_NCX1.1   TAAWSIFAYTWLYIILSVSSPGVVEVWEGLLTFFFFPICVVFAWVADRRLLFYKYVYKRYRAGKQRGMII
M.musculus_NCX1.1     TAAWSIFAYTWLYIILSVSSPGVVEVWEGLLTFFFFPICVVFAWVADRRLLFYKYVYKRYRAGKQRGMII
M.mulatta_NCX1.3      TAAWSIFAYTWLYIILSVISPGVVEVWEGLLTFFFFPICVVFAWVADRRLLFYKYVYKRYRAGKQRGMII
O.cuniculus_NCX1.2    TAAWSIFAYTWLYIILSVISPGIVEVWEGLLTFFFFPICVVFAWVADRRLLFYKYVYKRYRAGKQRGMII
S.tropicalis_NCX1     TAAWSIFAYTWLYMILSVFSPGVVEVWEGLLTFFFFPICVVFAWVADRRLLFYKYVYKRYRAGKQRGMII
O.mossambicus_NCX1.1  TAAWSVFAYTWLYLILAVFSPGVVEIWEGLLTLFFFPLCVGLAYIADRRLLFYKYMYKRYRAGKRKGVII
O.mykiss_NCX1         TATWSIFAYTWLYLILAVISPGIVQVWEGLVTLFFFPLCVGMAYVADRRLLVYKYMYKRYRAGKRRGVII
D.rerio_NCX1a         TATWSIFAYTWLYLILAVISPGIVEVWEGLLTLFFFPICVLFAWVADRRLLFYKYVYKRYRVGKKRGIII
H.sapiens_NCX3.3      TAAWSIFAYIWLYMILAVFSPGVVQVWEGLLTLFFFPVCVLLAWVADKRLLFYKYMHKKYRTDKHRGIII
R.norvegicus_NCX3.3   TAAWSVFAYIWLYMILAVFSPGVVQVWEGLLTLFFFPVCVLLAWVADKRLLFYKYMHKRYRTDKHRGIII
M.musculus_NCX3.1     TAAWSIFAYIWLYMILAVFSPGVVQVWEGLLTLFFFPVCVLLAWVADKRLLFYKYMHKKYRTDKHRGIII
G.gallus_NCX3         TAAWSIFAYIWLYMILAVFSPGVVQVWEGLLTLFFFPLCVLLAWIADRRLLFYKYMHKKYRTDKHRGIII
D.rerio_NCX4a         TAAWSIFAYIWLYLILSVFSPGVVEVWEAVLTFLFFPLCVVQAWIADRRLLFYKYVHKRYRTDKNRGIII
T.nigroviridis_NCX4a  TAAWSIFAYIWLYLILSVFSPGEVQVWEAVLTFCFFPLCVLQAWIADRRLLFYKYVHKRYRADKTRGIII
H.sapiens_NCX2.1      TASWSIFAYVWLYLILAVFSPGVVQVWEALLTLVFFPVCVVFAWMADKRLLFYKYVYKRYRTDPRSGIII
R.norvegicus_NCX2.1   TASWSIFAYVWLYLILAVFSPGVVQVWEALLTLVFFPVCVVFAWMADKRLLFYKYVYKRYRTDPRSGIII
M.musculus_NCX2.1     TASWSIFAYVWLYLILAVFSPGVVQVWEALLTLIFFPVCVVFAWMADKRLLFYKYVYKRYRTDPRSGIII
S.kowalevskii_3       TATCSLFAYIWLYMILAVFSAGIIEVWEALLTFMFFPLLVVVAYLVDTRVFFMKFLRKKYKAKTMPGESR
T.adhaerens_2         TASCSVFAYLWLLLILKGISRDVIDIWEAVVTLLFFPILVTIAWVADRQLLSFKKLSKKYRADGKNRAVI
L.opalescens_NCX      TATWSVFAYLWLYFILAVSSYGVVDVWEGLLTFMFFPATVVTAYIADRRLLNKYLSKKYRASKQKGVIVQ
A.mellifera_NCX       TATWSIFAYVWLYLILAVSSPGVLEVWEGILTFSFFPATVLTAYVADRRLLIYKYLHKGYRMNKRGVIVQ
D.melanogaster_NCX    TALFSVFAYVWLWLILSVFTPGVILVWEAIVTLLFFPLTVLWAYIAERRLLVYKYMDKNYRVNKRGTVVA
H.magnipapillata      TASCSMFAYIWLYLILSVFSPGVIEIWEGIITFLCFPAMVIAAWAIDRKINFYHLLRKKIRKVKKRGQAF
C.elegans_NCX1        TVVWSTFAYVWLYLILSVFSPGEVEVWEGVLTFVFFPLTVGSAYFADAHAGQFGQRLISGPLSSFVRRSP
C.briggsae_NCX1       TVVWSTFAYVWLYLILSVFSPGEVEVWEGVLTFVFFPLTVASAYFADAHAGQFGQRLISGPLSSFVRRSP
C.elegans_NCX2        TATWSVFAYVWLYLILAFFSPGEIEIWEGALTFIFFPLTVFTAYMADIKLIQNKFLPHRYRRGSHGQMIA
C.briggsae_NCX2       TATWSIFAYVWLYLILAFFSPGEIEIWEGLLTFVFFPLTVGTAYMADIKIIQNRFLPHRYRRGSHGQMIA
T.spiralis            TTGWSVFAYIWLIIVLVLVSPNRIEVWEGLLTLLFFGALLLHAYLVSKKLCSGGRGRGRPTTERDRTPKA
M.brevicollis_1       TAIFSIFAYIWLLIILIASSPDVIEIWEGVVTLLFFPILLGLSLMADRGYFDFSRDQKPHPNEEELQVIN
Salpingoeca           TAIFSVFAYVWLIIILVVSSPNVVEVWEGVLTLLFFPMLLGLSFMADRGYFDFGKRHPASVDNVLR..ID
S.kowalevskii_1       TSFFCIFAYIWLFLVLVVSSPDVVELWEAIFTFLCFPILVILAFVADKDYCGKKEVEDAELARLGIGHPG
S.kowalevskii_2       TAAFSIIAYIWLLLVLLVITPNIVELWEAICTFICFPILVVIAFMADKGYRGRNVAKDDDKQAL......
T.adhaerens_1         TATFSIFAYLWLYIVLVARTKNIVDLTEAIITLCFFVLVLISYFVDRNFFCPR...........KARRIS
D.pulex_1             TAVFSILAYLWLIFVLMGVSKNVVEVWEAVVTFLLFPILVYLAYLADKGFPWNKSRVSATTANGKQIELG
P.humanus_corporis    CSIFSILAYVWLWIILSLFSPHVVELWEAIATFLMFPALIFFAFATDKKWLDIRLLMSKKKRLTRAHEIK
O.dioica              TAFFSIFAYVWLLIILQVTSPGVIDLWEALVTLLMFPLLVGVAYLADRNMLCGQTRTDNDDEGQKQIMEY
A.pisum               IGAFSFFAYIWLLLIIEVISPNVIELWEAAATFLLFPILMFFAYATDQNWCGKST...............
P.marinus             TAGCSIFAYIWLLLVLQVSSPDVVDIWEAVVTLVLFPALVVM.....................AFAADKG
A.anophagefferens_1   TAFFSVFAYVWLLFILMVTSPDVVEIWEAIFTFLFFPILVAL.....................AYAADRG
E.huxleyi             TAAASVFAYVWLAYILLVSSPNVIEVWEGAATLAFFPLLVWLSHRCGIRVPPPTLSAQLRTMLAYLADVG
consensus>50          ta.wsifAYiW$yi!l.v.sp.vvevwEallT.lffpllv..ay..d.r..y.....r.....d.....
```

```
                                 250
A.thaliana              IDTSKDDNDND...........................................................
A.halleri               IDTSKDDNDND...........................................................
A.lyrata                IDTSKDENDND...........................................................
C.sativus               IDICKQDNPCR...........................................................
C.clementina            TTSCKDENCDY...........................................................
R.communis              ATSDKHQHNAY...........................................................
M.esculenta             ITSSKHQPIDY...........................................................
P.trichocarpa           VPSPTHENNVY...........................................................
P.persica               VVTCKSDSGPC...........................................................
C.papaya                VASVDHDNNDK...........................................................
A.coerulea              ASLDKLDNNDY...........................................................
V.vinifera              AASRKQDKIAG...........................................................
S.tuberosum             VVKHRPLDKVH...........................................................
S.lycopersicum          VVKYRPLDKVH...........................................................
M.guttatus_1            TAPYKDRDKYS...........................................................
M.guttatus_2            ..KNAPYRDGN...........................................................
G.max                   TPYFQHEAHAE...........................................................
M.truncatula            TPKQKS..HER...........................................................
E.grandis               AASYKHVDNVD...........................................................
S.italica               GAS.VDYDNCN...........................................................
Z.mays                  DVSAVYYAKCD...........................................................
S.bicolor               DVS.VDYDNCD...........................................................
T.aestivum              DTSLHHDKNCD...........................................................
B.distachyon            DTSVDHDKNCG...........................................................
O.sativa_J1             DASVDYDDNYD...........................................................
O.sativa_I1             DASVDYDDNYD...........................................................
O.sativa_J2             DASVDYDDNYD...........................................................
S.moellendorffii_1      LHELAN................................................................
P.patens_1              FSLSVHNGTVHDQSTAYSSSPRVDLEQHRS........................................
P.patens_2              YFSLAIYDGEIHDQSASFSNLEVFSEQSTS........................................
C.reinhardtii           PPSVTAIVVSPAPEQAPGEAP.................................................
C.intestinalis          DPAMVNGLSSRRGSAVGVKSPEKSRKMNWTNEGVCSVSNGDICGGTPNMGSPALKRHIRRVSIAAPTNPA
B.floridae              NVRVIGSPQGRASVLNTR...................................................A
T.adhaerens_3           VGGKSRHPSASIIRSTSRELSNVHRESVASAHNSTYDLESVTKLNPGENRSRANSARTSIS.........
N.vectensis_2           FTKGLVDRNSLALLRFVRFVKQARNCDVVSKTSRFSHLQVRVLGFTDAPSSHAMIHRSMEL.........
H.sapiens_NCX1.1        EHEGDRPS.SKTEIEMDGKVV.................................................
P.troglodytes_NCX1.1    EHEGDRPS.SKTEIEMDGKVV.................................................
C.familiaris_NCX1.1     EHEGDRPS.SKTEIEMDGKVV.................................................
F.catus_NCX1.1          EHEGDRPS.SKTEIEMDGKVV.................................................
C.porcellus_NCX1.1      EHEGDRPS.SKTEIEMDGKVV.................................................
B.taurus_NCX1.1         EHEGDRPS.SKTEIEMDGKVV.................................................
R.norvegicus_NCX1.1     EHEGDRPA.SKTEIEMDGKVV.................................................
M.musculus_NCX1.1       EHEGDRPA.SKTEIEMDGKVV.................................................
M.mulatta_NCX1.3        EHEGDRPS.SKTEIEMDGKVV.................................................
O.cuniculus_NCX1.2      EHEGDRPS.SKTEIEMDGKVV.................................................
S.tropicalis_NCX1       ETEGDRPS.SKADIEMDGKVL.................................................
O.mossambicus_NCX1.1    ETEGEPELPSKVDIEMDGKML.................................................
O.mykiss_NCX1           ETEGEAQIPSKMDIEMDGKML.................................................
D.rerio_NCX1a           ETEGEPELQSKADIEMDGGML.................................................
H.sapiens_NCX3.3        ETEGDHPKG....IEMDGKMM.................................................
R.norvegicus_NCX3.3     ETEGEHPKG....IEMDGKMM.................................................
M.musculus_NCX3.1       ETEGDHPKG....IEMDGKMM.................................................
G.gallus_NCX3           ESEGDHPKG....IEMDGKMM.................................................
D.rerio_NCX4a           ETEGDGMFT.KMDMEMDGQGA.................................................
T.nigroviridis_NCX4a    ESEGDAMFT.KMDMEMDGQGV.................................................
H.sapiens_NCX2.1        GAEGDPPKSIELDGTFVGAEA.................................................
R.norvegicus_NCX2.1     GAEGDPPKSIELDGTFVGTEV.................................................
M.musculus_NCX2.1       GAEGDPPKSIELDGTFVGTEV.................................................
S.kowalevskii_3         GNHYP.................................................................
T.adhaerens_2           VEPDSI.......IMRSPKEE.................................................
L.opalescens_NCX        CEGQDAEAGEGKSEDG......................................................
A.mellifera_NCX         AEAGDSDGGVELEIKPQQDSF.................................................
D.melanogaster_NCX      GEHDQVEMDAEKGPKQPMVTS.................................................
H.magnipapillata        IQTGDGDVVAVSLKSTIKHDS.................................................
C.elegans_NCX1          R.............RSPSKKT.................................................
C.briggsae_NCX1         R.............RSPSKKT.................................................
C.elegans_NCX2          T.............EAEEMKM.................................................
C.briggsae_NCX2         T.............EAEEMKM.................................................
T.spiralis              LRELNRRSWTGPALNGKEKLL.................................................
M.brevicollis_1         IRT...................................................................
Salpingoeca             NDV...................................................................
S.kowalevskii_1         DRY...................................................................
S.kowalevskii_2         ......................................................................
T.adhaerens_1           DTE...................................................................
D.pulex_1               SIQ...................................................................
P.humanus_corporis      MEE...................................................................
O.dioica                NPD...................................................................
A.pisum                 ......................................................................
P.marinus               MFS...................................................................
A.anophagefferens_1     YFD...................................................................
E.huxleyi               AFS...................................................................
consensus>50            ......................................................................
```

```
A.thaliana              ......................................................................
A.halleri               ......................................................................
A.lyrata                ......................................................................
C.sativus               ......................................................................
C.clementina            ......................................................................
R.communis              ......................................................................
M.esculenta             ......................................................................
P.trichocarpa           ......................................................................
P.persica               ......................................................................
C.papaya                ......................................................................
A.coerulea              ......................................................................
V.vinifera              ......................................................................
S.tuberosum             ......................................................................
S.lycopersicum          ......................................................................
M.guttatus_1            ......................................................................
M.guttatus_2            ......................................................................
G.max                   ......................................................................
M.truncatula            ......................................................................
E.grandis               ......................................................................
S.italica               ......................................................................
Z.mays                  ......................................................................
S.bicolor               ......................................................................
T.aestivum              ......................................................................
B.distachyon            ......................................................................
O.sativa_J1             ......................................................................
O.sativa_I1             ......................................................................
O.sativa_J2             ......................................................................
S.moellendorffii_1      ......................................................................
P.patens_1              ......................................................................
P.patens_2              ......................................................................
C.reinhardtii           .....................VRTHAHYRHILAARQRHAAAAHRRLPGAHGEGGDTELQLGSGGHAYGDV
C.intestinalis          AIELASISKKDNGRKESERRQVEPEPAYRAFARARFRHAAIWSIGGRKRKPINLNLQPSPKLPVNDPKLL
B.floridae              PTELLALEGRNRNLNDMDRPSQQGQQPTNALSRARFRHAVVRSMLYQK............KAPKPKARFA
T.adhaerens_3           .........RSSFVYEDHGKIQVDIKPSNSFARARFRHAAIRSLMGGKRHRRSKSVTDKMVELESGLFNS
N.vectensis_2           ..................RALEQHRGSVRNSSFDRFRHAALRSVTRSKKLIKINEGEDVE..........
H.sapiens_NCX1.1        .............................NSHVENFLDGALVLEVDERDQD.......DEEARREMARIL
P.troglodytes_NCX1.1    .............................NSHVENFLDGALVLEVDERDQD.......DEEARREMARIL
C.familiaris_NCX1.1     .............................NSHVDNFLDGALVLEVDERDQD.......DEEARREMARIL
F.catus_NCX1.1          .............................NSHVDNFLDGALVLEVDERDQD.......DEEARREMARIL
C.porcellus_NCX1.1      .............................NSHVENFLDGALVLEVDERDQD.......DEEARREMARIL
B.taurus_NCX1.1         .............................NSHVDSFLDGALVLEVDERDQD.......DEEARREMARIL
R.norvegicus_NCX1.1     .............................NSHVDNFLDGALVLEVDERDQD.......DEEARREMARIL
M.musculus_NCX1.1       .............................NSHVDNFLDGALVLEVDERDQD.......DEEARREMARIL
M.mulatta_NCX1.3        .............................NSHVENFLDGALVLEVDERDQD.......DEEARREMARIL
O.cuniculus_NCX1.2      .............................NSHVDNFLDGALVLDVDERDQD.......DEEARREMARIL
S.tropicalis_NCX1       .............................NSHTETFLDGSLVLEVDEKDQD.......EEEARRDMAKIL
O.mossambicus_NCX1.1    .............................NSHGEEFTEELE..G...KELD.......EEEARREVARIL
O.mykiss_NCX1           .............................NS..ESFMDGAM..GFDEKDLD.......EEEARREMVRIL
D.rerio_NCX1a           .............................NSHAEEFLDGAV..DNEDKDAD.......EDEARREMAKIL
H.sapiens_NCX3.3        .............................NSHFLDGNLVPLEGKEVD.............ESRREMIRIL
R.norvegicus_NCX3.3     .............................NSHFLDGNLIPLEGKEVD.............ESRREMIRIL
M.musculus_NCX3.1       .............................NSHFLDGNFTPLEGKEVD.............ESRREMIRIL
G.gallus_NCX3           .............................NSHFLDGNLVTVEGKEVD.............ESRKEMIRIL
D.rerio_NCX4a           .............................NSHH..KEALDGMLAGVEEGGGGGEE....EEARREMARTL
T.nigroviridis_NCX4a    .............................NSHTHPKEALDGMLEGMEEGGGMISEQDQEEEARREMARTL
H.sapiens_NCX2.1        .............................PGELGGLGPGPAEARELDA.............SRREVIQIL
R.norvegicus_NCX2.1     .............................PGELGALGTGPAEARELDA.............SRREVIQIL
M.musculus_NCX2.1       .............................PGELGALGTGPAEARELDA.............SRREVIQIL
S.kowalevskii_3         ................................LSHEYDDVEYRGRFEDDELDFEDIKDLDESRQEAIRIL
T.adhaerens_2           .............................RDSEGFNSVYKNNPMYDVDSIDDVDSEKNDEMRRKKAMQIV
L.opalescens_NCX        .............................ALKEGGDDVEVREF................EQHRKEYIEIL
A.mellifera_NCX         .............................HNMMDADTPEAKEF................EQTRRDYINTL
D.melanogaster_NCX      .............................ARGNDAEA.....F................DEARREYITLL
H.magnipapillata        .............................SNDNVDEKDIELLPLEEDDDPLEIINDKK.....KKAMEAF
C.elegans_NCX1          .............................RENVENGA..GLPGDATQNLIGGDADALAFEIHRRHYLDIF
C.briggsae_NCX1         .............................HENALESGNGGIPGDQNQSLIGGDADALAFEIHRRHYLDIF
C.elegans_NCX2          .............................LENGTQG................DPALKAFEEHRQEFIELM
C.briggsae_NCX2         .............................LENGSPQ..............GADPALKAFEEHRQEFIELM
T.spiralis              .............................DVVEQNRVD.............................RLS
M.brevicollis_1         ..............................EDGRNIAFDPYDM.........................AV
Salpingoeca             ..............................EHGHQFHDPYEAD.........................TI
S.kowalevskii_1         ..............................LTAENVTKEGVADVM............KEIGKHPDITPEQ
S.kowalevskii_2         ..............................LGIGNVTREGVADVM............KEMGKHPDITQEQ
T.adhaerens_1           ..............................NGHLAFDDSTGRLVPDSFDKSSANSFLKAVKTRTDITAEE
D.pulex_1               ..............................PGELEAMVDGLTLTKDGQVDPNELAKWVRQASRMGLTGED
P.humanus_corporis      ..............................SKKIFVERKFIKDGKI..DKETLYSFFKELKKYPGISNED
O.dioica                ..............................TPEGIELLGKQRPNSMALVDEFMKDFEGDPLTIDEQAAAI
A.pisum                 ...................................EHNTHQLDLDLGQNKENLIKFVKNVKKYSGITDAD
P.marinus               ..............................IKTNVASAHRDHIVGLEDLSPDELVD............LM
A.anophagefferens_1     ..............................TAKISPVSSAHVIQVGSTPFRPYEFS............DL
E.huxleyi               ..............................CPDKARPATHVVVAFTRDGKPIRHRSFAREAATQWARGDL
consensus>50            ......................................................................
```

```
A.thaliana              ......................................................................
A.halleri               ......................................................................
A.lyrata                ......................................................................
C.sativus               ......................................................................
C.clementina            ......................................................................
R.communis              ......................................................................
M.esculenta             ......................................................................
P.trichocarpa           ......................................................................
P.persica               ......................................................................
C.papaya                ......................................................................
A.coerulea              ......................................................................
V.vinifera              ......................................................................
S.tuberosum             ......................................................................
S.lycopersicum          ......................................................................
M.guttatus_1            ......................................................................
M.guttatus_2            ......................................................................
G.max                   ......................................................................
M.truncatula            ......................................................................
E.grandis               ......................................................................
S.italica               ......................................................................
Z.mays                  ......................................................................
S.bicolor               ......................................................................
T.aestivum              ......................................................................
B.distachyon            ......................................................................
O.sativa_J1             ......................................................................
O.sativa_I1             ......................................................................
O.sativa_J2             ......................................................................
S.moellendorffii_1      ......................................................................
P.patens_1              ......................................................................
P.patens_2              ......................................................................
C.reinhardtii           VVPA....................DPTKEQVMFRSRAYAFLESAGTARVAVTR.................
C.intestinalis          SVVSKIASIRKLYSNNNAIQDES...SGN.VAFASSAYSTLESAGELTIDVLLHRRKRPKG.........
B.floridae              EVVTKMVSIKALTSPAMRRQLAANDMSGK.FTFGTHTYSVLESSGAIEVDVLFHRRRLSKL.........
T.adhaerens_3           SEPKMAHFVNIITHRLNEQSNASSRNIAATFVFEIAAYSVREDSGSIDLIVLLNRHRALIKKSQENKNNK
N.vectensis_2           ........................DDNLNRFVFGTSSYSVLKSAKHIDLEIILLQN..............
H.sapiens_NCX1.1        KELKQKHPDKEIEQLIELANYQVLSQQQKSRAFYRIQATRLMTGAGNILKRHAADQA......RKAVSMH
P.troglodytes_NCX1.1    KELKQKHPDKEIEQLIELANYQVLSQQQKSRAFYRIQATRLMTGAGNILKRHAADQA......RKAVSMH
C.familiaris_NCX1.1     KELKQKHPEKEIEQLIELANYQVLSQQQKSRAFYRIQATRLMTGAGNILKRHAADQA......RKAVSMH
F.catus_NCX1.1          KELKQKHPEKEIEQLIELANYQVLSQQQKSRAFYRIQATRLMTGAGNILKRHAADQA......RKAVSMH
C.porcellus_NCX1.1      KELKQKHPEKEIEQLIELANYQVLSQQQKSRAFYRIQATRLMTGAGNILKRHAADQA......RKAVSMH
B.taurus_NCX1.1         KELKQKHPEKEIEQLIELANYQVLSQQQKSRAFYRIQATRLMTGAGNILKRHAADQA......RKAVSMH
R.norvegicus_NCX1.1     KELKQKHPDKEIEQLIELANYQVLSQQQKSRAFYRIQATRLMTGAGNILKRHAADQA......RKAVSMH
M.musculus_NCX1.1       KELKQKHPEKEIEQLIELANYQVLSQQQKSRAFYRIQATRLMTGAGNILKRHAADQA......RKAVSMH
M.mulatta_NCX1.3        KELKQKHPEKEIEQLIELANYQVLSQQQKSRAFYRIQATRLMTGAGNILKRHAADQA......RKAVSMH
O.cuniculus_NCX1.2      KELKQKHPEKEIEQLIELANYQVLSQQQKSRAFYRIQATRLMTGAGNILKRHAADQA......RKAVSMH
S.tropicalis_NCX1       KELKQKHPEKEIEQLIELANYQVLSQQQKSRAFYRIQATRLMTGAGNILKRHAADQA......RKAVSMH
O.mossambicus_NCX1.1    KELKQKHPEKEMEQLMELANYQVLTQQQKSRAFYRCQATRIMTGAGNVLKKHACDQA......KRAT..H
O.mykiss_NCX1           KELKQKHPEKETEQLIELANYQVLTQQQKSRAFYRCQATRIMTGAGNVLKKHAADQA......RKAVGAY
D.rerio_NCX1a           KELKQKHPEKEMEQLVELANYHVLSQQQKSRAFYRCQATRLMTGAGNILKKHAADQA......RKALGIH
H.sapiens_NCX3.3        KDLKQKHPEKDLDQLVEMANYYALSHQQKSRAFYRIQATRMMTGAGNILKKHAAEQA......KKASSMS
R.norvegicus_NCX3.3     KDLKQKHPEKDLDQLVEMANYYALSHQQKSRAFYRIQATRMMTGAGNILKKHAAEQA......KKTASMS
M.musculus_NCX3.1       KDLKQKHPEKDLDQLVEMANYYALSHQQKSRAFYRIQATRMMTGAGNILKKHAAEQA......KKTSSMS
G.gallus_NCX3           KDLKQKHPEKDLDQLVEMANYYALSHQQKSRAFYRIQATRMMTGAGNILKKHAAEQA......KKSTSLH
D.rerio_NCX4a           KELKQRHPEKDMEQLIEMANYQVLVQQQKSRAFYRIQATRMMIGAGNILKKHAADQA......RKVVSCH
T.nigroviridis_NCX4a    KELKQRHPEKDIEQLIEMANYQVLIQQQKSRAFYRIQATRMMIGAGNILKKHAADQA......RKVVSCH
H.sapiens_NCX2.1        KDLKQKHPDKDLEQLVGIANYYALLHQQKSRAFYRIQATRLMTGAGNVLRRHAADAS......RRAAPAE
R.norvegicus_NCX2.1     KDLKQKHPDKDLEQLVGIAKYYALLHQQKSRAFYRIQATRLMTGAGNVLRRHAADAA......RRPGAND
M.musculus_NCX2.1       KDLKQKHPDKDLEQLMGIAKYYALLHQQKSRAFYRIQATRLMTGAGNVLRRHAADAA......RRPGATD
S.kowalevskii_3         RDLKKKHPEADHETLEQMANYEALNRQTKSRAFYRIQATRKMVGAGNILKKNLD.........KKRTSIA
T.adhaerens_2           KEIKQNHPNATAKEIEELASYEALKYQPKSRAYYRIQATRKLTGSGNVIKQRKM..S......LPANPDG
L.opalescens_NCX        REMRKKNPTLDMKTLEDMAESEAVNRGPKSRAFYRIQATRKLTGSGNIIKKAKAQAGVAQPIVIDQKPED
A.mellifera_NCX         RELRKKYPALPLEQLEVMAHEEVLGKGPKSRAFYRVQATRKMVGAGNLSKKISERAQSDLSEVKAELQKQ
D.melanogaster_NCX      TELRQKYPDADLEQLEMMAQEQVLARSGRSRAFYRIQATRKMVGSGNLMRKIQERAHSDLTGVKAQLHAG
H.magnipapillata        HRARTKYPNADSITLKKLIEQENIKLQHKSRAFYRVQATHALTGQGNLLRLKDRDNSIELN.....NKME
C.elegans_NCX1          KQLRSEHPDAPVVELEKHAMEKVVGEQKKSRAFYRIQTTRKMIGSGDIQKKLK.KSN......KLEPMVV
C.briggsae_NCX1         KQLRSEHPDAPVDELEKHAMEKVVGEQKKSRAFYRIQTTRKMIGSGDIQKKMKNKKG......LLEPLVV
C.elegans_NCX2          REIRKQNPHITPTELQKQAEYEMISRGPKSRAFYRVQATRRLIGGGDIVKKRIDKEH......NKALDA.
C.briggsae_NCX2         REIRKQNPHITPTELQKQAEYEMISRGPKSRAFYRVQATRRLIGGGDIVKKRIDKEH......NKALDAL
T.spiralis              RELRRKYPHLTDDSLAEVVAKQIEKEAPHDHLWYRIMAVRRLASSMRSRVRAKPVSEQVCQKLETLSRTG
M.brevicollis_1         VLKDVETEALTEDEIANIAAARKIDGTKVSRARFRIDANRRLRGAAPLLPDWQRALLD.....TQKSRAI
Salpingoeca             IKRIARSNSMTMEDKVNLALAKKLKGERVSRAQFRIDATRKLVGSGGILPDYNKAKLQ.....EALLR..
S.kowalevskii_1         AATIAAIEAEKNSPH..............TKAWYRVNATRNMTGGQKLIPAVDPKLLSAYDQAKMGSAIS
S.kowalevskii_2         AATIAAIEAEKNSPH..............TRAWYRVSETRNMTGGQKLIPTVDPKLLLAYDQVKMGSSIS
T.adhaerens_1           AAKLAAAKLQEQEKK..............SYIWYRVGAARLVSGSKRVTPLLDARLQKIFDDIEMKNKKF
D.pulex_1               AAKLAAYKLITSKEH..............SRAWYRIGATRIFSGSKKIQPQLSIKLREVYDAINEHPDAV
P.humanus_corporis      AAILAASKIIDSEPQ..............SAATYRMSAIRDLTGGRKLRPRLSLKLQEVYDTINQYPLGS
O.dioica                AVASKMQGSFCVVPSFHVTKNLQCPTQGRSRAWYRVQATRTLTAGARIEPN............KALSRMA
A.pisum                 AAKLAATKLIDSTH..............RGAMWYRIGAVRRFTGGKQIEPVLEDHLRQ............
P.marinus               AEVTRRRRLQERCPSQIRQRHGSSLTDDAVMRIIQAERPPRTTRAQHRIQASRMLTRRSGASKRSSVWNS
A.anophagefferens_1     LKKLQDPTLEESERHDLVKKLAMGQKAKPS.............RAVLRMNAVRAATGQKVIKADGPDPKV
E.huxleyi               ARELQRMDPDAADGTSDAQGLASYLASSLGTLRSRAAYRVATTRGATGVAAPQARAAAEQEVAPPPSAAV
consensus>50            ...............................a.yr....r...g..........................
```

```
A.thaliana             ......................................................................
A.halleri              ......................................................................
A.lyrata               ......................................................................
C.sativus              ......................................................................
C.clementina           ......................................................................
R.communis             ......................................................................
M.esculenta            ......................................................................
P.trichocarpa          ......................................................................
P.persica              ......................................................................
C.papaya               ......................................................................
A.coerulea             ......................................................................
V.vinifera             ......................................................................
S.tuberosum            ......................................................................
S.lycopersicum         ......................................................................
M.guttatus_1           ......................................................................
M.guttatus_2           ......................................................................
G.max                  ......................................................................
M.truncatula           ......................................................................
E.grandis              ......................................................................
S.italica              ......................................................................
Z.mays                 ......................................................................
S.bicolor              ......................................................................
T.aestivum             ......................................................................
B.distachyon           ......................................................................
O.sativa_J1            ......................................................................
O.sativa_I1            ......................................................................
O.sativa_J2            ......................................................................
S.moellendorffii_1     ......................................................................
P.patens_1             ......................................................................
P.patens_2             ......................................................................
C.reinhardtii          ................................................VAPEGGSLDHPLRVHYRTEDGD
C.intestinalis         ............QTVIPTTNANGKFDTQIMPGVI..........................SVDYETREGT
B.floridae             ............TLLNSMSKLNLVNKSNNGPNNGPVVNGTTTDSGQSSAPSKQDEAKEDVSVEFETRDGT
T.adhaerens_3          TVTFGLGKSTSETIIKSLSSSFKTDKSIKSCDPITEESIQDLESSRYSDEEGDDATQNNFSVDFETRDGG
N.vectensis_2          ...................NNNNKNKEMIYAHNDSIADTISENSFEDGVTPSSPGSSQRFSVDYETHDGS
H.sapiens_NCX1.1       EVNTEVTENDPVSK............IFFEQGTYQCLENCGTVALTIIRRGGDL..TNTVFVDFRTEDGT
P.troglodytes_NCX1.1   EVNTEVTENDPVSK............IFFEQGTYQCLENCGTVALTIIRRGGDL..TNTVFVDFRTEDGT
C.familiaris_NCX1.1    EVNTEVAENDPVSK............IFFEQGTYQCLENCGTVALTIIRRGGDL..TNTVFVDFRTEDGT
F.catus_NCX1.1         EVNTEVAENDPVSK............IFFEQGTYQCLENCGTVALTILRRGGDL..TNTVFVDFRTEDGT
C.porcellus_NCX1.1     EVNTEVAENDPVSK............IFFEQGTYQCLENCGTVALTIIRRGGDL..TNTVFVDFRTEDGT
B.taurus_NCX1.1        EVNTEVAENDPVSK............IFFEQGTYQCLENCGTVALTIIRRGGDL..TNTVFVDFRTEDGT
R.norvegicus_NCX1.1    EVNMDVVENDPVSK............VFFEQGTYQCLENCGTVALTIIRRGGDL..TNTVFVDFRTEDGT
M.musculus_NCX1.1      EVNMEMAENDPVSK............IFFEQGTYQCLENCGTVALTIMRRGGDL..STTVFVDFRTEDGT
M.mulatta_NCX1.3       EVNTEVTENDPVSK............IFFEQGTYQCLENCGTVALTIIRRGGDL..TNTVFVDFRTEDGT
O.cuniculus_NCX1.2     EVNTEMAENDPVSK............IFFEQGTYQCLENCGTVALTIIRRGGDL..TNTVFVDFRTEDGT
S.tropicalis_NCX1      EVNTDVVENDPVSK............IYFEQATYQCLENCGTVALTIVRRGGDL..TNTVYVDFRTEDGS
O.mossambicus_NCX1.1   DIFSEVSVNDFSSK............VFFDPGTYQCLENCGSVALNVVRRGGDL..TSTVSVDYRTEDGT
O.mykiss_NCX1          EIRSEVSENDFSSK............VFFDPGTYQCLENCGTVALNVVRLGGDL..TNTVSVEYRTEDGT
D.rerio_NCX1a          ELRSEVSDNDISSK............IFFDPGTYQCLENCGTVALNVVRRGGDL..TSTVSVEYRTEDGT
H.sapiens_NCX3.3       EVHTDEPE.DFISK............VFFDPCSYQCLENCGAVLLTVVRKGGDM..SKTMYVDYKTEDGS
R.norvegicus_NCX3.3    EVHTDEPE.DFASK............VFFDPCSYQCLENCGAVLLTVVRKGGDI..SKTMYVDYKTEDGS
M.musculus_NCX3.1      EVHTDEPE.DFASK............VFFDPCSYQCLENCGAVLLTVVRKGGDI..SKTMYVDYKTEDGS
G.gallus_NCX3          EVRPDEPE.EFISK............IYFDPCSYQCLENCGAVLLTVVRKGGDV..SKTIYVDYKTEDGS
D.rerio_NCX4a          EANPQE.EDPHTIY............LEFEPSHYQCFENCGSLKLSVTRHGGDS..GCTVKVDYRTEDGT
T.nigroviridis_NCX4a   EASGQE.EDPHTIY............LQFEPSHYQCFENCGSLKLSVSRYGGES..GCTVKVDYRTEDAT
H.sapiens_NCX2.1       GAGED..EDDGASR............IFFEPSLYHCLENCGSVLLSVTCQGGEG..NSTFYVDYRTEDGS
R.norvegicus_NCX2.1    GAPDD..EDDGASR............IFFEPSLYHCLENCGSVLLSVACQGGEG..NSTFYVDYRTEDGS
M.musculus_NCX2.1      GAPDD..EDDGASR............IFFEPSLYHCLENCGSVLLSVACQGGEG..NSTFYVDYRTEDGS
S.kowalevskii_3        EVKIEVPQDQNFTR............VFFEPAEYTVFENVGTFNATVVRRGGDM..NSTIYVDYRSEDGT
T.adhaerens_2          NVDPEDIEKCSLTQ............IYFSPEKYTVLENCGTVGVTVERTGN.L..NNVLTVDYKTQDGT
L.opalescens_NCX       EIT..........R............VSFDPGHYTVMENVGTFYGTVTREGGDL..TKTLYVDYKTEDGT
A.mellifera_NCX        EAESIEIENVNAMR............IFFEPGHYTVMENVGSFEVGVTRAGGDL..SKPCTVDYCTEDGS
D.melanogaster_NCX     DDE....EADDPIR............MYFEPGHYTVMENCGEFEVRVVRRG.DI..STYASVEYETQDGT
H.magnipapillata       EVSASQKDTVLENC............VQFEPDQYTVVESCGQCFLTVKRFGNDL..SDTLYVDYETSDGT
C.elegans_NCX1         QKTMATVEFD....................PPHYTCLENVGDVYLTVKCDRGSVPEDTTVTVHYRTIADT
C.briggsae_NCX1        KQTMATVEFD....................PPHYTCLENVGDVYLTVKCDRGNVPEDTTVTVHYRTIADT
C.elegans_NCX2         ..AQEKQSRDNTCK............IFLDPAHYTVLESVGSFDVVVGRDGG..PDGLTVMVDYFTEDGS
C.briggsae_NCX2        VQAQEKQLRDNTCK............IFLDPAHYTVLESVGSFDVVVGRDGG..PDGLTVMVDYFTEDGT
T.spiralis             MLRRTQTETGVRSV............TNVEFAANAYAVDPTGERRVRLKVVRRGSARKQLVFNYRTMSGS
M.brevicollis_1        KDSYYAHRATALRQEQTFGFMTIEYHVREDAGA............VTLTIVRTGDLSQSASIGIETQEGT
Salpingoeca            .DDFFEAHKTKLAKKNFVGLESLQYSIGEGDGH............VNVKILRTGDLSLPLTIEYYTEGDT
S.kowalevskii_1        VGSTLISPE.....IPIVEFAASSCAVYENEKR............VRVYYVNRRGNIQPQVIFKFETLDGT
S.kowalevskii_2        IGSTLF.PE.....IPIVEFAAASCAVYEHEKR............VRVWINRKGNIHPQIVFKFETLDGT
T.adhaerens_1          SSTADVRDDPETTTRTTIEFTTAACAVMENEKM............VKVGISRTGKLNNTIKVRYETLPGT
D.pulex_1              NLSPLIETVPQNKAI..IEFHAIACAVPESVGR............FPITIVRSGKTDNSVSVRVETIDGS
P.humanus_corporis     ALSLLPEISMKNAIV...EFHSATCAVRENIGS............FFITVCRHGNLEPSVKVRVESVDGS
O.dioica               RSFSNLPNKNNTSDYAMFNFACTSSSVMENAKQ............IELKVMRTGNIMMPATCSYETIDGT
A.pisum                ...PPTLSQNDETKYAIIEFHAPTIAVNENIGR............FPVTIWRHGNLNTKATVRVDSINGT
P.marinus              LPAHLVALKDTVEADEEAVRSESTIPVINFKCSRFAVMEGAGRIVLPLVTSRPLERDVVVRYETLQGTAT
A.anophagefferens_1    LAKYLEGAAGPKHRPEDVVAFFSDVNGKINTKYALLESDKAVTLHVMRSPAEGPMTIKWATRDGTAKGSE
E.huxleyi              LSFAVSGAAFSEGAKE........................ASLVVERSGEELARELDWEGSVEYESRDGT
consensus>50           .............................................................v.y.t.dg.
```

```
A.thaliana              ......................................................................
A.halleri               ......................................................................
A.lyrata                ......................................................................
C.sativus               ......................................................................
C.clementina            ......................................................................
R.communis              ......................................................................
M.esculenta             ......................................................................
P.trichocarpa           ......................................................................
P.persica               ......................................................................
C.papaya                ......................................................................
A.coerulea              ......................................................................
V.vinifera              ......................................................................
S.tuberosum             ......................................................................
S.lycopersicum          ......................................................................
M.guttatus_1            ......................................................................
M.guttatus_2            ......................................................................
G.max                   ......................................................................
M.truncatula            ......................................................................
E.grandis               ......................................................................
S.italica               ......................................................................
Z.mays                  ......................................................................
S.bicolor               ......................................................................
T.aestivum              ......................................................................
B.distachyon            ......................................................................
O.sativa_J1             ......................................................................
O.sativa_I1             ......................................................................
O.sativa_J2             ......................................................................
S.moellendorffii_1      ......................................................................
P.patens_1              ......................................................................
P.patens_2              ......................................................................
C.reinhardtii           AVAGLDYEAREGTLHFAPGEAYKYVEVRIIDDDMTEPDVHFSIVLTGADAPNGAG...............
C.intestinalis          AKINKDFKYTSGTLIFPEDTYVQSISIPIINDDQYYPDTDFYVILKN...PSG.................
B.floridae              GKTGKDYTHTQGRLVFKETEYRKKIKIPIINDQQYQSDKDFYVILKS...PAPRE...............
T.adhaerens_3           AKQGVNFKYSAGRLNFTKSEYQKTITVDILNDGQYHPNLSFYVILKN...PS..E...............
N.vectensis_2           AKNGKEYQTVKGTMAFRAGINKHILRIPILPCEDFSKNKDFYVLLKN...PSPEG...............
H.sapiens_NCX1.1        ANAGSDYEFTEGTVVFKPGDTQKEIRVGIIDDDIFEEDENFLVHLSNVKVSSEASEDGILEA........
P.troglodytes_NCX1.1    ANAGSDYEFTEGTVVFKPGETQKEIRVGIIDDDIFEEDENFLVHLSNVKVSSEASEDGILEA........
C.familiaris_NCX1.1     ANAGSDYEFTEGTVVFKPGETQKEIRVGIIDDDIFEEDENFLVHLSNVKVSSEASEDGILEA........
F.catus_NCX1.1          ANAGSDYEFTEGTVVFKPGETQKEIRVGIIDDDIFEEDENFLVHLSNVKVSSEASEDGILEA........
C.porcellus_NCX1.1      ANAGSDYEFTEGTVVFKPGETQKEIRVGIIDDDIFEEDENFLVHLSNVKVSSEASEDGILEA........
B.taurus_NCX1.1         ANAGSDYEFTEGTVVFKPGETQKEIRVGIIDDDIFEEDENFLVHLSNVKVSLEASEDGILEA........
R.norvegicus_NCX1.1     ANAGSDYEFTEGTVIFKPGETQKEIRVGIIDDDIFEEDENFLVHLSNVRVSSEVSEDGILDS........
M.musculus_NCX1.1       ANAASDYEFTEGTVIFKPGETQKEIRVGIIDDDIFEEDENFLVHLSNVRVSSDVSEDGILES........
M.mulatta_NCX1.3        ANAGSDYEFTEGTVVFKPGETQKEIRVGIIDDDIFEEDENFLVHLSNVKVSSEASEDGILEA........
O.cuniculus_NCX1.2      ANAGSDYEFTEGTVVFKPGETQKEIRVGIIDDDIFEEDENFLVHLSNVKVSSETSEDGILEA........
S.tropicalis_NCX1       ANAGSDYEFTEGTIIFKPGETQKELRVGIIDDDIFEEDENFLVHLSNVRVNAENTEAN.LES........
O.mossambicus_NCX1.1    ANASSDYEFTEGTVVFKPGETEKEIRINIIDDDIFEEDEHFLVHLSNVRVRAEGSNSHE..A........
O.mykiss_NCX1           ANAGSDYQFTEGVVVFNPGETEKEIRIDIIDDDIFEEDEHFLVHLSNVKVISEGTGYVQPRA........
D.rerio_NCX1a           ANAGSDYQFTEGVIIFKPGETEKEIRVDIIDDDIFEEDEHFLVHLSNVKVISEGANNGNPGT........
H.sapiens_NCX3.3        ANAGADYEFTEGTVVLKPGETQKEFSVGIIDDDIFEEDEHFFVRLSNVRIEEEQPEEGMPPA........
R.norvegicus_NCX3.3     ANAGADYEFTEGTVVLKPGETQKEFSVGIIDDDIFEEDEHFFVRLSNVRVEEEQLEEGMTPA........
M.musculus_NCX3.1       ANAGADYEFTEGTVVLKPGETQKEFSVGIIDDDIFEEDEHFFVRLSNVRVEEEQLAEGMLPA........
G.gallus_NCX3           ANAGADYEFTEGTVVLKSGETQKDFAVGIIDDDIFEEDEHFFVRLSNLRVVDNEE.....PP........
D.rerio_NCX4a           ANAGSDYEFAEGTLVFKPGETVKELTVGVIDDDIFEEDEHFYVHLSNPRVVHRAEVSILDPNAVSPGNSI
T.nigroviridis_NCX4a    ASAGSDYEFAEGTLVFKPGETTKEFTVGIIDDDIFEEDEHFYQQHGGSFLSR..................
H.sapiens_NCX2.1        AKAGSDYEYSEGTLVFKPGETQKELRIGIIDDDIFEEDEHFFVRLLNLRVGDAQGMFEPDGGGR......
R.norvegicus_NCX2.1     AKAGSDYEYSEGTLVFKPGETQKELRIGIIDDDIFEEDEHFFVRLLNLRVGDAQGMFEPDGGGR......
M.musculus_NCX2.1       AKAGSDYEYSEGTLVFKPGETQKDLRIGIIDDDIFEEDEHFFVRLLNLRVGDAQGMFEPDGGGR......
S.kowalevskii_3         ANAGSDFHYAEGTLVFKPGETQKNIQLTIIDDDIFEEDEHFYIRLSNVRVGTADGMFESNHAG.......
T.adhaerens_2           ANSGEDYVTTNGTLRFKAGESRKDIKITIIDDDIYEEDEYFYVLLSNAKFVSTTDNEQ............
L.opalescens_NCX        ANAGSDYVYAEGTLVFYPMETHKQFPISIIDDDIFEEDEHFYIRLSNLRVGDSNGLFESGQAE.......
A.mellifera_NCX         AEAGSDYISAKGTLTFDSGETKKTIKLSVIDDELFEEDEHFYVRLSNASQP...................
D.melanogaster_NCX      ASAGTDFVGRKGLLSFPPGVDEQRFRIEVIDDDVFEEDECFYIRLFNPSEG...................
H.magnipapillata        ANEGDDYLPVKGTLEFKPNETTKKISITIIDDDLFELDEHFYCKLTAIRYYHVADNTQNKS.........
C.elegans_NCX1          AQAESDFVHTEGTITFEPGQTEQKIKVGIVDNDIYEDDEQFMVRLSQVRAFRSEHFSSVPARLG......
C.briggsae_NCX1         AQENSDFMPAEGTITFEPGQTEQKIKVGIVDNDIYEDDEQFMVRLSQVRAFRSEHFSAVPCRLG......
C.elegans_NCX2          ANAGSDYIPVKGTLTFYPEDKHQKVTIEVVDDDVFEEDEHFYLRLCNLRVRTKDGIIIDPTRIG......
C.briggsae_NCX2         ANAGSDYIPVKGTLTFYPEDKHQKVTIEVVDDDVFEEDEHFYLRLTNLRVRTKDGIIIDPTRIG......
T.spiralis              AVQDVHFLKKSETVVFAPQEREKTIRIEVCNPAQWRPGITFWVKLELIPGDKDDRITLGLASAAKIIYPK
M.brevicollis_1         AKAGSDYVALDRRVEFAAGQAEARVSIEIVDDDEPEDDEFFLARLKQPQP.......D............
Salpingoeca             ATMGVDFEETKGVAEFSAGEEEKVIPIKIIDDDEEEDDEHFYFYIKNPSP.......E............
S.kowalevskii_1         AEAGSDYIAQRDTMVFGSNETQKFIDIDIIDDNEWEPDEVFFVKLSVDSKSG...AK.............
S.kowalevskii_2         AEAGSDYIAQRDTMVFGSNETQKFIDIDIIDDNEWEPDEVFFVKLSVDSKSG...AK.............
T.adhaerens_1           AEPEKDYIPQNDVLVFEPNEKIKYVTIPIVDDNEWEPDENFFIQIWLEGSYD..................
D.pulex_1               AVGGQDFVTFNQVIQFEAGELEKIVTMEIIDDNQYEPDEQFYIKLSLTGTKA...IKQ............
P.humanus_corporis      AKAGDDYVKVDEILVFEPEETEKLVLVKIVDDTQWEPNEEFFLKLSLTEHSG...NAN............
O.dioica                ATKGEDYVEAKGVLEFREKETQKSITVEIIDDDQWEPDETFFVKLFLCVSRLVPNAAD............
A.pisum                 AKEGEDFVKVKEIVEFKENEEEKEIYIEILDDNKWEPDEEFFLRLSVVHH......KD............
P.marinus               AGEDYVPVIDGKITIRAGESSSDEVTIAIMDDDIVEEDENFWVRVTHVSEGAVI................
A.anophagefferens_1     VGGTGDYETCSGELTFADKENFKTIVIKVFDDNETEDDEVFYVDLLEVAHGGKAYAPG............
E.huxleyi               AHAGQDYTAVSGRLCFARGQRSAVLAVPLLDDEVAEEDETFFHRPSAGARL...................
consensus>50            a..g.dy....g...f...e..k...v.iiddd..e.de.f.v.l.........................
```

```
A.thaliana               ......................................................................
A.halleri                ......................................................................
A.lyrata                 ......................................................................
C.sativus                ......................................................................
C.clementina             ......................................................................
R.communis               ......................................................................
M.esculenta              ......................................................................
P.trichocarpa            ......................................................................
P.persica                ......................................................................
C.papaya                 ......................................................................
A.coerulea               ......................................................................
V.vinifera               ......................................................................
S.tuberosum              ......................................................................
S.lycopersicum           ......................................................................
M.guttatus_1             ......................................................................
M.guttatus_2             ......................................................................
G.max                    ......................................................................
M.truncatula             ......................................................................
E.grandis                ......................................................................
S.italica                ......................................................................
Z.mays                   ......................................................................
S.bicolor                ......................................................................
T.aestivum               ......................................................................
B.distachyon             ......................................................................
O.sativa_J1              ......................................................................
O.sativa_I1              ......................................................................
O.sativa_J2              ......................................................................
S.moellendorffii_1       ......................................................................
P.patens_1               ......................................................................
P.patens_2               ......................................................................
C.reinhardtii            ........REVLVAQERVRVTIVDDDDAGVIGFELPDYEVAF..............NEKRTFAEVTLVRR
C.intestinalis           ........DATLGDPSVSRVTIIDDDKPGDFVLETSQLYADM..............EKGEVSATV..LRQ
B.floridae               ........DAALGDPSVARVTIIDDDKPGNFVFEQPIYYGDS..............RTFKVTATV..LRQ
T.adhaerens_3            ........YSKLADPSVALVTIIDTNVPGEFQFEKAKYHVDK..............DSNQISLKV..LRK
N.vectensis_2            ........ITDLGEPNLTRITIIDDDMPGEFSFEQASYHANF..............VTGEATCTV..VRR
H.sapiens_NCX1.1         NHVS...TLACLGSPSTATVTIFDDDHAGIFTFEEPVTHV................SESIGIMEVKVLRT
P.troglodytes_NCX1.1     NHVS...TLACLGSPSTATVTIFDDDHAGIFTFEEPVTHV................SESIGIMEVKVLRT
C.familiaris_NCX1.1      NHVS...ALACLGSPSTATVTIFDDDHAGIFTFEEPVTHV................SESIGIMEVKVLRT
F.catus_NCX1.1           NHVS...TLACLGSPSTATVTIFDDDHAGIFTFEEPVTHV................SESIGIMEVKVLRT
C.porcellus_NCX1.1       NHIS...TLACLGSPSTATVTIFDDDHAGIFTFEEPVTHV................SESIGIMEVKVLRT
B.taurus_NCX1.1          SHVS...TLACLGSPSTATVTIFDDDHAGIFTFEEPVTHV................SESIGIMEVKVLRT
R.norvegicus_NCX1.1      NHVS...AIACLGSPNTATITIFDDDHAGIFTFEEPVTHV................SESIGIMEVKVLRT
M.musculus_NCX1.1        NHAS...SIACLGSPSTATITIFDDDHAGIFTFEEPVTHV................SESIGIMEVKVLRT
M.mulatta_NCX1.3         NHVS...TLACLGSPSTATVTIFDDDHAGIFTFEEPVTHV................SESIGIMEVKVLRT
O.cuniculus_NCX1.2       NHIS...TLACLGSPCTATVTIFDDDHAGIFTFEESVTHV................SESIGIMEVKVLRT
S.tropicalis_NCX1        NHVT...PLACLGATCTATVTIFDDDHAGIFTFEETVTHV................SESVGIMEVKVLRT
O.mossambicus_NCX1.1     NHVD...TLAGLGLPCTATVTIFDDDHAGIFTFEEPVMTV................SESVGVMEVKVIRT
O.mykiss_NCX1            NHLD...TLAGLGLPCSATVTIFDDDHAGIFTFEEPVMTI................SESIGMMEVKVLRT
D.rerio_NCX1a            NHVD...ALAGLGLPSTATVTIFDDDHAGIFLFEEPIVHI................SESIGMMEVKVVRT
H.sapiens_NCX3.3         IFNSLPLPRAVLASPCVATVTILDDDHAGIFTFECDTIHV................SESIGVMEVKVLRT
R.norvegicus_NCX3.3      ILNSLPLPRAVLASPCVATVTILDDDHAGIFTFECDTIHV................SESIGVMEVKVLRT
M.musculus_NCX3.1        ILNSLPLPRAVLASPCVATVTILDDDHAGIFTFECDTIHV................SESIGVMEVKVLRT
G.gallus_NCX3            ELGNSPYPKAILASPCVATVTILDDDHAGIFTFECDVIHV................SESIGVMEVKVLRT
D.rerio_NCX4a            IGSSHVPPKAALGNAHSATVTIYDDDHAGIFTFESNSTRV................SESVGIMQVKVHRT
T.nigroviridis_NCX4a     ......PPEAALGKAHTATVTIYDDDHAGIFTFESDSTKV................SESIGNMQVKVHRT
H.sapiens_NCX2.1         .......PKGRLVAPLLATVTILDDDHAGIFSFQDRLLHV................SECMGTVDVRVVRS
R.norvegicus_NCX2.1      .......PKGRLVAPLLATVTILDDDHAGIFSFQDRLLHV................SECMGTVDVRVVRS
M.musculus_NCX2.1        .......PKGRLVAPLLATVTILDDDHAGIFSFQDRLLHV................SECMGTVDVRVVRS
S.kowalevskii_3          .......PQARLVEPAFATVVILDDDHAGIFHFEEKLVKV................AETAGELEIKVVRS
T.adhaerens_2            ........KCVIESPSTATVVILDDDHPGVFSFSESKYSV................IETAGTLSLTVERK
L.opalescens_NCX         .......AKAQLANPFLATVMILDDDHPGIFQIDEKEMSV................TESSGEVEVRIIRT
A.mellifera_NCX          .........AMLVSPSLATVMILDDDHSGVFGFPERDMEL................VESVGQYPLRVMRY
D.melanogaster_NCX       .........VKLAVPMIATVMILDDDHAGIFAFTDSVFEI................TESVGRFELKVMRY
H.magnipapillata         .......TKTTIGKANTATIIILDDDYPGIFTFEHSNFEV................MESVGVLTLRVLRL
C.elegans_NCX1           .....LAA........TATVIIVDDDHAGSFGFLSEKFKC................TESCGSFVAEVIRS
C.briggsae_NCX1          .....PAA........TATVIIVDDDHAGCFGFASEKFKC................VESCGSFVAEVIRS
C.elegans_NCX2           .....GLPVAQLEMPNTATIMILDDDHAGVFGFEHDHFQV................VENCGHLSLQMKRH
C.briggsae_NCX2          .....GLPVAQLEMPNTATIMILDDDHAGVFGFDHDHFQV................VENCGHLSLQMKRH
T.spiralis               AAVGESVEFA...........ISDLRVGENEGFARIPISR................RASLSTDPRSVFVM
M.brevicollis_1          .......NYRLIPARELAKIIIVDDDKPGEIEFEQTRMEVE................EDCGCCEVVVRRR
Salpingoeca              .......QYLIVPGRNRARITIIDDDHPGVVSLELSKFSCL................ENVGTIFLSVLRQ
S.kowalevskii_1          ...........LGKKNTMQITIINDDEPGTFEFTMPSYLVK................ESIGVLQVPVSRN
S.kowalevskii_2          ...........LGKKHIMQITIINDEEPGTFEFTMPSYLVK................ESIGVLQVPVSRN
T.adhaerens_1            ........DAIIGEKNSTEITIINDDEPGEIAFNKASYLFK................ESCGTAKVEIVRK
D.pulex_1                .......KDVMLGRISIMEVTVLNDDDPGTITFEERGILVK................ESVGVAQVPVLRK
P.humanus_corporis       .........VQIGRISIMEITILDDDKPGVISFEKTGILVK................ESAECVVINVRRK
O.dioica                 .......PRCKLGDRSINQVTIINDDDPGTFQFKEPAFMCK................ESQGILKLPVCRT
A.pisum                  .......LSVKLGCISIMEVTIIDNDEPGIISFGKRGLLVK................ESTGFVEVPIVRT
P.marinus                ............GKLSVAEVTIIDDDEIGELVLEKEELVVEGDVSPSPWPVVDPSEARLWQEAQVTVLRT
A.anophagefferens_1      .......AF...GDASTCEVTIIDDDEPGELSFEPPKEGAEGGPVLTM........AETCGKARVKVGRY
E.huxleyi                ............GRRSTMAVTIIDDDGPGEVTFAVAETRVR................ESQRVAVLEVERK
consensus>50             ...................v.i.ddd..g...f........................e..g.....v.r.
```

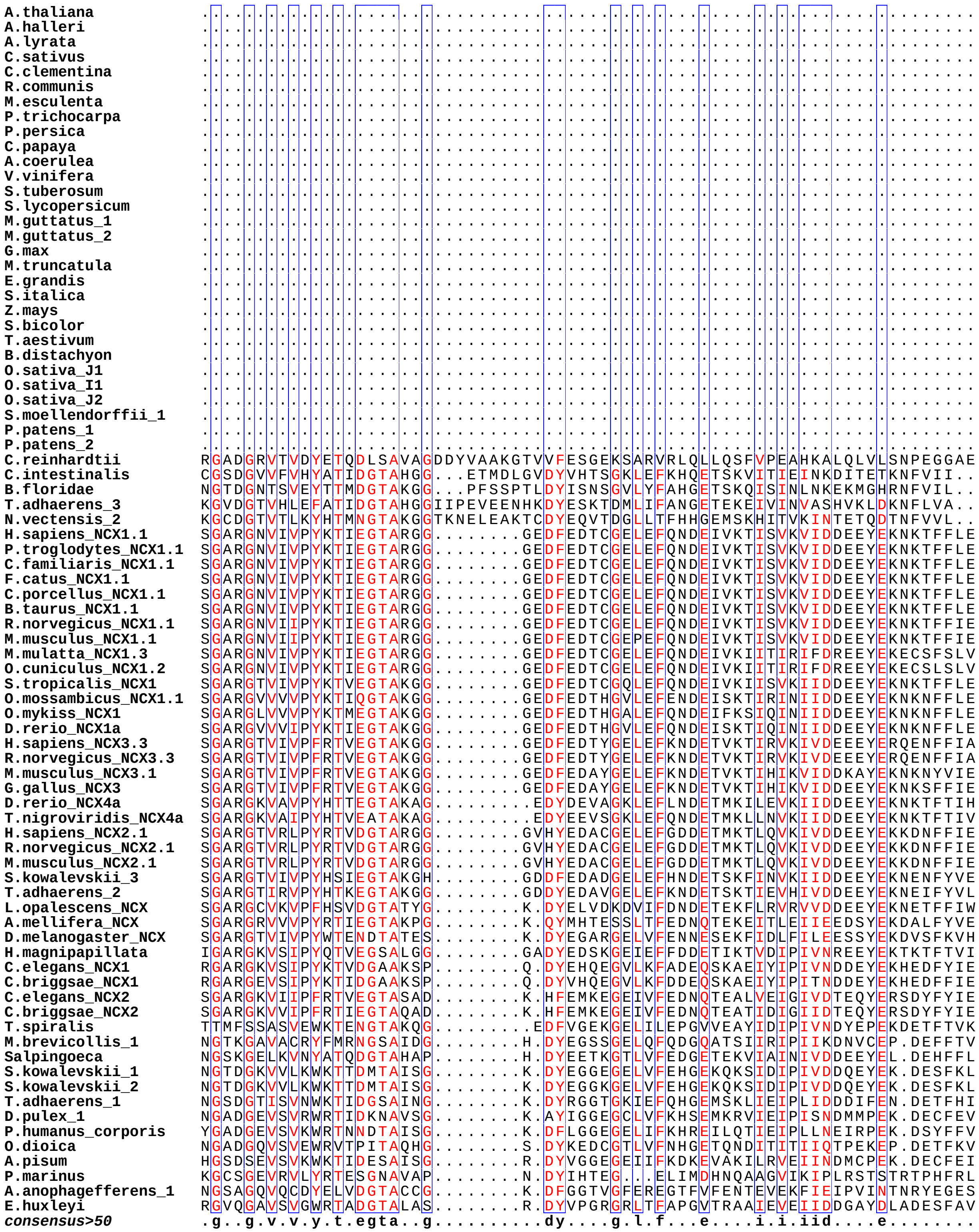
A.thaliana
A.halleri
A.lyrata
C.sativus
C.clementina
R.communis
M.esculenta
P.trichocarpa
P.persica
C.papaya
A.coerulea
V.vinifera
S.tuberosum
S.lycopersicum
M.guttatus_1
M.guttatus_2
G.max
M.truncatula
E.grandis
S.italica
Z.mays
S.bicolor
T.aestivum
B.distachyon
O.sativa_J1
O.sativa_I1
O.sativa_J2
S.moellendorffii_1
P.patens_1
P.patens_2
C.reinhardtii
C.intestinalis
B.floridae
T.adhaerens_3
N.vectensis_2
H.sapiens_NCX1.1
P.troglodytes_NCX1.1
C.familiaris_NCX1.1
F.catus_NCX1.1
C.porcellus_NCX1.1
B.taurus_NCX1.1
R.norvegicus_NCX1.1
M.musculus_NCX1.1
M.mulatta_NCX1.3
O.cuniculus_NCX1.2
S.tropicalis_NCX1
O.mossambicus_NCX1.1
O.mykiss_NCX1
D.rerio_NCX1a
H.sapiens_NCX3.3
R.norvegicus_NCX3.3
M.musculus_NCX3.1
G.gallus_NCX3
D.rerio_NCX4a
T.nigroviridis_NCX4a
H.sapiens_NCX2.1
R.norvegicus_NCX2.1
M.musculus_NCX2.1
S.kowalevskii_3
T.adhaerens_2
L.opalescens_NCX
A.mellifera_NCX
D.melanogaster_NCX
H.magnipapillata
C.elegans_NCX1
C.briggsae_NCX1
C.elegans_NCX2
C.briggsae_NCX2
T.spiralis
M.brevicollis_1
Salpingoeca
S.kowalevskii_1
S.kowalevskii_2
T.adhaerens_1
D.pulex_1
P.humanus_corporis
O.dioica
A.pisum
P.marinus
A.anophagefferens_1
E.huxleyi
consensus>50
.g..g.v.v.y.t.egta..g.........dy....g.l.f...e....i.i.iid....e........

```
A.thaliana             ......................................................................
A.halleri              ......................................................................
A.lyrata               ......................................................................
C.sativus              ......................................................................
C.clementina           ......................................................................
R.communis             ......................................................................
M.esculenta            ......................................................................
P.trichocarpa          ......................................................................
P.persica              ......................................................................
C.papaya               ......................................................................
A.coerulea             ......................................................................
V.vinifera             ......................................................................
S.tuberosum            ......................................................................
S.lycopersicum         ......................................................................
M.guttatus_1           ......................................................................
M.guttatus_2           ......................................................................
G.max                  ......................................................................
M.truncatula           ......................................................................
E.grandis              ......................................................................
S.italica              ......................................................................
Z.mays                 ......................................................................
S.bicolor              ......................................................................
T.aestivum             ......................................................................
B.distachyon           ......................................................................
O.sativa_J1            ......................................................................
O.sativa_I1            ......................................................................
O.sativa_J2            ......................................................................
S.moellendorffii_1     ......................................................................
P.patens_1             ......................................................................
P.patens_2             ......................................................................
C.reinhardtii          LG....................................................................
C.intestinalis         ......................................................................
B.floridae             ......................................................................
T.adhaerens_3          ......................................................................
N.vectensis_2          ......................................................................
H.sapiens_NCX1.1       IGEPRLVEMS......................................EKKALLLNELGGFTIT.GKYLF
P.troglodytes_NCX1.1   IGEPRLVEMS......................................EKKALLLNELGGFTIT.GKYLF
C.familiaris_NCX1.1    IGEPRLVEMS......................................EKKALLLNELGGFTIT.GKYLY
F.catus_NCX1.1         IGEPRLVEMS......................................EKKALLLNELGGFTIT.GKYLY
C.porcellus_NCX1.1     IGEPRLVEMS......................................EKKALLLNELGGFTIT.GKHLY
B.taurus_NCX1.1        IGEPRLVEMS......................................EKKALLLNELGGFTIT.GKYLY
R.norvegicus_NCX1.1    IGEPRLVEMS......................................EKKALLLNELGGFTLTEGKKMY
M.musculus_NCX1.1      IGEPRLVEMS......................................EKKALLLNELGGFTLT.GKEMY
M.mulatta_NCX1.3       LEEPKWIRRG......................................MK........GGFTIT......
O.cuniculus_NCX1.2     LEEPKWIRRG......................................MK.ALLLNELGGFTIT......
S.tropicalis_NCX1      VGEPRLVEMS......................................EKKALLLNELGDFTIT.GKILY
O.mossambicus_NCX1.1   IGEPRLLEMS......................................ERKAVLLQEVGGFVKT......
O.mykiss_NCX1          MGEPQLLEMS......................................ERKAVLLQEIGGFVKT......
D.rerio_NCX1a          IGEPQLVEMS......................................ERKAMLLHECGGFVKTD.KQLY
H.sapiens_NCX3.3       LGEPKWMERG......................................IS.ALLLSPDVTDRK.......
R.norvegicus_NCX3.3    LGEPKWMERG......................................IS.ALLLSPEVTDRK.......
M.musculus_NCX3.1      MMGPRMVDMS......................................VQKALLLSPEVTDRK.......
G.gallus_NCX3          LMSPRMVDMS......................................LQKALILLAE...RK.......
D.rerio_NCX4a          LGEPVLLEIG......................................QKHG..................
T.nigroviridis_NCX4a   LEEPILLEVG......................................QKHG..................
H.sapiens_NCX2.1       LGQPQWLKRG......................................ISALLLNQGDGDRK........
R.norvegicus_NCX2.1    LGQPQWLKRG......................................ISALLLNQGDGDRK........
M.musculus_NCX2.1      LGQPQWLKRG......................................ISALLLNQGNGDKK........
S.kowalevskii_3        LGEPRLVKRG......................................QGSDDEDSAYGDK.........
T.adhaerens_2          LGEPYFK...............................................................
L.opalescens_NCX       LDEPYLVKKP......................................TGSSSGSVVEDDDP........
A.mellifera_NCX        LGEPQLQGGD......................................GLAAEMKQPEER..........
D.melanogaster_NCX     IGEPRLAPDD......................................ELAAKIKEVEKKPV........
H.magnipapillata       LGEPKIVGKL......................................NSTITNLSSI............
C.elegans_NCX1         LGEPIWHRELADDEEGIEGKPILG..............................................
C.briggsae_NCX1        LGDPIWHKEIKDDEEGAEGKPILG..............................................
C.elegans_NCX2         LSPPIWAKKMNDLSRIQERFQRRMERKRGSSVASESKDSNTENALAPAEKSTRAASVDLLQPSSDPRRSS
C.briggsae_NCX2        LSPPIWAKKMNDLSRIQERFQRRMERKRGSSVASESKDSNTETALAPTDKSTRAASVDLLQPCSDPRRSS
T.spiralis             LLP...................................................................
M.brevicollis_1        HLEEVS................................................................
Salpingoeca            TLEESE................................................................
S.kowalevskii_1        ELLEVS................................................................
S.kowalevskii_2        ELLEVS................................................................
T.adhaerens_1          ELFDPN................................................................
D.pulex_1              ELSDPT................................................................
P.humanus_corporis     ELFSPS................................................................
O.dioica               SIVKVS................................................................
A.pisum                KLFETT................................................................
P.marinus              ILSKPS................................................................
A.anophagefferens_1    EFTIVL................................................................
E.huxleyi              VLDEPG................................................................
consensus>50           ......................................................................
```

```
                                                                             260           270       280
A.thaliana              ........................................VHDVYSDAAQDAVESGSRNIVDIFSIHSAN
A.halleri               ........................................VHDVYSDAAQEAVESGSRNIVDIFSIHSAN
A.lyrata                ........................................VHDVYSDAAQEAVESGSRNIVDIFSIHSAN
C.sativus               ........................................EEFQ.......AHENEQRSIVDIFSIHDSD
C.clementina            ........................................DECSEILQLGEDENRGVVDIFSIHSPNGAV
R.communis              ........................................EEYSEIVQVSEEDSRNIVDIFSIHSPVG..
M.esculenta             ........................................EEYSEILQVDKDESRNVVDIFSIHSNVE..
P.trichocarpa           ........................................GE..........ENRNVVDIFSIHSNNG..
P.persica               ........................................NNYSEILEVGEDENRNIVDIFSFHSGSGLG
C.papaya                ........................................F...............VVDIFSIHSAP...
A.coerulea              ........................................SEILQLSE....ED.NIVDIFSIHSEKETG
V.vinifera              ........................................DECSELFQ....ENGNIVDIFSIHSGD..G
S.tuberosum             ........................................EPHSEVSQVGEEENSGIVDIFSIHSGEGTG
S.lycopersicum          ........................................EPHSEVSQVGEEENTGIVDIFSIHSGEGTG
M.guttatus_1            ........................................EIPEDDHESGR.....IVDIFSVHSD....
M.guttatus_2            ........................................KTRDNYPEVDEDRNSIIVDIFSIHSANTGF
G.max                   ........................................VDFSDIKHVNE.ENGDTVDIFSIHSENPTD
M.truncatula            ........................................VECSEINHFNE.ENGDTVDIFSIHSENPRD
E.grandis               ........................................GPMYHNVPA.....................
S.italica               ........................................ET.SEILPGSSDKD..IADIFSGHSYHNA.
Z.mays                  ........................................GI.DETLPMGADGNDGIVDIFSAHSYYDAA
S.bicolor               ........................................DT.NETLPISANRNDGIVDVFSAHSYHIA.
T.aestivum              ........................................EN.SDILPSE...NDDVVDIFSIHSYSNAG
B.distachyon            ........................................ES.SEILPRE...NEDIVDIFSMHSYNNTG
O.sativa_J1             ........................................GI.GDILPGQ...NEDIVDIFSAHSYSNEG
O.sativa_I1             ........................................GI.GDILPGQ...NEDIVDIFSAHSYSNEG
O.sativa_J2             ........................................GI.GDILPGQ...NEDIVDIFSARSYSNEG
S.moellendorffii_1      .............................................VSRSSKFAAMRELVATRPHNFRLFF
P.patens_1              ...........................EWELVDLQHQPKQEIRNRVTCDDHPIKPIEWQQVAVAVDKNED
P.patens_2              ...........................DLERVDLQYLPMKEIRNGVTFDDHRISPFERQQVIGANDISEQ
C.reinhardtii           .............................KRSACKVTLVRRQFTLMPGAATAAGQKEGLELGNGSIKLVG
C.intestinalis          ............................................LRNPSQGSHLGEHSAAVVN..IN...
B.floridae              ............................................LRNPSLGSKIGEPGAAVGF..LCKDE
T.adhaerens_3           ............................................LRNASMGARIGEIGATIVSFDIERDA
N.vectensis_2           ............................................LHDPSTGARIGNRTATVVHISSGI..
H.sapiens_NCX1.1        GQPVFRKVHAREHPILSTVITIADEYDDKQPLTSKEEEERRIAEMGRPILGEHTKLEVIIEESYEFKSTV
P.troglodytes_NCX1.1    GQPVFRKVHAREHPILSTVITIADEYDDKQPLTSKEEEERRIAEMGRPILGEHTKLEVIIEESYEFKSTV
C.familiaris_NCX1.1     GQPVFRKVHAREHPIPSTVITIAEEYDDKQPLTSKEEEERRIAEMGRPILGEHTKLEVIIEESYEFKSTV
F.catus_NCX1.1          GQPVFRKVHAREHPIPSTVITIAEECDAKQPLTSKEEEERRIAEMGRPILGEHTKLEVIIEESYEFKSTV
C.porcellus_NCX1.1      GQPVLRKVHARDHPIPSTVITIADEYDDKQPLTSKEEEERRIAELGRPILGEHTKLEVIIEESYEFKSTV
B.taurus_NCX1.1         GQPVFRKVHAREHPLPSTIITIADEYDDKQPLTSKEEEERRIAEMGRPILGEHTRLEVIIEESYEFKSTV
R.norvegicus_NCX1.1     GQPVFRKVHARDHPIPSTVISISEEYDDKQPLTSKEEEERRIAEMGRPILGEHTKLEVIIEESYEFKSTV
M.musculus_NCX1.1       GQPIFRKVHARDHPIPSTVITISEEYDDKQPLTSKEEEERRIAEMGRPILGEHTKLEVIIQESYEFKSTV
M.mulatta_NCX1.3        .......................DEYDDKQPLTSKEEEERRIAEMGRPILGEHTKLEVIIEESYEFKSTV
O.cuniculus_NCX1.2      .......................EEYDDKQPLTSKEEEERRIAEMGRPILGEHTKLEVIIEESYEFKSTV
S.tropicalis_NCX1       GKPVLRKVQVRDHPIPSTVIILTEENEEKQPLTSKEEEERRIAEMGRPVLGEHTRLEIIIEESYEFKSTV
O.mossambicus_NCX1.1    GRDIYRKVQGREHPVPSNIISIA.EDGAEEPLTKKEEEERRIAEMGRPMLGEHVKLEVIIEESYEFKSTV
O.mykiss_NCX1           GRDVYRKVQGRDNPVPATIISLA.EEGDEEALSKKEEEERRIAEMGRPTLGEHVKLEVVIEESYEFKNTV
D.rerio_NCX1a           GRDVYRKVQGRDKPIPSTIISIS.EDGEEETLTKKEKDERRIAEMGRPTLGEHVKLEVIIEESYEFKSTV
H.sapiens_NCX3.3        ...............................LTMEEEEAKRIAEMGKPVLGEHPKLEVIIEESYEFKTTV
R.norvegicus_NCX3.3     ...............................LTMEEEEAKRIAEMGKPVLGEHPKLEVIIEESYEFKSTV
M.musculus_NCX3.1       ...............................LTVEEEEAKRIAEMGKPVLGEHPKLEVIIEESYEFKSTV
G.gallus_NCX3           ...............................LTVEEEEAKRIAEMGKPILGEHPKLEVIIEESYEFKSTV
D.rerio_NCX4a           ..........................DSNDNKPAVGAEEEEVAKMGCPSLGEHTKLEVVIEESYEFKNTV
T.nigroviridis_NCX4a    ..........................DTNDNKTSVGPED..VSKMGCPCLGEHTKLEVVIEESYEFKSTV
H.sapiens_NCX2.1        ...............................LTAEEEEARRIAEMGKPVLGENCRLEVIIEESYDFKNTV
R.norvegicus_NCX2.1     ...............................LTAEEEEAQRIAEMGKPVLGENCRLEVIIEESYDFKNTV
M.musculus_NCX2.1       ...............................ITAEQEEAQRIAEMGKPVLGENNRLEVIIEESYDFKNTV
S.kowalevskii_3         ...........................ENSGSNHMTEDEKRVAEMGKPRLGDLIKCEVKILESTEFKNTV
T.adhaerens_2           .......................DKKRIEKERNHVAKTTGNQPIDVNPSLGPYKEAEVTIIESHEFKNTV
L.opalescens_NCX        .................................VL.......AELGKPRRGENIKITVHIIESTEFKSVV
A.mellifera_NCX         ....................................TAEEKMALLGKPKLGEVFRAQIRIKESKEFKNTV
D.melanogaster_NCX      .................................QDLTELDRILLLSKPRNGELTTAYVRIRESQEFKATV
H.magnipapillata        ..................................KDDDLRKVLEVGKPTLGDHKACEIKIVESKEFRKTI
C.elegans_NCX1          ....................................................FSRCKVVITEDREFKNFM
C.briggsae_NCX1         ....................................................FARCKVVITEDRDFKNFV
C.elegans_NCX2          QTNSPHLTSRFRNRLGSWIAGMKGGNGDDEVTTSLTPSQLEIAEMGKPRLGEFTKCQITIRESKEFQGIV
C.briggsae_NCX2         QSNSPHLTSRFRNRLGSWIAGMKGGNGDDEVTTALTPSQLEIAEMGKPRLGEFTKCQITIRESKEFQGIV
T.spiralis              .............................................GKASLGACQSIVVTIVNDDNISRYY
M.brevicollis_1         ..............................................GSTLGKVTVCRVVILNDDKAQRLL
Salpingoeca             ..............................................DVTFGEVTKCKITILNDDEVTTLA
S.kowalevskii_1         .............................................DGAKLGKTRTSVITIVNDDEYNSLL
S.kowalevskii_2         .............................................DGAKLGKTRTSVITIVNDDELNNLL
T.adhaerens_1           .............................................GGAKLGSRTRTALTIVNDDVYNSLL
D.pulex_1               .............................................GGAVLGSITKLAVTITNDEEFNSVL
P.humanus_corporis      .............................................NDASLGDIKVICVTITNDEDFANII
O.dioica                .............................................TGAAMGHTRETVVTVIGDEEYKNLV
A.pisum                 .............................................GGARIGNVNRIAITISSDDDFDSVV
P.marinus               .............................................GGAKFCS............KTDGGH
A.anophagefferens_1     .............................................KNFKCAV............ERAKFG
E.huxleyi               .............................................GGASLGEIGRAVVVIESDDERRALV
consensus>50            ...............................................p..ge.....v.i.e..ef....
```

```
                              290       300                 310       320       330       340
                                .         .                   .         .         .         .
A.thaliana              NDTGITYHTVADTPPDSATKK....GKAKNSTVFDIWKHQFVDAITLETSESKKVDSIYLRIAKSFWHLL
A.halleri               NDTGITYHTVADTPPDSATKK....GKAKNSSVFDIWKHQFVDAITLETSESKKVDSIYLRIANSFWQLL
A.lyrata                NNTGITYHTVADTPPDSATKK....GKAKNSTVFGIWKHQFVDAITLETSESKKVDSIYLRIAKSFWQLL
C.sativus               GKVYHEVPGHDIAESSNSNIPEEMDGKADHPHVLKIWKQQFVDALSLETSESKQRNNIYLRSARLCWQLI
C.clementina            TSPLYQKVPGSEDVAEISKESFREEINSELPHVHALWKQQFVDSIALESPESRKMNNIYLRLARIFWQSL
R.communis              TDPVYQKVPETDEAAESSNNYSLSEKDL...DVVALWKQQFVDAIMLERPESRKLINSHLRLARISWQLF
M.esculenta             TDQVYHKVPETDDAVEFSDKNFHSEQDF...NVAALWKRQFLDAITLESLESRNMFSIHIRLARTFWEIL
P.trichocarpa           T..VYQKVPVTDDAAESSNKHFHQEKDL...HVLSLWKEQFVDALTLESLETRKLNSIHLRVARISWQLI
P.persica               ..PVYQNVPGTDETPEYSNKDSPEKMSLEDYHVFAIWGQQFVDAVKLKSTESRQLNNLYLRLARISWQLL
C.papaya                .STEYQKVPADENIPEPSGKSSRKEILLEDPNVLTLWKQQFVDIFKVEKSDSRKLDNIYLQLARIVWQSL
A.coerulea              PLYQPVSNS...DVAESSNMNFHNKSVLQDFNVFTVWKHQFMDAVMLENPESRKLDNTYLRLARGTWQVI
V.vinifera              SVYHRISGS...EVAEPSNEHFQKNIILEDSHLLSLWKQQFVDAFTLESSESRKLDNIYLWVARIFWQLL
S.tuberosum             HFYRNLAG....EDVTESSTPNNGNIIPEESDILSIWKHQFVDALMLESTESRKLNNIYLRVARIFWQLL
S.lycopersicum          HFYQNLAG....EDVTESSTPNNCYIIPEESDILSIWKHQFVDALMLESTESRKLNNTYLRVARIFWQLL
M.guttatus_1            HVYQNLSG....SDSGEPSGQSHEDIHVKDNLI.SIWKQQFVDAFVLEGQESRKLNNTYLRAAKVLWELL
M.guttatus_2            VYENVADT....DIHESSSEQCRVDTIPHKDDLLSVWKMQFVDALTLESPESKKLNSKSLRVAKALWKLV
G.max                   PLYARVPSI....NDEAEILDKAKETTLVDTHLLTIWRQQFLDALRLLRPESKKIKNACLRLACIFWQLL
M.truncatula            MSYVRVPQI....DD.AENSDKVIETRLEDTCLLTIWKQQFVDALTVESQESKKMNNIYIRTARIFWQLL
E.grandis               .....................SDLAESSNELHVLALWKEQFVDALMLESAETKKLYSIYVRALRMLWQSL
S.italica               EY....HKVPENDMECSSTMNNFVKNTREDTSWRSLWRQQFVDAFMLESPESRKMASVCLRLIRIFWNLL
Z.mays                  EY....SQLAEEDMEGSSTMDQVVKNTQDNMSWLSIWWQQFVDASMLESPESMKMDSVCLRYTRIFWNLI
S.bicolor               EY....SRVPEKDMEGSSTMDQVVKNTQEDISWLSIWWQQFVDASMLESPESRKMHSICLRFTTIFWNLI
T.aestivum              .Y....HHVPEKDIEESSKTTLVVKNTQEDIYWLSVWRQQFVDAVMLESPELKKMDPVCLRFIIICWNSI
B.distachyon            .Y....HHVPEKDIEESSKTTLVVKNTQEDTCWLSIWRQQFVDALMLESPEQRKMDSICLRFIIIFWNSI
O.sativa_J1             .Y....HHVSEEDVEESS.TGLTLKNKWEDTHWFSIWWQQFVDAATLESSVSRKMDSTCLRVIGISWNLI
O.sativa_I1             .Y....HHVSEEDVEESS.TGLTLKNKWEDTHWFSIWWQQFVDAATLESSVSRKMDSTCLRVIGISWNLI
O.sativa_J2             .Y....HHVSEKDVEESP.TGLTLKNKWEDTHWFSIWWQQFVDAATLESSVSRKMDSTCLSVIGISWNLI
S.moellendorffii_1      FSSRAEDVDTSSRDNFAFLREGAETEHFSWKALGTAWKNQFYDAMTVSFFEGRGQRKRTPSTLQLLHPI
P.patens_1              ENDPG....SNQEFLKQRLAYDIYEQTSMWTYIYSTWKKQFLDVIVFQGQVDDSGKNLALTAVEFIGYLI
P.patens_2              LTCGSKNSESNQELVKLRSTNEIDEHTSGRTNACSLWKMQFLDAIFIEKHVDESGKDHSPIAVDCVGHLI
C.reinhardtii           GAEGGAGSDKGEGEGEEFNLWSAWREQIVSV................FSPDEPDEGEVVSWAGLMLQYIN
C.intestinalis          LEACGINSMSRVLIDENDTTWVGQIRNAMIV................GGEMDDYGNESPPSNTDFLMHAI
B.floridae              LAERVANVVPMGDTEEEDTSWGGQFRSAMCL................ESEEDEDGKKIPPSVGQLIMHFV
T.adhaerens_3           LGDRVAEFAALNGVEGEDVSWAEQFTNALTV................GSDIDEFGEESPPSNLDYILHFI
N.vectensis_2           ...........................AMII................QGDEDKDGNELPLNSIDFIMHFL
H.sapiens_NCX1.1        DKLIKKTNLALV...VGTNSWREQFIEAITVSAG.E.....DDDDDECG......EEKLPSCFDYVMHFL
P.troglodytes_NCX1.1    DKLIKKTNLALV...VGTNSWREQFIEAITVSAG.E.....DDDDDECG......EEKLPSCFDYVMHFL
C.familiaris_NCX1.1     DKLIKKTNLALV...VGTNSWREQFIEAITVSAG.E.....DDDDDECG......EEKLPSCFDYVMHFL
F.catus_NCX1.1          DKLIKKTNLALV...VGTNSWREQFIEAITVSAG.E.....DDDDDECG......EEKLPSCFDYVMHFL
C.porcellus_NCX1.1      DKLIKKTNLALV...VGTNSWREQFIEAITVSAG.E.....DDDDDECG......EEKLPSCFDYVMHFL
B.taurus_NCX1.1         DKLIKKTNLALV...VGTNSWREQFIEAITVSAG.E.....DDDDDECG......EEKLPSCFDYVMHFL
R.norvegicus_NCX1.1     DKLIKKTNLALV...VGTNSWREQFIEAITVSAG.E.....DDDDDECG......EEKLPSCFDYVMHFL
M.musculus_NCX1.1       DKLIKKTNLALV...VGTNSWREQFIEAITVSAG.E.....DDDDDECG......EEKLPSCFDYVMHFL
M.mulatta_NCX1.3        DKLIKKTNLALV...VGTNSWREQFIEAITVSAG.E.....DDDDDECG......EEKLPSCFDYVMHFL
O.cuniculus_NCX1.2      DKLIKKTNLALV...VGTNSWREQFIEAITVSAG.E.....DDDDDECG......EEKLPSCFDYVMHFL
S.tropicalis_NCX1       DKLIKKTNLALV...VGTNSWREQFIEAITVSAG.E.....DDDDDECG......EEKLPSCFDYVMHFL
O.mossambicus_NCX1.1    DKLIKKTNLALL...IGTNSWREQFVEAITVSSG.......DDD..ECR......EEKLPSCFDYVMHFL
O.mykiss_NCX1           DKLIKKTNLALL...IGTNSWRQQFMEAITVSSG.......DDDEDECG......EEKLPSCFDYVMHFL
D.rerio_NCX1a           DKLIKKTNLALL...VGTNSWRDQFVEAITVNSG.......DDDDEECG......QEKMPSCFDYVMHFL
H.sapiens_NCX3.3        DKLIKKTNLALV...VGTHSWRDQFMEAITVSAA.G.....DEDEDESG......EERLPSCFDYVMHFL
R.norvegicus_NCX3.3     DKLIKKTNLALV...VGTHSWRDQFMEAITVSAA.G.....DEEEDESG......EERLPSCFDYVMHFL
M.musculus_NCX3.1       DKLIKKTNLALV...VGTHSWRDQFMEAITVSAG.G.....DEDEDESG......EERLPSCFDYVMHFL
G.gallus_NCX3           DKLIKKTNLALV...VGTHSWRDQFLEAITVSAA.G.....DEDEDESG......EERLPSCFDYVMHFL
D.rerio_NCX4a           DKLIKKTNLALV...VGSSSWREQFVSAVTVSAG.......DDDEEESG......EERLPSCFDYIMHFL
T.nigroviridis_NCX4a    DKLIKKTNLALV...VGSSSWREQFVSAVTVSAG.......DDDEEESG......EERLPSCFDYIMHFL
H.sapiens_NCX2.1        DKLIKKTNLALV...IGTHSWREQFLEAITVSAGDE.....EEEEDGSR......EERLPSCFDYVMHFL
R.norvegicus_NCX2.1     DKLIKKTNLALV...IGTHSWREQFLEAVTVSAGDE.....EEDEDGSR......EERLPSCFDYVMHFL
M.musculus_NCX2.1       DKLIKKTNLALV...IGTHSWREQFIEAVTVSAGDE.....EEDEDGPR......EERLPSCFDYVMHFL
S.kowalevskii_3         DKLIKKANLSLV...VGTSSWREQFVEAVTVSSG.........DEDEDG......EEKLPSCSDYVMHFL
T.adhaerens_2           DKLLKKANLAMA...IGTSTWREQFRAALTVNGGDN.....DDDDD.........EETKPTCTDYIMHYL
L.opalescens_NCX        DKLLKKANLSLV...VGTSSWREQFIEAITVN.AEG.....DDDDEEGE......EEKLPSCMDYIMHFV
A.mellifera_NCX         DKLVQRANASIL...LGTSSWKEQFTEALTVSGGDE.....D..DEGGG......EPAAPSTLDYLMHGV
D.melanogaster_NCX      DKLVAKANVSAV...LGTSSWKEQFKDALTVIPADE.....SEFDNDDE......EEEVPSCFSYVSHFV
H.magnipapillata        DQMLSAKNKSIL....GSSSWAQQFKDAFQVEYGG.........DEEEG......DDVEPTYGDYVMHYL
C.elegans_NCX1          DRALVTANTSIM...VGTSSWKQQFTEAWTLEP....................EEEDGEVTTMEKVMHYI
C.briggsae_NCX1         DKVLVTANTSIM...VGTSSWKQQFTEALTLEP....................EEEGGDVSLQEKIMHYV
C.elegans_NCX2          DRMIKNANTRIM...LGTHSWREQFMEALVVSAGDD.....DDDEGEDGEDGEEKEPEEPGCMDYVMHVL
C.briggsae_NCX2         DRMIKNANTRIM...LGTHSWREQFMEALVVSAGDD.....DDDDEE.GDDGEEKEPEEPGCMDYFMHVL
T.spiralis              AKFEEKVRQSLLTCKLESSTWKEQFLKAVSVNGG.....................ETSDASMVDCFAHII
M.brevicollis_1         EKVSRRMKINMDRFRVGSSDWKQQFRDAV.....................AWPE...REAGVLGIVMHLI
Salpingoeca             DKVTALLNLNLDKFRIGGTDWKSQFVDAL.....................AWPE...EGSGTFAIVMHLI
S.kowalevskii_1         DRVVSITNVNLDRLRIGNASWADQFKEAM.....................NVNGGDIESATWVDYVMHFL
S.kowalevskii_2         DRVVSITNVNLDRLRIGNASWADQFKEAM.....................NVNGGDIESATWVDYVMHFL
T.adhaerens_1           DKVVALTRINVHRLKLGSQSWGEQFTNAM.....................TVNGGDVENATGMDYFLHFL
D.pulex_1               NRLADMTTANVDSMEVHHSTWTSQIKDAM.....................NVNGGDIENATTSDYVMHFL
P.humanus_corporis      DKMLAMTNLKLKTLSIYRKTWASQIKDAM.....................NVNGGDVKNATATDYVLHFI
O.dioica                KRVAAQTHVALSKMEIGGDSWYEQFVEAM.....................NVNGGDIENATGFDYIMHLL
A.pisum                 DRLMLLTNINIHALELHRQTWAEQIKTAM.....................TVNGGDLTNATTLTYVLHFF
P.marinus               DSLICTIWIKPKSGISQGRTTVTDLLNVHWGKMSLGATNWREQFNEALWVNGSREEQSEAGVGDWVMHVI
A.anophagefferens_1     KHTELKVVIVADTETKDMIDNVQKYLESNDASYNVGSTWGQQFSDALKVGGGDADDDFKASAGDWVMHII
E.huxleyi               KQVASLMSLNVDAMQLAGASWREQLAEAVTFEPPRQATTARALDGPLLGGASPPAALSLSCPFAFAGFAL
consensus>50            d......n............w.eqf.ea..............d............ee......dyvmh.l
```

```
                        350       360       370       380       390       400       410
A.thaliana              LAPWKLLFAFVPP..CNIAHGWIAFICSLLFISGVAFVVTRFTDLISCVTGINPYVIAFTALASGTSWPD
A.halleri               LAPWKLLFAFVPP..CNIAHGWIAFIFSLLFISGVAFVVTRFTDLISCVTGINPYVIAFTALASGTSWPD
A.lyrata                LAPWKLLFAFVPP..CNIAHGWIAFICSLLFISGVAFVVTRLTDLISCVTGINPYVIAFTALAGGTSWPD
C.sativus               VAPWRLLFAFVPP..YHIAHGWVAFICSLMFISGIAYVLTKFTDLISCVSGINPYVIAFTALASGTSWPD
C.clementina            LVPWRVLFAFVPP..YHIAHGWIAFICSLIFISGIAYIVTKLTDIISCVTGINAYVIAFTALASGTSWPD
R.communis              LVPWRLLFAFVPP..YHIAHGWIAFICSLLFISGIAYIVTQLTDLISCVTGINAYVIAFTALAAGTSWPD
M.esculenta             LAPWRLLFAFVPP..YHIAHGWVAFICSLIFISGIAYIVTKITDLISCVTGINAYVIAFTALASGSSWPD
P.trichocarpa           LAPWRLLFAFVPP..YHFAHGWIAFIFSLLFISGIAYIVTKLTDVISCVTGINAYVIAFTALASGTSWPD
P.persica               LVPWRLLFAFVPP..YHIAHGWIAFICSLVFISAIAYIVTQLTDLISCVTGINPYVIAFTALASGTSWPD
C.papaya                LAPWRFMFAFVPP..YHISHGWIAFVCSLLFISGISYIVTKLTDLVSCVTGINPYVIAFTALACGTSWPD
A.coerulea              LAPWRVLFAFVPP..CHIAHGWIAFICSLIFISGIAYIVTKLTDMISCVTGINGYVIAFTALASGTSWPD
V.vinifera              LLPWRFLFAFVPP..PHIAHGWFAFICSLIFISGIAYIVTKLTDLISCTSGINSYVIAFTALAAGTSWPD
S.tuberosum             LLPWKLLFAFVPP..YQIAHGWIAFICSLIFISGIAYVVTKITDLISCVTGINPYVIAFTALASGTSWPD
S.lycopersicum          LLPWKLLFAFVPP..YQIAHGWIAFICSLIFISGIAYVVTKITDLISCVTGINPYVIAFTALASGTSWPD
M.guttatus_1            LAPWRILFAFVPP..CQIAHGWISFISSLIFISGIAYVVTKLTDIISCVTGINAYVIAFTALAAGTSWPD
M.guttatus_2            LAPWRLLFAFVPP..YQIANGWIAFIFSLVFISGIAYIVTKITDLISCVTGINAYIIALTALAAGTSWPD
G.max                   ILPWRFLFAFVPP..CQIAHGWISFICSLLFISGIAYVVTKITDVISCVTGINAYVIAFTALASGTSWPD
M.truncatula            LLPWRFMFAFVPP..CHIAHGWISFICSLLFISGIAYIVTKITDLISCVTGINAYVIAFTALASGTSWPD
E.grandis               LAPWRLMFALVPP..CQIAHGWFAFICSLTFISGIAYVVTKLTDLISCVTGINAYVIAFTALATGTSWPD
S.italica               IAPWKLFFAFVPP..YHIAHGWVAFICSLIFISGIAYGVTKLTDQISCVTGVSPYVIAFTALAAGTSWPD
Z.mays                  IAPWKFLFAFVPP..YHIAHGWIAFICSLIFISGIAYGVTKLTDQISCVTGVSPYVIAFTALAAGTSWPD
S.bicolor               IAPWKFLFAFVPP..YNIAHGWIAFICSLIFISGIAYGVTKLTDQISCVTGVSPYVIAFTALAAGTSWPD
T.aestivum              IAPWKLLFAFVPP..YQIAHGWIAFIFSLIFISGIAYGVTNITDQISCVTGLNPYVIAFTALAAGTSWPD
B.distachyon            IAPWKLLFAFVPP..YQIAHGWIAFICSLIFISGIAYGVTKITDQISCVTGVNLYVIAFTALAAGTSWPD
O.sativa_J1             IAPWKMLFAFVPP..YEIAHGWIAFICSLIFISGIAYGVTKITDQISCVTGVSPYVIAFTALAAGTSWPD
O.sativa_I1             IAPWKMLFAFVPP..YEIAHGWIAFICSLIFISGIAYGVTKITDQISCVTGVSPYVIAFTALAAGTSWPD
O.sativa_J2             IAPWKMLFAFIPP..YEIAHGWIAFICSLIFISGIAYGVTKITDQISCVTGVSPYVIAFTALAAGTSWPD
S.moellendorffii_1      VSYWKVSFAFIPP..VQLLHGWLAFLGSICFITWISYVVVALSNRINCVTGISSYVLALTVLAAGTSLPN
P.patens_1              TLPWRFIFAFLPP..PSLLHGWAAFLCALAHITVIACFLIKLTNLFGCVTGISKYTLALTVLAAGTSLPD
P.patens_2              ILPWRFLFAFLPP..PMLLNGWPAFMCALAFITVISCFLIKLANSFGCVTGVSDYVLALTILAVGTSWPD
C.reinhardtii           ITWKLVLFILVPPAEWK..GGYPCFFAALGSIVGIVYLVNEAGSLFGCIIGLKEVMVGVSIVAVGTSLPD
C.intestinalis          SMPWKIVFALAPTRHAY..GGFPAFIISLLMIGCLTAIIEQLGHLLGCVATLKTQVTGITIIALGTSLPD
B.floridae              TFFWKVLFAFIPPRSML..GGWPAFVMSLGFITMLTAFIETLGHLLGCVFGVRTSVTGITIIALGTSVPD
T.adhaerens_3           AFLWKIIAATIPPRTVL..GGWPAFVLSLVYIGFLTAIIEQFGNLLGCVILLKPSVTGITIVALGTSLPD
N.vectensis_2           TFFWKVLFAFIPPRPLL..GGWPAFVVSLLFIAALTAVIEQLGKLLGCVVDLRNSVTGITIIAIGTSLPD
H.sapiens_NCX1.1        TVFWKVLFAFVPPTEYW..NGWACFIVSILMIGLLTAFIGDLASHFGCTIGLKDSVTAVVFVALGTSVPD
P.troglodytes_NCX1.1    TVFWKVLFAFVPPTEYW..NGWACFIVSILMIGLLTAFIGDLASHFGCTIGLKDSVTAVVFVALGTSVPD
C.familiaris_NCX1.1     TVFWKVLFAFVPPTEYW..NGWACFIVSILMIGILTAFIGDLASHFGCTIGLKDSVTAVVFVALGTSVPD
F.catus_NCX1.1          TVFWKVLFAFVPPTEYW..NGWACFIVSILMIGILTAFIGDLASHFGCTIGLKDSVTAVVFVALGTSVPD
C.porcellus_NCX1.1      TVFWKVLFAFVPPTEYW..NGWACFIVSILMIGLLTAFIGDLASHFGCTIGLKDSVTAVVFVALGTSVPD
B.taurus_NCX1.1         TVFWKVLFAFVPPTEYW..NGWACFIVSILMIGLLTAFIGDLASHFACTIALKDSVTAVVFVALGTSVPD
R.norvegicus_NCX1.1     TVFWKVLFAFVPPTEYW..NGWACFIVSILMIGLLTAFIGDLASHFGCTIGLKDSVTAVVFVALGTSVPD
M.musculus_NCX1.1       TVFWKVLFAFVPPTEYW..NGWACFIVSILMIGLLTAFIGDLASHFGCTIGLKDSVTAVVFVALGTSVPD
M.mulatta_NCX1.3        TVFWKVLFAFVPPTEYW..NGWACFIVSILMIGLLTAFIGDLASHFGCTIGLKDSVTAVVFVALGTSVPD
O.cuniculus_NCX1.2      TVFWKVLFAFVPPTEYW..NGWACFIVSILMIGLLTAFIGDLASHFGCTIGLKDSVTAVVFVALGTSVPD
S.tropicalis_NCX1       TVFWKVLFAFVPPTEYW..NGWACFIVSISMIGLLTAFIGDLASHFGCTIGLKDSVTAVVFVALGTSVPD
O.mossambicus_NCX1.1    TIFWKLLFAFVPPTDYW..NGWACFVVSITVIGMLTAVIGDLASHFGCTVGLKDSVTAVVFVALGTSVPD
O.mykiss_NCX1           TVFWKLLFAFVPPTDYW..NGWACFVVSISMIGLLTAFIGDLASHFGCTVGLKDSVTAVVFVALGTSVPD
D.rerio_NCX1a           TVFWKVLFAFVPPTDYW..NGWACFIVSIMMIGVLTAFIGDIASHFGCTIGLKDSVTAVVFVALGTSVPD
H.sapiens_NCX3.3        TVFWKVLFACVPPTEYC..HGWACFAVSILIIGMLTAIIGDLASHFGCTIGLKDSVTAVVFVAFGTSVPD
R.norvegicus_NCX3.3     TVFWKVLFACVPPTEYC..HGWACFVVSILIIGMLTAIIGDLASHFGCTIGLKDSVTAVVFVAFGTSVPD
M.musculus_NCX3.1       TVFWKVLFACVPPTEYC..PGWACFVVSILIIGMLTAIIGDLASHFGCTIGLKDSVTAVVFVAFGTSVPD
G.gallus_NCX3           TVFWKVLFACVPPTEYC..NGWACFVVSILIIGMLTAVIGDLASHFGCTIGLKDSVTAVVFVAFGTSVPD
D.rerio_NCX4a           TVFWKVLFAFVPPTEYW..NGWACFIVSITLIGVLTAVTGDLASHFGCTVGLKDTVTAVVFVALGTSVPD
T.nigroviridis_NCX4a    TVFWKVLFAFVPPTEYW..NGWACFFVSISLIGVLTAVTGDLASHFGCTIGLKDSVTAVVFVALGTSVPD
H.sapiens_NCX2.1        TVFWKVLFACVPPTEYC..HGWACFGVSILVIGLLTALIGDLASHFGCTVGLKDSVNAVVFVALGTSIPD
R.norvegicus_NCX2.1     TVFWKVLFACLPPTEYC..HGWACFGVCILVIGLLTALIGDLASHFGCTVGLKDSVNAVVFVALGTSIPD
M.musculus_NCX2.1       TVFWKVLFACVPPTEYC..NGWACFGVCILVIGVLTALIGDLASHFGCTVGLKDSVNAVVFVALGTSIPD
S.kowalevskii_3         SIFWKILFAFVPPTDIW..GGWACFTVSILMIGLLTAVIGDLASHFGCTVGLKDTVTAISFVALGTSVPD
T.adhaerens_2           TIFWKLIFALVPPTEIW..GGWACFCVSIIMIGILTTFIGDLASHFGCTVGLKDSVTAITFVALGTSLPD
L.opalescens_NCX        CLFWKVLFAFVPPTDYW..GGWACFTVSIILIGVLTAFIGDLATYFGCTIGLKDAVTAVSFVALGTSVPD
A.mellifera_NCX         TILWKVLFAFVPPTDIA..GGYLCFVVSIFGIGVVTAVIGDVASYFGCTLGIKDSVTAVAFVALGTSIPD
D.melanogaster_NCX      CLFWKVLFAFVPPTDIC..GGYVTFVVSIFVIGVITAIIGDAASYFGCALNIKDSVTAILFVALGTSIPD
H.magnipapillata        SFFWKVLFACVPPTDIW..GGWACFTASITMIGLLTAITGDIASHFGCTVGLADSVVAISFVALGTSLPD
C.elegans_NCX1          ALPWKLLFALIPPTDYF..NGWLCFVVAIAMIGLLTAFIGDIAAAFGCTVGLKDSVTALTLVAMGTSLPD
C.briggsae_NCX1         ALPWKLLFALIPPTDYF..NGWCCFVVAIIMIGVLTAFIGDIAAAFGCTVGLKDSVTALTLVAMGTSLPD
C.elegans_NCX2          TVPWKLTFATIPPTDYF..GGWATFVVAIFMIGVLTAVVGDLASQFGCWVGLKDAVTAISFVALGTSVPD
C.briggsae_NCX2         TVPWKLTFATIPPTDYI..GGWATFVVAIFMIGVLTAVVGDLASQFGCWVGLKDAVTAISFVALGTSVPD
T.spiralis              AFPWKVIAAFVPPTTIF..GGWLAFIVALALIGLLTAIIGDLAAIFGCLVGLKDSVTAITFVALGTSLPD
M.brevicollis_1         TLPWKVLAACIPPTVFVH..GWVAFGVALCFIGLVTALIGDLASLFGCSLGLADTITAITFVALGTSLPD
Salpingoeca             NIPWKIIAACLPPTLFLN..GWVTFGSALIMIGIVTATIGDLASLMGCAMGLEDAVTAITFVALGTSLPD
S.kowalevskii_1         TFGWKVIFALVPPPSFLNSGGWICFTVAISFIGLLTAIIGDLAAIFGCLIDLRESVVAITFVALGTSLPD
S.kowalevskii_2         SFGWKVIFALVPPPSFLDSGGWISFCVALTFIGVLTTIIGSLGSIFGCLINMKDSVTGLTFVTLGTCIVD
T.adhaerens_1           TFGWKVIFAIIPPPSML..GGWLTFIASLGMIGFLTAIVGDLAGIFGCIVGLRDAVTAITFVALGTSLPD
D.pulex_1               TFGWKLLFALVPPAGIW..GGWLSFFVSLVVIGALTAIVGDLASIFGCLVGLDDAVTAITFVALGTSLPD
P.humanus_corporis      SFNWKVLFSFIPPPSMF..RGWLCFIVSLCAIGFVTAVIEDIASLFGCVVGLKDTITAITLVAMGTSLPD
O.dioica                TFGFKTIFAIIPPSNMG..GGFPCFFGSLTMIGVMTVIISDLANIFGCLVGLKPAVNSITLVALGTSLPD
A.pisum                 SFFWKVVFAFIPPAAVFS..GWLRFFTSLTLIGVMATIIGDLATIFGCLIGLDDAVTAITIVALGMSLPD
P.marinus               TFPWKIVFAFCPPLEYA..DGWLTFFISLSMIGGVTAIIADMASLLGCTMDIPDSVTAITLVALGTSLPD
A.anophagefferens_1     TLPWKVAFAFVPPTSYG..GGWVCFYVALTFIGIVTAFIGDLAALMGCCLGLKDTVTAITFVALGTSLPD
E.huxleyi               ALPWRLLFALTPPPRVA..GGWACFFVALVGIGVITALVGDLAAHMGCCLGMKPAVTAITFVALGTSLPD
consensus>50            ...wkvlfafvPp..y....Gw..Fivsl..Ig.ltaiigdla..fgC.iglkd.vtavt.valGts.p#
```

```
                           420       430       440       450       460
A.thaliana                LVASKIAA..ERQLTADSAIANITCSNSVNIYVGIGVPWLINTVYNYF....................A
A.halleri                 LVASKIAA..ERQLTADSAIANITCSNSVNIYVGIGVPWLINTVYNYF....................A
A.lyrata                  LVASKIAA..ERQLTADSAIANITCSNSVNIYVGIGVPWLINTVYNYF....................A
C.sativus                 LVASKIAA..ERQTTADSAIANITCSNSVNIYVGIGVPWLISTTYNFI....................A
C.clementina              LVASKIAA..ERQITADSAIANITCSNSVNIYVGIGIPWLIDTAYNFI....................V
R.communis                LVASKIAA..ERQITADSAIANITCSNSVNIYVGIGVPWLIDTAYNFF....................A
M.esculenta               LVASKIAA..ERQTTADSAIANITCSNSVNIYVGIGVPWLIDTTYNFF....................V
P.trichocarpa             LVASKIAA..ERQTTADSAIANITCSNSVNIYVGIGVPWLIDTAYNFF....................M
P.persica                 LVASKIAA..ERQITADSAIANITCSNSVNIYVGIGVPWLIDTAYNFF....................V
C.papaya                  LVASKIAV..ERQITADSAIANITCSNSVNIYVGIGVPWLINTLYNFF....................A
A.coerulea                LVASKIAA..ERQTTADSAIANITCSNSVNIYIGIGIPWLIDTTYNFI....................A
V.vinifera                LVASKIAA..ERQTTADSAIANIICSNSVNIYMGIGIPWLINTTYNFI....................A
S.tuberosum               LVASKIAA..ERQLTADSAIANITCSNSVNIYIGIGVPWLINTLYNYI....................A
S.lycopersicum            LVASKIAA..ERQLTADSAIANITCSNSVNIYIGIGVPWLIDTLYNYI....................A
M.guttatus_1              LVASKIAA..ERQTTADSAIANITCSNSVNIYVGIGVPWLINTMYNYF....................A
M.guttatus_2              LVASKIAA..ERQITADSAIANITCSNSVNIYIGIGVPWLIDTLYNYI....................A
G.max                     LVASKIAA..ERQKTADSAIANITCSNSVNIYVGIGVPWLIDTLYNFI....................A
M.truncatula              LVASKIAA..KRQKTADSAIANITCSNSVNIYVGIGVPWLIDTLYNFI....................A
E.grandis                 LVASKIAA..ERQTTADSAIANITCSNSVNIYVGIGVPWLINTAYNFI....................M
S.italica                 LVASKIAA..ERQVTADSAIANITCSNSVNIYVGIGVPWLIDTVYNFF....................V
Z.mays                    LVASKIAA..ERQVTADSAIANITCSNSVNIYVGIGVPWLIDTVYNFF....................V
S.bicolor                 LVASKIAA..ERQVTADSAIANITCSNSVNIYVGIGVPWLIDTVYNFF....................V
T.aestivum                LVASKIAA..ERQVTADSAIANITCSNSVNIYVGIGVPWLINTVYNFF....................A
B.distachyon              LVASKIAA..ERQVTADSAIANITCSNSVNIYVGIGVPWLIDTVYNYF....................V
O.sativa_J1               LVASKIAA..ERQITADSAITNITCSNSVNIYVGIGVPWLVDTMYNYF....................V
O.sativa_I1               LVASKIAA..ERQITADSAITNITCSNSVNIYVGIGVPWLVDTMYNYF....................V
O.sativa_J2               LVASKIAA..ERQITADSAIANITCSNSVNIYVGIGVPWLVDTMYNYF....................V
S.moellendorffii_1        LMASKIAA..EEYDTADSAIANINASNCINVYVGFGVPWCVSALYSTG....................F
P.patens_1                LIASKIAA..EIQPTADSAIANINASNCINVYVGIGIPWLMQSFYNWI....................H
P.patens_2                LIASKIAA..KHLPTADSAIANINASNCINVYVGTGIPWLLQSFYNKL....................Q
C.reinhardtii             TLASRIAAVKDPD..ADAAIGNITGSNGVNVFLGLGLPWAVCSVYYHV....................R
C.intestinalis            TIASRTAALQDDY..ADASIGNITGSNSVNVFLGLGLPWVIRTMYFAV....................K
B.floridae                TFASRTVAIHDVH..ADAAIGNVTGSNSVNVFLGLGLPWVISTMYHLI....................N
T.adhaerens_3             TFASRNAAVHDHS..ADASIGNVTGSNSVNVFLGLGLPWVLVTTYRQI....................M
N.vectensis_2             TMASRSAALQDTG..ADAAIGNITGYNSVNVFLGLGLPWVMSTCYHAA....................R
H.sapiens_NCX1.1          TFASKVAATQDQY..ADASIGNVTGSNAVNVFLGIGVAWSIAAIYHAA.....................
P.troglodytes_NCX1.1      TFASKVAATQDQY..ADASIGNVTGSNAVNVFLGIGVAWSIAAIYHAA.....................
C.familiaris_NCX1.1       TFASKVAATQDQY..ADASIGNVTGSNAVNVFLGIGVAWSIAAIYHAA.....................
F.catus_NCX1.1            TFASKVAATQDQY..ADASIGNVTGSNAVNVFLGIGVAWSIAAIYHAA.....................
C.porcellus_NCX1.1        TFASKVAATQDQY..ADASIGNVTGSNAVNVFLGIGVAWSIAAIYHAA.....................
B.taurus_NCX1.1           TFASKVAATQDQY..ADASIGNVTGSNAVNVFLGIGVAWSIAAIYHAA.....................
R.norvegicus_NCX1.1       TFASKVAATQDQY..ADASIGNVTGSNAVNVFLGIGVAWSIAAIYHAA.....................
M.musculus_NCX1.1         TFASKVAATQDQY..ADASIGNVTGSNAVNVFLGIGVAWSIAAIYHAA.....................
M.mulatta_NCX1.3          TFASKVAATQDQY..ADASIGNVTGSNAVNVFLGIGVAWSIAAIYHAA.....................
O.cuniculus_NCX1.2        TFASKVAATQDQY..ADASIGNVTGSNAVNVFLGIGVAWSIAAIYHAA.....................
S.tropicalis_NCX1         TFASKVAATQDQY..ADASIGNVTGSNAVNVFLGIGVAWSIAAIYHAA.....................
O.mossambicus_NCX1.1      TFASKVAAIQDQY..ADASIGNVTGSNAVNVFLGIGVAWSIAAVYHYT.....................
O.mykiss_NCX1             TFASKVAAIQDQY..ADAFIGNVTGSNAVNVFLGIGVAWSIAAIYHNS.....................
D.rerio_NCX1a             TFASKVAAIQDQY..ADASIGNVTGSNAVNVFLGIGVAWSIAAIFHQS.....................
H.sapiens_NCX3.3          TFASKAAALQDVY..ADASIGNVTGSNAVNVFLGIGLAWSVAAIYWAL.....................
R.norvegicus_NCX3.3       TFASKAAALQDVY..ADASIGNVTGSNAVNVFLGIGLAWSVAAIYWAM.....................
M.musculus_NCX3.1         TFASKAAALQDVY..ADASIGNVTGSNAVNVFLGIGLAWSVAAIYWAM.....................
G.gallus_NCX3             TFASKAAAIQDVY..ADASITNVTGSNAVNVFLGIGLAWSVAAIYWAS.....................
D.rerio_NCX4a             TFASKVAAIQDQY..ADASIGNVTGSNAVNVFLGIGVAWTIAAVYWHS.....................
T.nigroviridis_NCX4a      TFASKVAAIQDQY..ADASIGNVTGSNAVNVFLGIGVAWTIAAVYWHS.....................
H.sapiens_NCX2.1          TFASKVAALQDQC..ADASIGNVTGSNAVNVFLGLGVAWSVAAVYWAV.....................
R.norvegicus_NCX2.1       TFASKVAALQDQC..ADASIGNVTGSNAVNVFLGLGVAWSVAAVYWAV.....................
M.musculus_NCX2.1         TFASKVAALQDQC..ADASIGNVTGSNAVNVFLGLGVAWSVAAVYWAV.....................
S.kowalevskii_3           TFASKTAAVGDKY..ADASVGNVTGSNAVNVFLGIGLAWSAAAIYWAA.....................
T.adhaerens_2             TFASKTAAVNDKY..ADSSIGNVTGSNSVNVFLGLGLAWCVAAVANAI.....................
L.opalescens_NCX          TFASKVAAINDKY..ADSSIGNVTGSNAVNVFLGIGIAWSIAAIYHAA.....................
A.mellifera_NCX           TFASKVAACQDKY..ADASVGNVTGSNAVNVFLGIGVAWSIAAIYHAL.....................
D.melanogaster_NCX        TFASMIAAKHDEG..ADNCIGNVTGSNAVNVFLGIGLAWTIAAVYHSS.....................
H.magnipapillata          TFASKVATINDEH..ADGSIGNVTGSNSVNVFLGIGLAWSIAAIYHAS.....................
C.elegans_NCX1            TFASRTAAVGDQW..ADGSIGNVTGSNAVNVFLGIGIAWMIAACVHAY.....................
C.briggsae_NCX1           TFASRTAAVGDQW..ADGSIGNVTGSNAVNVFLGIGIAWMIAACVHAY.....................
C.elegans_NCX2            TFASKVSAVQDKY..ADNAVGNVTGSNAVNVFLGIGIAWSMAAIYHWN.....................
C.briggsae_NCX2           TFASKVSAVQDKY..ADNAVGNVTGSNAVNVFLGIGIAWSMAAIYHWN.....................
T.spiralis                TFASRLAALQDKT..ADNAIGNINGSNSVNVFLGLGLPWFIASVYWAA.....................
M.brevicollis_1           TFASRSATLASKT..ADAAVTNVTGSNSVNVFLGLGLSWFIAAVYWPA....MGATDQWRARVPTCLQQK
Salpingoeca               TFASMSATIASDT..ADAAITNVTGSNSVNVFLGLGLSWIIAAIYWPV....VGKTDEWIATVPPCINAK
S.kowalevskii_1           LFASKTAAVNEKY..ADASIGNVTGSNSVNVFLGLGLSWLIASIYWTS....KGL..............
S.kowalevskii_2           LCVSKKASVGEKY..ADCCM...TGSYSVYVFFGLGLSWVTASIYWTS....KGL..............
T.adhaerens_1             LFASKTAALNEKY..ADNSIGNVTGSNSVNVFLGLGLPWLIATIYWNS....QNK..............
D.pulex_1                 LFASRAAAMNEKY..ADNAIGNVTGSNSVNVFLGLGLPWLIASIYHAS....NGN..............
P.humanus_corporis        TFASRTAALKEQY..ADTSIGNITGSNSVNVFLGLGLPWLMATIYHTH....NGT..............
O.dioica                  LFASKIAATNEPN..ADDAVGNVTGSNSVNVFLGLGLPWSMAAIYHYC....NGS..............
A.pisum                   ILGACMVTRAETH..ADEAMIHIAGSIAVKVLMGVGLPWFISALYHYS....HGN..............
P.marinus                 TFASRLAAMQDPY..ADASIGNVTGSNSVNVFLGLGLPWVIGSLYWS....AKGATQEWIARVGLDIYTK
A.anophagefferens_1       AFASKAATINDDS..ADAAVGNVTGSNSVNVFLGLGLPWSIAAIYWS....GGFAGGS..AEKQWRIKYG
E.huxleyi                 TFASRTAAVKERERHADSSIGNITGSNSVNVFLGLGLPWLAAALFWASPRGAAQEASWRDRYRGEAWYSE
consensus>50              tfaskiaa..d....AD.aignvtgsns!n!flGiGvpW.ia.iy.......................
```

```
                                 470       480       490       500       510       520       530
                                   .         .         .         .         .         .         .
A.thaliana                  YREPLYIENAKGLSFSLLIFFATSVGCIVVLVLRR...LIIGAELGGPRLWAWLTSAYFMMLWVVFVVLS
A.halleri                   YREPLYIENAKGLSFSLLIFFATSVGCIVVLVLRR...LIIGAELGGPRLWAWLTSAYFMMLWVVFVVLS
A.lyrata                    YREPLYIENAKGLSFSLLIFFATSVGCIVVLVLRR...LIIGAELGGPRLWAWLTSAYFMMLWVVFVVLS
C.sativus                   YKEPLKIKDAGGLSFSLLVFFSTSVACIVVLVFRR...VTLGAELGGPKVWAWITCIFFMVLWVIFVVLS
C.clementina                YKEPLRVQNAEGLSFSLLVFFCTSVGCIAVLVLRR...LTLGAELGGPKLWAWVTSVYFMLLWIIFVVLS
R.communis                  YREPLRVQDAAGLSFSLLVFFSTSVGCIAVLVYRR...LTLGAELGGPRIWAWVTSVYFMFLWLIFVVLS
M.esculenta                 YRQPLRIENAEGLSFSLLVFFATSVGCIGVLVYRR...LTLGAELGGPRIWAWVTCVYFMLLWLIFVVLS
P.trichocarpa               YNEPLRIQNAAGLSFSLLVFFCTSIGCIAVLVLRR...LTLKAELGGPRIWAWVTFVYFMLLWIIFVVLS
P.persica                   YKEPLRIENAAGLSFSLLVFFSTSVGCIAVLVIRR...RTLGAELGGPRLWAWITFVFFMLLWLIFVVLS
C.papaya                    YREPLGIQNAEGLSFSLLVFFSTSVGCIAVLVFRR...LTLGAELGGPRFWAWVTCVYFMLLWIIFVVLS
A.coerulea                  YREPLRIENADGLSFSLLIFFLTSIGCISVLVLRR...LTIGAELGGPRIWAWITSVYFMSLWIVFVVLS
V.vinifera                  YREPLGVQNAEGLSFSLLVFFCTSICCIGVLVLRR...LTLGAELGGPKIWARLTCVYFMSLWIIFVVLS
S.tuberosum                 YNEPLRIDNAEGLSFSLLVFFSTSVACIGVLVFRR...LTIGAELGGPRVWAWVTCIFFMLLWLIFVVLS
S.lycopersicum              YNEPLRIENAEGLSFSLLVFFSTSVACIGVLVFRR...LTIGAELGGPRVWAWVTCIFFMLLWLIFVVLS
M.guttatus_1                YNEPLRIENAGGLSFSLLVFFATSIGCIGGLVFRR...LTLGAELGGPKIWAWITCAYFMFLWLIFVVLS
M.guttatus_2                YKKPLRIENAAGLSFSLLIFFATSVGCIGVLVFRR...LTLGAELGGPRHWAWLTCFYLMLLWLVFVVLS
G.max                       YREPLRIQNAGGLSFSLIVFFSTSVGCISVLVLRR...IIFGAELGGPRLWAWITCAFFMLLWIIFVVLS
M.truncatula                YRQPLRIQNAGGLSFSLIVFFATSVGCISVLVARR...IVFGAELGGPRLWAWITCAFFMLLWIIFVVLS
E.grandis                   YREPLRIQNAAGLSFSLIVFFSTSVGCISVLVLRR...LTLGAELGGPKPWAWLTSVYLMLLWVIFLVLS
S.italica                   YREPLYIDNAAGLSFSLLVFFATSFGCITVLVLRR...IIIGAELGGPRLWAWITSVYFMILWIVFVVFS
Z.mays                      YREPLYIDNAAGLSFSLLVFFATSFGCITVLVLRR...IILGAELGGPRLWAWVTSVYFMILWVVFVVFS
S.bicolor                   YQEPLYIDNAAGLSFSLLVFFATSFGCITVLVLRR...IILGAELGGPRLWAWVTSVYFMILWVVFVVFS
T.aestivum                  YQEPLYIDNAAGLSFSLLVFFATSFGCITVLVLRR...IVFGAELGGPRLWAWATSAYFMVLWVVFVLLS
B.distachyon                YQEPLYIDNAAGLSFSLLVFFATSFGCITVLVLRR...IVLGAELGGPRLWAWVTSTYFMVLWVVFVVLS
O.sativa_J1                 YQKPLYIDNAAGLSFSLLVFFATSFGCITVLVLRR...VILGAELGGPRMWAWATSVYFMILWVVFVVLS
O.sativa_I1                 YQKPLYIDNAAGLSFSLLVFFATSFGCITVLVLRR...VILGAELGGPRMWAWATSVYFMILWVVFVVLS
O.sativa_J2                 YKKPLYIDNAAGLSFSLLVFFATSFGCITVLVLRR...VILGAELGGPRMWAWATSVYFMILWVVFVVLS
S.moellendorffii_1          HQNLV.VPVE.GLKFLLSVYFSTAITCFVALTARR...RLLGGELGGPRKWAWASAIFFFLLWITFLTLA
P.patens_1                  LKEEFRVPSA.GLGFALVLFFVTFAICLVVIIARR...FLFGGELGGPRKWAWVSSFCFLSLWLIFVIFS
P.patens_2                  LDEEFRVPSV.GIGFSLMLFLVTFVLCQIVVVGRR...FIFEGELGGPRKWAWASSFYFIFLWLIFVIFS
C.reinhardtii               GEK..YVTPGGDLEFAVMLYAILGGCGIFILAVAR...Y.FGGELGGTKLRQYSIAGLLTVLWLLYLILS
C.intestinalis              GGQ..YIVDTDGLDFAVTLFDSFGAVCVVLLILRR...YVLGGELGGSTRVKWASGIFLFTLWFIFIIVS
B.floridae                  GTQ..YRVLPGNLRFAVFTFLAVGSLCLLMLFVRR...KCFGGELGGKKVPKWISALLMFSFWVVFIVLC
T.adhaerens_3               FSNSDYAISPGDMQFSVILFTVVAGAGVAILLFRR...FVLGGELGGDIKWQYITAATLVLLWAVYITLA
N.vectensis_2               GTV..YKVSSGNLAFSVLVFALCGGVCLFLLVLRR...YLYKGELGGPTLSKWMTGLLLLFLWFIYVVLS
H.sapiens_NCX1.1            .NGEQFKVSPGTLAFSVTLFTIFAFINVGVLLYRRRPEI.G.GELGGPRTAKLLTSCLFVLLWLLYIFFS
P.troglodytes_NCX1.1        .NGEQFKVSPGTLAFSVTLFTIFAFINVGVLLYRRRPEI.G.GELGGPRTAKLLTSCLFVLLWLLYIFFS
C.familiaris_NCX1.1         .NGEQFKVSPGTLAFSVTLFTIFAFINVGVLLYRRRPEI.G.GELGGPRTAKLLTSCLFVLLWLLYIFFS
F.catus_NCX1.1              .NGEQFKVSPGTLAFSVTLFTIFAFINVGVLLYRRRPEI.G.GELGGPRTAKLLTSCLFVLLWLLYIFFS
C.porcellus_NCX1.1          .NGEQFKVSPGTLAFSVTLFTIFAFINVGVLLYRRRPEI.G.GELGGPRTAKLLTSCLFVLLWLLYIFFS
B.taurus_NCX1.1             .NGEQFKVSPGTLAFSVTLFTIFAFINVGVLLYRRRPEI.G.GELGGPRTAKLLTSCLFVLLWLLYIFFS
R.norvegicus_NCX1.1         .NGEQFKVSPGTLAFSVTLFTIFAFINVGVLLYRRRPEI.G.GELGGPRTAKLLTSSLFVLLWLLYIFFS
M.musculus_NCX1.1           .NGEQFKVSPGTLAFSVTLFTIFAFINVGVLLYRRRPEI.G.GELGGPRTAKLLTSSLFVLLWLLYIFFS
M.mulatta_NCX1.3            .NGEQFKVSPGTLAFSVTLFTIFAFINVGVLLYRRRPEI.G.GELGGPRTAKLLTSCLFVLLWLLYIFFS
O.cuniculus_NCX1.2          .NGEHFKVSPGTLAFSVTLFTIFAFINVGVLLYRRRPEI.G.GELGGPRTAKLLTSCLFVLLWLLYIFFS
S.tropicalis_NCX1           .NGDVFRVQPGTLAFSVTLFTIFAFINVGVLLYRRRPEI.G.GELGGPRTAKLLTTALFTLLWLLYIFFS
O.mossambicus_NCX1.1        .QGQEFKVNPGTLAFSVTLFTIFAFICIAVLIYRRRPEI.G.GELGGPRIPKILTTCLFFSLWLMYIVFS
O.mykiss_NCX1               .KGNDFRVDPGTLAFSVTLFTIFAFVAVAVLMYRRRPEI.G.GELGGPRGPKIATTCLFFSLWLMYIVFS
D.rerio_NCX1a               .QGRQFRVDPGTLAFSVTLFTIFAFVCIAVLMYRRRPEI.G.GELGGPRHPKIITTTLFFSLWLMYIILS
H.sapiens_NCX3.3            .QGQEFHVSAGTLAFSVTLFTIFAFVCISVLLYRRRPHL.G.GELGGPRGCKLATTWLFVSLWLLYILFA
R.norvegicus_NCX3.3         .QGQEFHVSAGTLAFSVTLFTIFAFVCLSVLLYRRRPHL.G.GELGGPRGCKLATTWLFVSLWLLYVLFA
M.musculus_NCX3.1           .QGQEFHVSAGTLAFSVTLFTIFAFVCLSVLLYRRRPHL.G.GELGGPRGCKLATTWLFVSLWLLYILFA
G.gallus_NCX3               .QGQEFQVSAGTLAFSVTLFTIFAFICISVLLYRRRPHL.G.GELGGPRGCKLATTLLFVSLWLLYILFA
D.rerio_NCX4a               .QGKKFQVPPGSLAFSVTLFTILALVCVVTLLYRRRPSVSG.GELGGPRTPKLLTAFLFIVLWLIYIMLA
T.nigroviridis_NCX4a        .KGKPFKVNPGSLAFSVTLFTIMALVCVLVLLYRRRPSVSG.GELGGPRTAKLVTFFLFISLWFVYILLA
H.sapiens_NCX2.1            .QGRPFEVRTGTLAFSVTLFTVFAFVGIAVLLYRRRPHI.G.GELGGPRGPKLATTALFLGLWLLYILFA
R.norvegicus_NCX2.1         .QGRPFEVRTGTLAFSVTLFTVFAFVGIAVLLYRRRPHI.G.GELGGPRGPKLATTALFLGLWFLYILFA
M.musculus_NCX2.1           .QGRPFEVRAGTLAFSVTLFTVFAFVCIAVLLYRRRPQI.G.GELGGPRGPKLATTALFLGLWFLYILFS
S.kowalevskii_3             .KGEVFEVKPGSLAFSVTIYSIFAVIAITVLMIRRKLPNTL.GELGGPKTTKIITSCFLVGLWLLYLLLC
T.adhaerens_2               .KGEKFYVPSGSLAFSVTIFCAVAIVCIAVLMLRRTKSV.G.GELGGPRGPKYATAVFFIFLWLLYIILS
L.opalescens_NCX            .NGTVFRVDPGTLAFSVTIFCVFAVCTIVLLVCRRHHLV.G.GELGGPPRCKYITSGILGSFWVSYLLLT
A.mellifera_NCX             .HGDKFRVKPGNLAFCVTLFCTEACLVILVLLLRRT.KSIG.GELGGPFVPKVITSLILFFLWVFYLIMS
D.melanogaster_NCX          .HGMTFNVEPGTIGFAVALFCGEALIAIMLIMFRRWHKGIG.AELGGPKVSKYISAAILVFLWVFYVVIC
H.magnipapillata            .NGNKFEVDPGSLGFSVMLFCLEASVCIGILMYRRSSKNIR.AELGGPSISRYITFTTFLLLWIIYLLVS
C.elegans_NCX1              .RGTKFLVATGSLAFSVTMFLIGSVVCVALLQYRRFNRKVN.GELGGPMGWRIISAGIFVSVWLLYILLS
C.briggsae_NCX1             .RGTKFLVSTGSLAFSVTMFLIGSVICVALLQYRRFNRKIN.GELGGPTSWRVISAGIFVSVWLLYILLS
C.elegans_NCX2              .QGTKFLVDPGNLGFSVLIFCTEAVLCIIVLVLRR.NKKVG.GELGGPIALRWMTAMFFASLWFMYLLLS
C.briggsae_NCX2             .KGTKFLVDPGNLGFSVLIFCVEALLCIAVLVLRR.SKKVG.GELGGPTALRWMTSMFFCSLWLMYLLLS
T.spiralis                  .RGEAFVVHSGDVGFSVTIYTVLSLICIVMMMLRRFLPALGEAELGGPTIPKWICSVTLILFWFLYILLS
M.brevicollis_1             HDGAFFYVPAGDLAFSVVIFTCCCLVCLGSLLFRR...YKFGAELGGPYSRQ..FAFFFIGLWIFYVLMS
Salpingoeca                 YSNAVFWVPSGDLAFSVIIFSLCCIICLGTLVVRR...YTVGAELGGAYAKP..TAALFLTLWLMYILLS
S.kowalevskii_1             ....VFEMEAGALSFSVMIYTICAIMCLSLLVLRRFIPFFGKAELGGPTAGRYSSSIFLILLWIIYILLC
S.kowalevskii_2             ....VFVVDSSSFSFSVMMYIICAIMCLSLLVLRRFTPFFGKAELGGPTAGRYLSSIFLLLLWCLYILLC
T.adhaerens_1               ....EFAVPAGNLASSVILFTICAVICIALLMLRRIIPSFGGSELGGPTKSKYISGLLLVTLWIVYVVIS
D.pulex_1                   ....EFKVEAGSLGFSITVFTITAVLSLALLVTRRCSAACGRGELGGPLGTRYASAIFLLVLWMAYVLLS
P.humanus_corporis          ....VFKVPAGTLGFSVLLFTISAVIAIALLIGRRNSSFCGKAELGGSVFFKLLSGGLLIILWVFYIVMS
O.dioica                    ....TFNVPPGSLVFSVTIFSICAVCTIAFLFLRRTLSYFGNAELGGPQKQKWFSGIFLICLWLFYVLMS
A.pisum                     ....LFEIPSTRIGFNVLLYSVMAILAVTVLMVRRNVEFFGKAELGGPTKGRYITVAILISLWVIYLSLT
P.marinus                   YPDGGFVVEAGDLSFSVMIFTICAFLCLTVLVARR...AKYGGELGGTKHGKRISAAILTYALEVDVTSN
A.anophagefferens_1         GKDVAFIVPAGNLGLSVGVFVFCACTCLGVLAYRR...KAYGAELGGDRTMANRHATLFVMLWFLYVAMS
E.huxleyi                   AMPVAFVVASGDLGFSVAVFTVLALVCLAVLVARR...ATVGGELGGGAASKYSTATLFTALWLGYIWLS
consensus>50                .....f.vn.g.l.fsv.v%.i.a..ci.vlv.rR.......gELGGpr.....t...f..lw..yv.ls
```

**A.thaliana**	SLKVSGVI...................
**A.halleri**	SLKVSGVI...................
**A.lyrata**	SLKVSGII...................
**C.sativus**	SLKVSDII...................
**C.clementina**	SLKVSGII...................
**R.communis**	SLRVSGII...................
**M.esculenta**	SLRVSGFI...................
**P.trichocarpa**	SLRVSGII...................
**P.persica**	SLKVSGII...................
**C.papaya**	SLRVSGII...................
**A.coerulea**	SLKVSDII...................
**V.vinifera**	SLKIYGII...................
**S.tuberosum**	SLRVSGII...................
**S.lycopersicum**	SLRVSGII...................
**M.guttatus_1**	SLRVSGFI...................
**M.guttatus_2**	SLQVSNII...................
**G.max**	SLKVSGFI...................
**M.truncatula**	SLKVSGFI...................
**E.grandis**	SLKVSGII...................
**S.italica**	SLKVYGII...................
**Z.mays**	SLRVSGVI...................
**S.bicolor**	SLRVSGVI...................
**T.aestivum**	SLRVSGVI...................
**B.distachyon**	SLRVSGVI...................
**O.sativa_J1**	SLKISGVI...................
**O.sativa_I1**	SLKISGVI...................
**O.sativa_J2**	SLRISGVI...................
**S.moellendorffii_1**	CLAGRFT....................
**P.patens_1**	CLRNYHHL...................
**P.patens_2**	CLRNYNLL...................
**C.reinhardtii**	GLRAYGNI...................
**C.intestinalis**	SLKAYNYF...................
**B.floridae**	SLKAYDLLPF.................
**T.adhaerens_3**	SLRAYDVIRFQ................
**N.vectensis_2**	SLMAYGHISGSL...............
**H.sapiens_NCX1.1**	SLEAYCHIKGF................
**P.troglodytes_NCX1.1**	SLEAYCHIKGF................
**C.familiaris_NCX1.1**	SLEAYCHIKGF................
**F.catus_NCX1.1**	SLEAYCHIKGF................
**C.porcellus_NCX1.1**	SLEAYCHIKGF................
**B.taurus_NCX1.1**	SLEAYCHIKGF................
**R.norvegicus_NCX1.1**	SLEAYCHIKGF................
**M.musculus_NCX1.1**	SLEAYCHIKGF................
**M.mulatta_NCX1.3**	SLEAYCHIKGF................
**O.cuniculus_NCX1.2**	SLEAYCHIKGF................
**S.tropicalis_NCX1**	SLEAYCHIKGF................
**O.mossambicus_NCX1.1**	SLEAYCHVKGF................
**O.mykiss_NCX1**	SLEAYCHVKGF................
**D.rerio_NCX1a**	SMEAYCIIKGF................
**H.sapiens_NCX3.3**	TLEAYCYIKGF................
**R.norvegicus_NCX3.3**	TLEAYCYIKGF................
**M.musculus_NCX3.1**	TLEAYCYIKGF................
**G.gallus_NCX3**	TLEAYCYIKGF................
**D.rerio_NCX4a**	SLEAYCHVPGF................
**T.nigroviridis_NCX4a**	SLEAYCHVHSF................
**H.sapiens_NCX2.1**	SLEAYCHIRGF................
**R.norvegicus_NCX2.1**	SLEAYCHIRGF................
**M.musculus_NCX2.1**	SLEAYCHIRGF................
**S.kowalevskii_3**	SFESYCYIEGF................
**T.adhaerens_2**	SLESYCHIPGF................
**L.opalescens_NCX**	GLMSYCHIPGF................
**A.mellifera_NCX**	ILEAYGYIEGF................
**D.melanogaster_NCX**	ILEAYDVIRV.................
**H.magnipapillata**	GLESYCYFTLKF...............
**C.elegans_NCX1**	TLEAYCIIKGF................
**C.briggsae_NCX1**	TLEAYCVIKGF................
**C.elegans_NCX2**	ALEAYCIIPGF................
**C.briggsae_NCX2**	ALEAYCIIPGF................
**T.spiralis**	ALQAYGHISSF................
**M.brevicollis_1**	SLQVEGYIASI................
**Salpingoeca**	SLSTYEHISL.................
**S.kowalevskii_1**	SLQAYGYIHF.................
**S.kowalevskii_2**	SFQSYDYIDF.................
**T.adhaerens_1**	SLQVYRIIPPIVKT.............
**D.pulex_1**	SLETYGHITGF................
**P.humanus_corporis**	CLKEYRVIDVNF...............
**O.dioica**	TLQAYEFIPGF................
**A.pisum**	CFQPF......................
**P.marinus**	GVALNSNLHLNYRDASDVDRGEY....
**A.anophagefferens_1**	ILASEGIV...................
**E.huxleyi**	IAKTEGDPRLARLLQQLPPQARALLGA
consensus>50	**sle.y..i...................**
