# Supplementary material for: Phylogeny and a structural model of plant MHX transporters
Source: BMC Plant Biol. 2013 May 2;13:75. doi: 10.1186/1471-2229-13-75 (PMC3679957; doi:10.1186/1471-2229-13-75)
Supplement: Additional file 10 — A TMpred-based prediction of MHX TMSs. [file 1471-2229-13-75-S10.doc]

**Additional file 10. A TMpred-based prediction of MHX TMSs**

The sequences of all currently identified MHX proteins were aligned by Multalin. Each protein was named by the first letters of the genus and species of its source organism, except when there were more than one protein from the same plant. Ppe refers to *P. persica*, and Pp to *P. patens*. Identical residues are indicated by uppercase letters, highlighted in gray, in the consensus (Cons) sequence. Similar residues, or the most conserved residues in each position, are indicated by lowercase letters in the consensus sequence. The similarity codes are ! - I or V, $ - L or M, % - F or Y, and # - D, E, N, or Q. Dots indicate gaps. Regions predicted to be TMSs by the TMpred algorithm are indicated by bold, underlined letters on the sequence of each protein. Residues predicted to be localized in a TMS in more than 50% of the sequences are indicated by bold, underlined letters on the consensus sequence. The indication of TMS numbers, loop orientation, and the positions of the reentrant loops are based on the experimentally-determined structure of the corresponding regions of NCX1.

**N-ter (out)**

At .......................MASILNQTQELQESSKVLGHLRCENFFLFPGENTLSDGLR**GVLYFLG**

Ah .......................MASILNQTQELQEASKVLGHVRCENFFIFPGENTLSDGLR**GVLYFLG**

Al .......................MASILNQTQELQEASKVLGHVRCENFFIFPGENTLSDGLR**GVLYFLG**

Eg MQ**ACFASVRLSLSSSLDSLPIFSVSFRR**AGTPGDISRSHMMGHGVCESYLLFPGETALGDAFRT**FLYFLG**

Cc ......................MASIHTQAIESISRAFNILHYEKCESYLLFRGETSLGDGFR**AFLYFLG**

Rc .....................MALETNYQSGWSRFGLLNILASEKCESYLVFRGETGLSVGFRT**FLYFLG**

Me .....................MALETQNQSAGSKFGLLNILGRERCESYLLFHGETALDNGFRT**FLYFLA**

Pt ......................MASAYNQSDGGRFGDTNIVGHEKCESYFLFRGETTLGEGFRT**FLYFLG**

Ppe .................................MPGNSSTMGQENCESYFIFQGETGLGVGFRT**FLYFLG**

Cp ......................MTWAHWRNAESIIQNFNVLEQEKCESYLIFRSETALSNGFR**AFLYFLG**

Ac ............................................KCESYFLFQGEMALGNGLR**AFLYLLG**

Vv ......................MAWVHEQLPGNILEAYNISVREKCESYLLFSGETSLGDGFRT**FLYFLG**

Gm .................................MVPFSNILGHEKCESYLIFGGETTLGDNFR**AFFYFLC**

Mt ............................MYKMIGYLSSFLAHDEKCESYLIFSGETNLGNSVR**ILLYFLG**

St ......................MTSLGNYTTDSTNGHSNILRHEKCDAYLLIHLETALGEGFRT**FLYFLG**

Sl ......................MTSLGNYTTHSTNGHSNILRHEKCDAYLLFHLETALGGGFRT**FLYFLG**

Mg1 ......................MDPLNNSTAEIIDGGSNILGVEKCQLYLLFHDETVLSDGFR**GFLYFMA**

Mg2 ...........................MASLNSSSTWDSNLGHERCLFYFIFHFETKLSH**GLRGFLYFLA**

Cs ......................MASFAIENVESGQAISSISGTGKCESYFIFSIETSLGDALR**IFLYFMG**

Si .....................................MSGPSSACEGTYLLFHGETLLSGGVRAS**LYTVA**

Zm ..........................MAGAATPPPSSSSSASCGTDAAYLLFRGETLLPDGVHAS**LYAVA**

Sb .......................MSNIAMVAAPPSSSCDSDT......YLLFHGETLLSTGARAS**LYTVA**

Ta ......................................MGSTTRSCDAYLLFNGETLLPNGVR**AFLCTVA**

Bd .................................MANISMGSNAPSCNAYLLFYAEKMLPNGVRA**FAYTVA**

OsJ1 .................................MANINMADTVPSCDTYLLFNGETLLPIGVR**AFIYTAV**

OsI1 .................................MANINMADTAPSCDTYLLFNGETLLPIGVR**AFIYTAV**

OsJ2 .................................MANINMADTAPSCDTYLLFNGETLLPNGVR**AFIYTVV**

OsI2 .................................MANINMADTVPSCDTYLLFNGETLLPNGVR**AFIYTVV**

Sm .............................................CPSLLLTQEEQRWRFGTR**AFAYCLG**

Pp1 ............MAGFTNASISAHPSPGVWTNHFANAAVNATAELCSNHIIFKAEPSWW**LSARAILYGLC**

Pp2 .............AGLSSALVSAFSSPEKNTIHVVHASATSTAELCPNHIMFEAEPTWLHSSR**AVLYAFC**

Cons ...........................................e.ce.yllf.gEt.l.dg.r**aflyflg**

**TMS 1 loop a (in) TMS2**

At **LAYCFIGLSAITA**RFFK...SMENVVKHSRKVVT.IDPITKAEVITYKK**VWNFTIADISLLAFGTSFPQI**

Ah **LAYCFIGLSAITA**RFFK...SMENVVKHSRKVVA.IDPITKAEIITYKK**VWNFTIADISLLAFGTSFPQI**

Al **LAYCFIGLSAITA**RFFK...SMENVVKHSRKVVA.VDPITKAEVITYKK**VWNFTIADISLLAFGTSFPQI**

Eg **LAYCFIGLSAITA**RFFS...SMENVVKQTREVVE.IDPQTNSKVVRQEKVWN**YTIADITLLAFGTSFPQI**

Cc **LAYCFIGLSAITA**RFFR...SMENVVKHSRKVVE.IDPVTKAEVVRYEK**VWNYAIADIALLAFGTSFPQI**

Rc **LAYCFIGLSAITG**RFFR...SMENVVKHSRKVVE.IDPYSNTEVIRYEK**VWNYTIADISLLAFGTSFPQI**

Me **LAYCFFGLSAITA**RFFR...SMENVVKHSRKIVE.VDPDTNTEVIRYEKVWN**YAIADISLLAFGTSFPQI**

Pt **LAYCFIGLSAITA**RFFR...SMENVVKQSRKVVE.IDPYTNAEVIRYEKVWN**YAIADISLLAFGTSFPQI**

Ppe **LAYCFVGLSAITA**RFFQ...SMESVVSHTRKVVD.INPYTGAEIIRYEK**VWNFTIADISLLAFGTSFPQI**

Cp **IAYCFLGLSAITGR**FFR...SMEHVVKHTREVVE.IDPITNQEITRHEKVWN**YTIADISLLAFGTSFPQI**

Ac **LAYCFFGLSAITA**RFFR...SMENVVKQTRKVVE.IDHNTNTEVIRHEK**VWNYAIADITLLAFGTSFPQI**

Vv **LAYCFIGLSAITA**RFFQ...SMENVVKHKRKVVK.IDPRSNTE.IRHEK**VWNYTIADITLLAFGTSFPQI**

Gm **LAYCFIGLSAITA**RFFQ...SMENVVKHTRKVVE.VDPVTKTETIRHEK**VWNYTIADISLLTFGTSFPQI**

Mt **LAYCFIGLSAITS**RFFQ...SMENVVKHSREVVV.IDPVTKAETIRHEK**VWNYTIADISLLAFGTSFPQI**

St **LAYCFIGLSAITA**RFFR...SMESVVKHSRTVET.IDPLTNTKIVKNEK**VWNYTIADITLLAFGTSFPQI**

Sl **LAYCFIGLSAITA**RFFR...SMESVVKHSRTVET.IDPLTNTKAVKNEK**VWNYTIADITLLAFGTSFPQI**

Mg1 **LAYCFIGLSAITS**RFFQ...SMENVVKHTRTVEE.IDPCTNTKTVRHEK**VWNYTIADITLLAFGTSFPQI**

Mg2 **LAYCFVGLSAIT**DRFFR...SMENVVKHSRAVEE.IDPLTNTKVIKYEK**VWNYTIADITLLAFGTSFPQI**

Cs **LAYCFVGLSAITA**RFFR...SMENVVKHSRKVVE.IDPHTNTEIIRYEK**VWNFTIADISLLAFGTSFPQI**

Si **LAYCFIGLSAITA**RFFK...SMEQIMKHSREVVS.VDPHTNAPVVKQEKVWN**YTIADIALLAFGTSFPQI**

Zm **LAYCFVGLSAVTA**RFFK...SMEQIMRHSREVVVGVDPRTGAPVVRRDK**VWNYTVADIALLAFGTSFPQI**

Sb **LAYCFIGLSAITA**RFFK...SMEQIMKHSREVVVI.DPHTKEPVVKHEK**VWNYTVADIALLAFGTSFPQI**

Ta **LAYCFIGLSAITA**RFFK...SMESITNHSREVVT.VDTETNTPIVKHEK**VWNYTIADIALLAFGTSFPQI**

Bd **LGYCFIGLSAITARFF**K...SMESITNHSREVVT.IDPHTNTPIVKHEKVWNYT**IADIALLAFGTSFPQI**

OsJ1 **LAYCFIGLSAITG**RFFK...SMESIMRHSREVVT.VDPHTNATIVKHEK**VWNYTIADVALLAFGTSFPQI**

OsI1 **LAYCFIGLSAITG**RFFK...SMESIMRHSREVVT.VDPHTNATIVKHEK**VWNYTIADVALLAFGTSFPQI**

OsJ2 **LAYCFIGLSAISG**RFFK...SMESIMRHSREVVT.IDPHTNATIVKHEK**VWNYTIADVALLAFGTSFPQI**

OsI2 **LAYCFIGLCAITG**RFFK...SMESIMRHSREVVT.VDPHTNATIVKHEK**VWNYTIADVALLAFGTSFPQI**

Sm **LAYCFLGLAVIT**RVYMRALDS....IVRHSRKSVHRDPITGLRIFSERR**IWNPTVADITLLALGTCAPQV**

Pp1 **LLYCFVGLA**TITNLFMQAGGSAMGTIANRTRKIVRHNDESGSEEVVHVR**VWNPVIADITLLALGTSAPQI**

Pp2 **IIYCFVGLATITNL**YMEARSSAMEKIVHQTRKVVRHNYEIGSDEIVHER**IWNLVIADITLLALGTSAPQI**

Cons **laYCFiGLsa!ta**r%f....Smenvvkhsr.vv..i#p.tn.evi..ek**!WNyt!AD!.LLafGTsfPQ!**

**out-RE1-out TMS3 loop b (in) TMS4**

At **SLAT**IDAIRNMGER**YAGGLGPGTLVGSAAFDLFPIHAVCVV**VPKAGELKKISD**LGVWLVELVWSFWAYIW**

Ah **SLATI**DAIRNIGER**YAGGLGPGTLVGSAAFDLFPIHAVCVV**VPKAGELKKISD**LGVWLVELVWSFWAYIW**

Al **SLATI**DAIRNIGER**YAGGLGPGTLVGSAAFDLFPIHAVCVV**VPKAGELKKISD**LGVWLVELVWSFWAYIW**

Eg **SLA**TIDALRNLGEL**YAGGLGPGTLVGSAAFDLFPIHAVCVV**VPKSGELKKISD**LAVWAVELFWSFWAYIW**

Cc **SLATI**DAIRSIGNL**YAGGLGPGTLVGSAAFDLFPIHAVCVV**VPKAGELKKISD**IGVWLVELFWSFWAYVW**

Rc **SLATI**DAIRNIGNL**YAGGLGPGTLVGSAAFDLFPIHAVCVV**VPKAGELKKISD**LGVWLVELFWSFWAYVW**

Me **SLATI**DAIRNIGNL**YAGGLGPGTLVGSAAFDLFPIHAVCVV**APKAGELKKIAD**LGVWIVELFWSFWAYIW**

Pt **SLATI**DAIQNIGNL**YAGGMGPGTLVGSAAFDLFPIHAVCVV**VPKAGELKKIAD**IGVWLVELFWSFWAYIW**

Ppe **SLATI**DAIRNLGNL**YAGGLGPGTLVGSAAFDLFPIHAVCVV**VPRAGELKKIAD**VGVWLVELFWSFWAYIW**

Cp **SLATI**DAIRNLGNL**NAGGLGPGTLVGSAAFDLFPIHAVCVV**VPKGGEMKKIAD**IGVWLVELFWSFWAYIW**

Ac **SLATI**DAIRN**LGQIYAGGLGPGTLVGSAAF**DLFPIHAVCVVVPKAGELKKISD**IGVWLVELFWSFWAYIW**

Vv **SLAII**DSIQNLGSR**YAGGLGPGTLVGSAAFDLFPIHAVCVV**VPKAGELKKISDV**GVWLVELFWSFWAYVW**

Gm **SLATI**DAIQNIGKL**YAGGLGPGTLVGSAAFDLFPIHAVCVV**VPKAGELKKIAD**LGVWLVELFWSFWAYIW**

Mt **SLATI**DAIRNLGDL**YAGGLGPGTLVGSAAFDLFPIHAVCVVIP**KAGELKKIAD**LGVWIVELFWSFWAYIW**

St **SLATI**DAIRNIGKL**YAGGLGPGTLVGSAAFDLFPIHAVCVV**VPKAGELKKISD**IGVWLVELFWSFWAYIW**

Sl **SLATI**DAIRNIGKL**YAGGLGPGTLVGSAAFDLFPIHAVCVV**VPKAGELKKISD**IGVWLVELFWSFWAYIW**

Mg1 **SLATI**DAIR**NLGSLYAGGLGPGTLVGSAAF**DLFPIHAVCVVVPKAGELKKISD**VGVWLVELFWSFWAYIW**

Mg2 **SLATI**DAIRN**IGSLTAGGLGPGTLVGSAAF**DLFPIHAVCVVVPKAGELKRISD**IGVWLVELFWSFWAYIW**

Cs **SLATI**DAIRNIGNL**YAGGLGPGTLVGSAAFDLFPIHAVCVV**VPKAGELKKISD**IGVWLVELVWSFWAYIW**

Si **SLA**TIDAIRN**LGQLTAGGLGPGTLVGSAAF**DMFPIHAVCVIMPKAGSKKKITD**LGVWLVELFWSFWAYIW**

Zm **SLA**TIDAIRNL**GQLTAGGLGPGTLVGSAAFDLFPIHAVCVLMPRAG**SKKKISD**LGVWLVELFWSFWAYIW**

Sb **SLAI**IDAIRNLGQL**TAGGLGPGTLVGSAAFDLFPIHAVCVVMPRAGS**KKKISD**LGVWLVELVWSFWAYIW**

Ta **SLA**TIDAIRNLGQL**TAGGLGPGTLVGSAAFDLFPIHAVCV**VMPRAGSMKKISDL**GVWLVELFWSFWAYIW**

Bd **SLA**TIDAIRNLGQ**LTAGGLGPGTLVGSAAFDLFPIHAVCV**IMPRAGSMKKISDLG**VWLVELFWSFWAYIW**

OsJ1 **SLATI**DAIRNLGQL**TAGGLGPGTLVGSAAFDLFPIHAVCVVMPRAG**SKKKISD**LGVWLVELFWSFWAYIW**

OsI1 **SLATI**DAIRNLGQL**TAGGLGPGTLVGSAAFDLFPIHAVCVVMPRAGS**KKKISD**LGVWLVELFWSFWAYIW**

OsJ2 **SLATI**DAIRNLGQL**TAGGLGPGTLVGSAAFDLFPIHAVCVVMPRAGS**KKKISD**LGVWLVELFWSFWAYIW**

OsI2 **SLATI**DTIRNLDQL**TAGGLGPGILVGSAAFDLFPIHAVCVVMP**RAG.KKKISD**LGVWLVELFWSFRAYIW**

Sm **SLAVI**DAFQHIGQ.TSGK**LGAGTLLGSTAFNLFLILAVCVVAP**KRFQTKSIRN**VGVYIIEVVWSFWAYVW**

Pp1 **SLSII**DAIQQIGQKTNAGLGPE**TIVGSAAFNLYPILAVCVLVP**KAGSVKRIQN**VGVWIVELSWSIWAYVW**

Pp2 **SLAII**DAFQQLGQKTEAGLGPG**TIIGSAAFNLYIILAVCVL**VPKAGSTKHIRS**IGVWIVELTWSMWAYIW**

Cons **SLatI**DairnlGql**yagg$GpgTlvGSaAF#$%pIhAVCVv**vPkagelKkIsd**lgVwl!ElfWSfWAY!W**

**loop c (out) TMS5 loop d (in)**

At **LYIILEVW**SPN**VITLVEALLTVLQYGLLLVHAY**AQDKRWPYLSLPMSRGDRPEEWVPEEIDTSKDDNDND

Ah **LYIILEVW**SPN**VITLVEALLTVLQYGLLLVHAY**AQDKRWPYLSLPMSRGDRPEEWVPEEIDTSKDDNDND

Al **LYIILEVW**SPN**VITLVEALLTVLQYGLLLVHAY**AQDKRWPYLSLPMSRGDRPEEWVPEEIDTSKDENDND

Eg **LYIILKVW**TPD**IVTLWEALLTVLQFGLLLVHAY**AQDKRWPYLSIPLARGERPEEWVPEEAASYKHVDNVD

Cc **LYIILEVW**TPNV**ITFWEALLTVLQYGLLLIHAY**AQDKRWPYLSLPIARTERPEDWVPQETTSCKDENCDY

Rc **LYIILEVW**TPNV**ITLWEALLTVLQYGLLLTHAY**AQDKRWPYLSLPIERTERPEEWVPEEATSDKHQHNAY

Me **LYIILEVW**TPNV**ITLWEALVTVLQYGLLLIHAY**AQDKRWSYLSLPLGRTERPKEWVPEEITSSKHQPIDY

Pt **LYIILEVW**TPNV**ITLWEALLTVLQYGLLLMHAY**AQDKRWPYLSLPIPRTERPEEWVPGEVPSPTHENNVY

Ppe **LYIILEVW**TPNV**ITIWEALLTVLQYGLLLMHAY**AQDKRWPYFSLPLPRSERPEDWVPAEVVTCKSDSGPC

Cp **LYIILEVW**TPNV**VTLWEALLTVLQYGLLLTHAY**AQDKRWPYLSLPIGRTERPEDWVPEEVASVDHDNNDK

Ac **LYIILEVW**TPNV**VTLWEALITVLQFGLLLIHTY**AQDKRWPYLSLPLARTERPEDWVPVEASLDKLDNNDY

Vv **LYIILEVW**TPNV**ITIWEALLTVLQFGLLLIHAY**AQDKQWPYLSLPLPRTERPEDWVPDEAASRKQDKIAG

Gm **LYIILEVW**TPNV**VTLWEALLTVLQYGLLLTHAY**AQDKRWPYISLPIARDERPEDWVPEETPYFQHEAHAE

Mt **LYIILEVW**TPNV**ITLWEALLTVLQYGLLLIHAY**AQDKRWPYISLPIARDERPEDWVPEETPKQKS..HER

St **LYIILEVW**TPNV**VTLWESILTVLQFGLLLIHAY**AQDKRWPYLSLPLERAERPEEWVPAEVVKHRPLDKVH

Sl **LYIILEVW**TPNV**VTLWESILTVLQFGLLLIHAY**AQDKRWPYLSLPLERAERPEEWVPAEVVKYRPLDKVH

Mg1 **LYIILEVW**TPN**IITLLEALLTVLQFGLLLIHAY**AQDKRWPYLSLPIGRTERPEEWVPVETAPYKDRDKYS

Mg2 **LYIILKIW**TPNV**ITLWEALLTVAQFGLLLIHAY**AQDKRWPYVSLPMTRSERPEDWVPAE..KNAPYRDGN

Cs **LYIILEVW**TPKV**ITLWEALLTVLQYGLLLTHAY**AQDKRWPYLSLPLARTERPEEWVPPEI....DICKQD

Si **LYVILEVW**TPK**VITVWEALLTVLQYGLLLLHAY**AQDKRWPYVSIPLVRGDRPEDWVPEEGAS.VDYDNCN

Zm **LYVILEVW**TPRV**ITLWEASLTVLQYGLLLLHAY**AQDKRCPFVSIPFARGERPEDWVPPEDVSAVYYAKCD

Sb **LYVILEVW**TPKV**ITLWEALLTVLQYGLLLLHAY**AQDKRWPFVSIPFVRGERPEDWVPPEDVS.VDYDNCD

Ta **LYIILEVW**TPNV**ITLWEALLTVLQYGLLLVHAY**AQDKRWPYVSIPLVRGERPEDWVPAEDTSLHHDKNCD

Bd **LYIIL**EVWTPNVIT**LWEALLTVLQYGLLLVHAY**AQDKRWPYVSIPLVRGERPEDWVPAEDTSVDHDKNCG

OsJ1 **LYIILEVW**TPRV**ITLWEALLTVLQYGLLLLHAY**AQDKRWPYVSIPLARGERPEDWVPAEDASVDYDDNYD

OsI1 **LYIILEVW**TPRV**ITLWEALLTVLQYGLLLLHAY**AQDKRWPYVSIPLARGERPEDWVPAEDASVDYDDNYD

OsJ2 **LYIILEVW**TPRV**ITLWEALLTVLQYGLLLLHAY**AQDKRWPYVSIPLARGDRPEDWVPTEDASVDYDDNYD

OsI2 **LYIIL**EVWTPRV**ITLWEALLTVLQYGLLLLHAY**AQDKRWPYVSIPLARGDRPEDWVPTEDASVDYDDNYD

Sm **LFLILEIW**TPNE**ITLWEAVLTVAQFPLLVLHAY**VQDRNWKYLSIPLRESDAIESSDMNNLHELAN.....

Pp1 **LAIILQVS**SPNVVEPWEAMCTVLQFPILMTHAYIQDKGWGNFCRPLWPCMHVSWSPLKDFSLSVHNGTVH

Pp2 **LAIILQVS**SPDVVEPWEAVCTVLQFPILMMHAYVQDVGLETLCCPS...MIVSWKPIRKYFSLAIYDGEI

Cons **LyiILe!w**tPnv**!tlwEallTVlQ%glLliHaY**aQDkrwpylslPl.r.erpedwvp.e..s...ddn..

At VHDVYSDAAQDAVESGSRNIVDIFSIHSANNDTGITYHTVADTPPDSATKKGKAKN..............

Ah VHDVYSDAAQEAVESGSRNIVDIFSIHSANNDTGITYHTVADTPPDSATKKGKAKN..............

Al VHDVYSDAAQEAVESGSRNIVDIFSIHSANNNTGITYHTVADTPPDSATKKGKAKN..............

Eg GPMYHNVPASDLAESSNELHVLAL..............................................

Cc DEC...SEILQLGEDENRGVVDIFSIHSPNGAVTSPLYQKVPGSEDVAEISKESFRE.............

Rc EEY...SEIVQVSEEDSRNIVDIFSIHSPVG..TDPVYQKVPETDEAAESSNNYSLS.............

Me EEY...SEILQVDKDESRNVVDIFSIHSNVE..TDQVYHKVPETDDAVEFSDKNFHS.............

Pt GE.............ENRNVVDIFSIHSNNG..T..VYQKVPVTDDAAESSNKHFHQ.............

Ppe NNY...SEILEVGEDENRNIVDIFSFHSGSGLG..PVYQNVPGTDETPEYSNKDSPE.............

Cp F..................VVDIFSIHSAP....STEYQKVPADENIPEPSGKSSRK.............

Ac SEI...LQLSE....ED.NIVDIFSIHSEKETGPLYQPVSNS...DVAESSNMNFHN.............

Vv DEC...SELFQ....ENGNIVDIFSIHSGD..GSVYHRISGS...EVAEPSNEHFQK.............

Gm VDF...SDIKHVNE.ENGDTVDIFSIHSENPTDPLYARVPSIND....EAEILDKAK.............

Mt VEC...SEINHFNE.ENGDTVDIFSIHSENPRDMSYVRVPQIDD.....AENSDKVI.............

St EPH...SEVSQVGEEENSGIVDIFSIH.SGEGTGHFYRN...LAGEDVTESSTPNNG.............

Sl EPH...SEVSQVGEEENTGIVDIFSIH.SGEGTGHFYQN...LAGEDVTESSTPNNC.............

Mg1 EIP...EDDHESGR.....IVDIFSVH.SD....HVYQN...LSGSDSGEPSGQSHE.............

Mg2 KTR...DNYPEVDEDRNSIIVDIFSIH.SANTGFVYENV...ADTDIHESSSEQCRV.............

Cs NPC...REEFQAHENEQRSIVDIFSIHDSDGKVYHEVPG...HDIAESSNSNIPEEM.............

Si ET....SEILPGSSDKD..IADIFSGHSYHNA.EYHKV....PENDMECSSTMNNFV.............

Zm GI....DETLPMGADGNDGIVDIFSAHSYYDAAEYSQL....AEEDMEGSSTMDQVV.............

Sb DT....NETLPISANRNDGIVDVFSAHSYHIA.EYSRV....PEKDMEGSSTMDQVV.............

Ta EN....SDILPSE...NDDVVDIFSIHSYSNAG.YHHV....PEKDIEESSKTTLVV.............

Bd ES....SEILPRE...NEDIVDIFSMHSYNNTG.YHHV....PEKDIEESSKTTLVV.............

OsJ1 GI....GDILPGQ...NEDIVDIFSAHSYSNEG.YHHV....SEEDVEESS.TGLTL.............

OsI1 GI....GDILPGQ...NEDIVDIFSAHSYSNEG.YHHV....SEEDVEESS.TGLTL.............

OsJ2 GI....GDILPGQ...NEDIVDIFSARSYSNEG.YHHV....SEKDVEESP.TGLTL.............

OsI2 GI....GDILPGQ...NEDIVDIFSAHSYSNEG.YHHV....SEKDVEESP.TGLTL.............

Sm .....................................VSRSSKFAAMRELVATRPHNFRLFFFSSRAEDV

Pp1 DQSTAYSSSPRVDLEQHRSEWELVDLQHQPKQEIRNRVTCDDHPIKPIEWQQVAVAVDKNEDENDPG...

Pp2 HDQSASFSNLEVFSEQSTSDLERVDLQYLPMKEIRNGVTFDDHRISPFERQQVIGANDISEQLTCGSKNS

Cons .......e..e.....n.nivdifsihs..n..............d..e.s...................

At ......................STVFDIWKHQFVDAITLETSESKKVDSIYLRIAKSFWHLLLAPWKLLF

Ah ......................SSVFDIWKHQFVDAITLETSESKKVDSIYLRIANSFWQLLLAPWKLLF

Al ......................STVFGIWKHQFVDAITLETSESKKVDSIYLRIAKSFWQLLLAPWKLLF

Eg ............................WKEQFVDALMLESAETKKLYSIYVRALRMLWQSLLAPWRLMF

Cc ................EINSELPHVHALWKQQFVDSIALESPESRKMNNIYLRLARIFWQSLLVPWRVLF

Rc ................EKDL...DVVALWKQQFVDAIMLERPESRKLINSHLRLARISWQLFLVPWRLLF

Me ................EQDF...NVAALWKRQFLDAITLESLESRNMFSIHIRLARTFWEILLAPWRLLF

Pt ................EKDL...HVLSLWKEQFVDALTLESLETRKLNSIHLRVARISWQLILAPWRLLF

Ppe ................KMSLEDYHVFAIWGQQFVDAVKLKSTESRQLNNLYLRLARISWQLLLVPWRLLF

Cp ................EILLEDPNVLTLWKQQFVDIFKVEKSDSRKLDNIYLQLARIVWQSLLAPWRFMF

Ac ................KSVLQDFNVFTVWKHQFMDAVMLENPESRKLDNTYLRLARGTWQVILAPWRVLF

Vv ................NIILEDSHLLSLWKQQFVDAFTLESSESRKLDN**IYLWVARIFWQLLLLPWRFLF**

Gm ................ETTLVDTHLLTIWRQQFLDALRLLRPESKKIKN**ACLRLACIFWQLLILPWRFLF**

Mt ................ETRLEDTCLLTIWKQQFVDALTVESQESKKMNNIYIRTARIFWQLLLLPWRFMF

St ................NIIPEESDILSIWKHQFVDALMLESTESRKLNNIYLRVARIFWQLLLLPWKLLF

Sl ................YIIPEESDILSIWKHQFVDALMLESTESRKLNNTYLRVARIFWQLLLLPWKLLF

Mg1 ................DIHVKDNLI.SIWKQQFVDAFVLEGQESRKLNNTYLRAAKVLWELLLAPWRILF

Mg2 ................DTIPHKDDLLSVWKMQFVDALTLESPESKKLNSKSLRVAKALWKLVLAPWRLLF

Cs ................DGKADHPHVLKIWKQQFVDALSLETSESKQRNNIYLRSARLCWQLIVAPWRLLF

Si ................KNTREDTSWRSLWRQQFVDAFMLESPESRKMASVCLRLIRIFWNLLIAPWKLFF

Zm ................KNTQDNMSWLSIWWQQFVDASMLESPESMKMDSVCLRYTRIFWNLIIAPWKFLF

Sb ................KNTQEDISWLSIWWQQFVDASMLESPESRKMHS**ICLRFTTIFWNLIIAPWKFLF**

Ta ................KNTQEDIYWLSVWRQQFVDAVMLESPELKKMDPVCLRFIIICWNSIIAPWKLLF

Bd ................KNTQEDTCWLSIWRQQFVDALMLESPEQRKMDSICLR**FIIIFWNSIIAPWKLLF**

OsJ1 ................KNKWEDTHWFSIWWQQFVDAATLESSVSRKMDST**CLRVIGISWNLIIAPWKMLF**

OsI1 ................KNKWEDTHWFSIWWQQFVDAATLESSVSRKMDST**CLRVIGISWNLIIAPWKMLF**

OsJ2 ................KNKWEDTHWFSIWWQQFVDAATLESSVSRKMDST**CLSVIGISWNLIIAPWKMLF**

OsI2 ................KNKWEDTHWFSIWWQQFVDAATLESSVSRKMDSTCLRVIGISWNLIIAPWKMLF

Sm DTSSRDNFAFLREGAETEHFSWKALGTAWKNQFYDAMTVSFFEGRGQRKRTPSTLQLLLHPIVSYWKVSF

Pp1 .SNQEFLKQRLAYDIYEQTSMWTYIYSTWKKQFLDVIVFQGQVDDSGKNLALTAVEFIGYLITLPWRFIF

Pp2 ESNQELVKLRSTNEIDEHTSGRTNACSLWKMQFLDAIFIEKHVDESGKDHSPIAVDCVGHLIILPWRFLF

Cons ....................ed..v.siWkqQFvDa..les.esrkld.i.lr.ari.wqlllapWrllF

**TMS6 loop e (out) TMS7 (in)**

At AFVPPCNIAH**GWIAFICSLLFISGVAFVV**TRFTD**LISCVTGINPYVIAFTALA**SGTSWPDLVASKIAAER

Ah AFVPPCNIAH**GWIAFIFSLLFISGVAFVV**TRFTD**LISCVTGINPYVIAFTALA**SGTSWPDLVASKIAAER

Al AFVPPCNIAH**GWIAFICSLLFISGVAFVV**TRLTD**LISCVTGINPYVIAFTALA**GGTSWPDLVASKIAAER

Eg ALVPPCQIAH**GWFAFICSLTFISGIAY**VVTKLTD**LISCVTGINAYVIAFTALA**TGTSWPDLVASKIAAER

Cc AFVPPYHIAH**GWIAFICSLIFISGIAYIV**TKLTD**IISCVTGINAYVIAFTALA**SGTSWPDLVASKIAAER

Rc AFVPPYHIAH**GWIAFICSLLFISGIAYIV**TQLTD**LISCVTGINAYVIAFTALAA**GTSWPDLVASKIAAER

Me AFVPPYHIAH**GWVAFICSLIFISGIAYIV**TKITD**LISCVTGINAYVIAFTALA**SGSSWPDLVASKIAAER

Pt AFVPPYHFAH**GWIAFIFSLLFISGIAYIV**TKLTD**VISCVTGINAYVIAFTALA**SGTSWPDLVASKIAAER

Ppe AFVPPYHIAH**GWIAFICSLVFISAIAYIV**TQLTD**LISCVTGINPYVIAFTALA**SGTSWPDLVASKIAAER

Cp AFVPPYHISH**GWIAFVCSLLFISGISYIV**TKLTD**LVSCVTGINPYVIAFTALA**CGTSWPDLVASKIAVER

Ac AFVPPCHIAH**GWIAFICSLIFISGIAYIV**TKLTD**MISCVTGINGYVIAFTALA**SGTSWPDLVASKIAAER

Vv **AF**VPPPHIAH**GWFAFICSLIFISGIAYIVT**KLTDLISCTS**GINSYVIAFTALAAGTSWPDLV**ASKIAAER

Gm **AF**VPPCQIAH**GWISFICSLLFISGIAYVVT**KITD**VISCVTGINAYVIAFTALASG**TSWPDLVASKIAAER

Mt AFVPPCHIAH**GWISFICSLLFISGIAYIV**TKITD**LISCVTGINAYVIAFTALA**SGTSWPDLVASKIAAKR

St AFVPPYQIAH**GWIAFICSLIFISGIAYVV**TKITD**LISCVTGINPYVIAFTALA**SGTSWPDLVASKIAAER

Sl AFVPPYQIAH**GWIAFICSLIFISGIAYVV**TKITD**LISCVTGINPYVIAFTALA**SGTSWPDLVASKIAAER

Mg1 AFVPPCQIAH**GWISFISSLIFISGIAY**VVTKLTD**IISCVTGINAYVIAFTALAA**GTSWPDLVASKIAAER

Mg2 AFVPPYQ**IANGWIAFIFSLVFISGIAYIV**TKITD**LISCVTGINAYIIALTALA**AGTSWPDLVASKIAAER

Cs AFVPPYHIAH**GWVAFICSLMFISGIAYVL**TKFTD**LISCVSGINPYVIAFTALA**SGTSWPDLVASKIAAER

Si AFVPPYHIAH**GWVAFICSLIFISGIAY**GVTKLTDQIS**CVTGVSPYVIAFTALAAGTSWPDLV**ASKIAAER

Zm AFVPPYHIAH**GWIAFICSLIFISGIAY**GVTKLTDQISC**VTGVSPYVIAFTALAAGTSWPDLV**ASKIAAER

Sb **AF**VPPYNIAH**GWIAFICSLIFISGIAY**GVTKLTDQISC**VTGVSPYVIAFTALAAGTS**WPDLVASKIAAER

Ta AFVPPYQIAH**GWIAFIFSLIFISGIAYGV**TNITDQISCVTGLN**PYVIAFTALAAGTSWPDLV**ASKIAAER

Bd **A**FVPPYQIAH**GWIAFICSLIFISGIAY**GVTKITDQ**ISCVTGVNLYVIAFTALAA**GTSWPDLVASKIAAER

OsJ1 **AF**VPPYEIAH**GWIAFICSLIFISGIAY**GVTKITDQISC**VTGVSPYVIAFTALAAGTS**WPDLVASKIAAER

OsI1 **AF**VPPYEIAH**GWIAFICSLIFISGIAY**GVTKITDQISC**VTGVSPYVIAFTALAAGT**SWPDLVASKIAAER

OsJ2 **AF**IPPYEIAH**GWIAFICSLIFISGIAY**GVTKITDQISC**VTGVSPYVIAFTALAAGTS**WPDLVASKIAAER

OsI2 AFIPPYEIAH**GWIAFICSLIFISGIAY**GVTKITDQIS**CVTGVSPYVIAFTALAAGTSWPDLV**ASKIAAER

Sm AFIPPVQLLH**GWLAFLGSICFITWISYV**VVALSNRINCVTGISSYV**LALTVLAAGTSLPNLMAS**KIAAEE

Pp1 AFLPPPSLLH**GWAAFLCALAHITVIACFLI**KLT**NLFGCVTGISKYTLALTVLAAGTSLPDLI**ASKIAAEI

Pp2 AFLPPPMLLNGWP**AFMCALAFITVISCFLI**KLANSFGC**VTGVSDYVLALTILAVG**TSWPDLIASKIAAKH

Cons AfvPPyqiah**GWiaFicsl**i**fIsg!ayvv**tklt#**lisCvtGinpYviAfTaLA.**GtSwP#LvASKIAaer

**RE-loop 2** **(in)** **TMS8**

At QLTADSAIANITCSN**SVNIYVGIGVPWLINTVYNYF**AYREPLYIENAKGLS**FSLLIFFATSVGCIVVLVL**

Ah QLTADSAIANITCSN**SVNIYVGIGVPWLINTVYNYF**AYREPLYIENAKGLS**FSLLIFFATSVGCIVVLVL**

Al QLTADSAIANITCSN**SVNIYVGIGVPWLINTVYNYF**AYREPLYIENAKGLS**FSLLIFFATSVGCIVVLVL**

Eg QTTADSAIANITCSN**SVNIYVGIGVPWLINTAYNFI**MYREPLRIQNAAGLS**FSLIVFFSTSVGCISVLVL**

Cc QITADSAIAN**ITCSNSVNIYVGIGIPWLI**DTAYNFIVYKEPLRVQNAEGLS**FSLLVFFCTSVGCIAVLVL**

Rc QITADSAIANITCSNSVNI**YVGIGVPWLIDTAYNFFAYREPLRVQ**DAAGLS**FSLLVFFSTSVGCIAVLVY**

Me QTTADSAIAN**ITCSNSVNIYVGIGVPWLI**DTTYNFFVYRQPLRIENAEGLS**FSLLVFFATSVGCIGVLVY**

Pt QTTADSAIAN**ITCSNSVNIYVGIGVPWLIDTAYNFFMY**NEPLRIQNAAGLS**FSLLVFFCTSIGCIAVLVL**

Ppe QITADSAIAN**ITCSNSVNIYVGIGVPWLIDTAYNFF**VYKEPLRIENAAGLS**FSLLVFFSTSVGCIAVLVI**

Cp QITADSAIANITCSN**SVNIYVGIGVPWLINTLYNFF**AYREPLGIQNAEGLS**FSLLVFFSTSVGCIAVLVF**

Ac QTTADSAIAN**ITCSNSVNIYIGIGIPWLI**DTTYNFIAYREPLRIENADGLS**FSLLIFFLTSIGCISVLVL**

Vv QTTADSAIANIICSNSVNIY**MGIGIPWLINTTYNFIA**YREPLGVQNAEGLSF**SLLVFFCTSICCIGVLVL**

Gm QKTADSAIANITCSNSVN**IYVGIGVPWLIDTLYNFIAY**REPLR**IQNAGGLSFSLIVFFSTSVGCISVLVL**

Mt QKTADS**AIANITCSNSVNIYVGIGVPWLI**DTLYNFIAYRQPLRIQNAGGLS**FSLIVFFATSVGCISVLVA**

St QLTADSAIANITCSN**SVNIYIGIGVPWLINTLYNYI**AYNEPLRIDNAEGLS**FSLLVFFSTSVACIGVLVF**

Sl QLTADS**AIANITCSNSVNIYIGIGVPWLI**DTLYNYIAYNEPLRIENAEGLS**FSLLVFFSTSVACIGVLVF**

Mg1 QTTADSAIANITCSNS**VNIYVGIGVPWLINTMYNYF**AYNEPLRIENAGG**LSFSLLVFFATSIGCIGGLVF**

Mg2 QITADS**AIANITCSNSVNIYIGIGVPWLI**DTLYNYIAYKKPLRIENAAGLS**FSLLIFFATSVGCIGVLVF**

Cs QTTADSAIANITCSNSVN**IYVGIGVPWLISTTYNFI**AYKEPLKIKDAGGLS**FSLLVFFSTSVACIVVLVF**

Si QVTADSAIAN**ITCSNSVNIYVGIGVPWLIDTVYNFFVY**REPLYIDNAAGLS**FSLLVFFATSFGCITVLVL**

Zm QVTADSAIAN**ITCSNSVNIYVGIGVPWLIDTVYNFFVY**REPLYIDNAAGLS**FSLLVFFATSFGCITVLVL**

Sb QVTADSAIANITCSNSVN**IYVGIGVPWLIDTVYNF**FVYQEPLYIDN**AAGLSFSLLVFFATSFGCITVLVL**

Ta QVTADSAIANITCSN**SVNIYVGIGVPWLINTVYNFF**AYQEPLYIDNAAGLS**FSLLVFFATSFGCITVLVL**

Bd QVTADSAIAN**ITCSNSVNIYVGIGVPWLI**DTVYNYFVYQEPLYIDNAAGLS**FSLLVFFATSFGCITVLVL**

OsJ1 QITADSAIT**NITCSNSVNIYVGIGVPWLVDTMYNY**FVYQKPLYIDNA**AGLSFSLLVFFATSFGCITVLVL**

OsI1 QITADSAIT**NITCSNSVNIYVGIGVPWLVDTMYNY**FVYQKPLYIDN**AAGLSFSLLVFFATSFGCITVLVL**

OsJ2 QITADSAIA**NITCSNSVNIYVGIGVPWLVDTMYNYFV**YKKPLYIDN**AAGLSFSLLVFFATSFGCITVLV**L

OsI2 QITADS**AIANITCSNSVNIYVGIGVPWLV**DTMYNYFVYKKPLYIDNAAGLS**FSLLVFFATSFGCITVLVL**

Sm YDTADSAIANINASNCIN**VYVGFGVPWCVSALYSTG**FHQNLV.VPVE.GLK**FLLSVYFSTAITCFVAL**TA

Pp1 QPTADSAIANIN**ASNCINVYVGIGIPWLMQSF**YNWIHLKEEFRVPSA.GLG**FALVLFFVTFAICLVVIIA**

Pp2 LPTADSAIANINASNCINVYVGTGIPWLLQSFYNKLQLDEEFRVPS**V.GIGFSLMLFLVTFVLCQIVVV**G

Cons q.TADSAIaNItcSNs!N!YvGiG!PWlidt.Ynffay.eplr!ena.Gls**FsLlv%faTsvgCi.vlvl**

**loop f (out)** **TMS9 C-ter (in)**

At RRLIIGAELGGPR**LWAWLTSAYFMMLWVVFVVLSSL**KVSGVI

Ah RRLIIGAELGGPR**LWAWLTSAYFMMLWVVFVVLSSL**KVSGVI

Al RRLIIGAELGGPR**LWAWLTSAYFMMLWVVFVVLSSL**KVSGII

Eg RRLTLGAELGGPKPW**AWLTSVYLMLLWVIFLVLSSL**KVSGII

Cc RRLTLGAELGGPK**LWAWVTSVYFMLLWIIFVVLSSL**KVSGII

Rc RRLTLGAELGGPR**IWAWVTSVYFMFLWLIFVVLSSL**RVSGII

Me RRLTLGAELGGPR**IWAWVTCVYFMLLWLIFVVLSSL**RVSGFI

Pt RRLTLKAELGGPR**IWAWVTFVYFMLLWIIFVVL**SSLRVSGII

Ppe RRRTLGAELGGPRL**WAWITFVFFMLLWLIFVVL**SSLKVSGII

Cp RRLTLGAELGGPRF**WAWVTCVYFMLLWIIFVVLSSL**RVSGII

Ac RRLTIGAELGGPR**IWAWITSVYFMSLWIVFVVL**SSLKVSDII

Vv RRLTLGAELGGPKIWAR**LTCVYFMSLWIIFVVLSSLKIYGII**

Gm RRIIFGAELGGPRLW**AWITCAFFMLLWIIFVVLSSL**KVSGFI

Mt RRIVFGAELGGPRL**WAWITCAFFMLLWIIFVVL**SSLKVSGFI

St RRLTIGAELGGPRV**WAWVTCIFFMLLWLIFVVL**SSLRVSGII

Sl RRLTIGAELGGPRV**WAWVTCIFFMLLWLIFVVL**SSLRVSGII

Mg1 RRLTLGAELGGPKI**WAWITCAYFMFLWLIFVVLSSL**RVSGFI

Mg2 RRLTLGAELGGPRH**WAWLTCFYLMLLWLVFVV**LSSLQVSNII

Cs RRVTLGAELGGPK**VWAWITCIFFMVLWVIFVVL**SSLKVSDII

Si RRIIIGAELGGPR**LWAWITSVYFMILWIVFVVFSSL**KVYGII

Zm RRIILGAELGGPRL**WAWVTSVYFMILWVVFVVFSSL**RVSGVI

Sb RRIILGAELGGPR**LWAWVTSVYFMILWVVFVVFSSL**RVSGVI

Ta RRIVFGAELGGPRL**WAWATSAYFMVLWVVFVLLSSL**RVSGVI

Bd RRIVLGAELGGPR**LWAWVTSTYFMVLWVVFVVLSSL**RVSGVI

OsJ1 RRVILGAELGGPR**MWAWATSVYFMILWVVFVVLSSL**KISGVI

OsI1 RRVILGAELGGPR**MWAWATSVYFMILWVVFVVLSSL**KISGVI

OsJ2 RRVILGAELGGPR**MWAWATSVYFMILWVVFVVLSSL**RISGVI

OsI2 RRVILGAELGGPRMWAWATSVY....................

Sm RRRLLGGELGGPRK**WAWASAIFFFLLWITFLTLACLAG**RFT.

Pp1 RRFLFGGELGGPRK**WAWVSSFCFLSLWLIFVIFSCL**RNYHHL

Pp2 RRFIFEGELGGPRK**WAWASSFYFIFLWLIFVIFSCL**RNYNLL

Cons RRl.lgaELGGPrl**WAwvtsvyfmlLWviFvvlssL**.vsgii
